# Composition and Functional Characteristics and Influencing Factors of Bacterioplankton Community in the Huangshui River, China

**DOI:** 10.3390/microorganisms9112260

**Published:** 2021-10-29

**Authors:** Qianqian Zhang, Zhenbing Wu, Juan Zhao, Guojie Wang, Jingwen Hao, Shuyi Wang, Yaoyao Lin, Hongtao Guan, Jinyong Zhang, Shenglong Jian, Aihua Li

**Affiliations:** 1 State Key Laboratory of Freshwater Ecology and Biotechnology, Institute of Hydrobiology, Chinese Academy of Sciences, Wuhan 430072, China; zqq@ihb.ac.cn (Q.Z.); wuzhenbing@ihb.ac.cn (Z.W.); haojingwen@ihb.ac.cn (J.H.); wangsy@ihb.ac.cn (S.W.); linyaoyao@ihb.ac.cn (Y.L.); 2 College of Life Sciences, University of Chinese Academy of Sciences, Beijing 100049, China; 3 Qinghai Provincial Fishery Environmental Monitoring Center, Xining 810012, China; qhshnchxl@163.com (J.Z.); wangguojie178@163.com (G.W.); ght13139058581@163.com (H.G.); 4 Key Laboratory of Plateau Aquatic and Ecological Environmental in Qinghai Province, Xining 810012, China; 5 The Laboratory of Aquatic Parasitology, School of Marine Science and Engineering, Qingdao Agricultural University, Qingdao 266237, China; zhangjy@ihb.ac.cn

**Keywords:** Huangshui River, high-throughput sequencing, bacterioplankton community structure, environment factors, PICRUSt2 analysis

## Abstract

Bacterial community plays a key role in environmental and ecological processes of river ecosystems. Given the special climatic and geographical conditions, studying the compositional characteristics of microorganisms in highland rivers and the relationship between such microorganisms and water physicochemical factors is important for an in-depth understanding of microbial ecological mechanisms. In the present study, high-throughput sequencing was used to investigate and study the bacterioplankton community of the Huangshui River in the ecotone zone of the Qinghai Plateau and Loess Plateau. The results showed that the Huangshui River had significantly lower alpha diversity than the plain rivers. Despite the similarity in their environmental conditions, the main taxonomic compositions of the bacterial communities were distinct between the Huangshui River and polar regions (the Arctic and Antarctica). Proteobacteria accounted for the largest proportion (30.79–99.98%) of all the sequences, followed by Firmicutes (0–49.38%). *Acidiphilium* was the most numerous genera, which accounted for 0.03–86.16% of the assigned 16S reads, followed by *Acidocella* (0–95.9%), both belonging to Alphaproteobacteria. The diverse taxa of potential pathogens, such as Acinetobacter, Pseudomonas, and Aeromonas, were also identified. A principal coordinates analysis, coupled with a canonical correspondence analysis, showed spatial variations in the bacterial community composition. The water physical properties (e.g., Cr^6+^, total phosphorus, and COD_Mn_); altitude; and land use (e.g., urban land cover and aquaculture) determined the distribution of the bacterioplankton composition. PICRUSt2 revealed that the overall functional profiles of the bacterial communities in different samples were similar, and our results suggested the potential health risks of water sources in this area. This work provided valuable insight into the composition of the plankton bacterial community and its relationship with the environmental factors in the Huangshui River in the ecotone zone of the Qinghai Plateau and Loess Plateau and a theoretical foundation for ecological health management.

## 1. Introduction

Microorganisms are primary decomposers of organic compounds in natural ecosystems, which play a role in regional climate formation, the geochemical cycle, biological evolution, and natural ecosystems [1]. The distribution of microorganisms has attracted increasing interest in the last decade, and the diversity patterns of microbial communities have been widely observed [2]. Bacterioplanktons are essential for aquatic ecosystems [3], which are a fundamental and highly variable component of river ecosystems, exhibiting a comprehensive response to environmental pressure and disturbances [4,5,6,7,8,9]. Changes in the river water quality and ecological environment caused by human activities will lead to variations in the microbial community structure [10]. Several environmental factors have been considered as potential determinants of bacterial community compositions, including organic contaminants [11] and the concentration of nitrogen [12], phosphorous [13], and heavy metals [14]. By analyzing the changes in the microbial community structure, the state of a river ecosystem’s health can be evaluated [15]. Therefore, investigating the diversity and structure of bacterial communities is important to understanding the response of bacteria to elevated environmental pressure in plateau rivers.

Qinghai Province, as the “Chinese water tower”, is located in the west of China and in the northeastern part of the Qinghai Plateau. Its river ecosystem plays an important role in the ecological status of the whole province and even the whole of China. The Huangshui Basin is located in the transition zone between the Qinghai Plateau and the Loess Plateau, and it consists of the main stream area of the Huangshui River and the tributary Datong River. Given its particularity, the plateau has been considered as a sensitive area of the natural ecosystem, and its ecological environment is relatively fragile. Since the 20th century, the rapid development of human activities in industry, agriculture, mining, and overgrazing has caused outstanding problems in the aquatic environment, including the reduction of water resources, deterioration of water quality, and decline of aquatic biodiversity. Therefore, the ecological and environmental issues of plateau rivers need urgent attention.

Macrobenthic community compositions in the upper reaches of the Huangshui River have been reported. These works have only provided the community structure of macrobenthos, assessments of ecosystem health, and the quality of water in the Huangshui River [16,17]. However, bacteria play a critical role in environmental and ecological processes of river ecosystems. Exploring the composition of aquatic planktonic bacteria can provide a comprehensive understanding of accurate microbial distribution patterns and of the Huangshui River.

Here, as a complement to previous studies, 16S rRNA gene high-throughput sequencing was conducted in the upper and middle reaches of the Huangshui River. The current study aimed to explore the composition and functional characteristics of the Huangshui River, identify key environmental driving factors, and understand the association of such driving factors with the bacterioplankton community. Based on previous reports, this study was the first to investigate the bacterioplankton community and its influencing factors in the Huangshui River.

## 2. Materials and Methods

### 2.1. Study Sites and Sample Collection

The Huangshui River, which is the largest tributary of the upper reaches of the Yellow River, is located in the intersecting zone of the Qinghai–Tibet Plateau and Loess Plateau (36°02′–38°20′ N, 98°54′–103°24′ E), at an altitude of 1650 to 4400 m, originating from the Baohutu Mountain in Haibei County, Qinghai Province and flowing into the Yellow River in Gansu Province [18]. The terrain in the basin is high in the northwest and low in the southeast, with high mountains and deep valleys. The climate belongs to the plateau arid and semi-arid continental climates, with vertical changes and large regional differences because of the diversity of the local topography. The temperature decreases as it goes upstream, whereas the precipitation increases, and the evaporation decreases. Moreover, humid marshes are found. The average annual temperature in the basin is 0.6–7.9 °C, and the average annual precipitation is 360–540 mm [19].

**Figure 1:**
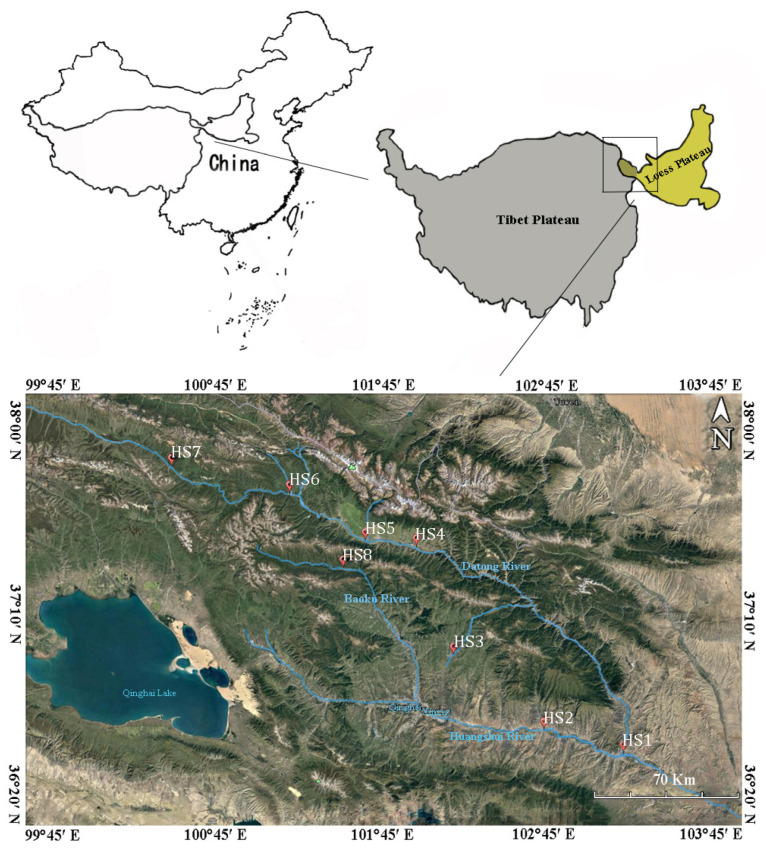
Distribution of the sampling sites in the Huangshui River. Map image and geographical information were cited from Google Earth Pro (version 7.1.8.3036).

The Baoku River is located in the northeast of Qinghai Province, and it is the headwater of the Huangshui River. The Datong River is located in the Qilian Mountains of the Northeastern Qinghai–Tibet Plateau, and it is the primary stream of the Huangshui River. Eight sampling sites (HS1–HS8) were collected along the midstream of the Hungshui River from 8 June to 19 June 2019 (Figure 1). Specific latitude and longitude information about each sampling site was shown as follows: HS1 (36°20′28.9″ N, 102°50′07.4″ E), HS2 (36°28′39.2″ N, 102°25′10.8″ E), HS3 (36°50′07.0″ N, 101°58′00.5″ E), HS4 (36°19′20.4″ N, 101°48′57.8″ E), HS5 (37°21′54.7″ N, 101°32′38.7″ E), HS6 (37°35′45.5″ N, 101°09′18.4″ E), HS7 (37°44′53.8″ N, 100°31′38.3″ E), and HS8 (37°15′09.3″ N, 101°24′51.8″ E). Two liters of water samples were collected 2 m from the middle point of the river. One liter of water samples was filtered through a 20-μm mesh (Millipore Corporation, Billerica, MA, USA) to remove large particles or organisms and subsequently filtered through 0.22-μm polycarbonate membranes (Millipore Corporation, Billerica, MA, USA). The filters were frozen at −80 °C until further processing. The remaining water samples were transported to the laboratory in dark cooling boxes and processed 3–5 h after sampling within 4 h for immediate physicochemical analysis.

### 2.2. Physicochemical Analyses

Water chemistries such as the temperature, pH, and dissolved oxygen (DO) were monitored in situ using YSI Pro2030 (YSI Incorporated, Yellow Springs, OH, USA). The total phosphorus (TP); total nitrogen (TN); permanganate index (COD_Mn_); NH_4_^+^-N; As; and metal ions (Cr^6+^, Cu, Zn, Pb, Cd, and Hg) were analyzed at the Qinghai Fishery Environment Monitoring Station. TP, TN, and Cr^6+^ were analyzed using a dual–beam ultraviolet (UV)–visible spectrophotometer (TU–1901, Persee, Beijing, China). As and Hg were analyzed using an atomic fluorescence photometer (AFS–230E, HaiGuang, Beijing, China), and the metal ions were determined using a graphite furnace flame atomic absorption spectrophotometer (AA6300, Shimadzu, Kyoto, Japan). Many environmental factors are related to the distribution of planktonic bacterial communities, but most of these factors have strong contributory relationships. The environmental factors screened in this study were pH, altitude, TN, TP, NH_4_^+^-N, COD_Mn_, and Cr^6+^.

### 2.3. DNA Extraction, Polymerase Chain Reaction, and Illumina Miseq Sequencing

Microbial community genomic DNA was extracted from the water samples using the E.Z.N.A.^®^ water DNA Kit (Omega Bio-tek, Norcross, GA, USA) in accordance with the manufacturer′s instructions. The final DNA concentration and purity were determined by a NanoDrop 2000 UV–vis spectrophotometer (Thermo Scientific, Wilmington, NC, USA), and the DNA quality was checked by 1% agarose gel electrophoresis. The V3–V4 hypervariable regions of the bacteria 16S rRNA gene were amplified with primers 338F (5′–ACTCCTACGGGAGGCAGCAG–3′) and 806R (5′–GGACTACHVGGGTWTCTAAT–3′) by a thermocycler PCR system (GeneAmp 9700, ABI, Waltham, MA, USA). The PCR reactions were conducted using the following program: 3 min of denaturation at 95 °C, 27 cycles of 30 s at 95 °C, 30 s for annealing at 55 °C, 45 s for elongation at 72 °C, and a final extension at 72 °C for 10 min. The PCR reactions were performed in triplicate using 20-μL mixtures containing 4 μL of 5 × FastPfu buffer, 2 μL of 2.5-mM dNTPs, 0.8 μL of each primer (5 μM), 0.4 μL of FastPfu polymerase, and 10 ng of template DNA. The resulting PCR products were extracted from 2% agarose gel, further purified using the AxyPrep DNA Gel Extraction Kit (Axygen Biosciences, Union City, CA, USA), and quantified using QuantiFluor™-ST (Promega, Madison, WI, USA), according to the manufacturer′s protocols.

Purified amplicons were pooled equimolarly and paired-end sequenced (2 × 300) on an Illumina MiSeq platform (Illumina, San Diego, CA, USA), according to the standard protocols by Majorbio Bio-Pharm Technology Co., Ltd. (Shanghai, China).

### 2.4. Processing of Sequencing Data

Raw fastq files were demultiplexed, quality-filtered by trimmomatic, and merged by FLASH with the following criteria: (i) the 300-bp reads were truncated at any site receiving an average quality score of <20 over a 50-bp sliding window, and the truncated reads shorter than 50 bp were discarded; reads containing ambiguous characters were also discarded; (ii) only overlapping sequences longer than 10 bp were assembled according to their overlapped sequence. The maximum mismatch ratio of the overlap region was 0.2. Reads that could not be assembled were discarded; (iii) samples were distinguished according to the barcode and primers, and the sequence direction was adjusted and exact barcode matched, with 2 nucleotide mismatches in the primer matching.

Operational taxonomic units (OTUs) with 97% similarity cutoff [20,21] were clustered using UPARSE version 7.1 [20], and chimeric sequences were identified and removed. The taxonomy of each OTU representative sequence was analyzed by RDP Classifier version 2.2 [22] against the 16S rRNA database (SILVA SSU 132) using a confidence threshold of 0.7.

### 2.5. Statistical Analysis and Functional Prediction

All samples were randomly resampled to 56,234 reads on the basis of the minimum number of sequences to eliminate the effect of the sequencing depth on subsequent analyses [23]. We evaluated the alpha diversity using the indices of the observed richness (OTUs) and Chao [24], the diversity indices of Shannon and Simpson [25], and Good′s coverage. Nonmetric multidimensional scaling was performed on the OTU data using Bray–Curtis distance matrices to examine the differences in bacterial community among the water samples. The environmental factors were screened through a variance inflation factor (VIF) analysis to retain those with low collinearity. The VIF is a measure of collinearity among predictor variables within a multiple regression. It is calculated by taking the variance ratio of all betas of a given model divided by the variance of a single beta. The environmental factors screened in this study were as follows: COD_Mn_, Cr^6+^, altitude, pH, TN, TP, and NH_4_^+^-N. A canonical correspondence analysis (CCA) was performed to determine the environmental variables associated with the changes in the bacterioplankton community structure using the vegan package. Linear discriminant analysis (LDA) effect size (LEfSe) is an algorithm for high-dimensional biomarker discovery and explanation, which identifies genomic features (genes, pathways, or taxa) characterizing the differences between two or more biological conditions [26]. In the present study, LEfSe was conducted to identify differential microbial functions between the main stream and tributary using an alpha parameter of 0.05 and an LDA threshold value of 3.5. Differences between two independent groups were evaluated by the Mann–Whitney *U* test. PICRUSt (phylogenetic investigation of communities by reconstruction of unobserved states) was used to predict the microbial functions and metabolic pathways [27]. Moreover, the functional profiles of the bacterial communities were predicted using PICRUSt2 from the Kyoto Encyclopedia of Genes and Genomes (KEGG) pathways.

## 3. Results

### 3.1. Environmental Characteristics

The environmental characteristics of the samples from the eight investigated sites are summarized in Table 1. The elevation of the eight sampling sites ranged from 1728 m to 3436 m, and the sites were generally considered as high plateau areas. The surface water temperature ranged from 6.8 °C to 17.2 °C, and the pH ranged from 8.34 to 8.47, indicating a weak alkaline. The Hg ranged from 0.03 mg/mL to 0.11 mg/mL, which was significantly lower in HS1 and HS2. A similar trend was found for As mg/mL (Mann–Whitney *U* test, *p* < 0.05). From the upper reaches to the lower reaches of the Datong River (HS7–HS3), the DO level showed a downward trend. On the contrary, the TN level showed an upward trend. The Cr^6+^, Cu, Zn, Pb, and Cd levels remained consistent in the eight investigated sites.

### 3.2. Diversity and Structure of the Bacterioplankton Communities

After quality filtering, a total of 497,604 valid reads (ranging from 56,234 to 69,445 per sample) were generated from eight sampling sites along the Huangshui River. All the samples were randomly resampled to 56,234 reads, and the reserved sequences were clustered into a total of 2384 OTUs. Good′s coverage (all samples, 99.80% ± 0.14%) suggested that the majority of the microbial species present in the samples were detected. The rarefaction curves tended to approach the saturation plateau, and the Shannon curves were stable (Figure S1), indicating a sufficient sampling depth of all the libraries. The microbial diversity index listed in Table 2 comprised community richness indices (Ace and Chao) and community diversity indices (Shannon and Simpson). The Shannon index of the water samples of HS4, HS6, and HS8, which indicated bacterial diversity, was significantly higher than that of the other sampling sites. The range of Chao was 58.33 to 1764.65, whereas the range of Ace was 111.46 to 1765.84, which were considered richness indices. HS6 had the highest level of the Chao and Ace indices, whereas HS5 had the lowest (Table 2). Except for HS5, HS1 and HS2 in the main stream had lower α diversities than the samples in the tributary.

**Figure 2:**
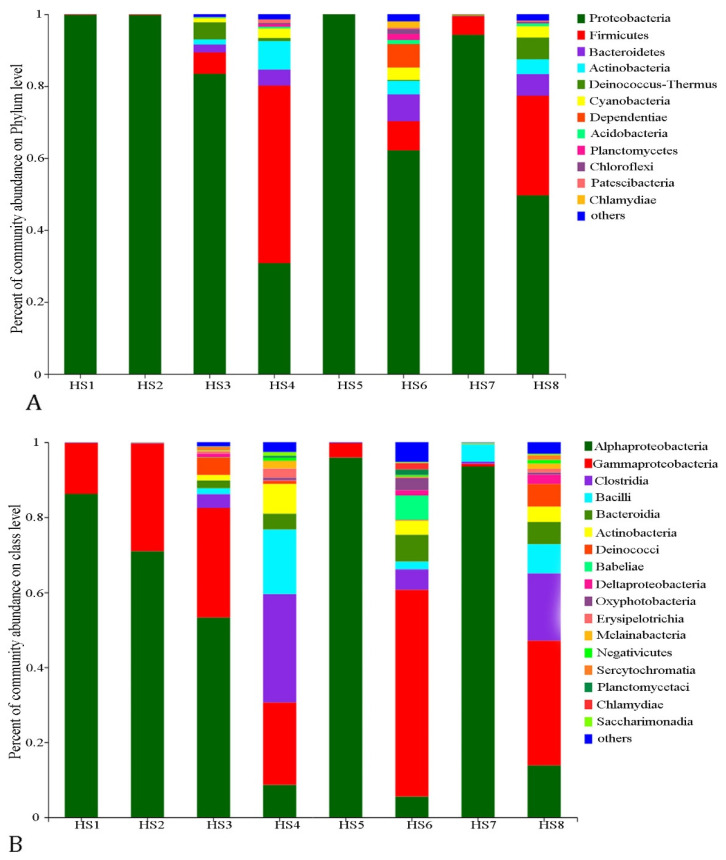
Bacterial composition of different sampling sites from the Huangshui River, the ecotone zone of Qinghai Plateau and Loess Plateau, China. (**A**) The relative abundance of the dominant bacterial phylum and (**B**) relative abundance of the dominant bacterial class are shown in a stack plot. The relative abundances are based on the ≥97% similarity clusters of the OTUs.

**Figure 3:**
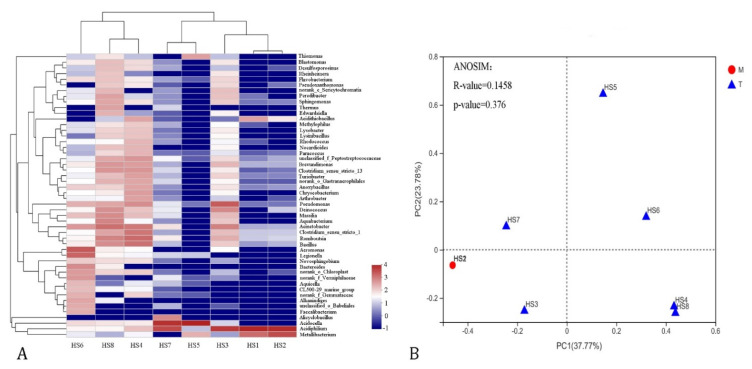
(**A**) A hierarchical clustering heatmap showing the main bacterial species in different groups at the genus level. Each rectangle is colored on the basis of a log_10_ value of the taxa percentage, as shown in the legend at the right of the plot. The top tree showed the clustering relationship of the samples. (**B**) A principal coordinates analysis of the main stream (red) and tributary stream (blue) of the Huangshui River based on the composition and abundance of the bacterial communities at the OUT level.

The OTUs were classified into 34 phyla, 78 classes, 216 orders, 386 families, and 821 genera (Table S1). Differences in the bacterial community composition were observed among different groups (Figure 2A,B and Figure 3A,B). Across all the sampling sites, the bacterioplankton communities were dominated by Proteobacteria and Firmicutes, and the relative abundance of these two phyla accounted for 77.48% and 12.08%, respectively. Notably, the relative abundance of Proteobacteria in HS1, HS2, HS5, and HS7 was above 99%, and it was as high as 99.98% at the HS5 sampling site. Interestingly, Proteobacteria dominated the sampling sites, but its classes were distributed differently. The abundance of Alphaproteobacteria was markedly different among the eight sampling sites, with a higher amount at the HS5 and HS7 sites (95.93% and 93.53%, respectively) and a lower amount at the HS4 and HS6 sites (8.68% and 5.52%, respectively). The Gammaproteobacteria was the most abundant class in HS6 (55.163%), whereas only 0.73% Gammaproteobacteria OTUs were detected in HS7 (Table S2). At the taxonomic level of genera, the bacterial communities of different sampling sits were different (Figure 3A). *Acidiphilum* (0.03–86.16%) and *Acidocella* (0–95.9%) were the most abundant genera, and they had a polar distribution within all the sampling sites. Remarkably, *Pseudomonas* was the dominant genus in HS3 (18.15%), and *Aeromonas* was dominant in HS6 (19.77%), whereas only a few *Pseudomonas* and *Aeromonas* OTUs were detected in the other sampling sites (Table S3).

Multivariate statistical analyses were conducted to compare the structure of the bacterial communities in different sampling sites. The principal coordinate analysis (PCoA) plot showed the first two PCs, which accounted for 37.77% and 23.78% of the total variation in the bacterioplankton communities, and the samples from the main stream were clustered (Figure 3B). ANOSIM revealed no significant differences (*p* = 0.376) in the bacterial community structures between the main stream and tributary stream.

### 3.3. Correlation Analysis of Bacterioplankton Communities and Environmental Factors

**Figure 4:**
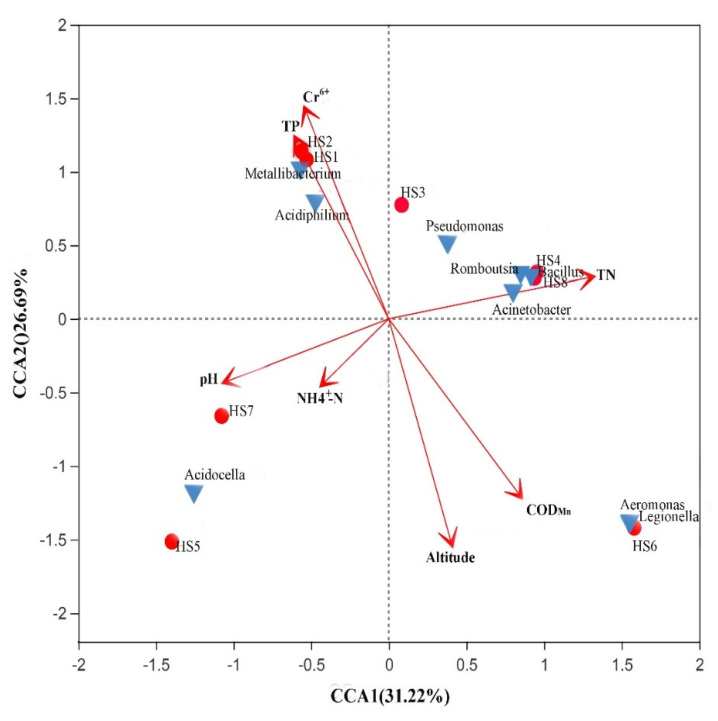
Canonical correspondence analysis (CCA) graph for the microorganism community and environmental factors. Red-filled circles indicate the water samples at the HS1–HS8 sites in the Huangshui River. Blue triangles indicate the top 8 genera among the eight sampling sites.

In this study, the water temperature; altitude; and 13 physicochemical parameters, including the pH and concentrations of DO, TN, TP, NH_4_^+^-N, Cr^6+^, COD_Mn_, Cu, Zn, Pb, Cd, Hg, and As, were considered to evaluate their relative contributions to the bacterial community and genera. A canonical correspondence analysis [27] was performed to determine the environmental variables associated with changes in the bacterioplankton community structure (Figure 4). The first axis accounted for 31.22% of the total variance, whereas the second axis accounted for 26.69%, indicating that the selected environmental factors led to differences in the bacterioplankton community structures. For correlations between environmental indicators and genera, the results showed that TP and Cr^6+^ contributed positively to *Acidiphilium* and *Metallibacterium* but contributed negatively to *Acidocella* and *Acinetobacter.* Furthermore, *Aeromonas* and *Legionella* seemed to be positively correlated with altitude and COD_Mn_.

### 3.4. Functional Prediction by PICRUSt2

**Figure 5:**
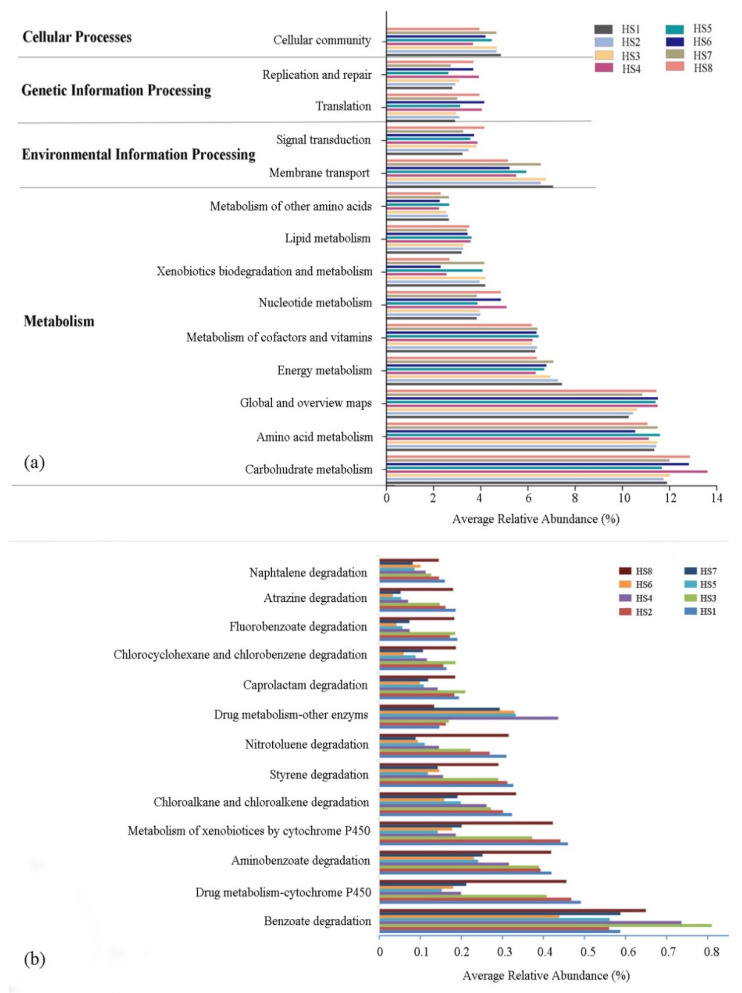
Functional level 1 categories (Kyoto Encyclopedia of Genes and Genomes (KEGG) (**a**)) of the bacterial communities and richness of the genes related to xenobiotic biodegradation and metabolism (**b**), as predicted by the Phylogenetic Investigation of Communities by Reconstruction of Unobserved States (PICRUSt2) analysis. Functional groups with less than 1% relative abundance are not included.

A community prediction analysis using PICRUSt2 was performed to determine the functions of the observed bacterioplankton. The results indicated that the main functional gene families were related to metabolism, environmental information processing, genetic information processing, and cellular processes (Figure 5a). Of all the different functional pathways identified, carbohydrate metabolism, amino acid metabolism, global and overview maps, energy metabolism, and membrane transport were substantially overrepresented compared with other pathways. However, no significant differences between the main stream and tributary microbial assemblages were found in the relative abundances of the three core metabolic pathways, including the carbohydrate metabolism, amino acid metabolism, and global and overview maps. In addition, rivers are the main recipients of pollutants and xenobiotics imported from river basins. Most aquatic organisms and bacteria are exposed to these xenobiotics [28]. Therefore, we focused on the functional genes of xenobiotic biodegradation and metabolism, and the 13 corresponding KEGG pathways were analyzed (Figure 5b). The average relative abundance of benzoate degradation was the highest, followed by drug metabolism–cytochrome P450 and aminobenzoate degradation. The main stream microbial assemblages had a high abundance of KEGG orthologs (Kos), which belonged to human diseases and cellular processes. Similarly, the tributary had a high abundance of KO, which belonged to the metabolism (Figure S2). In addition, several fairly abundant pathways were related to human diseases, including antibiotic resistance, cancers, and neurogenerative and infectious diseases. Among these diseases, antibiotic resistance was the most dominant (antimicrobial; Table S4).

## 4. Discussion

Bacteria play an important role in ensuring and maintaining the environment and ecological processes of river ecosystems, particularly in plateaus, which are a hotspot of microbial diversity. Comparatively few reports have focused on the bacterial diversity of the Qinghai–Tibet Plateau. Most studies have focused on bacterial communities in salts, gas fields, and polluted lakes [29,30]. Therefore, we aimed to study the Huangshui River to supplement the full-scale research of the river ecosystem on the plateau.

In our study, the Chao1 richness and Shannon index of the river samples were lower than those of plain rivers based on previous papers, which may be due to the special geographical environment. For instance, Fan et al. [31] reported that the Chao1 and Shannon indices of the planktonic bacterial community in the upper section of the tidal reach in the Yangtze River ranged from 764.33 to 861 and 4.36 to 4.72, respectively (Table 1). Hauptmann et al. [32] investigated the bacterioplankton community along a small Arctic River and revealed that the Chao1 indices ranged from 1408 to 19,117 OTUs, and the Shannon indices ranged from 5.6 to 10.8 per sample. Yang et al. [33] reported that the Chao1 and Shannon indices of bacteria in the Songhua River, one of the seven largest rivers in China, ranged from 1249.26 to 5780.18 and 2.85 to 5.21, respectively, which were higher than those of the Huangshui River (Table 2). Considering that the air is relatively thinner and the concentration of atmospheric suspended particle matter is lower in the plateau region, the solar UV radiation reaching the plateau surface is less attenuated. A high UV intensity can destroy the molecular structure of DNA or RNA in microbial organisms, resulting in low microbial diversities in plateau rivers [34]. However, the Chao1 and Shannon indices of the Heihe River, also known as the Plateau River, were similar to those of the Huangshui River, ranging from 300.546 to 2014.803 and 0.789 to 5.529, respectively [35]. Similar results were also found in the microbial abundance of stream biofilms, which was negatively correlated with altitude [36]. Given the extreme unique climate of the Qinghai–Tibet Plateau, including cold, hypoxia, and strong UV radiation, microorganisms may produce a series of community changes and genetic selection [37].

The decrease in alpha diversity at the input sites from the tributary of the Datong River (HS5) indicates that the tributary inputs less diverse bacterial communities into the main stream of the Datong River. The diversity of the two tributaries of the Datong River and Baoku River is significantly higher than that of the main stream of the Huangshui River, but the diversity of HS1 and HS2 has not increased because of the confluence of the two tributaries (Table 2). This result shows that the microbial community in the main stream of the Huangshui River does not change significantly because of the influx of tributaries. Moreover, the highly diverse communities from the Datong River and Baoku River are concealed by the low diversity of the main stream of the Huangshui River.

The bacterial community in the water samples was different with regards to the phylum, class, and genus (Figure 2A,B and Figure 3A,B). The high-throughput sequencing data showed that the dominant phylum was Proteobacteria, accounting for 99.98% of the sequences, followed by Firmicutes (49.38%). Due to extreme conditions, such as low temperature and oligotrophy, the rivers in the Qinghai–Tibet Plateau (also termed “the Third pole”) have similar environmental characteristics to those in the polar regions. However, the most abundant endemic taxa identified in the Huangshui River were different from those found in the polar regions (e.g., Bacteroidetes, Alphaproteobacteria, and Gammaproteobacteria in Antarctica; Betaproteobacteria, Bacteroidetes, and Cyanobacteria in the Arctic) [38]. This result may be due to the severe climates of these regions (the Qinghai Plateau and Loess Plateau, Antarctica, and the Arctic), and their geographic isolation has led to a high level of endemism. This result is similar to the results of the local and international studies of planktonic bacterial communities in rivers [39] and lakes [40,41]. The relative abundance of Proteobacteria is 77.48%, which is considered the most abundant phylum in wastewater [42,43]. However, notably, proteobacteria, which is the dominant phylum, consists of Alphaproteobacteria and Gammaproteobacteria, and the relative content of the two classes of bacteria is as high as 76.82%. Moreover, Alphaproteobacteria and Gammaproteobacteria can be found in freshwater and seawater, but they are more abundant in seawater [44,45,46]. This finding may be due to the salt mineral resources, such as the gypsum and calcium Glauber′s salt mine, which are widely distributed along the banks of the Huangshui River. Furthermore, the microbial composition of the Huangshui River is significantly different from other freshwater rivers at the class level.

At the genus level, the top six species in relative abundance at each sampling site were *Acidiphilium*, *Acidocella*, *Metallibacterium*, *Acinetobacter, Pseudomonas,* and *Aeromonas* (Table S3). Based on previous studies, *Acidiphilium*, *Acidocella*, and *Metallibacterium* dominate the microbial communities within mineral-leaching environments or acidic aquatic environments [47]. The pH of the Huangshui River was 8.34–8.47, indicating a weak alkaline, and the content of metal ions was low, which was not a suitable condition for the enrichment of these microorganisms (Table 1). Apart from its unique hydrology, biology, climate, and landform, the study area had notable geological and metallogenic conditions. Zeng [48] found that the Huangshui River had evident heavy metal pollution for the contents of Cd, Pb, Hg, and Cr in muddy sediments, which were all higher than the background values under natural conditions. We inferred that these acidophilic bacteria—namely, *Acidiphilium*, *Acidocella*, and *Metallibacterium*—in the upper reaches of the Huangshui River were derived from the sediments along the river, which were affected by strong winds and rainfall. Interestingly, HS4-7 is located in the same river system, but the bacterial communities at HS5 and -7 are very different from those at HS4 and HS6. According to our analysis, because there are mining areas near HS5 and HS7, acidophilic bacteria transferred to water bodies due to precipitation, wind, sand, etc., but the water environment was not suitable for its growth, so the levels of *Acidocella* near the mining site were high, while at the sampling sites far away from the mining site were low. In addition, we identified diverse taxa of potential pathogens, such as *Acinetobacter*, *Pseudomonas*, and *Aeromonas*. The abundance of *Acinetobacter* was primarily contributed by HS8 and HS4, whereas *Pseudomonas* and *Aeromonas* were primarily contributed by HS3 and HS6, respectively. *Acinetobacter* and *Pseudomonas* are major opportunistic waterborne pathogens causing hospital-acquired infections. These bacteria are opportunistic pathogens that do not affect healthy people. However, they can infect immunocompromised patients in hospitals (especially patients in intensive care units) with infections such as pneumonia, bacteremia, and urinary tract infections. Therefore, the presence of these bacteria in water may increase their colonization, with subsequent hospital-acquired infections [49]. The quality and quantity of rivers are influenced by human activities when they flow through different settlement types. Ibekwe et al. reported that urban areas, wastewater treatment work, and agricultural areas had significantly different effects on the microbial composition of the Santa Ana River in Southern California [50]. *Aeromonas*-associated cases of gastroenteritis are generally considered waterborne. *Aeromonas* spp. are primarily aquatic organisms that may be readily isolated from lakes, rivers, estuarine environments, sewage effluents, groundwater, drinking waters, and a wide range of raw foods [51]. The HS6 sampling site has a fish breeding station, which is an aquaculture area; thus, the enrichment of *Aeromonas* could be explained in this sampling site.

The bacterial community in river water can be affected by many factors, including the temperature, nutrient concentration, pH, and DO [52,53,54]. The physical and chemical indicators of the water bodies in the upper main stream and tributaries of the Heihe River vary at different sampling sites, and the water environment has a high spatial heterogeneity because of the variations in the environmental pressure carried by different river sections and the surrounding human activities [55]. In this study, the Cr^6+^, TP, altitude, and COD_Mn_ indicated the observed variability in the bacterioplankton community composition (Figure 4). The heavy metal ion Cr^6+^ has been proven to have an impact on the bacterial diversity [56]. COD_Mn_ is a comprehensive index to measure the degree of water pollution, which can greatly represent the organic matter components in water. The higher the COD_Mn_ is, the greater the degree of water pollution. Excessive phosphorus leads to eutrophication and deteriorates the water quality because of human activities and agriculture. Moreover, inorganic phosphorus is an important nitrogen source for heterotrophic bacteria. TP and Cr^6+^ increase the abundance of *Acidiphilium* and *Metallibacterium*, indicating that these two genera have good adaptability to changes in organic pollutions and heavy metal ions in rivers.

Understanding the functional characteristics of bacterial communities is of great significance to the study of ecosystem processes and community composition mechanisms. In the current study, the functional results showed that the overall functional profiles of the bacterial communities in the different samples were similar, and the pathways for carbohydrate metabolism and amino acid metabolism were enriched in all the samples. As the metabolic pathways of the core resources, the two pathways are the potential driving force for the structure and function of the microbial community in the river [28]. This was consistent with previous studies that the carbohydrate metabolism and amino acid metabolism of the bacterial communities might be highly correlated with environmental stress, such as a low temperature [57,58]. In addition, several pathways for xenobiotics biodegradation and metabolism were enriched in the bacterioplankton communities, suggesting that the bacterioplankton community plays an important role in self-purification and exogenous contamination remediation in water ecosystems [28]. Several studies have reported on environmental bacterial functions related to human diseases, as reviewed by Sandifer [59]. Antibiotic resistance has been shown to spread throughout the environment through mechanisms such as urban and agricultural runoffs, wind, and biological forces such as animals and humans [60]. The increasing number of reports on antimicrobial resistance is undeniably a troubling truth challenging humanity’s movement towards its global health agenda [61]. Given that rivers such as the Huangshui River are the main sources of water for drinking and personal and household hygiene in many developing countries, many people who use these polluted waters without prior treatment might be exposed to negative health effects because of the lack of adequate potable water supplies [56,62]. Our results suggested potential health risks of water sources in this area. Therefore, the effective governance of untreated sewage in rivers is of great importance.

## 5. Conclusions

The present study provided the first insights into the bacterial communities of the upper and middle reaches of the Huangshui River using high-throughput sequencing in the ecotone zone of the Qinghai Plateau and Loess Plateau. As a plateau river, the Huangshui River had a relatively low bacterial community diversity compared with plain rivers. Despite the similarities in the environmental conditions, the main taxa compositions of the bacterial communities were distinct between the Huangshui River and the polar regions (the Arctic and Antarctica). Proteobacteria was the dominant phyla, followed by Firmicutes. Given the salt mineral resources widely distributed along the banks of the Huangshui River, the Alphaproteobacteria and Gammaproteobacteria were significantly enriched. The predominant bacterial genera were *Acidiphilium*, *Acidocella*, *Metallibacterium*, and *Acinetobacter*. The diverse taxa of potential pathogens, such as *Acinetobacter*, *Pseudomonas*, and *Aeromonas*, were also identified. We also found site differences in the bacterial community structure in the river water. The water physical properties (e.g., Cr^6+^, TP, and COD_Mn_); altitude; and land use (e.g., urban land cover and aquaculture) could determine the distribution of the bacterioplankton composition. PICRUSt2 revealed that the overall functional profiles of the bacterial communities in the different samples were similar, and notably, our results suggested the potential health risks of water sources in this area. Furthermore, this work provided valuable insight into the composition of the plankton bacterial community and its relationship with the environmental factors in the Huangshui River in the ecotone zone of the Qinghai Plateau and Loess Plateau.

## Figures and Tables

**Table 1:** The water quality parameters of the different sampling sites.

Sample Site	HS1	HS2	HS3	HS4	HS5	HS6	HS7	HS8
T (°C)	13	14	13	17.20	16.00	9.30	6.80	9.30
pH	8.47	8.34	8.42	8.35	8.46	8.39	8.39	8.34
Altitude (m)	1728	1960	2630	2741	2838	3096	3436	2942
DO (mg/L)	/	/	6.20	7.80	6.30	6.90	7.30	7.10
TN (mg/L)	1.06	1.70	2.41	2.39	1.54	1.83	1.33	1.94
TP (mg/L)	0.01	0.02	0.01	0.01	0.01	0.01	0.01	0.01
NH_4_^+^-N (mg/L)	0.17	0.16	0.14	0.19	0.19	0.16	0.14	0.12
Cr^6+^ (mg/L)	0.01	0.01	0.01	0.01	0.01	0.01	0.01	0.01
COD_Mn_ (mg/L)	/	/	1.60	2.08	2.24	2.00	1.60	2.96
Cu (mg/mL)	0.50	0.50	0.50	0.50	0.50	0.50	0.50	0.50
Zn (mg/L)	0.03	0.03	0.03	0.03	0.03	0.03	0.03	0.03
Pb (mg/L)	0.01	0.01	0.01	0.01	0.01	0.01	0.01	0.01
Cd (mg/mL)	0.50	0.50	0.50	0.50	0.50	0.50	0.50	0.50
Hg (mg/mL)	0.03	0.03	0.11	0.11	0.11	0.11	0.11	0.11
As (mg/mL)	0.03	0.03	0.68	0.69	0.49	0.80	0.44	0.75

**Table 2:** Diversity index of microorganisms in each sampling sites.

Sampling Site	Sequence Number	Shannon	Simpson	Ace	Chao	Coverage
HS1	69,445	0.55	0.75	423.91	175.63	99.90%
HS2	56,403	0.69	0.58	184.05	104.15	99.93%
HS3	63,972	2.47	0.27	464.03	469.37	99.83%
HS4	56,234	4.34	0.03	382.38	383.50	99.96%
HS5	67,826	0.31	0.89	111.46	58.33	99.97%
HS6	59,560	5.05	0.06	1765.84	1764.35	99.67%
HS7	65,202	1.535	0.32	979.80	610.00	99.61%
HS8	58,962	4.97	0.02	640.05	641.02	99.91%

## Data Availability

The raw reads were deposited in the NCBI Sequence Read Archive database (Accession Number: SRP294338).

## Supplementary Materials

The following are available online at https://www.mdpi.com/article/10.3390/microorganisms9112260/s1. Figure S1: Rarefaction curves of all the sequences of different sampling sites of the Huangshui River, the ecotone zone of the Qinghai Plateau and Loess Plateau, China. Figure S2: A LDA analysis determined the differences in the bacterial functions between the main stream (M) and tributary (T). Table S1: OTU taxonomic statistics of the different sampling sites. Table S2: Composition of the top 6 microorganism groups in each sampling at the class level. Table S3: Composition of the top 6 microorganism groups in each sampling at the genus level. Table S4: Details of the KEGG database on Level 3.
