# Supplementary material for: Composition and Functional Characteristics and Influencing Factors of Bacterioplankton Community in the Huangshui River, China
Source: Microorganisms. 2021 Oct 29;9(11):2260. doi: 10.3390/microorganisms9112260 (PMC8623840; doi:10.3390/microorganisms9112260)
Supplement: Supplementary file 1 [file microorganisms-09-02260-s001.zip › Table S1.pdf]

Domain	Kingdom	Phylum	Class	Order	Family	Genus	Species	OTU
d__Bacteri	k__norank_	p__Proteob	c__Gamma	o__Betapro	f__Rhodocy	g__Zoogloe	s__uncultu	OTU2679
d__Bacteri	k__norank_	p__Gemma	c__Gemma	o__Gemma	f__Gemma	g__norank_	s__unclassi	OTU2672
d__Bacteri	k__norank_	p__Proteob	c__Gamma	o__Diploric	f__Diploric	g__Aquicel	s__uncultu	OTU2374
d__Bacteri	k__norank_	p__Chlamy	c__Chlamy	o__Chlamy	f__unclassi	g__unclass	s__unclassi	OTU2269
d__Bacteri	k__norank_	p__Actinob	c__Actinob	o__Propion	f__Nocardi	g__Nocardi	s__unclassi	OTU1392
d__Bacteri	k__norank_	p__Firmicu	c__Clostrid	o__Clostrid	f__Family_	g__unclass	s__unclassi	OTU1152
d__Bacteri	k__norank_	p__Firmicu	c__Clostrid	o__Clostrid	f__Clostridi	g__Clostrid	s__unclassi	OTU1348
d__Bacteri	k__norank_	p__Synergi	c__Synergis	o__Synergi	f__Synergis	g__Fretibac	s__uncultu	OTU1447
d__Bacteri	k__norank_	p__Depend	c__Babelia	o__Babelia	f__UBA124	g__norank_	s__uncultu	OTU1916
d__Bacteri	k__norank_	p__Proteob	c__Gamma	o__Diploric	f__Diploric	g__Aquicel	s__uncultu	OTU1955
d__Bacteri	k__norank_	p__Proteob	c__Gamma	o__unclass	f__unclassi	g__unclass	s__unclassi	OTU2224
d__Bacteri	k__norank_	p__Chlorof	c__Dehaloc	o__S085	f__norank_	g__norank_	s__uncultu	OTU3287
d__Bacteri	k__norank_	p__Fusoba	c__Fusobac	o__Fusoba	f__Fusobac	g__Fusoba	s__unclassi	OTU2497
d__Bacteri	k__norank_	p__Actinob	c__Actinob	o__Coryneb	f__Coryneb	g__Coryneb	s__Coryneb	OTU2767
d__Bacteri	k__norank_	p__Bacterc	c__Rhodoth	o__Rhodot	f__Rhodoth	g__Rubrivir	s__uncultu	OTU1336
d__Bacteri	k__norank_	p__Depend	c__Babelia	o__Babelia	f__Vermiph	g__norank_	s__uncultu	OTU2628
d__Bacteri	k__norank_	p__Acidoba	c__Acidoba	o__Solibac	f__Solibact	g__Candida	s__metage	OTU403
d__Bacteri	k__norank_	p__Firmicu	c__Bacilli	o__Bacillale	f__Paeniba	g__Ammor	s__uncultu	OTU1088
d__Bacteri	k__norank_	p__Proteob	c__Gamma	o__Xantho	f__Xanthor	g__Thermo	s__Thermo	OTU1403
d__Bacteri	k__norank_	p__Bacterc	c__Bactero	o__Bacterc	f__Bactero	g__Bactero	s__Bactero	OTU1134
d__Bacteri	k__norank_	p__Proteob	c__Gamma	o__Diploric	f__Diploric	g__Aquicel	s__uncultu	OTU1710
d__Bacteri	k__norank_	p__Actinob	c__Actinob	o__Nitrliru	f__Nitriliru	g__norank_	s__uncultu	OTU2226
d__Bacteri	k__norank_	p__Proteob	c__Gamma	o__Oceano	f__Halomo	g__Halomo	s__metage	OTU2614
d__Bacteri	k__norank_	p__Proteob	c__Gamma	o__Diploric	f__Diploric	g__Aquicel	s__uncultu	OTU1719
d__Bacteri	k__norank_	p__Bacterc	c__Bactero	o__Bacterc	f__Paludiba	g__F0058	s__uncultu	OTU810
d__Bacteri	k__norank_	p__Proteob	c__Deltapr	o__Sva048	f__norank_	g__norank_	s__uncultu	OTU1913
d__Bacteri	k__norank_	p__Firmicu	c__Clostrid	o__Clostrid	f__Peptocc	g__Desulfo	s__unclassi	OTU1267
d__Bacteri	k__norank_	p__Depend	c__Babelia	o__Babelia	f__Vermiph	g__norank_	s__uncultu	OTU2718
d__Bacteri	k__norank_	p__Depend	c__Babelia	o__Babelia	f__Vermiph	g__norank_	s__uncultu	OTU2714
d__Bacteri	k__norank_	p__Proteob	c__Gamma	o__Betapro	f__Burkhol	g__Parasut	s__unclassi	OTU1135
d__Bacteri	k__norank_	p__Firmicu	c__Clostrid	o__Clostrid	f__Helioba	g__Hydrog	s__unclassi	OTU1491
d__Bacteri	k__norank_	p__Deinoc	c__Deinocc	o__Deinoc	f__Deinocc	g__norank_	s__uncultu	OTU1381
d__Bacteri	k__norank_	p__Depend	c__Babelia	o__Babelia	f__Babelia	g__norank_	s__uncultu	OTU2784
d__Bacteri	k__norank_	p__Depend	c__Babelia	o__Babelia	f__unclassi	g__unclass	s__unclassi	OTU2472
d__Bacteri	k__norank_	p__Proteob	c__Gamma	o__Betapro	f__Burkhol	g__Pelomo	s__uncultu	OTU118
d__Bacteri	k__norank_	p__Bacterc	c__Bactero	o__Chitino	f__Chitinop	g__Flavisol	s__unclassi	OTU1176
d__Bacteri	k__norank_	p__Proteob	c__Gamma	o__Diploric	f__Diploric	g__norank_	s__uncultu	OTU1801
d__Bacteri	k__norank_	p__Proteob	c__Gamma	o__Betapro	f__Burkhol	g__unclass	s__unclassi	OTU744
d__Bacteri	k__norank_	p__Depend	c__Babelia	o__Babelia	f__unclassi	g__unclass	s__unclassi	OTU2236
d__Bacteri	k__norank_	p__Proteob	c__Alphapr	o__Rhizobi	f__Xanthob	g__Pseudo	s__uncultu	OTU1146
d__Bacteri	k__norank_	p__Patescil	c__WS6_D	o__norank_	f__norank_	g__norank_	s__candida	OTU2240
d__Bacteri	k__norank_	p__Proteob	c__Gamma	o__Legione	f__Legione	g__Legione	s__uncultu	OTU1765
d__Bacteri	k__norank_	p__Bacterc	c__Bactero	o__Flavoba	f__Crocinit	g__Fluviico	s__unclassi	OTU1469
d__Bacteri	k__norank_	p__Cyanob	c__Oxypho	o__Chlorop	f__norank_	g__norank_	s__unclassi	OTU1879
d__Bacteri	k__norank_	p__Proteob	c__Gamma	o__Diploric	f__Diploric	g__norank_	s__uncultu	OTU1215
d__Bacteri	k__norank_	p__Proteob	c__Gamma	o__Betapro	f__Burkhol	g__unclass	s__unclassi	OTU1507

d__Bacteri	k__norank_	p__Verrucc	c__Verrucc	o__Verrucc	f__Rubrital	g__Luteolit	s__uncultu	OTU1311
d__Bacteri	k__norank_	p__Firmicu	c__Clostrid	o__Clostrid	f__Clostridi	g__Oxobac	s__unclassi	OTU1495
d__Bacteri	k__norank_	p__Proteok	c__Gamma	o__Diploric	f__Diploric	g__Aquicel	s__unclassi	OTU2080
d__Bacteri	k__norank_	p__Proteok	c__Alphapr	o__Rhizobi	f__Rhizobia	g__Aureim	s__unclassi	OTU3615
d__Bacteri	k__norank_	p__Proteok	c__Alphapr	o__Holospo	f__Holospo	g__norank_	s__uncultu	OTU2038
d__Bacteri	k__norank_	p__Depenc	c__Babelia	o__Babelia	f__Vermipł	g__norank_	s__uncultu	OTU2848
d__Bacteri	k__norank_	p__Plancto	c__Phycisp	o__Phycisp	f__Phycispl	g__CL500-3	s__unclassi	OTU1920
d__Bacteri	k__norank_	p__Actinok	c__Actinob	o__Microc	f__Microcc	g__Arthrob	s__Arthrob	OTU1566
d__Bacteri	k__norank_	p__Actinok	c__Actinob	o__Strepto	f__Strepto	g__Nonom	s__unclassi	OTU4134
d__Bacteri	k__norank_	p__Bacterc	c__Bactero	o__Flavoba	f__Flavoba	g__Flavoba	s__unclassi	OTU1138
d__Bacteri	k__norank_	p__Proteok	c__Gamma	o__Diploric	f__Diploric	g__norank_	s__unclassi	OTU1871
d__Bacteri	k__norank_	p__Firmicu	c__Clostrid	o__Clostrid	f__Rumino	g__unclass	s__unclassi	OTU1642
d__Bacteri	k__norank_	p__Proteok	c__Gamma	o__Diploric	f__Diploric	g__Aquicel	s__unclassi	OTU2352
d__Bacteri	k__norank_	p__Proteok	c__Deltapr	o__Desulfo	f__Desulfo	g__Desulfo	s__Desulfo	OTU2764
d__Bacteri	k__norank_	p__Patescil	c__Parcuba	o__Candida	f__norank_	g__norank_	s__unclassi	OTU504
d__Bacteri	k__norank_	p__Proteok	c__Gamma	o__Betaprc	f__Methylc	g__unclass	s__unclassi	OTU3017
d__Bacteri	k__norank_	p__Actinok	c__Actinob	o__PeM15	f__norank_	g__norank_	s__unclassi	OTU4065
d__Bacteri	k__norank_	p__Actinok	c__Actinob	o__Microc	f__Microba	g__Candida	s__unclassi	OTU3552
d__Bacteri	k__norank_	p__Acidoba	c__Subgrou	o__norank_	f__norank_	g__norank_	s__unclassi	OTU3760
d__Bacteri	k__norank_	p__Proteok	c__Gamma	o__unclass	f__unclassi	g__unclass	s__unclassi	OTU2583
d__Bacteri	k__norank_	p__Acidoba	c__Blastoc	o__Blastoc	f__Blastoca	g__Blastoc	s__uncultu	OTU1363
d__Bacteri	k__norank_	p__Proteok	c__Gamma	o__Betaprc	f__Rhodoc	g__Sulfurit	s__uncultu	OTU37
d__Bacteri	k__norank_	p__Cyanob	c__Oxypho	o__Nostoca	f__Phormic	g__Tychon	s__Tychon	OTU2623
d__Bacteri	k__norank_	p__Proteok	c__Gamma	o__Immun	f__Immunc	g__Immun	s__unclassi	OTU925
d__Bacteri	k__norank_	p__Actinok	c__Actinob	o__Microtr	f__Iamiace	g__Iamia	s__uncultu	OTU1983
d__Bacteri	k__norank_	p__Proteok	c__Alphapr	o__Rhodos	f__norank_	g__norank_	s__unclassi	OTU3882
d__Bacteri	k__norank_	p__Cyanob	c__Oxypho	o__Chlorop	f__norank_	g__norank_	s__unclassi	OTU1644
d__Bacteri	k__norank_	p__Proteok	c__Alphapr	o__Rhizobi	f__Xanthok	g__norank_	s__unclassi	OTU185
d__Bacteri	k__norank_	p__Cyanob	c__Melaina	o__Gastrar	f__norank_	g__norank_	s__uncultu	OTU1488
d__Bacteri	k__norank_	p__Proteok	c__Gamma	o__Betaprc	f__TRA3-20	g__norank_	s__unclassi	OTU3515
d__Bacteri	k__norank_	p__Cyanob	c__Melaina	o__Gastrar	f__norank_	g__norank_	s__Candida	OTU1553
d__Bacteri	k__norank_	p__Bacterc	c__Bactero	o__Bacterc	f__Muribac	g__unclass	s__unclassi	OTU2824
d__Bacteri	k__norank_	p__Actinok	c__Actinob	o__Gaiellal	f__norank_	g__norank_	s__uncultu	OTU2658
d__Bacteri	k__norank_	p__Patescil	c__Microge	o__Candida	f__norank_	g__norank_	s__Candida	OTU1815
d__Bacteri	k__norank_	p__Proteok	c__Gamma	o__Betaprc	f__Rhodoc	g__Zoogloe	s__metage	OTU1274
d__Bacteri	k__norank_	p__Cyanob	c__Melaina	o__Gastrar	f__norank_	g__norank_	s__uncultu	OTU1494
d__Bacteri	k__norank_	p__Proteok	c__Deltapr	o__Desulfu	f__Geobaci	g__Geobac	s__unclassi	OTU2179
d__Bacteri	k__norank_	p__Proteok	c__Alphapr	o__Rhizobi	f__Rhizobia	g__Phreatc	s__unclassi	OTU2174
d__Bacteri	k__norank_	p__Proteok	c__Gamma	o__Betaprc	f__Burkhol	g__Noviher	s__unclassi	OTU107
d__Bacteri	k__norank_	p__Actinok	c__Actinob	o__Microc	f__Intraspo	g__unclass	s__unclassi	OTU1543
d__Bacteri	k__norank_	p__Proteok	c__Alphapr	o__Acetoba	f__Acetoba	g__Acidoce	s__uncultu	OTU996
d__Bacteri	k__norank_	p__Depenc	c__Babelia	o__Babelia	f__Vermipł	g__norank_	s__metage	OTU2778
d__Bacteri	k__norank_	p__Chlamy	c__Chlamy	o__Chlamy	f__Simkani	g__norank_	s__unclassi	OTU1616
d__Bacteri	k__norank_	p__Proteok	c__Gamma	o__Betaprc	f__Neisseri	g__norank_	s__metage	OTU2119
d__Bacteri	k__norank_	p__Verrucc	c__Verrucc	o__Verrucc	f__Rubrital	g__Luteolit	s__unclassi	OTU1692
d__Bacteri	k__norank_	p__Firmicu	c__Clostrid	o__Clostrid	f__Family_	g__Tissiere	s__unclassi	OTU2252
d__Bacteri	k__norank_	p__Firmicu	c__Clostrid	o__Clostrid	f__Rumino	g__Ruminc	s__uncultu	OTU1087

d__Bacteri	k__norank_	p__Proteol	c__Gamma	o__Xantho	f__Rhodan	g__Metallil	s__uncultu	OTU1303
d__Bacteri	k__norank_	p__Proteol	c__Gamma	o__Legione	f__Legione	g__Legione	s__uncultu	OTU2086
d__Bacteri	k__norank_	p__Firmicu	c__Bacilli	o__Bacillal	f__Planoco	g__unclass	s__unclassi	OTU1587
d__Bacteri	k__norank_	p__Proteol	c__Gamma	o__Diploric	f__Diploric	g__Aquicel	s__unclassi	OTU1842
d__Bacteri	k__norank_	p__Bacterc	c__Ignavib	o__OPB56	f__norank_	g__norank_	s__uncultu	OTU1307
d__Bacteri	k__norank_	p__Patescil	c__Sacchar	o__Sacchar	f__unclassi	g__unclass	s__unclassi	OTU1433
d__Bacteri	k__norank_	p__Bacterc	c__Bactero	o__Bacterc	f__Rikenell	g__Rikenel	s__unclassi	OTU1133
d__Bacteri	k__norank_	p__Patescil	c__Sacchar	o__Sacchar	f__unclassi	g__unclass	s__unclassi	OTU1438
d__Bacteri	k__norank_	p__Bacterc	c__Bactero	o__Bacterc	f__Muribac	g__norank_	s__uncultu	OTU2993
d__Bacteri	k__norank_	p__Caldise	c__Caldiser	o__Caldise	f__Caldiser	g__Caldiser	s__unclassi	OTU2406
d__Bacteri	k__norank_	p__Chlamy	c__Chlamy	o__Chlamy	f__cvE6	g__norank_	s__uncultu	OTU2541
d__Bacteri	k__norank_	p__Chlamy	c__Chlamy	o__Chlamy	f__cvE6	g__norank_	s__unclassi	OTU2359
d__Bacteri	k__norank_	p__Firmicu	c__Clostrid	o__Clostrid	f__Christer	g__Christer	s__unclassi	OTU1185
d__Bacteri	k__norank_	p__Proteol	c__Alphapr	o__Acetob	f__Acetoba	g__Acidiph	s__unclassi	OTU1299
d__Bacteri	k__norank_	p__Proteol	c__Alphapr	o__Acetob	f__Acetoba	g__Acidiph	s__unclassi	OTU1297
d__Bacteri	k__norank_	p__Proteol	c__Gamma	o__Betaprc	f__TRA3-2(	g__norank_	s__unclassi	OTU3225
d__Bacteri	k__norank_	p__Bacterc	c__Bactero	o__Sphingc	f__env.OPS	g__norank_	s__uncultu	OTU1549
d__Bacteri	k__norank_	p__Proteol	c__Gamma	o__Diploric	f__Diploric	g__Aquicel	s__unclassi	OTU2836
d__Bacteri	k__norank_	p__Firmicu	c__Clostrid	o__Clostrid	f__Rumino	g__Ruminc	s__unclassi	OTU1057
d__Bacteri	k__norank_	p__Proteol	c__Gamma	o__Diploric	f__Diploric	g__Aquicel	s__uncultu	OTU1004
d__Bacteri	k__norank_	p__Actinob	c__Actinob	o__Frankia	f__Sporicht	g__hgcl_cla	s__unclassi	OTU3577
d__Bacteri	k__norank_	p__Depenc	c__Babelia	o__Babelia	f__Vermipl	g__norank_	s__uncultu	OTU2159
d__Bacteri	k__norank_	p__Firmicu	c__Clostrid	o__Clostrid	f__Lachnos	g__Lachnos	s__uncultu	OTU2009
d__Bacteri	k__norank_	p__Chlamy	c__Chlamy	o__Chlamy	f__unclassi	g__unclass	s__unclassi	OTU2781
d__Bacteri	k__norank_	p__Proteol	c__Gamma	o__Betaprc	f__Nitrosor	g__GOUTA	s__metage	OTU2165
d__Bacteri	k__norank_	p__Proteol	c__Gamma	o__Diploric	f__Diploric	g__Aquicel	s__unclassi	OTU1191
d__Bacteri	k__norank_	p__Firmicu	c__Negativ	o__Selenor	f__Acidami	g__Succinic	s__uncultu	OTU2103
d__Bacteri	k__norank_	p__Depenc	c__Babelia	o__Babelia	f__unclassi	g__unclass	s__unclassi	OTU1850
d__Bacteri	k__norank_	p__Firmicu	c__Bacilli	o__Bacillal	f__Alicyclol	g__Tumeba	s__Tumeba	OTU1249
d__Bacteri	k__norank_	p__Bacterc	c__Bactero	o__Chitino	f__Chitinop	g__Dinghui	s__metage	OTU2275
d__Bacteri	k__norank_	p__Proteol	c__Gamma	o__Betaprc	f__Burkhol	g__unclass	s__unclassi	OTU2744
d__Bacteri	k__norank_	p__Chlorof	c__OLB14	o__norank_	f__norank_	g__norank_	s__unclassi	OTU223
d__Bacteri	k__norank_	p__Bacterc	c__Bactero	o__Flavoba	f__NS9_ma	g__norank_	s__unident	OTU2839
d__Bacteri	k__norank_	p__Acidoba	c__Subgrou	o__unclass	f__unclassi	g__Luteital	s__uncultu	OTU1241
d__Bacteri	k__norank_	p__Firmicu	c__Clostrid	o__Clostrid	f__Lachnos	g__norank_	s__Clostrid	OTU1047
d__Bacteri	k__norank_	p__Bacterc	c__Ignavib	o__OPB56	f__norank_	g__norank_	s__uncultu	OTU159
d__Bacteri	k__norank_	p__Proteol	c__Gamma	o__Legione	f__Legione	g__Legione	s__uncultu	OTU541
d__Bacteri	k__norank_	p__Proteol	c__Gamma	o__Diploric	f__Diploric	g__Aquicel	s__uncultu	OTU2626
d__Bacteri	k__norank_	p__Firmicu	c__Clostrid	o__Clostrid	f__Gracilib	g__Gracilib	s__uncultu	OTU1094
d__Bacteri	k__norank_	p__Chlamy	c__Chlamy	o__Chlamy	f__Parachla	g__unclass	s__unclassi	OTU2062
d__Bacteri	k__norank_	p__Proteol	c__Alphapr	o__Acetob	f__Acetoba	g__norank_	s__uncultu	OTU4124
d__Bacteri	k__norank_	p__Proteol	c__Gamma	o__Betaprc	f__Burkhol	g__Burkhol	s__Parabur	OTU2874
d__Bacteri	k__norank_	p__Firmicu	c__Bacilli	o__Lactoba	f__Enteroc	g__Enteroc	s__Enteroc	OTU560
d__Bacteri	k__norank_	p__Verrucc	c__Verrucc	o__Verrucc	f__Verruco	g__Prosthe	s__uncultu	OTU907
d__Bacteri	k__norank_	p__Depenc	c__Babelia	o__Babelia	f__unclassi	g__unclass	s__unclassi	OTU2437
d__Bacteri	k__norank_	p__Proteol	c__Gamma	o__Compe	f__Compet	g__Candida	s__wastew	OTU1284
d__Bacteri	k__norank_	p__Proteol	c__Alphapr	o__Paracae	f__Paracae	g__Candida	s__uncultu	OTU1038

d__Bacteri	k__norank_	p__Actinob	c__Actinob	o__Microc	f__Microba	g__Agromy	s__Agromy	OTU1182
d__Bacteri	k__norank_	p__Firmicu	c__Bacilli	o__Bacillal	f__Planoco	g__Solibaci	s__Solibaci	OTU540
d__Bacteri	k__norank_	p__Firmicu	c__Clostrid	o__Clostrid	f__Helioba	g__Hydrog	s__unclassi	OTU1109
d__Bacteri	k__norank_	p__Firmicu	c__Clostrid	o__Clostrid	f__Helioba	g__Hydrog	s__unclassi	OTU1108
d__Bacteri	k__norank_	p__Firmicu	c__Negativ	o__Selenor	f__Veillone	g__Megam	s__unclassi	OTU1223
d__Bacteri	k__norank_	p__Firmicu	c__Clostrid	o__Clostrid	f__Christer	g__norank_	s__uncultu	OTU1157
d__Bacteri	k__norank_	p__Acidoba	c__Thermo	o__Thermo	f__Thermo	g__Subgrou	s__uncultu	OTU1694
d__Bacteri	k__norank_	p__Chlamy	c__Chlamy	o__Chlamy	f__Parachla	g__Neochla	s__unclassi	OTU1810
d__Bacteri	k__norank_	p__Proteob	c__Gamma	o__Betapro	f__Burkhol	g__Limnoh	s__uncultu	OTU2370
d__Bacteri	k__norank_	p__Depend	c__Babelia	o__Babelia	f__Vermiph	g__norank_	s__uncultu	OTU2490
d__Bacteri	k__norank_	p__Chlamy	c__Chlamy	o__Chlamy	f__Parachla	g__unclass	s__unclassi	OTU2407
d__Bacteri	k__norank_	p__Depend	c__Babelia	o__Babelia	f__norank_	g__norank_	s__uncultu	OTU2663
d__Bacteri	k__norank_	p__Proteob	c__Gamma	o__Legione	f__Legione	g__Legione	s__unclassi	OTU2553
d__Bacteri	k__norank_	p__Proteob	c__Gamma	o__Legione	f__Legione	g__Legione	s__unclassi	OTU2557
d__Bacteri	k__norank_	p__Proteob	c__Alphapr	o__Rhizobi	f__Xanthob	g__Pseudo	s__metage	OTU3992
d__Bacteri	k__norank_	p__Proteob	c__Gamma	o__Gamma	f__unclassi	g__Candida	s__uncultu	OTU2648
d__Bacteri	k__norank_	p__Gemma	c__Gemma	o__Gemma	f__Gemma	g__norank_	s__uncultu	OTU1148
d__Bacteri	k__norank_	p__Proteob	c__Deltapr	o__Desulfo	f__Desulfo	g__Desulfo	s__unclassi	OTU1767
d__Bacteri	k__norank_	p__Actinob	c__Actinob	o__Microc	f__Cellulon	g__Cellulor	s__uncultu	OTU1540
d__Bacteri	k__norank_	p__Proteob	c__Deltapr	o__NB1-j	f__norank_	g__norank_	s__unclassi	OTU2602
d__Bacteri	k__norank_	p__Depend	c__Babelia	o__Babelia	f__Vermiph	g__norank_	s__unclassi	OTU2766
d__Bacteri	k__norank_	p__Bacterc	c__Bactero	o__Bacterc	f__Prevote	g__Prevote	s__uncultu	OTU70
d__Bacteri	k__norank_	p__Firmicu	c__Clostrid	o__Clostrid	f__Lachnos	g__norank_	s__unclassi	OTU1030
d__Bacteri	k__norank_	p__Proteob	c__Gamma	o__Betapro	f__Burkhol	g__Comam	s__uncultu	OTU2443
d__Bacteri	k__norank_	p__Verruco	c__Verruco	o__Verruco	f__Rubrital	g__Luteolib	s__uncultu	OTU1231
d__Bacteri	k__norank_	p__Acidoba	c__Subgrou	o__norank_	f__norank_	g__norank_	s__unclassi	OTU1822
d__Bacteri	k__norank_	p__Depend	c__Babelia	o__Babelia	f__Vermiph	g__norank_	s__uncultu	OTU1978
d__Bacteri	k__norank_	p__Proteob	c__Gamma	o__unclass	f__unclassi	g__unclass	s__unclassi	OTU2456
d__Bacteri	k__norank_	p__Acidoba	c__Subgrou	o__norank_	f__norank_	g__norank_	s__unclassi	OTU3779
d__Bacteri	k__norank_	p__Proteob	c__Gamma	o__Diploric	f__Diploric	g__norank_	s__unclassi	OTU2111
d__Bacteri	k__norank_	p__Firmicu	c__unclassi	o__unclass	f__unclassi	g__unclass	s__unclassi	OTU2571
d__Bacteri	k__norank_	p__Bacterc	c__Bactero	o__Bacterc	f__Prevote	g__Prevote	s__unclassi	OTU2908
d__Bacteri	k__norank_	p__Firmicu	c__Clostrid	o__Clostrid	f__Rumino	g__Rumino	s__unclassi	OTU1541
d__Bacteri	k__norank_	p__Actinob	c__Actinob	o__Microc	f__Microcc	g__Nestere	s__uncultu	OTU635
d__Bacteri	k__norank_	p__Firmicu	c__Bacilli	o__Bacillal	f__Bacillac	g__Anaerol	s__unclassi	OTU1420
d__Bacteri	k__norank_	p__Depend	c__Babelia	o__Babelia	f__unclassi	g__unclass	s__unclassi	OTU1625
d__Bacteri	k__norank_	p__Firmicu	c__Bacilli	o__Bacillal	f__Paeniba	g__Paeniba	s__Paeniba	OTU1482
d__Bacteri	k__norank_	p__Proteob	c__Alphapr	o__Acetob	f__Acetoba	g__Acidoce	s__unclassi	OTU1580
d__Bacteri	k__norank_	p__Proteob	c__Alphapr	o__Rickett	f__Midichl	g__norank_	s__uncultu	OTU1973
d__Bacteri	k__norank_	p__Bacterc	c__Bactero	o__Bacterc	f__Barnesi	g__Barnesi	s__Barnesi	OTU1721
d__Bacteri	k__norank_	p__Cyanob	c__Sericytc	o__norank_	f__norank_	g__norank_	s__uncultu	OTU1372
d__Bacteri	k__norank_	p__Proteob	c__Gamma	o__Gamma	f__unclassi	g__norank_	s__uncultu	OTU1787
d__Bacteri	k__norank_	p__Proteob	c__Alphapr	o__unclass	f__unclassi	g__unclass	s__unclassi	OTU2016
d__Bacteri	k__norank_	p__Proteob	c__Alphapr	o__Caulob	f__Cauloba	g__unclass	s__unclassi	OTU297
d__Bacteri	k__norank_	p__Firmicu	c__Bacilli	o__Bacillal	f__Thermo	g__Risungb	s__unclassi	OTU1167
d__Bacteri	k__norank_	p__Chlamy	c__Chlamy	o__Chlamy	f__Parachla	g__Neochla	s__unclassi	OTU2551
d__Bacteri	k__norank_	p__Firmicu	c__Bacilli	o__Bacillal	f__Bacillac	g__Sinibaci	s__uncultu	OTU1253

d__Bacteri	k__norank_	p__Firmicu	c__Clostrid	o__Clostrid	f__Rumino	g__Candida	s__unclassi	OTU1559
d__Bacteri	k__norank_	p__Depenc	c__Babelia	o__Babelia	f__unclassi	g__unclass	s__unclassi	OTU87
d__Bacteri	k__norank_	p__Plancto	c__Plancto	o__Isospha	f__Isospha	g__Paludis	s__uncultu	OTU1314
d__Bacteri	k__norank_	p__Proteol	c__Gamma	o__Diploric	f__Diploric	g__norank_	s__unclassi	OTU2609
d__Bacteri	k__norank_	p__Depenc	c__Babelia	o__Babelia	f__Vermipl	g__norank_	s__uncultu	OTU2388
d__Bacteri	k__norank_	p__Depenc	c__Babelia	o__Babelia	f__Vermipl	g__norank_	s__uncultu	OTU2386
d__Bacteri	k__norank_	p__Proteol	c__Gamma	o__Alteron	f__Shewan	g__Psychrc	s__uncultu	OTU2558
d__Bacteri	k__norank_	p__Depenc	c__Babelia	o__Babelia	f__Vermipl	g__norank_	s__uncultu	OTU2383
d__Bacteri	k__norank_	p__Proteol	c__Gamma	o__Pseudo	f__Moraxe	g__Perlucic	s__uncultu	OTU876
d__Bacteri	k__norank_	p__Firmicu	c__Clostrid	o__Clostrid	f__Lachnos	g__Ruminc	s__unclassi	OTU2251
d__Bacteri	k__norank_	p__Firmicu	c__Clostrid	o__Clostrid	f__Peptocc	g__Desulfo	s__uncultu	OTU1234
d__Bacteri	k__norank_	p__Depenc	c__Babelia	o__Babelia	f__Vermipl	g__norank_	s__uncultu	OTU1718
d__Bacteri	k__norank_	p__Firmicu	c__Clostrid	o__Clostrid	f__Rumino	g__Flavonif	s__unclassi	OTU1906
d__Bacteri	k__norank_	p__Fibroba	c__Fibroba	o__Fibroba	f__B5-096	g__norank_	s__uncultu	OTU1942
d__Bacteri	k__norank_	p__Proteol	c__Gamma	o__Acidithi	f__Acidithi	g__Acidithi	s__Acidithi	OTU562
d__Bacteri	k__norank_	p__Acidoba	c__Subgrou	o__norank_	f__norank_	g__norank_	s__unclassi	OTU3308
d__Bacteri	k__norank_	p__Proteol	c__Gamma	o__Betaprc	f__Methylc	g__Methyl	s__uncultu	OTU491
d__Bacteri	k__norank_	p__Verrucc	c__Verrucc	o__Verrucc	f__Rubrital	g__Luteolil	s__uncultu	OTU3676
d__Bacteri	k__norank_	p__Deinoc	c__Deinocc	o__Deinoc	f__Deinocc	g__Deinoc	s__unclassi	OTU1330
d__Bacteri	k__norank_	p__Proteol	c__Gamma	o__Betaprc	f__Nitrosor	g__MND1	s__unclassi	OTU3146
d__Bacteri	k__norank_	p__Proteol	c__Alphapr	o__Rhizobi	f__Devosia	g__Pelagib	s__uncultu	OTU1359
d__Bacteri	k__norank_	p__Firmicu	c__Bacilli	o__Bacillal	f__Paeniba	g__Paeniba	s__uncultu	OTU644
d__Bacteri	k__norank_	p__Firmicu	c__Clostrid	o__Clostrid	f__Christer	g__Christer	s__uncultu	OTU1189
d__Bacteri	k__norank_	p__Bacterc	c__Bactero	o__Flavoba	f__Crocinit	g__Fluviico	s__unclassi	OTU1724
d__Bacteri	k__norank_	p__Proteol	c__Gamma	o__Pseudo	f__Pseudor	g__Pseudo	s__unclassi	OTU1023
d__Bacteri	k__norank_	p__Firmicu	c__Clostrid	o__Clostrid	f__Rumino	g__Ruminic	s__uncultu	OTU1070
d__Bacteri	k__norank_	p__Firmicu	c__Bacilli	o__Lactoba	f__Strepto	g__Strepto	s__Strepto	OTU2448
d__Bacteri	k__norank_	p__Proteol	c__Gamma	o__Legione	f__Legione	g__Legione	s__unclassi	OTU1599
d__Bacteri	k__norank_	p__Chlorof	c__OLB14	o__norank_	f__norank_	g__norank_	s__unclassi	OTU1969
d__Bacteri	k__norank_	p__Actinol	c__Actinob	o__Propior	f__Propion	g__Cutibac	s__Cutibac	OTU1153
d__Bacteri	k__norank_	p__Proteol	c__Deltapr	o__Oligofle	f__0319-6C	g__norank_	s__unclassi	OTU3296
d__Bacteri	k__norank_	p__Hydrog	c__Hydrog	o__Hydrog	f__Hydroge	g__norank_	s__uncultu	OTU2391
d__Bacteri	k__norank_	p__Rokuba	c__NC10	o__Rokuba	f__norank_	g__norank_	s__uncultu	OTU1150
d__Bacteri	k__norank_	p__Firmicu	c__Bacilli	o__Bacillal	f__Pasteuri	g__Pasteur	s__unclassi	OTU2831
d__Bacteri	k__norank_	p__Proteol	c__Gamma	o__Legione	f__Legione	g__Legione	s__uncultu	OTU1775
d__Bacteri	k__norank_	p__Proteol	c__Gamma	o__Betaprc	f__Hydroge	g__Thiobac	s__unclassi	OTU1532
d__Bacteri	k__norank_	p__Chlamy	c__Chlamy	o__Chlamy	f__unclassi	g__unclass	s__unclassi	OTU1777
d__Bacteri	k__norank_	p__Proteol	c__Gamma	o__unclass	f__unclassi	g__unclass	s__unclassi	OTU2415
d__Bacteri	k__norank_	p__Acidoba	c__Blastoc	o__Blastoc	f__Blastoca	g__JGI_00C	s__uncultu	OTU852
d__Bacteri	k__norank_	p__Actinol	c__Actinob	o__Actinor	f__Actinom	g__Actinon	s__unclassi	OTU769
d__Bacteri	k__norank_	p__Proteol	c__Alphapr	o__Acetob	f__Acetoba	g__unclass	s__unclassi	OTU2924
d__Bacteri	k__norank_	p__Depenc	c__Babelia	o__Babelia	f__Vermipl	g__norank_	s__unclassi	OTU3787
d__Bacteri	k__norank_	p__Proteol	c__Gamma	o__Coxiella	f__Coxiella	g__Coxiella	s__uncultu	OTU1621
d__Bacteri	k__norank_	p__Proteol	c__Gamma	o__Methyl	f__Methylc	g__Crenoth	s__uncultu	OTU1889
d__Bacteri	k__norank_	p__Chlamy	c__Chlamy	o__Chlamy	f__Simkani	g__norank_	s__unclassi	OTU2489
d__Bacteri	k__norank_	p__Firmicu	c__Bacilli	o__Bacillal	f__Thermo	g__Thermc	s__unclassi	OTU1426
d__Bacteri	k__norank_	p__unclass	c__unclassi	o__unclass	f__unclassi	g__unclass	s__unclassi	OTU1471

d__Bacteri	k__norank_	p__Patescil	c__Sacchar	o__Sacchar	f__norank_	g__norank_	s__unclassi	OTU2019
d__Bacteri	k__norank_	p__Firmicu	c__Bacilli	o__Bacillal	f__Paeniba	g__Paeniba	s__Paeniba	OTU1009
d__Bacteri	k__norank_	p__Chlamy	c__Chlamy	o__Chlamy	f__Parachla	g__Neochla	s__unclassi	OTU1826
d__Bacteri	k__norank_	p__Bacterc	c__Bactero	o__Cytoph	f__Spirosor	g__Flectob	s__Flectob	OTU1126
d__Bacteri	k__norank_	p__Firmicu	c__Bacilli	o__Lactoba	f__Carnoba	g__Trichoc	s__unclassi	OTU2647
d__Bacteri	k__norank_	p__Verrucc	c__Verrucc	o__Chthon	f__Terrimic	g__FukuN1	s__uncultu	OTU1671
d__Bacteri	k__norank_	p__Actinob	c__Actinob	o__Microtr	f__Ilumato	g__CL500-2	s__uncultu	OTU882
d__Bacteri	k__norank_	p__Firmicu	c__Bacilli	o__Bacillal	f__Bacillac	g__Marino	s__Marino	OTU1317
d__Bacteri	k__norank_	p__Proteob	c__Gamma	o__Gamma	f__unclassi	g__Candida	s__uncultu	OTU2436
d__Bacteri	k__norank_	p__Proteob	c__Alphapr	o__Rhizobi	f__Beijerin	g__Bosea	s__uncultu	OTU1306
d__Bacteri	k__norank_	p__Depend	c__Babelia	o__Babelia	f__norank_	g__norank_	s__uncultu	OTU2616
d__Bacteri	k__norank_	p__Proteob	c__Gamma	o__Cardiob	f__Wohlfat	g__Ignatzs	s__uncultu	OTU2527
d__Bacteri	k__norank_	p__Proteob	c__Gamma	o__Legione	f__Legione	g__Legione	s__unclassi	OTU2011
d__Bacteri	k__norank_	p__Chlamy	c__Chlamy	o__Chlamy	f__cvE6	g__norank_	s__uncultu	OTU2393
d__Bacteri	k__norank_	p__Proteob	c__Gamma	o__Legione	f__Legione	g__Legione	s__uncultu	OTU1932
d__Bacteri	k__norank_	p__Proteob	c__Gamma	o__Betaprc	f__T34	g__norank_	s__uncultu	OTU1958
d__Bacteri	k__norank_	p__Chlorof	c__Anaerol	o__Anaero	f__Anaerol	g__norank_	s__unclassi	OTU2175
d__Bacteri	k__norank_	p__Proteob	c__Deltapr	o__Desulfc	f__Desulfol	g__Sva008	s__uncultu	OTU2120
d__Bacteri	k__norank_	p__Depend	c__Babelia	o__Babelia	f__Vermipł	g__norank_	s__uncultu	OTU1852
d__Bacteri	k__norank_	p__Proteob	c__Gamma	o__Legione	f__Legione	g__Legione	s__unclassi	OTU2304
d__Bacteri	k__norank_	p__Firmicu	c__Clostrid	o__Clostrid	f__Clostridi	g__Clostrid	s__Clostrid	OTU2055
d__Bacteri	k__norank_	p__Proteob	c__Alphapr	o__Rhodob	f__Rhodob	g__Rubellir	s__unclassi	OTU1291
d__Bacteri	k__norank_	p__Acidoba	c__Blastoc	o__Pyrinon	f__Pyrinom	g__RB41	s__uncultu	OTU3404
d__Bacteri	k__norank_	p__Chlorof	c__Anaerol	o__SBR103	f__A4b	g__norank_	s__wastew	OTU1068
d__Bacteri	k__norank_	p__Bacterc	c__Bactero	o__Chitino	f__Chitinop	g__Niastell	s__uncultu	OTU1631
d__Bacteri	k__norank_	p__Proteob	c__Gamma	o__Betaprc	f__Methylc	g__Candida	s__unclassi	OTU936
d__Bacteri	k__norank_	p__Firmicu	c__Clostrid	o__Clostrid	f__Eubacte	g__Alkaliba	s__uncultu	OTU1060
d__Bacteri	k__norank_	p__Proteob	c__Gamma	o__Diploric	f__Diploric	g__norank_	s__unclassi	OTU2298
d__Bacteri	k__norank_	p__Bacterc	c__Bactero	o__Flavoba	f__Flavoba	g__Flavoba	s__unclassi	OTU2638
d__Bacteri	k__norank_	p__Actinob	c__Actinob	o__Frankia	f__Nakamu	g__Nakamu	s__uncultu	OTU1001
d__Bacteri	k__norank_	p__Depend	c__Babelia	o__Babelia	f__unclassi	g__unclass	s__unclassi	OTU2747
d__Bacteri	k__norank_	p__Chlamy	c__Chlamy	o__Chlamy	f__Simkani	g__norank_	s__unclassi	OTU2069
d__Bacteri	k__norank_	p__Bacterc	c__Bactero	o__Bacterc	f__Prevote	g__Prevote	s__Prevote	OTU1073
d__Bacteri	k__norank_	p__Proteob	c__Gamma	o__Pseudo	f__Moraxe	g__Psychrc	s__Psychro	OTU2630
d__Bacteri	k__norank_	p__Firmicu	c__Bacilli	o__Bacillal	f__Paeniba	g__Paeniba	s__Paeniba	OTU1039
d__Bacteri	k__norank_	p__Firmicu	c__Bacilli	o__Lactoba	f__Strepto	g__Lactoco	s__unclassi	OTU2433
d__Bacteri	k__norank_	p__Bacterc	c__Bactero	o__Cytoph	f__Spirosor	g__Rhabdo	s__unclassi	OTU1136
d__Bacteri	k__norank_	p__Proteob	c__Gamma	o__Betaprc	f__Burkhol	g__Limnob	s__uncultu	OTU915
d__Bacteri	k__norank_	p__Depend	c__Babelia	o__Babelia	f__Vermipł	g__norank_	s__uncultu	OTU2769
d__Bacteri	k__norank_	p__Proteob	c__Alphapr	o__Acetob	f__Acetoba	g__Roseom	s__unclassi	OTU1354
d__Bacteri	k__norank_	p__Plancto	c__Plancto	o__Gemma	f__Gemma	g__norank_	s__unclassi	OTU988
d__Bacteri	k__norank_	p__Depend	c__Babelia	o__Babelia	f__Vermipł	g__norank_	s__uncultu	OTU2763
d__Bacteri	k__norank_	p__Proteob	c__Alphapr	o__NRL2	f__norank_	g__norank_	s__uncultu	OTU18
d__Bacteri	k__norank_	p__Depend	c__Babelia	o__Babelia	f__Babelia	g__norank_	s__uncultu	OTU2215
d__Bacteri	k__norank_	p__Proteob	c__Gamma	o__Betaprc	f__Burkhol	g__MWH-U	s__beta_pr	OTU2537
d__Bacteri	k__norank_	p__Proteob	c__Gamma	o__Betaprc	f__Methylc	g__Methyl	s__unclassi	OTU3804
d__Bacteri	k__norank_	p__Proteob	c__Gamma	o__Diploric	f__Diploric	g__norank_	s__metage	OTU2473

d__Bacteri	k__norank_	p__Proteo	c__Gamma	o__unclass	f__unclassi	g__unclass	s__unclassi	OTU2521
d__Bacteri	k__norank_	p__Depen	c__Babelia	o__Babelia	f__Babelia	g__norank_	s__uncultu	OTU2750
d__Bacteri	k__norank_	p__Depen	c__Babelia	o__Babelia	f__unclassi	g__unclass	s__unclassi	OTU1997
d__Bacteri	k__norank_	p__Proteo	c__Gamma	o__Betapr	f__TRA3-2	g__norank_	s__unclassi	OTU442
d__Bacteri	k__norank_	p__Proteo	c__Gamma	o__unclass	f__unclassi	g__unclass	s__unclassi	OTU2200
d__Bacteri	k__norank_	p__Firmicu	c__Clostrid	o__Clostri	f__Rumino	g__unclass	s__unclassi	OTU1762
d__Bacteri	k__norank_	p__Verruc	c__Verruc	o__Pedosp	f__Pedosp	g__norank_	s__uncultu	OTU3696
d__Bacteri	k__norank_	p__Depen	c__Babelia	o__Babelia	f__Vermip	g__norank_	s__uncultu	OTU2366
d__Bacteri	k__norank_	p__Depen	c__Babelia	o__Babelia	f__Vermip	g__norank_	s__uncultu	OTU2363
d__Bacteri	k__norank_	p__Depen	c__Babelia	o__Babelia	f__Vermip	g__norank_	s__uncultu	OTU2362
d__Bacteri	k__norank_	p__Proteo	c__Gamma	o__Coxiell	f__Coxiella	g__Coxiella	s__uncultu	OTU2345
d__Bacteri	k__norank_	p__Firmicu	c__Clostrid	o__Clostri	f__Lachno	g__unclass	s__unclassi	OTU1963
d__Bacteri	k__norank_	p__Firmicu	c__Clostrid	o__Clostri	f__Helioba	g__norank_	s__uncultu	OTU1195
d__Bacteri	k__norank_	p__Gemma	c__Gemma	o__Gemma	f__Gemma	g__Gemma	s__unclassi	OTU3351
d__Bacteri	k__norank_	p__Proteo	c__Gamma	o__Xantho	f__Xantho	g__Pseudo	s__Pseudo	OTU551
d__Bacteri	k__norank_	p__Firmicu	c__Bacilli	o__Bacilla	f__Thermo	g__Risung	s__unclassi	OTU1588
d__Bacteri	k__norank_	p__Proteo	c__Gamma	o__Nitroso	f__Nitroso	g__Cl75cm	s__uncultu	OTU2133
d__Bacteri	k__norank_	p__Proteo	c__Alphap	o__Sphing	f__Sphingo	g__Sphing	s__unclassi	OTU701
d__Bacteri	k__norank_	p__Depen	c__Babelia	o__Babelia	f__UBA124	g__norank_	s__metage	OTU2617
d__Bacteri	k__norank_	p__Chlamy	c__Chlamy	o__Chlamy	f__Simkani	g__norank_	s__unclassi	OTU1776
d__Bacteri	k__norank_	p__Proteo	c__Gamma	o__Betapr	f__Nitroso	g__Ellin60	s__uncultu	OTU2201
d__Bacteri	k__norank_	p__Chlorof	c__Chlorof	o__Thermo	f__JG30-K	g__norank_	s__uncultu	OTU1535
d__Bacteri	k__norank_	p__Proteo	c__Alphap	o__Acetob	f__Acetoba	g__unclass	s__unclassi	OTU422
d__Bacteri	k__norank_	p__Bacter	c__Bactero	o__Sphing	f__env.OP	g__norank_	s__uncultu	OTU2572
d__Bacteri	k__norank_	p__Bacter	c__Bactero	o__Chitino	f__norank_	g__norank_	s__unclassi	OTU1346
d__Bacteri	k__norank_	p__Bacter	c__Bactero	o__Flavoba	f__Flavoba	g__norank_	s__unclassi	OTU2247
d__Bacteri	k__norank_	p__Cyanob	c__Melain	o__Obscur	f__norank_	g__norank_	s__uncultu	OTU2815
d__Bacteri	k__norank_	p__Firmicu	c__Bacilli	o__Bacilla	f__Paeniba	g__Paeniba	s__uncultu	OTU1335
d__Bacteri	k__norank_	p__Proteo	c__Deltapr	o__Desulfu	f__Geobac	g__Geobac	s__uncultu	OTU2380
d__Bacteri	k__norank_	p__Depen	c__Babelia	o__Babelia	f__unclassi	g__unclass	s__unclassi	OTU2250
d__Bacteri	k__norank_	p__Depen	c__Babelia	o__Babelia	f__unclassi	g__unclass	s__unclassi	OTU2390
d__Bacteri	k__norank_	p__Proteo	c__Gamma	o__Methyl	f__Methyl	g__unclass	s__unclassi	OTU1927
d__Bacteri	k__norank_	p__unclass	c__unclassi	o__unclass	f__unclassi	g__unclass	s__unclassi	OTU2327
d__Bacteri	k__norank_	p__Proteo	c__Deltapr	o__Bdellov	f__Bdellovi	g__Bdellov	s__Bdellovi	OTU127
d__Bacteri	k__norank_	p__Proteo	c__Gamma	o__Xantho	f__Xantho	g__unclass	s__unclassi	OTU1006
d__Bacteri	k__norank_	p__Firmicu	c__Negativ	o__Seleno	f__Veillone	g__Anaero	s__uncultu	OTU1238
d__Bacteri	k__norank_	p__Actino	c__Actinob	o__Microc	f__Dermac	g__Barrien	s__unclassi	OTU1166
d__Bacteri	k__norank_	p__Proteo	c__Gamma	o__Legion	f__Legione	g__Legion	s__unclassi	OTU1780
d__Bacteri	k__norank_	p__Proteo	c__Gamma	o__Gamma	f__unclassi	g__Candid	s__unclassi	OTU2598
d__Bacteri	k__norank_	p__Depen	c__Babelia	o__Babelia	f__Vermip	g__norank_	s__unclassi	OTU1840
d__Bacteri	k__norank_	p__Chlorof	c__Anaero	o__Anaero	f__Anaero	g__norank_	s__uncultu	OTU2841
d__Bacteri	k__norank_	p__Proteo	c__Deltapr	o__Oligofl	f__0319-6	g__norank_	s__bacteri	OTU1807
d__Bacteri	k__norank_	p__Depen	c__Babelia	o__Babelia	f__Vermip	g__norank_	s__unclassi	OTU2774
d__Bacteri	k__norank_	p__Proteo	c__Alphap	o__Caulob	f__Cauloba	g__Asticca	s__Asticca	OTU376
d__Bacteri	k__norank_	p__Bacter	c__Bactero	o__Cytoph	f__Spiroso	g__norank_	s__metage	OTU265
d__Bacteri	k__norank_	p__Bacter	c__Bactero	o__Flavoba	f__Flavoba	g__Flavoba	s__unclassi	OTU938
d__Bacteri	k__norank_	p__Proteo	c__Alphap	o__Sphing	f__Sphingo	g__Sphing	s__unclassi	OTU1010

d__Bacteri	k__norank_	p__Firmicu	c__Clostrid	o__Clostrid	f__Clostridi	g__Oxobac	s__uncultu	OTU1643
d__Bacteri	k__norank_	p__Proteol	c__Gamma	o__Ga0077	f__norank_	g__norank_	s__unclassi	OTU2786
d__Bacteri	k__norank_	p__Proteol	c__Gamma	o__Enterol	f__Enterob	g__Serratia	s__Serratia	OTU2503
d__Bacteri	k__norank_	p__Bacterc	c__Bactero	o__Flavoba	f__Flavoba	g__Flavoba	s__unclassi	OTU1397
d__Bacteri	k__norank_	p__Firmicu	c__Bacilli	o__Bacillal	f__Alicyclol	g__Tumeba	s__uncultu	OTU1430
d__Bacteri	k__norank_	p__Firmicu	c__Bacilli	o__Lactoba	f__Lactoba	g__Lactoba	s__unclassi	OTU2515
d__Bacteri	k__norank_	p__Proteol	c__Gamma	o__Methyl	f__Methylc	g__unclass	s__unclassi	OTU2338
d__Bacteri	k__norank_	p__Verrucc	c__Verrucc	o__Opituta	f__Puniceic	g__Verruc-	s__Opituta	OTU1652
d__Bacteri	k__norank_	p__Firmicu	c__Erysipel	o__Erysipe	f__Erysipel	g__ZOR000	s__Cetobac	OTU1165
d__Bacteri	k__norank_	p__Verrucc	c__Verrucc	o__Verrucc	f__Verruco	g__Prosthe	s__uncultu	OTU1281
d__Bacteri	k__norank_	p__Bacterc	c__Bactero	o__Flavoba	f__Flavoba	g__Capnoc	s__Capnoc	OTU1325
d__Bacteri	k__norank_	p__Chlamy	c__Chlamy	o__Chlamy	f__Criblam	g__norank_	s__unclassi	OTU2573
d__Bacteri	k__norank_	p__Chlorof	c__Anaerol	o__SBR103	f__norank_	g__norank_	s__uncultu	OTU1614
d__Bacteri	k__norank_	p__Chlamy	c__Chlamy	o__Chlamy	f__Simkani	g__norank_	s__unclassi	OTU2248
d__Bacteri	k__norank_	p__Proteol	c__Deltapr	o__MBNT1	f__norank_	g__norank_	s__uncultu	OTU1837
d__Bacteri	k__norank_	p__Proteol	c__Gamma	o__Betapro	f__T34	g__norank_	s__uncultu	OTU2703
d__Bacteri	k__norank_	p__Proteol	c__Gamma	o__Diploric	f__Diploric	g__unclass	s__unclassi	OTU2154
d__Bacteri	k__norank_	p__Acidoba	c__Acidoba	o__Solibac	f__Solibact	g__Bryobac	s__uncultu	OTU1098
d__Bacteri	k__norank_	p__Firmicu	c__Bacilli	o__Bacillal	f__Alicyclol	g__Acidiba	s__Acidibac	OTU564
d__Bacteri	k__norank_	p__Verrucc	c__Verrucc	o__Pedosp	f__Pedospl	g__norank_	s__unclassi	OTU2077
d__Bacteri	k__norank_	p__Bacterc	c__Bactero	o__Flavoba	f__Flavoba	g__Capnoc	s__Capnoc	OTU797
d__Bacteri	k__norank_	p__Proteol	c__Alphapr	o__Reyran	f__Reyrane	g__norank_	s__unclassi	OTU3838
d__Bacteri	k__norank_	p__Bacterc	c__Bactero	o__Bacterc	f__Bactero	g__norank_	s__uncultu	OTU3679
d__Bacteri	k__norank_	p__Chlamy	c__Chlamy	o__Chlamy	f__Parachla	g__unclass	s__unclassi	OTU2346
d__Bacteri	k__norank_	p__unclass	c__unclassi	o__unclass	f__unclassi	g__unclass	s__unclassi	OTU1897
d__Bacteri	k__norank_	p__Proteol	c__Gamma	o__Betapro	f__SC-I-84	g__norank_	s__unclassi	OTU1846
d__Bacteri	k__norank_	p__Actinob	c__Actinob	o__Gaiellal	f__norank_	g__norank_	s__metage	OTU2332
d__Bacteri	k__norank_	p__Proteol	c__Deltapr	o__Bdellov	f__Bacteric	g__Peredib	s__unclassi	OTU1256
d__Bacteri	k__norank_	p__Actinob	c__Actinob	o__Microc	f__Microba	g__Microba	s__Microba	OTU173
d__Bacteri	k__norank_	p__Firmicu	c__Negativ	o__Selenor	f__Veillone	g__unclass	s__unclassi	OTU1119
d__Bacteri	k__norank_	p__Bacterc	c__Bactero	o__Cytoph	f__Cytopha	g__Cytopha	s__Cytopha	OTU1290
d__Bacteri	k__norank_	p__Depenc	c__Babelia	o__Babelia	f__Vermipl	g__norank_	s__unclassi	OTU2411
d__Bacteri	k__norank_	p__Firmicu	c__Clostrid	o__Clostrid	f__Rumino	g__unclass	s__unclassi	OTU805
d__Bacteri	k__norank_	p__Proteol	c__Gamma	o__Betapro	f__Chromo	g__Vogesel	s__uncultu	OTU1163
d__Bacteri	k__norank_	p__Firmicu	c__Clostrid	o__Clostrid	f__Rumino	g__Ruminc	s__metage	OTU1930
d__Bacteri	k__norank_	p__Patescil	c__Gracilib	o__norank_	f__norank_	g__norank_	s__unclassi	OTU1782
d__Bacteri	k__norank_	p__Depenc	c__Babelia	o__Babelia	f__Vermipl	g__norank_	s__unclassi	OTU2808
d__Bacteri	k__norank_	p__Proteol	c__Gamma	o__Legione	f__Legione	g__Legione	s__uncultu	OTU2545
d__Bacteri	k__norank_	p__Verrucc	c__Verrucc	o__Verrucc	f__Rubrital	g__Luteolil	s__uncultu	OTU3782
d__Bacteri	k__norank_	p__Depenc	c__Babelia	o__Babelia	f__norank_	g__norank_	s__uncultu	OTU2463
d__Bacteri	k__norank_	p__Proteol	c__Deltapr	o__Myxocc	f__BIrii41	g__norank_	s__uncultu	OTU1922
d__Bacteri	k__norank_	p__Proteol	c__Gamma	o__Diploric	f__Diploric	g__norank_	s__unclassi	OTU1740
d__Bacteri	k__norank_	p__Firmicu	c__Clostrid	o__Clostrid	f__Rumino	g__Ruminic	s__unclassi	OTU1512
d__Bacteri	k__norank_	p__Bacterc	c__Bactero	o__Flavoba	f__Weekse	g__Chrysec	s__Candida	OTU1002
d__Bacteri	k__norank_	p__Proteol	c__Gamma	o__Legione	f__Legione	g__Legione	s__unclassi	OTU1768
d__Bacteri	k__norank_	p__Firmicu	c__Negativ	o__Selenor	f__Veillone	g__Megam	s__unclassi	OTU2409
d__Bacteri	k__norank_	p__Proteol	c__Gamma	o__Betapro	f__Burkhol	g__unclass	s__unclassi	OTU2554

d__Bacteri	k__norank_	p__Proteob	c__Gamma	o__Betapro	f__B1-7BS	g__norank_	s__unclassi	OTU2258
d__Bacteri	k__norank_	p__Fusoba	c__Fusobac	o__Fusoba	f__Leptotri	g__Leptotr	s__unclassi	OTU1258
d__Bacteri	k__norank_	p__Firmicu	c__Clostrid	o__Clostrid	f__Lachnos	g__Lachnos	s__Clostrid	OTU4126
d__Bacteri	k__norank_	p__Proteob	c__Gamma	o__Betapro	f__Neisseri	g__norank_	s__metage	OTU1803
d__Bacteri	k__norank_	p__Proteob	c__Gamma	o__Betapro	f__Methylo	g__OM43_	s__uncultu	OTU2560
d__Bacteri	k__norank_	p__Firmicu	c__Bacilli	o__Bacillal	f__Thermo	g__Shimazu	s__uncultu	OTU1184
d__Bacteri	k__norank_	p__Proteob	c__Alphapr	o__Ricketts	f__Ricketts	g__norank_	s__metage	OTU2007
d__Bacteri	k__norank_	p__unclass	c__unclassi	o__unclass	f__unclassi	g__unclass	s__unclassi	OTU1266
d__Bacteri	k__norank_	p__Actinob	c__Actinob	o__Actinor	f__Actinom	g__F0332	s__uncultu	OTU1028
d__Bacteri	k__norank_	p__Verruco	c__Verruco	o__Pedosp	f__Pedosph	g__norank_	s__bacteriu	OTU1866
d__Bacteri	k__norank_	p__Cyanob	c__Sericyto	o__norank_	f__norank_	g__norank_	s__uncultu	OTU2044
d__Bacteri	k__norank_	p__Proteob	c__Gamma	o__Diploric	f__Diploric	g__Aquicel	s__uncultu	OTU1596
d__Bacteri	k__norank_	p__Proteob	c__Deltapr	o__Myxoco	f__P3OB-4	g__norank_	s__metage	OTU825
d__Bacteri	k__norank_	p__Proteob	c__Alphapr	o__unclass	f__unclassi	g__unclass	s__unclassi	OTU1592
d__Bacteri	k__norank_	p__unclass	c__unclassi	o__unclass	f__unclassi	g__unclass	s__unclassi	OTU1669
d__Bacteri	k__norank_	p__Firmicu	c__Clostrid	o__Clostrid	f__Rumino	g__unclass	s__unclassi	OTU1194
d__Bacteri	k__norank_	p__Chlamy	c__Chlamy	o__Chlamy	f__Parachla	g__unclass	s__unclassi	OTU2641
d__Bacteri	k__norank_	p__Proteob	c__Gamma	o__Xantho	f__Rhodan	g__Metallib	s__unclassi	OTU1294
d__Bacteri	k__norank_	p__Proteob	c__Gamma	o__Diploric	f__Diploric	g__Aquicel	s__uncultu	OTU2643
d__Bacteri	k__norank_	p__Chlamy	c__Chlamy	o__Chlamy	f__Simkani	g__norank_	s__unclassi	OTU1748
d__Bacteri	k__norank_	p__Firmicu	c__Clostrid	o__Clostrid	f__Clostridi	g__Clostrid	s__unclassi	OTU1557
d__Bacteri	k__norank_	p__Depend	c__Babelia	o__Babelia	f__Babeliac	g__norank_	s__unclassi	OTU2353
d__Bacteri	k__norank_	p__Fusoba	c__Fusobac	o__Fusoba	f__Leptotri	g__Hypnoc	s__unclassi	OTU4131
d__Bacteri	k__norank_	p__Firmicu	c__Clostrid	o__Clostrid	f__Christer	g__Christer	s__uncultu	OTU1421
d__Bacteri	k__norank_	p__Armatir	c__Fimbriir	o__Fimbriir	f__Fimbriir	g__norank_	s__metage	OTU1391
d__Bacteri	k__norank_	p__Plancto	c__BD7-11	o__norank_	f__norank_	g__norank_	s__uncultu	OTU1441
d__Bacteri	k__norank_	p__Epsilon	c__Campyl	o__Campyl	f__Arcobac	g__Arcobac	s__uncultu	OTU1618
d__Bacteri	k__norank_	p__Firmicu	c__Bacilli	o__Bacillal	f__Paeniba	g__Paeniba	s__Paeniba	OTU1040
d__Bacteri	k__norank_	p__Cyanob	c__Oxypho	o__Chlorop	f__norank_	g__norank_	s__Nephro	OTU1584
d__Bacteri	k__norank_	p__Proteob	c__Gamma	o__Diploric	f__Diploric	g__Aquicel	s__uncultu	OTU1833
d__Bacteri	k__norank_	p__Proteob	c__Gamma	o__Cardiob	f__Cardiob	g__Cardiob	s__uncultu	OTU675
d__Bacteri	k__norank_	p__Nitrosp	c__Thermo	o__norank_	f__norank_	g__norank_	s__uncultu	OTU2981
d__Bacteri	k__norank_	p__Firmicu	c__Clostrid	o__Clostrid	f__Rumino	g__Rumino	s__unclassi	OTU1276
d__Bacteri	k__norank_	p__Proteob	c__Alphapr	o__Caulob	f__Hyphom	g__SWB02	s__unclassi	OTU11
d__Bacteri	k__norank_	p__Proteob	c__Gamma	o__Betapro	f__Nitrosor	g__unclass	s__unclassi	OTU603
d__Bacteri	k__norank_	p__Proteob	c__Gamma	o__Salinisp	f__Solimon	g__norank_	s__uncultu	OTU79
d__Bacteri	k__norank_	p__Firmicu	c__Bacilli	o__Bacillal	f__Paeniba	g__Paeniba	s__Paeniba	OTU660
d__Bacteri	k__norank_	p__unclass	c__unclassi	o__unclass	f__unclassi	g__unclass	s__unclassi	OTU1319
d__Bacteri	k__norank_	p__Proteob	c__Gamma	o__Pseudo	f__Moraxe	g__Acineto	s__unclassi	OTU2633
d__Bacteri	k__norank_	p__Bactero	c__Bactero	o__Chitino	f__Chitinop	g__unclass	s__unclassi	OTU1338
d__Bacteri	k__norank_	p__Proteob	c__Gamma	o__Diploric	f__Diploric	g__Aquicel	s__uncultu	OTU1935
d__Bacteri	k__norank_	p__Bactero	c__Bactero	o__Bactero	f__Muribac	g__norank_	s__unclassi	OTU2832
d__Bacteri	k__norank_	p__Depend	c__Babelia	o__Babelia	f__Vermiph	g__norank_	s__uncultu	OTU2559
d__Bacteri	k__norank_	p__Depend	c__Babelia	o__Babelia	f__Vermiph	g__norank_	s__uncultu	OTU2196
d__Bacteri	k__norank_	p__Proteob	c__Gamma	o__Betapro	f__Burkhol	g__Ralston	s__Ralstoni	OTU63
d__Bacteri	k__norank_	p__Firmicu	c__Clostrid	o__Clostrid	f__Clostridi	g__Fontice	s__uncultu	OTU1099
d__Bacteri	k__norank_	p__Depend	c__Babelia	o__Babelia	f__Vermiph	g__norank_	s__uncultu	OTU2447

d__Bacteri	k__norank_	p__Proteob	c__Gamma	o__unclass	f__unclassi	g__unclass	s__unclassi	OTU2730
d__Bacteri	k__norank_	p__Proteob	c__Gamma	o__Betapro	f__Burkhol	g__Thiomo	s__Thiomo	OTU1579
d__Bacteri	k__norank_	p__Proteob	c__Gamma	o__Betapro	f__Burkhol	g__Polynuc	s__metage	OTU4069
d__Bacteri	k__norank_	p__Chlorof	c__KD4-96	o__norank_	f__norank_	g__norank_	s__uncultu	OTU300
d__Bacteri	k__norank_	p__Firmicu	c__Clostrid	o__Clostrid	f__Clostridi	g__Clostrid	s__unclassi	OTU1021
d__Bacteri	k__norank_	p__Acidoba	c__Subgrou	o__norank_	f__norank_	g__norank_	s__unclassi	OTU503
d__Bacteri	k__norank_	p__Depend	c__Babelia	o__Babelia	f__UBA124	g__norank_	s__metage	OTU2535
d__Bacteri	k__norank_	p__Firmicu	c__Clostrid	o__Clostrid	f__Rumino	g__Rumino	s__unclassi	OTU868
d__Bacteri	k__norank_	p__Proteob	c__Gamma	o__Pseudo	f__Moraxe	g__Perlucic	s__Perlucic	OTU3610
d__Bacteri	k__norank_	p__Depend	c__Babelia	o__Babelia	f__Vermiph	g__norank_	s__uncultu	OTU2287
d__Bacteri	k__norank_	p__unclass	c__unclassi	o__unclass	f__unclassi	g__unclass	s__unclassi	OTU2210
d__Bacteri	k__norank_	p__Bacterc	c__Bactero	o__Flavoba	f__Weekse	g__Chrysec	s__unclassi	OTU1476
d__Bacteri	k__norank_	p__Cyanob	c__Oxypho	o__Chlorop	f__norank_	g__norank_	s__unclassi	OTU2762
d__Bacteri	k__norank_	p__Firmicu	c__Bacilli	o__Bacillal	f__Planoco	g__Domiba	s__unclassi	OTU1521
d__Bacteri	k__norank_	p__Firmicu	c__Clostrid	o__Clostrid	f__Syntrop	g__Syntrop	s__uncultu	OTU1251
d__Bacteri	k__norank_	p__Depend	c__Babelia	o__Babelia	f__Vermiph	g__norank_	s__uncultu	OTU2084
d__Bacteri	k__norank_	p__Proteob	c__Gamma	o__Diploric	f__Diploric	g__norank_	s__unclassi	OTU2100
d__Bacteri	k__norank_	p__Proteob	c__Gamma	o__Pseudo	f__Pseudor	g__Pseudo	s__Pseudo	OTU1246
d__Bacteri	k__norank_	p__Plancto	c__Plancto	o__Isospha	f__Isospha	g__Paludis	s__uncultu	OTU1271
d__Bacteri	k__norank_	p__Chlamy	c__Chlamy	o__Chlamy	f__Parachla	g__norank_	s__metage	OTU1623
d__Bacteri	k__norank_	p__Epsilon	c__Campyl	o__Campyl	f__Arcobac	g__Arcoba	s__unclassi	OTU4112
d__Bacteri	k__norank_	p__Bacterc	c__Bactero	o__Cytoph	f__Hymenc	g__Hymen	s__Hymen	OTU149
d__Bacteri	k__norank_	p__Depend	c__Babelia	o__Babelia	f__unclassi	g__unclass	s__unclassi	OTU2395
d__Bacteri	k__norank_	p__Proteob	c__Deltapr	o__Bdellov	f__Bacteric	g__Peredib	s__unclassi	OTU1244
d__Bacteri	k__norank_	p__Proteob	c__Gamma	o__Gamma	f__unclassi	g__Candida	s__uncultu	OTU1675
d__Bacteri	k__norank_	p__Firmicu	c__Negativ	o__Selenor	f__Veillone	g__Megasp	s__uncultu	OTU2217
d__Bacteri	k__norank_	p__Proteob	c__Gamma	o__Vibrion	f__Vibriona	g__Vibrio	s__Vibrio_s	OTU2205
d__Bacteri	k__norank_	p__Firmicu	c__Clostrid	o__Clostrid	f__Rumino	g__Candida	s__uncultu	OTU1492
d__Bacteri	k__norank_	p__Proteob	c__Deltapr	o__Desulfo	f__Desulfo	g__norank_	s__uncultu	OTU1304
d__Bacteri	k__norank_	p__Proteob	c__Deltapr	o__Desulfa	f__Desulfa	g__Desulfa	s__unclassi	OTU2772
d__Bacteri	k__norank_	p__Cyanob	c__Oxypho	o__Chlorop	f__norank_	g__norank_	s__unclassi	OTU1649
d__Bacteri	k__norank_	p__Chlorof	c__Anaerol	o__RBG-13	f__norank_	g__norank_	s__unclassi	OTU2180
d__Bacteri	k__norank_	p__Proteob	c__Gamma	o__Diploric	f__Diploric	g__norank_	s__metage	OTU1804
d__Bacteri	k__norank_	p__Proteob	c__Gamma	o__Diploric	f__Diploric	g__Aquicel	s__unclassi	OTU2522
d__Bacteri	k__norank_	p__Depend	c__Babelia	o__Babelia	f__unclassi	g__unclass	s__unclassi	OTU2268
d__Bacteri	k__norank_	p__Gemma	c__Gemma	o__norank_	f__norank_	g__norank_	s__uncultu	OTU2237
d__Bacteri	k__norank_	p__Proteob	c__Alphapr	o__Acetob	f__Acetoba	g__Roseom	s__metage	OTU187
d__Bacteri	k__norank_	p__Proteob	c__Deltapr	o__Myxocc	f__Haliangi	g__Haliang	s__uncultu	OTU3281
d__Bacteri	k__norank_	p__Proteob	c__Gamma	o__Betapro	f__Methylo	g__Methylo	s__uncultu	OTU775
d__Bacteri	k__norank_	p__Proteob	c__Gamma	o__Diploric	f__Diploric	g__norank_	s__uncultu	OTU1928
d__Bacteri	k__norank_	p__Actinob	c__Actinob	o__Frankia	f__Geoderi	g__Blastoc	s__Blastoc	OTU76
d__Bacteri	k__norank_	p__Proteob	c__Gamma	o__Diploric	f__Diploric	g__norank_	s__unclassi	OTU1739
d__Bacteri	k__norank_	p__Depend	c__Babelia	o__Babelia	f__Vermiph	g__norank_	s__unclassi	OTU2347
d__Bacteri	k__norank_	p__Firmicu	c__Bacilli	o__Bacillal	f__Paeniba	g__norank_	s__metage	OTU1115
d__Bacteri	k__norank_	p__Firmicu	c__Clostrid	o__Clostrid	f__Gracilib	g__Lutispo	s__uncultu	OTU1178
d__Bacteri	k__norank_	p__Bacterc	c__Bactero	o__Flavoba	f__Flavoba	g__Flavoba	s__unclassi	OTU1093
d__Bacteri	k__norank_	p__Firmicu	c__Clostrid	o__Clostrid	f__Rumino	g__Rumino	s__unclassi	OTU131

d__Bacteri	k__norank_	p__Proteol	c__Deltapr	o__Bdellov	f__Bdellovi	g__OM27_	s__uncultu	OTU2818
d__Bacteri	k__norank_	p__Firmicu	c__Clostrid	o__Clostrid	f__Rumino	g__Sporob	s__uncultu	OTU2051
d__Bacteri	k__norank_	p__Actinob	c__Actinob	o__Corynel	f__Coryneb	g__Corynel	s__Coryneb	OTU123
d__Bacteri	k__norank_	p__Bacterc	c__Bactero	o__Cytoph	f__Microsc	g__Chrysec	s__uncultu	OTU1518
d__Bacteri	k__norank_	p__Firmicu	c__Clostrid	o__Clostrid	f__Gracilib	g__Lutispo	s__unclassi	OTU1332
d__Bacteri	k__norank_	p__Proteol	c__Gamma	o__Diploric	f__Diploric	g__norank_	s__unclassi	OTU1755
d__Bacteri	k__norank_	p__Proteol	c__Deltapr	o__Syntrop	f__Syntrop	g__Desulfo	s__uncultu	OTU40
d__Bacteri	k__norank_	p__Proteol	c__Gamma	o__Pasteur	f__Pasteur	g__Aggrega	s__uncultu	OTU1214
d__Bacteri	k__norank_	p__Proteol	c__Gamma	o__Pseudo	f__Moraxe	g__Alkanin	s__uncultu	OTU2721
d__Bacteri	k__norank_	p__Bacterc	c__Bactero	o__Flavoba	f__Flavoba	g__Flavoba	s__unclassi	OTU2608
d__Bacteri	k__norank_	p__Verrucc	c__Verrucc	o__Verrucc	f__Rubrital	g__Luteolik	s__Verruco	OTU3660
d__Bacteri	k__norank_	p__Proteol	c__Gamma	o__Betaprc	f__Burkhol	g__unclass	s__unclassi	OTU2548
d__Bacteri	k__norank_	p__Chlamy	c__Chlamy	o__Chlamy	f__Parachla	g__unclass	s__unclassi	OTU3527
d__Bacteri	k__norank_	p__Proteol	c__Alphapr	o__Azospir	f__Azospiri	g__Azospir	s__Azospiri	OTU823
d__Bacteri	k__norank_	p__Proteol	c__Alphapr	o__Rhizobi	f__KF-JG30	g__norank_	s__metage	OTU3915
d__Bacteri	k__norank_	p__Proteol	c__Deltapr	o__Myxocc	f__Polyang	g__Sorangi	s__unclassi	OTU1872
d__Bacteri	k__norank_	p__Bacterc	c__Bactero	o__Chitino	f__Chitinop	g__Pseudo	s__Niastell	OTU2645
d__Bacteri	k__norank_	p__Proteol	c__Deltapr	o__NB1-j	f__norank_	g__norank_	s__uncultu	OTU2800
d__Bacteri	k__norank_	p__Proteol	c__Deltapr	o__Bdellov	f__Bdellovi	g__Bdellov	s__unclassi	OTU1275
d__Bacteri	k__norank_	p__Proteol	c__Gamma	o__Betaprc	f__Burkhol	g__unclass	s__unclassi	OTU1323
d__Bacteri	k__norank_	p__Proteol	c__Gamma	o__Betaprc	f__Rhodocy	g__Azoarcu	s__unclassi	OTU1814
d__Bacteri	k__norank_	p__Cyanob	c__Sericytc	o__norank_	f__norank_	g__norank_	s__uncultu	OTU1743
d__Bacteri	k__norank_	p__Chlamy	c__Chlamy	o__Chlamy	f__Parachla	g__Candida	s__Candida	OTU2204
d__Bacteri	k__norank_	p__Bacterc	c__Bactero	o__Cytoph	f__Cytopha	g__Cytopha	s__uncultu	OTU636
d__Bacteri	k__norank_	p__Actinob	c__Actinob	o__Catenul	f__Actinos	g__Actinos	s__Actinos	OTU2850
d__Bacteri	k__norank_	p__Firmicu	c__Clostrid	o__Clostrid	f__Clostridi	g__Clostrid	s__uncultu	OTU553
d__Bacteri	k__norank_	p__Proteol	c__Deltapr	o__Bdellov	f__Bacteric	g__Peredib	s__uncultu	OTU1819
d__Bacteri	k__norank_	p__Cyanob	c__Oxypho	o__Chlorop	f__norank_	g__norank_	s__Chlorop	OTU1683
d__Bacteri	k__norank_	p__Chlamy	c__Chlamy	o__Chlamy	f__unclassi	g__unclass	s__unclassi	OTU2301
d__Bacteri	k__norank_	p__Firmicu	c__Clostrid	o__Clostrid	f__Rumino	g__Rumino	s__unclassi	OTU1516
d__Bacteri	k__norank_	p__Gemma	c__Gemma	o__Gemma	f__Gemma	g__norank_	s__uncultu	OTU1742
d__Bacteri	k__norank_	p__Proteol	c__Gamma	o__Diploric	f__Diploric	g__Aquicel	s__uncultu	OTU2122
d__Bacteri	k__norank_	p__Actinob	c__Actinob	o__Micrococ	f__Micrococ	g__Rothia	s__unclassi	OTU634
d__Bacteri	k__norank_	p__Firmicu	c__Clostrid	o__Clostrid	f__Rumino	g__Rumino	s__unclassi	OTU993
d__Bacteri	k__norank_	p__Bacterc	c__Bactero	o__Sphingo	f__Sphingo	g__Sphingo	s__unclassi	OTU2401
d__Bacteri	k__norank_	p__BRC1	c__norank_	o__norank_	f__norank_	g__norank_	s__uncultu	OTU2012
d__Bacteri	k__norank_	p__Bacterc	c__Bactero	o__Chitino	f__Chitinop	g__norank_	s__metage	OTU1210
d__Bacteri	k__norank_	p__Firmicu	c__Clostrid	o__Clostrid	f__Rumino	g__Rumino	s__unclassi	OTU1460
d__Bacteri	k__norank_	p__Firmicu	c__Clostrid	o__Clostrid	f__Lachnos	g__Fusicat	s__uncultu	OTU46
d__Bacteri	k__norank_	p__Firmicu	c__Negativ	o__Selenor	f__Veillone	g__Megam	s__uncultu	OTU130
d__Bacteri	k__norank_	p__Proteol	c__Alphapr	o__Caulob	f__Hyphom	g__Hyphon	s__uncultu	OTU175
d__Bacteri	k__norank_	p__Proteol	c__Gamma	o__Betaprc	f__SC-I-84	g__norank_	s__unclassi	OTU2173
d__Bacteri	k__norank_	p__Proteol	c__Deltapr	o__Oligofle	f__0319-6C	g__norank_	s__unclassi	OTU2725
d__Bacteri	k__norank_	p__Depenc	c__Babelia	o__Babelia	f__Vermipl	g__norank_	s__uncultu	OTU2746
d__Bacteri	k__norank_	p__Depenc	c__Babelia	o__Babelia	f__Babeliac	g__norank_	s__uncultu	OTU2732
d__Bacteri	k__norank_	p__Bacterc	c__Bactero	o__Chitino	f__Chitinop	g__Dinghui	s__unclassi	OTU1081
d__Bacteri	k__norank_	p__Deinoc	c__Deinocc	o__Deinoc	f__Deinocc	g__Deinoc	s__Deinocc	OTU1127

d__Bacteri	k__norank_	p__Actinob	c__Actinob	o__Propion	f__Propion	g__Cutibac	s__Cutibac	OTU665
d__Bacteri	k__norank_	p__Proteob	c__Gamma	o__Betapro	f__Burkhol	g__unclass	s__unclassi	OTU1162
d__Bacteri	k__norank_	p__Proteob	c__Gamma	o__Betapro	f__A21b	g__norank_	s__unclassi	OTU2244
d__Bacteri	k__norank_	p__Actinob	c__Actinob	o__PeM15	f__norank_	g__norank_	s__metage	OTU157
d__Bacteri	k__norank_	p__Proteob	c__Alphapr	o__Acetob	f__Acetoba	g__Acidoce	s__uncultu	OTU2979
d__Bacteri	k__norank_	p__Firmicu	c__Clostrid	o__Clostrid	f__Peptocc	g__Desulfo	s__unclassi	OTU1501
d__Bacteri	k__norank_	p__Bacterc	c__Bactero	o__Cytoph	f__Cycloba	g__Algoripl	s__unclassi	OTU3796
d__Bacteri	k__norank_	p__Proteob	c__Gamma	o__Xantho	f__Xanthor	g__Arenim	s__uncultu	OTU3199
d__Bacteri	k__norank_	p__Proteob	c__Gamma	o__unclass	f__unclassi	g__unclass	s__unclassi	OTU2724
d__Bacteri	k__norank_	p__Proteob	c__Gamma	o__unclass	f__unclassi	g__unclass	s__unclassi	OTU2093
d__Bacteri	k__norank_	p__Proteob	c__Gamma	o__Betapro	f__Neisseri	g__norank_	s__unclassi	OTU2382
d__Bacteri	k__norank_	p__Proteob	c__Gamma	o__Pseudo	f__Moraxe	g__Acineto	s__Acineto	OTU1965
d__Bacteri	k__norank_	p__Gemma	c__Gemma	o__Gemma	f__Gemma	g__norank_	s__uncultu	OTU1881
d__Bacteri	k__norank_	p__Bacterc	c__Bactero	o__Chitino	f__Saprosp	g__Phaeod	s__uncultu	OTU1750
d__Bacteri	k__norank_	p__Firmicu	c__Clostrid	o__Clostrid	f__Clostridi	g__Haloimp	s__unclassi	OTU1112
d__Bacteri	k__norank_	p__Chlorof	c__TK10	o__norank_	f__norank_	g__norank_	s__uncultu	OTU1054
d__Bacteri	k__norank_	p__Bacterc	c__Bactero	o__Chitino	f__Saprosp	g__norank_	s__metage	OTU1147
d__Bacteri	k__norank_	p__Proteob	c__Gamma	o__Betapro	f__Burkhol	g__Comam	s__Comam	OTU2140
d__Bacteri	k__norank_	p__Bacterc	c__Bactero	o__Chitino	f__Chitinop	g__unclass	s__unclassi	OTU1232
d__Bacteri	k__norank_	p__Firmicu	c__Clostrid	o__Clostrid	f__Lachnos	g__Lachnos	s__uncultu	OTU2799
d__Bacteri	k__norank_	p__Proteob	c__Alphapr	o__Rhodob	f__Rhodob	g__Rubellir	s__uncultu	OTU863
d__Bacteri	k__norank_	p__Proteob	c__Gamma	o__unclass	f__unclassi	g__unclass	s__unclassi	OTU2434
d__Bacteri	k__norank_	p__Firmicu	c__Bacilli	o__Lactoba	f__Lactoba	g__Lactoba	s__Lactoba	OTU671
d__Bacteri	k__norank_	p__Bacterc	c__Bactero	o__Bacterc	f__Bactero	g__Bactero	s__Bactero	OTU1658
d__Bacteri	k__norank_	p__Depenc	c__Babelia	o__Babelia	f__Vermipl	g__norank_	s__unclassi	OTU2586
d__Bacteri	k__norank_	p__Actinob	c__Actinob	o__Gaiellal	f__norank_	g__norank_	s__metage	OTU1308
d__Bacteri	k__norank_	p__Bacterc	c__Bactero	o__Bacterc	f__Bactero	g__norank_	s__uncultu	OTU2261
d__Bacteri	k__norank_	p__Plancto	c__OM190	o__norank_	f__norank_	g__norank_	s__uncultu	OTU2428
d__Bacteri	k__norank_	p__Depenc	c__Babelia	o__Babelia	f__Vermipl	g__norank_	s__unclassi	OTU2588
d__Bacteri	k__norank_	p__Proteob	c__Gamma	o__Betapro	f__Neisseri	g__norank_	s__uncultu	OTU1384
d__Bacteri	k__norank_	p__Proteob	c__Gamma	o__Cellvibr	f__Spongiib	g__BD1-7_	s__metage	OTU1662
d__Bacteri	k__norank_	p__Proteob	c__Alphapr	o__Rhizobi	f__Beijerin	g__Methylo	s__Methylo	OTU3012
d__Bacteri	k__norank_	p__Depenc	c__Babelia	o__Babelia	f__Vermipl	g__norank_	s__unclassi	OTU2612
d__Bacteri	k__norank_	p__Proteob	c__Gamma	o__Betapro	f__Burkhol	g__Thiomo	s__mine_d	OTU923
d__Bacteri	k__norank_	p__Proteob	c__Alphapr	o__Paracae	f__Paracae	g__norank_	s__uncultu	OTU1640
d__Bacteri	k__norank_	p__Proteob	c__Alphapr	o__Sphingo	f__Sphingo	g__unclass	s__unclassi	OTU530
d__Bacteri	k__norank_	p__Actinob	c__Actinob	o__Frankia	f__Sporicht	g__norank_	s__metage	OTU3711
d__Bacteri	k__norank_	p__Firmicu	c__Clostrid	o__Clostrid	f__Rumino	g__Ruminc	s__uncultu	OTU1405
d__Bacteri	k__norank_	p__Proteob	c__Deltapr	o__Myxocc	f__Myxoco	g__Corallo	s__Corallo	OTU1915
d__Bacteri	k__norank_	p__Proteob	c__Deltapr	o__Myxocc	f__Blfdi19	g__norank_	s__unclassi	OTU2176
d__Bacteri	k__norank_	p__Proteob	c__Gamma	o__Legione	f__Legione	g__Legione	s__unclassi	OTU1723
d__Bacteri	k__norank_	p__Firmicu	c__Clostrid	o__Clostrid	f__Clostridi	g__Clostrid	s__unclassi	OTU1377
d__Bacteri	k__norank_	p__Bacterc	c__Bactero	o__Bacterc	f__Muribac	g__norank_	s__uncultu	OTU1773
d__Bacteri	k__norank_	p__Firmicu	c__Bacilli	o__Lactoba	f__Strepto	g__Strepto	s__unclassi	OTU781
d__Bacteri	k__norank_	p__Proteob	c__Gamma	o__unclass	f__unclassi	g__unclass	s__unclassi	OTU2414
d__Bacteri	k__norank_	p__Proteob	c__Gamma	o__Betapro	f__Neisseri	g__norank_	s__unclassi	OTU2768
d__Bacteri	k__norank_	p__Bacterc	c__Bactero	o__Bacterc	f__Bactero	g__Bactero	s__Bactero	OTU66

d__Bacteri	k__norank_	p__Depend	c__Babelia	o__Babelia	f__Vermipl	g__norank_	s__uncultu	OTU2606
d__Bacteri	k__norank_	p__Proteob	c__Gamma	o__Gamma	f__unclassi	g__Candida	s__unclassi	OTU2193
d__Bacteri	k__norank_	p__Firmicu	c__Bacilli	o__Bacillal	f__Planoco	g__Planom	s__unclassi	OTU1485
d__Bacteri	k__norank_	p__Firmicu	c__Clostrid	o__Clostrid	f__Rumino	g__unclass	s__unclassi	OTU1225
d__Bacteri	k__norank_	p__Bacterc	c__Bactero	o__Sphingo	f__Sphingo	g__Sphingo	s__Sphingo	OTU1700
d__Bacteri	k__norank_	p__Bacterc	c__Bactero	o__Sphingo	f__Sphingo	g__Sphingo	s__unclassi	OTU1142
d__Bacteri	k__norank_	p__Acidoba	c__Holopha	o__Subgrou	f__norank_	g__norank_	s__unclassi	OTU2789
d__Bacteri	k__norank_	p__Proteob	c__Deltapr	o__Bdellov	f__Bdellovi	g__Bdellov	s__unclassi	OTU1248
d__Bacteri	k__norank_	p__Bacterc	c__Bactero	o__Bacterc	f__unclassi	g__unclass	s__unclassi	OTU1465
d__Bacteri	k__norank_	p__Proteob	c__Gamma	o__Legione	f__Legione	g__Legione	s__uncultu	OTU2738
d__Bacteri	k__norank_	p__Proteob	c__Alphapr	o__Reyran	f__Reyrane	g__Reyrane	s__unclassi	OTU3666
d__Bacteri	k__norank_	p__Latescib	c__Latescib	o__norank_	f__norank_	g__norank_	s__uncultu	OTU1901
d__Bacteri	k__norank_	p__Halanae	c__Halanae	o__Halanae	f__Halanae	g__Halocel	s__uncultu	OTU1542
d__Bacteri	k__norank_	p__Proteob	c__Deltapr	o__Oligofle	f__Oligofle	g__Silvanig	s__unclassi	OTU2487
d__Bacteri	k__norank_	p__Proteob	c__Gamma	o__Pseudo	f__Moraxe	g__Acineto	s__Acineto	OTU844
d__Bacteri	k__norank_	p__Firmicu	c__Clostrid	o__Clostrid	f__Lachnos	g__unclass	s__unclassi	OTU1366
d__Bacteri	k__norank_	p__Proteob	c__Gamma	o__Gamma	f__unclassi	g__Candida	s__uncultu	OTU2202
d__Bacteri	k__norank_	p__Proteob	c__Gamma	o__Betapro	f__Burkhol	g__unclass	s__unclassi	OTU1310
d__Bacteri	k__norank_	p__Chlamy	c__Chlamy	o__Chlamy	f__cvE6	g__norank_	s__uncultu	OTU2499
d__Bacteri	k__norank_	p__Chlamy	c__Chlamy	o__Chlamy	f__Chlamy	g__unclass	s__unclassi	OTU2331
d__Bacteri	k__norank_	p__Chlamy	c__Chlamy	o__Chlamy	f__Simkani	g__unclass	s__unclassi	OTU1893
d__Bacteri	k__norank_	p__Firmicu	c__Clostrid	o__Clostrid	f__Lachnos	g__Eubacte	s__uncultu	OTU2041
d__Bacteri	k__norank_	p__Patescil	c__Sacchar	o__Sacchar	f__Sacchar	g__norank_	s__unclassi	OTU1170
d__Bacteri	k__norank_	p__Proteob	c__Deltapr	o__Bdellov	f__Bdellovi	g__Bdellov	s__unclassi	OTU2519
d__Bacteri	k__norank_	p__Acidoba	c__Blastoca	o__Blastoc	f__Blastoca	g__norank_	s__uncultu	OTU336
d__Bacteri	k__norank_	p__unclass	c__unclassi	o__unclass	f__unclassi	g__unclass	s__unclassi	OTU2782
d__Bacteri	k__norank_	p__unclass	c__unclassi	o__unclass	f__unclassi	g__unclass	s__unclassi	OTU3956
d__Bacteri	k__norank_	p__Proteob	c__Gamma	o__Legione	f__Legione	g__Legione	s__unclassi	OTU930
d__Bacteri	k__norank_	p__Proteob	c__Gamma	o__Betapro	f__Neisseri	g__norank_	s__uncultu	OTU2272
d__Bacteri	k__norank_	p__Actinob	c__Actinob	o__Pseudo	f__Pseudon	g__Sacchar	s__Sacchar	OTU1305
d__Bacteri	k__norank_	p__Verruco	c__Verruco	o__Verruco	f__Rubrital	g__Luteolib	s__uncultu	OTU3426
d__Bacteri	k__norank_	p__Proteob	c__Gamma	o__Legione	f__Legione	g__Legione	s__uncultu	OTU2361
d__Bacteri	k__norank_	p__Bacterc	c__Bactero	o__Flavoba	f__Weekse	g__Chrysec	s__Chrysec	OTU1474
d__Bacteri	k__norank_	p__Proteob	c__Gamma	o__Oceanc	f__Halomo	g__Cobetia	s__unclassi	OTU2682
d__Bacteri	k__norank_	p__Bacterc	c__Bactero	o__Chitino	f__Chitinop	g__Cnuella	s__uncultu	OTU1318
d__Bacteri	k__norank_	p__Bacterc	c__Bactero	o__Bacterc	f__Prevote	g__Allopre	s__uncultu	OTU2134
d__Bacteri	k__norank_	p__Proteob	c__Deltapr	o__SAR324	f__norank_	g__norank_	s__uncultu	OTU2178
d__Bacteri	k__norank_	p__Depend	c__Babelia	o__Babelia	f__Vermipl	g__norank_	s__uncultu	OTU1758
d__Bacteri	k__norank_	p__Firmicu	c__unclassi	o__unclass	f__unclassi	g__unclass	s__unclassi	OTU1609
d__Bacteri	k__norank_	p__Proteob	c__Alphapr	o__Rhizobi	f__Rhizobia	g__norank_	s__uncultu	OTU372
d__Bacteri	k__norank_	p__Firmicu	c__Clostrid	o__Clostrid	f__Peptocc	g__Thermi	s__unclassi	OTU1498
d__Bacteri	k__norank_	p__Proteob	c__Deltapr	o__Myxocc	f__Phaselic	g__Phaseli	s__unclassi	OTU1904
d__Bacteri	k__norank_	p__Depend	c__Babelia	o__Babelia	f__unclassi	g__unclass	s__unclassi	OTU2385
d__Bacteri	k__norank_	p__Depend	c__Babelia	o__Babelia	f__UBA124	g__norank_	s__unclassi	OTU2562
d__Bacteri	k__norank_	p__Bacterc	c__Bactero	o__Cytopha	f__Spirosor	g__Emticici	s__Emticici	OTU1053
d__Bacteri	k__norank_	p__Proteob	c__Alphapr	o__Azospir	f__Azospiri	g__Skerma	s__uncultu	OTU244
d__Bacteri	k__norank_	p__Acidoba	c__Blastoca	o__Pyrinon	f__Pyrinom	g__RB41	s__uncultu	OTU1615

d__Bacteri	k__norank_	p__Firmicu	c__unclassi	o__unclass	f__unclassi	g__unclass	s__unclassi	OTU1041
d__Bacteri	k__norank_	p__Chlamy	c__Chlamy	o__Chlamy	f__cvE6	g__norank_	s__uncultu	OTU2468
d__Bacteri	k__norank_	p__Proteob	c__Gamma	o__Methyl	f__Methylo	g__Methyl	s__uncultu	OTU1968
d__Bacteri	k__norank_	p__Firmicu	c__Clostrid	o__Clostrid	f__Rumino	g__Rumino	s__uncultu	OTU2087
d__Bacteri	k__norank_	p__Rokuba	c__NC10	o__Rokuba	f__norank_	g__norank_	s__uncultu	OTU1593
d__Bacteri	k__norank_	p__Cyanob	c__Melaina	o__Gastran	f__norank_	g__norank_	s__uncultu	OTU1385
d__Bacteri	k__norank_	p__Cyanob	c__Melaina	o__Gastran	f__norank_	g__norank_	s__uncultu	OTU1386
d__Bacteri	k__norank_	p__Chlamy	c__Chlamy	o__Chlamy	f__Parachla	g__unclass	s__unclassi	OTU2743
d__Bacteri	k__norank_	p__Bacterc	c__Bactero	o__Flavoba	f__Flavoba	g__unclass	s__unclassi	OTU1064
d__Bacteri	k__norank_	p__Proteob	c__Deltapr	o__Myxocc	f__P3OB-4	g__norank_	s__uncultu	OTU2023
d__Bacteri	k__norank_	p__Chlorof	c__Anaerol	o__Anaero	f__Anaerol	g__norank_	s__unclassi	OTU1595
d__Bacteri	k__norank_	p__Proteob	c__Alphapr	o__norank_	f__norank_	g__norank_	s__unclassi	OTU360
d__Bacteri	k__norank_	p__Spiroch	c__Spiroch	o__Spiroch	f__Spiroch	g__Spiroch	s__unclassi	OTU1419
d__Bacteri	k__norank_	p__Proteob	c__Gamma	o__Acidithi	f__Acidithi	g__Acidithi	s__unclassi	OTU1296
d__Bacteri	k__norank_	p__Proteob	c__Gamma	o__norank_	f__norank_	g__norank_	s__metage	OTU1975
d__Bacteri	k__norank_	p__Proteob	c__Alphapr	o__Rhizobi	f__Rhizobia	g__unclass	s__unclassi	OTU1569
d__Bacteri	k__norank_	p__Patescil	c__Gracilib	o__Candida	f__norank_	g__norank_	s__unclassi	OTU3375
d__Bacteri	k__norank_	p__Proteob	c__Gamma	o__Diploric	f__Diploric	g__Aquicel	s__unclassi	OTU1722
d__Bacteri	k__norank_	p__Proteob	c__Gamma	o__Acidithi	f__Acidithi	g__Acidithi	s__unclassi	OTU1298
d__Bacteri	k__norank_	p__Firmicu	c__Bacilli	o__Bacillal	f__Paeniba	g__Breviba	s__Breviba	OTU1520
d__Bacteri	k__norank_	p__Firmicu	c__Clostrid	o__Clostrid	f__unclassi	g__unclass	s__unclassi	OTU1155
d__Bacteri	k__norank_	p__Depenc	c__Babelia	o__Babelia	f__Vermiph	g__norank_	s__uncultu	OTU1876
d__Bacteri	k__norank_	p__Proteob	c__Gamma	o__Alteron	f__Marinob	g__Marino	s__uncultu	OTU1519
d__Bacteri	k__norank_	p__Cyanob	c__Oxypho	o__Chlorop	f__norank_	g__norank_	s__unclassi	OTU1612
d__Bacteri	k__norank_	p__Firmicu	c__Negativ	o__Selenor	f__Acidami	g__Acidam	s__uncultu	OTU1798
d__Bacteri	k__norank_	p__Armatir	c__Armatir	o__Armatir	f__norank_	g__norank_	s__uncultu	OTU1487
d__Bacteri	k__norank_	p__Depenc	c__Babelia	o__Babelia	f__unclassi	g__unclass	s__unclassi	OTU2729
d__Bacteri	k__norank_	p__Proteob	c__Gamma	o__Betapro	f__Burkhol	g__unclass	s__unclassi	OTU2153
d__Bacteri	k__norank_	p__Proteob	c__Alphapr	o__Rhodos	f__Magnet	g__norank_	s__uncultu	OTU2073
d__Bacteri	k__norank_	p__Depenc	c__Babelia	o__Babelia	f__unclassi	g__unclass	s__unclassi	OTU2726
d__Bacteri	k__norank_	p__Firmicu	c__Clostrid	o__Clostrid	f__Lachnos	g__Blautia	s__unclassi	OTU134
d__Bacteri	k__norank_	p__Proteob	c__Gamma	o__Betapro	f__Gallione	g__Gallione	s__unclassi	OTU917
d__Bacteri	k__norank_	p__Bacterc	c__Bactero	o__Chitino	f__Saprosp	g__norank_	s__metage	OTU1046
d__Bacteri	k__norank_	p__Chlorof	c__TK10	o__norank_	f__norank_	g__norank_	s__unclassi	OTU2021
d__Bacteri	k__norank_	p__Acidoba	c__Subgrou	o__norank_	f__norank_	g__norank_	s__uncultiv	OTU282
d__Bacteri	k__norank_	p__Firmicu	c__Clostrid	o__Clostrid	f__Clostridi	g__Fontice	s__uncultu	OTU1477
d__Bacteri	k__norank_	p__Chlorof	c__Anaerol	o__Caldilin	f__Caldiline	g__norank_	s__uncultu	OTU1504
d__Bacteri	k__norank_	p__Proteob	c__Gamma	o__Aeromo	f__Aeromo	g__Tolumo	s__Tolumo	OTU1524
d__Bacteri	k__norank_	p__Chlamy	c__Chlamy	o__Chlamy	f__unclassi	g__unclass	s__unclassi	OTU2221
d__Bacteri	k__norank_	p__Actinob	c__Actinob	o__Propior	f__Nocardi	g__Nocardi	s__unclassi	OTU3216
d__Bacteri	k__norank_	p__Depenc	c__Babelia	o__Babelia	f__Babelia	g__norank_	s__unclassi	OTU1908
d__Bacteri	k__norank_	p__Firmicu	c__Clostrid	o__Clostrid	f__Rumino	g__unclass	s__unclassi	OTU1517
d__Bacteri	k__norank_	p__Plancto	c__vadinHA	o__norank_	f__norank_	g__norank_	s__uncultu	OTU61
d__Bacteri	k__norank_	p__Acidoba	c__Holopha	o__Subgrou	f__norank_	g__norank_	s__uncultu	OTU2399
d__Bacteri	k__norank_	p__Proteob	c__Alphapr	o__Rhodos	f__Magnet	g__norank_	s__metage	OTU4002
d__Bacteri	k__norank_	p__Firmicu	c__Clostrid	o__Clostrid	f__Family_	g__Tissiere	s__uncultu	OTU2293
d__Bacteri	k__norank_	p__Proteob	c__Alphapr	o__Caulob	f__Cauloba	g__Phenylo	s__metage	OTU609

d__Bacteri	k__norank_	p__Firmicu	c__Bacilli	o__Bacillal	f__Paeniba	g__Paeniba	s__Paeniba	OTU1219
d__Bacteri	k__norank_	p__Depend	c__Babelia	o__Babelia	f__unclassi	g__unclass	s__unclassi	OTU1701
d__Bacteri	k__norank_	p__Proteob	c__Deltapr	o__Myxocc	f__Archang	g__Anaero	s__uncultu	OTU1374
d__Bacteri	k__norank_	p__Bacterc	c__Bactero	o__Bacterc	f__Rikenell	g__Acetoba	s__uncultu	OTU2639
d__Bacteri	k__norank_	p__Cyanob	c__Sericytc	o__norank_	f__norank_	g__norank_	s__metage	OTU1399
d__Bacteri	k__norank_	p__Depend	c__Babelia	o__Babelia	f__unclassi	g__unclass	s__unclassi	OTU1709
d__Bacteri	k__norank_	p__Verrucc	c__Verrucc	o__Pedosp	f__Pedosph	g__norank_	s__unclassi	OTU1907
d__Bacteri	k__norank_	p__Proteob	c__unclassi	o__unclass	f__unclassi	g__unclass	s__unclassi	OTU2128
d__Bacteri	k__norank_	p__Verrucc	c__Verrucc	o__Verrucc	f__Verruco	g__Prosthe	s__uncultu	OTU1483
d__Bacteri	k__norank_	p__Proteob	c__Deltapr	o__Oligofle	f__Oligofle	g__norank_	s__unclassi	OTU2798
d__Bacteri	k__norank_	p__Proteob	c__Gamma	o__Pseudo	f__Moraxe	g__Acineto	s__Acineto	OTU2020
d__Bacteri	k__norank_	p__Proteob	c__Gamma	o__Gamma	f__unclassi	g__Candida	s__unclassi	OTU2397
d__Bacteri	k__norank_	p__Proteob	c__Gamma	o__Betaprc	f__SC-I-84	g__norank_	s__unclassi	OTU2344
d__Bacteri	k__norank_	p__Proteob	c__Deltapr	o__Oligofle	f__Oligofle	g__norank_	s__unclassi	OTU2797
d__Bacteri	k__norank_	p__Firmicu	c__Bacilli	o__Bacillal	f__Family_	g__Exiguob	s__Exiguob	OTU41
d__Bacteri	k__norank_	p__Proteob	c__Alphapr	o__Caulob	f__Cauloba	g__Brevund	s__Brevund	OTU960
d__Bacteri	k__norank_	p__Proteob	c__Alphapr	o__norank_	f__norank_	g__norank_	s__unclassi	OTU1990
d__Bacteri	k__norank_	p__Depend	c__Babelia	o__Babelia	f__Babelia	g__norank_	s__uncultu	OTU2231
d__Bacteri	k__norank_	p__Bacterc	c__Bactero	o__unclass	f__unclassi	g__unclass	s__unclassi	OTU2653
d__Bacteri	k__norank_	p__Proteob	c__Gamma	o__Betaprc	f__Burkhol	g__Undiba	s__Undiba	OTU2783
d__Bacteri	k__norank_	p__Bacterc	c__Bactero	o__Bacterc	f__Prevote	g__unclass	s__unclassi	OTU2540
d__Bacteri	k__norank_	p__GAL15	c__norank_	o__norank_	f__norank_	g__norank_	s__uncultu	OTU1676
d__Bacteri	k__norank_	p__Proteob	c__Alphapr	o__Ricketts	f__unclassi	g__unclass	s__unclassi	OTU2299
d__Bacteri	k__norank_	p__Depend	c__Babelia	o__Babelia	f__Vermiph	g__norank_	s__uncultu	OTU2754
d__Bacteri	k__norank_	p__Firmicu	c__Clostrid	o__Clostrid	f__Peptocc	g__norank_	s__uncultu	OTU1278
d__Bacteri	k__norank_	p__Bacterc	c__Bactero	o__Sphingo	f__NS11-12	g__norank_	s__uncultu	OTU2279
d__Bacteri	k__norank_	p__Proteob	c__Gamma	o__Legione	f__Legione	g__Legione	s__uncultu	OTU1729
d__Bacteri	k__norank_	p__Patescil	c__Sacchar	o__Sacchar	f__Sacchar	g__norank_	s__uncultu	OTU1448
d__Bacteri	k__norank_	p__Chlamy	c__Chlamy	o__Chlamy	f__cvE6	g__norank_	s__uncultu	OTU1808
d__Bacteri	k__norank_	p__Proteob	c__Alphapr	o__Rhizobi	f__Beijerin	g__alphaI_	s__uncultu	OTU3834
d__Bacteri	k__norank_	p__Depend	c__Babelia	o__Babelia	f__unclassi	g__unclass	s__unclassi	OTU2273
d__Bacteri	k__norank_	p__Proteob	c__Alphapr	o__unclass	f__unclassi	g__unclass	s__unclassi	OTU2067
d__Bacteri	k__norank_	p__Bacterc	c__Bactero	o__Flavoba	f__Flavoba	g__Flavoba	s__unclassi	OTU1714
d__Bacteri	k__norank_	p__Proteob	c__Gamma	o__Steroid	f__Steroidc	g__norank_	s__uncultu	OTU920
d__Bacteri	k__norank_	p__Proteob	c__Gamma	o__Betaprc	f__Burkhol	g__unclass	s__unclassi	OTU4067
d__Bacteri	k__norank_	p__Depend	c__Babelia	o__Babelia	f__Vermiph	g__norank_	s__unclassi	OTU2358
d__Bacteri	k__norank_	p__Chlamy	c__Chlamy	o__Chlamy	f__Parachla	g__Neochla	s__metage	OTU2181
d__Bacteri	k__norank_	p__Proteob	c__Deltapr	o__Oligofle	f__0319-6G	g__norank_	s__uncultu	OTU1396
d__Bacteri	k__norank_	p__Bacterc	c__Bactero	o__Flavoba	f__NS9_ma	g__norank_	s__metage	OTU77
d__Bacteri	k__norank_	p__Bacterc	c__Bactero	o__Chitino	f__Saprosp	g__Phaeod	s__uncultu	OTU2655
d__Bacteri	k__norank_	p__Proteob	c__Deltapr	o__MBNT1	f__norank_	g__norank_	s__uncultu	OTU2333
d__Bacteri	k__norank_	p__unclass	c__unclassi	o__unclass	f__unclassi	g__unclass	s__unclassi	OTU1641
d__Bacteri	k__norank_	p__Firmicu	c__Bacilli	o__Bacillal	f__Alicyclol	g__Acidiba	s__uncultu	OTU1252
d__Bacteri	k__norank_	p__Acidoba	c__Holopha	o__Subgro	f__norank_	g__norank_	s__unclassi	OTU2811
d__Bacteri	k__norank_	p__Firmicu	c__Negativ	o__Selenor	f__Veillone	g__Selenor	s__unclassi	OTU1110
d__Bacteri	k__norank_	p__Bacterc	c__Bactero	o__Chitino	f__Chitinop	g__Ferrugi	s__uncultu	OTU511
d__Bacteri	k__norank_	p__Proteob	c__Gamma	o__Betaprc	f__Nitrosor	g__MND1	s__unclassi	OTU3623

d__Bacteri	k__norank_	p__unclass	c__unclassi	o__unclass	f__unclassi	g__unclass	s__unclassi	OTU1429
d__Bacteri	k__norank_	p__Firmicu	c__Clostrid	o__Clostrid	f__Clostridi	g__Clostrid	s__uncultu	OTU1114
d__Bacteri	k__norank_	p__Depen	c__Babelia	o__Babelia	f__Babelia	g__unclass	s__unclassi	OTU1828
d__Bacteri	k__norank_	p__Proteo	c__Alphapr	o__Caulob	f__Hyphom	g__Hirschia	s__unclassi	OTU348
d__Bacteri	k__norank_	p__Cyanob	c__Oxypho	o__Chloro	f__norank_	g__norank_	s__unclassi	OTU1940
d__Bacteri	k__norank_	p__Firmicu	c__Clostrid	o__Clostrid	f__Family_	g__Eubacte	s__unclassi	OTU820
d__Bacteri	k__norank_	p__Proteo	c__Alphapr	o__Sphing	f__Sphingo	g__Sphing	s__unclassi	OTU536
d__Bacteri	k__norank_	p__Chlamy	c__Chlamy	o__Chlamy	f__Parachl	g__unclass	s__unclassi	OTU991
d__Bacteri	k__norank_	p__Proteo	c__Deltapr	o__Bdellov	f__Bacteri	g__Peredib	s__unclassi	OTU579
d__Bacteri	k__norank_	p__Proteo	c__Gamma	o__Betapr	f__Nitroso	g__Nitroso	s__unclassi	OTU2089
d__Bacteri	k__norank_	p__Depen	c__Babelia	o__Babelia	f__UBA124	g__norank_	s__unclassi	OTU2777
d__Bacteri	k__norank_	p__Verruc	c__Verruc	o__Pedosp	f__Pedosp	g__norank_	s__unclassi	OTU3465
d__Bacteri	k__norank_	p__Actino	c__Actinob	o__Microtr	f__Iamiace	g__Iamia	s__unclassi	OTU1408
d__Bacteri	k__norank_	p__Chlamy	c__Chlamy	o__Chlamy	f__cvE6	g__norank_	s__unclassi	OTU1971
d__Bacteri	k__norank_	p__Proteo	c__Gamma	o__Vibrion	f__Vibrion	g__unclass	s__unclassi	OTU2459
d__Bacteri	k__norank_	p__Bacter	c__Bactero	o__Cytoph	f__Microsc	g__norank_	s__Hassalli	OTU1034
d__Bacteri	k__norank_	p__Depen	c__Babelia	o__Babelia	f__Vermip	g__norank_	s__uncultu	OTU1886
d__Bacteri	k__norank_	p__Actino	c__Actinob	o__Microm	f__Microm	g__Lueden	s__unclassi	OTU2234
d__Bacteri	k__norank_	p__Actino	c__Actinob	o__Microc	f__Breviba	g__Breviba	s__Breviba	OTU1277
d__Bacteri	k__norank_	p__Chlorof	c__Anaerol	o__Anaero	f__Anaerol	g__unclass	s__unclassi	OTU2684
d__Bacteri	k__norank_	p__Firmicu	c__Clostrid	o__Clostrid	f__Family_	g__Tissiere	s__unclassi	OTU1605
d__Bacteri	k__norank_	p__Bacter	c__Bactero	o__Bacter	f__Rikenell	g__Alistipe	s__unclassi	OTU1475
d__Bacteri	k__norank_	p__Proteo	c__Deltapr	o__Bradym	f__norank_	g__norank_	s__uncultu	OTU2840
d__Bacteri	k__norank_	p__Bacter	c__Bactero	o__Bacter	f__SB-5	g__norank_	s__unclassi	OTU2622
d__Bacteri	k__norank_	p__Bacter	c__Bactero	o__Bacter	f__Porphyr	g__Porphy	s__uncultu	OTU64
d__Bacteri	k__norank_	p__Bacter	c__Ignavib	o__OPB56	f__norank_	g__norank_	s__unclassi	OTU2066
d__Bacteri	k__norank_	p__Firmicu	c__Clostrid	o__Clostrid	f__Clostridi	g__Hathew	s__unclassi	OTU1966
d__Bacteri	k__norank_	p__Firmicu	c__Bacilli	o__Bacillal	f__Paeniba	g__Breviba	s__Breviba	OTU1333
d__Bacteri	k__norank_	p__Acidob	c__Acidoba	o__Solibac	f__Solibact	g__Paludib	s__uncultu	OTU2246
d__Bacteri	k__norank_	p__Bacter	c__Bactero	o__Chitino	f__Chitino	g__norank_	s__unclassi	OTU846
d__Bacteri	k__norank_	p__unclass	c__unclassi	o__unclass	f__unclassi	g__unclass	s__unclassi	OTU1328
d__Bacteri	k__norank_	p__Bacter	c__Bactero	o__Bacter	f__Rikenell	g__Rikenel	s__uncultu	OTU2520
d__Bacteri	k__norank_	p__Cyanob	c__Oxypho	o__Synech	f__Cyanobi	g__Cyanob	s__uncultu	OTU83
d__Bacteri	k__norank_	p__Depen	c__Babelia	o__Babelia	f__UBA124	g__norank_	s__uncultu	OTU2753
d__Bacteri	k__norank_	p__Chlamy	c__Chlamy	o__Chlamy	f__unclassi	g__unclass	s__unclassi	OTU2482
d__Bacteri	k__norank_	p__Proteo	c__Gamma	o__Diploric	f__Diploric	g__norank_	s__unclassi	OTU2717
d__Bacteri	k__norank_	p__Chlamy	c__Chlamy	o__Chlamy	f__Parachl	g__unclass	s__unclassi	OTU2376
d__Bacteri	k__norank_	p__Proteo	c__Deltapr	o__Bdellov	f__Bacteri	g__Bacteri	s__uncultu	OTU1598
d__Bacteri	k__norank_	p__Proteo	c__Gamma	o__Coxiella	f__Coxiella	g__Coxiella	s__uncultu	OTU2529
d__Bacteri	k__norank_	p__Firmicu	c__Clostrid	o__Clostrid	f__Rumino	g__Oscillos	s__uncultu	OTU2161
d__Bacteri	k__norank_	p__Firmicu	c__Clostrid	o__DTU01	f__norank_	g__norank_	s__uncultu	OTU1561
d__Bacteri	k__norank_	p__Firmicu	c__Bacilli	o__Bacillal	f__Bacillac	g__Bacillus	s__unclassi	OTU1243
d__Bacteri	k__norank_	p__Proteo	c__Gamma	o__Betapr	f__SC-I-84	g__norank_	s__uncultu	OTU2453
d__Bacteri	k__norank_	p__Bacter	c__Bactero	o__Flavoba	f__Cryomo	g__norank_	s__uncultu	OTU1698
d__Bacteri	k__norank_	p__Bacter	c__Bactero	o__Chitino	f__Chitino	g__norank_	s__metage	OTU1449
d__Bacteri	k__norank_	p__Acidob	c__Acidoba	o__Subgro	f__norank_	g__norank_	s__uncultu	OTU2847
d__Bacteri	k__norank_	p__Depen	c__Babelia	o__Babelia	f__unclassi	g__unclass	s__unclassi	OTU1818

d__Bacteri	k__norank_	p__Proteo	c__Gamma	o__Diplori	f__Diploric	g__norank_	s__unclassi	OTU204
d__Bacteri	k__norank_	p__Firmicu	c__Clostrid	o__Clostri	f__Rumino	g__Rumino	s__unclassi	OTU2018
d__Bacteri	k__norank_	p__Proteo	c__Gamma	o__Coxiell	f__Coxiella	g__Coxiella	s__uncultu	OTU2292
d__Bacteri	k__norank_	p__Bacter	c__Bactero	o__Sphing	f__Sphingo	g__Arcticib	s__unclassi	OTU1289
d__Bacteri	k__norank_	p__unclass	c__unclassi	o__unclass	f__unclassi	g__unclass	s__unclassi	OTU2310
d__Bacteri	k__norank_	p__Cyanob	c__Melain	o__Gastra	f__norank_	g__norank_	s__unclassi	OTU4038
d__Bacteri	k__norank_	p__Actino	c__Actinob	o__Gaiella	f__norank_	g__norank_	s__uncultu	OTU1996
d__Bacteri	k__norank_	p__Actino	c__Actinob	o__Microtr	f__Ilumato	g__CL500-	s__uncultu	OTU2045
d__Bacteri	k__norank_	p__Proteo	c__Gamma	o__Xantho	f__Rhodan	g__norank_	s__uncultu	OTU726
d__Bacteri	k__norank_	p__Depen	c__Babelia	o__Babelia	f__UBA124	g__norank_	s__uncultu	OTU1813
d__Bacteri	k__norank_	p__Firmicu	c__Clostrid	o__Clostri	f__Rumino	g__Rumino	s__uncultu	OTU1066
d__Bacteri	k__norank_	p__Bacter	c__Bactero	o__Flavob	f__Weekse	g__Cloacib	s__unclassi	OTU1226
d__Bacteri	k__norank_	p__Depen	c__Babelia	o__Babelia	f__Babelia	g__unclass	s__unclassi	OTU2139
d__Bacteri	k__norank_	p__Depen	c__Babelia	o__Babelia	f__unclassi	g__unclass	s__unclassi	OTU2430
d__Bacteri	k__norank_	p__Depen	c__Babelia	o__Babelia	f__unclassi	g__unclass	s__unclassi	OTU2709
d__Bacteri	k__norank_	p__Depen	c__Babelia	o__Babelia	f__unclassi	g__unclass	s__unclassi	OTU1925
d__Bacteri	k__norank_	p__Bacter	c__Bactero	o__Bacter	f__Bactero	g__Bacter	s__Bactero	OTU2294
d__Bacteri	k__norank_	p__Proteo	c__Gamma	o__Xantho	f__Rhodan	g__Rhodan	s__uncultu	OTU2986
d__Bacteri	k__norank_	p__Proteo	c__Gamma	o__unclass	f__unclassi	g__unclass	s__unclassi	OTU2575
d__Bacteri	k__norank_	p__Proteo	c__Gamma	o__PLTA13	f__norank_	g__norank_	s__uncultu	OTU2354
d__Bacteri	k__norank_	p__Firmicu	c__Bacilli	o__Bacilla	f__Paeniba	g__Paeniba	s__unclassi	OTU1171
d__Bacteri	k__norank_	p__Firmicu	c__Bacilli	o__Bacilla	f__Alicyclo	g__Tumeba	s__unclassi	OTU1204
d__Bacteri	k__norank_	p__Proteo	c__Gamma	o__Coxiell	f__Coxiella	g__Coxiella	s__unclassi	OTU2257
d__Bacteri	k__norank_	p__Chlorof	c__Anaerol	o__Caldilin	f__Caldilin	g__norank_	s__metage	OTU607
d__Bacteri	k__norank_	p__Proteo	c__Deltapr	o__Sva048	f__norank_	g__norank_	s__uncultu	OTU170
d__Bacteri	k__norank_	p__Chlamy	c__Chlamy	o__Chlamy	f__cvE6	g__norank_	s__metage	OTU2336
d__Bacteri	k__norank_	p__Chlorof	c__Anaerol	o__RBG-13	f__norank_	g__norank_	s__metage	OTU1783
d__Bacteri	k__norank_	p__Bacter	c__Bactero	o__Bacter	f__Muriba	g__norank_	s__uncultu	OTU2582
d__Bacteri	k__norank_	p__Plancto	c__Plancto	o__Gemma	f__Gemma	g__norank_	s__unclassi	OTU1863
d__Bacteri	k__norank_	p__Bacter	c__Bactero	o__Cytoph	f__Hymeno	g__Adhaer	s__unclassi	OTU1036
d__Bacteri	k__norank_	p__Proteo	c__Gamma	o__Betapr	f__Burkhol	g__Rhizob	s__uncultu	OTU3592
d__Bacteri	k__norank_	p__Bacter	c__Bactero	o__Flavob	f__Flavoba	g__Flavoba	s__unclassi	OTU1874
d__Bacteri	k__norank_	p__Firmicu	c__Bacilli	o__Bacilla	f__Planoco	g__Lysiniba	s__unclassi	OTU1525
d__Bacteri	k__norank_	p__Chlorof	c__Chlorof	o__Thermo	f__JG30-KF	g__norank_	s__uncultu	OTU1622
d__Bacteri	k__norank_	p__Cyanob	c__Oxypho	o__Chloro	f__norank_	g__norank_	s__uncultu	OTU1851
d__Bacteri	k__norank_	p__Cyanob	c__Oxypho	o__Chloro	f__norank_	g__norank_	s__Castane	OTU1450
d__Bacteri	k__norank_	p__Proteo	c__Gamma	o__Betapr	f__Burkhol	g__unclass	s__unclassi	OTU1959
d__Bacteri	k__norank_	p__Plancto	c__Phycisp	o__Phycisp	f__Phycispl	g__CL500-	s__uncultu	OTU1638
d__Bacteri	k__norank_	p__Bacter	c__Bactero	o__Bacter	f__unclassi	g__unclass	s__unclassi	OTU2476
d__Bacteri	k__norank_	p__Proteo	c__Gamma	o__Diplori	f__Diploric	g__norank_	s__unclassi	OTU2150
d__Bacteri	k__norank_	p__Actino	c__Actinob	o__Frankia	f__Geoder	g__Blastoc	s__unclassi	OTU1033
d__Bacteri	k__norank_	p__Proteo	c__Gamma	o__Betapr	f__Gallione	g__Gallion	s__unclassi	OTU3003
d__Bacteri	k__norank_	p__Firmicu	c__Bacilli	o__Bacilla	f__unclassi	g__unclass	s__unclassi	OTU1356
d__Bacteri	k__norank_	p__Proteo	c__Gamma	o__Diplori	f__Diploric	g__Aquicel	s__unclassi	OTU1812
d__Bacteri	k__norank_	p__Depen	c__Babelia	o__Babelia	f__Babelia	g__norank_	s__uncultu	OTU2536
d__Bacteri	k__norank_	p__Bacter	c__Bactero	o__Chitino	f__Saprosp	g__norank_	s__metage	OTU1050
d__Bacteri	k__norank_	p__Proteo	c__Gamma	o__Gamma	f__unclassi	g__Candida	s__uncultu	OTU1728

d__Bacteri	k__norank_	p__Bacterc	c__Bactero	o__Flavoba	f__Cryomo	g__norank_	s__uncultu	OTU2666
d__Bacteri	k__norank_	p__Bacterc	c__Ignavib	o__Kryptor	f__BSV26	g__norank_	s__uncultu	OTU2618
d__Bacteri	k__norank_	p__Bacterc	c__Bactero	o__Bacterc	f__Muribac	g__norank_	s__uncultu	OTU2057
d__Bacteri	k__norank_	p__Proteol	c__Gamma	o__Diploric	f__Diploric	g__Aquicel	s__unclassi	OTU2613
d__Bacteri	k__norank_	p__Proteol	c__Gamma	o__Xantho	f__Xanthor	g__Vulcani	s__uncultu	OTU696
d__Bacteri	k__norank_	p__Proteol	c__Gamma	o__Legione	f__Legione	g__Legione	s__unclassi	OTU93
d__Bacteri	k__norank_	p__Bacterc	c__Bactero	o__Bacterc	f__Rikenell	g__Rikenel	s__uncultu	OTU1890
d__Bacteri	k__norank_	p__Firmicu	c__Clostrid	o__Clostrid	f__Lachnos	g__Lachno	s__uncultu	OTU787
d__Bacteri	k__norank_	p__Firmicu	c__Clostrid	o__Clostrid	f__Rumino	g__Caproic	s__uncultu	OTU1107
d__Bacteri	k__norank_	p__Cyanob	c__Melaina	o__Obscuri	f__norank_	g__norank_	s__uncultu	OTU2063
d__Bacteri	k__norank_	p__Proteol	c__Alphapr	o__Rhizobi	f__Rhizobia	g__Allorhiz	s__unclassi	OTU151
d__Bacteri	k__norank_	p__Bacterc	c__Bactero	o__Chitino	f__Chitinop	g__Flavisol	s__uncultu	OTU1084
d__Bacteri	k__norank_	p__Proteol	c__Alphapr	o__Acetob	f__Acetoba	g__Acidiph	s__unclassi	OTU1300
d__Bacteri	k__norank_	p__Proteol	c__Gamma	o__Diploric	f__Diploric	g__norank_	s__uncultu	OTU1736
d__Bacteri	k__norank_	p__Bacterc	c__Ignavib	o__Ignavib	f__PHOS-H	g__norank_	s__uncultu	OTU1711
d__Bacteri	k__norank_	p__Proteol	c__Deltapr	o__Desulfo	f__Desulfol	g__unclass	s__unclassi	OTU2775
d__Bacteri	k__norank_	p__Deinoc	c__Deinocc	o__Deinoc	f__Deinocc	g__Deinoc	s__Deinocc	OTU1376
d__Bacteri	k__norank_	p__Proteol	c__Gamma	o__Betapr	f__T34	g__norank_	s__metage	OTU2328
d__Bacteri	k__norank_	p__Firmicu	c__Clostrid	o__Clostrid	f__Rumino	g__Ruminc	s__unclassi	OTU1453
d__Bacteri	k__norank_	p__Proteol	c__Gamma	o__Betapr	f__Burkhol	g__Polaron	s__unclassi	OTU2532
d__Bacteri	k__norank_	p__Chlamy	c__Chlamy	o__Chlamy	f__cvE6	g__norank_	s__uncultu	OTU2450
d__Bacteri	k__norank_	p__Firmicu	c__Clostrid	o__Clostrid	f__Rumino	g__Eubacte	s__unclassi	OTU1646
d__Bacteri	k__norank_	p__Chlamy	c__Chlamy	o__Chlamy	f__cvE6	g__norank_	s__uncultu	OTU2104
d__Bacteri	k__norank_	p__Bacterc	c__Bactero	o__Cytoph	f__Spirosoı	g__Larkine	s__unclassi	OTU1105
d__Bacteri	k__norank_	p__Proteol	c__unclassi	o__unclass	f__unclassi	g__unclass	s__unclassi	OTU1581
d__Bacteri	k__norank_	p__Chlorof	c__Anaerol	o__Caldilin	f__Caldiline	g__norank_	s__uncultu	OTU1802
d__Bacteri	k__norank_	p__Verrucc	c__Verrucc	o__Verrucc	f__unclassi	g__unclass	s__unclassi	OTU1250
d__Bacteri	k__norank_	p__Proteol	c__Gamma	o__Diploric	f__Diploric	g__Aquicel	s__uncultu	OTU1753
d__Bacteri	k__norank_	p__Bacterc	c__Bactero	o__Bacterc	f__Prevote	g__Prevote	s__uncultu	OTU1699
d__Bacteri	k__norank_	p__Depenc	c__Babelia	o__Babelia	f__UBA124	g__norank_	s__unclassi	OTU2550
d__Bacteri	k__norank_	p__Depenc	c__Babelia	o__Babelia	f__Vermipl	g__norank_	s__unclassi	OTU1713
d__Bacteri	k__norank_	p__Actinok	c__Actinob	o__Corynel	f__Coryneb	g__Lawson	s__uncultu	OTU964
d__Bacteri	k__norank_	p__Depenc	c__Babelia	o__Babelia	f__unclassi	g__unclass	s__unclassi	OTU2284
d__Bacteri	k__norank_	p__Proteol	c__Deltapr	o__Oligofle	f__Oligofle	g__Silvanig	s__unclassi	OTU3054
d__Bacteri	k__norank_	p__Depenc	c__Babelia	o__Babelia	f__unclassi	g__unclass	s__unclassi	OTU2283
d__Bacteri	k__norank_	p__Proteol	c__Gamma	o__Legione	f__Legione	g__Legione	s__uncultu	OTU1735
d__Bacteri	k__norank_	p__Chlorof	c__Anaerol	o__Ardenti	f__norank_	g__norank_	s__unclassi	OTU2131
d__Bacteri	k__norank_	p__Chlamy	c__Chlamy	o__Chlamy	f__cvE6	g__norank_	s__metage	OTU2081
d__Bacteri	k__norank_	p__Bacterc	c__Bactero	o__Bacterc	f__Prevote	g__Prevote	s__unclassi	OTU799
d__Bacteri	k__norank_	p__Proteol	c__Alphapr	o__Rhodos	f__AEGEAN	g__norank_	s__unclassi	OTU879
d__Bacteri	k__norank_	p__Proteol	c__Alphapr	o__Acetob	f__Acetoba	g__unclass	s__unclassi	OTU2966
d__Bacteri	k__norank_	p__Depenc	c__Babelia	o__Babelia	f__Babelia	g__norank_	s__candida	OTU2198
d__Bacteri	k__norank_	p__Proteol	c__Deltapr	o__Bdellov	f__Bacteric	g__Peredib	s__unclassi	OTU1179
d__Bacteri	k__norank_	p__Proteol	c__Deltapr	o__Desulfu	f__Geobac	g__Geobac	s__uncultu	OTU2987
d__Bacteri	k__norank_	p__Firmicu	c__Clostrid	o__Clostrid	f__Rumino	g__Ruminc	s__uncultu	OTU1358
d__Bacteri	k__norank_	p__Depenc	c__Babelia	o__Babelia	f__Vermipl	g__norank_	s__unclassi	OTU1903
d__Bacteri	k__norank_	p__Bacterc	c__Bactero	o__Bacterc	f__Rikenell	g__Alistipe	s__uncultu	OTU2404

d__Bacteri	k__norank_	p__Firmicu	c__Clostrid	o__Clostrid	f__Rumino	g__norank_	s__unclassi	OTU1156
d__Bacteri	k__norank_	p__Proteol	c__Gamma	o__Enterol	f__Enterob	g__Escheri	s__Escheri	OTU947
d__Bacteri	k__norank_	p__Depenc	c__Babelia	o__Babelia	f__Vermipl	g__norank_	s__unclassi	OTU1909
d__Bacteri	k__norank_	p__Acidoba	c__Subgrou	o__norank_	f__norank_	g__norank_	s__uncultu	OTU2439
d__Bacteri	k__norank_	p__Plancto	c__Plancto	o__Gemma	f__Gemma	g__norank_	s__uncultu	OTU2265
d__Bacteri	k__norank_	p__Firmicu	c__Clostrid	o__Clostrid	f__Rumino	g__Ruminic	s__uncultu	OTU2060
d__Bacteri	k__norank_	p__Bacterc	c__Bactero	o__Sphingo	f__Sphingo	g__Pedoba	s__unclassi	OTU1129
d__Bacteri	k__norank_	p__Firmicu	c__Clostrid	o__Clostrid	f__Clostridi	g__unclass	s__unclassi	OTU1100
d__Bacteri	k__norank_	p__Proteol	c__Deltapr	o__Desulfo	f__Desulfol	g__SEEP-SF	s__uncultu	OTU2637
d__Bacteri	k__norank_	p__Proteol	c__Gamma	o__Betaprc	f__Burkhol	g__Thiomo	s__Thiomo	OTU554
d__Bacteri	k__norank_	p__Gemma	c__Gemma	o__Gemma	f__Gemma	g__norank_	s__unclassi	OTU2373
d__Bacteri	k__norank_	p__Proteol	c__Gamma	o__Pseudo	f__Pseudor	g__Pseudo	s__Pseudor	OTU2228
d__Bacteri	k__norank_	p__Firmicu	c__Bacilli	o__Bacillal	f__Paeniba	g__Paeniba	s__unclassi	OTU1500
d__Bacteri	k__norank_	p__Proteol	c__Deltapr	o__NB1-j	f__norank_	g__norank_	s__unclassi	OTU1230
d__Bacteri	k__norank_	p__Proteol	c__Gamma	o__Diploric	f__Diploric	g__norank_	s__unclassi	OTU108
d__Bacteri	k__norank_	p__Proteol	c__Gamma	o__Diploric	f__Diploric	g__norank_	s__unclassi	OTU2816
d__Bacteri	k__norank_	p__Proteol	c__Deltapr	o__RCP2-5	f__norank_	g__norank_	s__unclassi	OTU2219
d__Bacteri	k__norank_	p__Proteol	c__Gamma	o__Betaprc	f__Burkhol	g__Burkhol	s__unclassi	OTU1528
d__Bacteri	k__norank_	p__Chlorof	c__Anaerol	o__Anaero	f__Anaerol	g__norank_	s__unclassi	OTU1737
d__Bacteri	k__norank_	p__Actinob	c__Actinob	o__Corynel	f__Coryneb	g__Corynel	s__Coryneb	OTU85
d__Bacteri	k__norank_	p__Proteol	c__Gamma	o__Legione	f__Legione	g__Legione	s__unclassi	OTU3588
d__Bacteri	k__norank_	p__Proteol	c__Gamma	o__Diploric	f__Diploric	g__Ricketts	s__unclassi	OTU888
d__Bacteri	k__norank_	p__Depenc	c__Babelia	o__Babelia	f__unclassi	g__unclass	s__unclassi	OTU2446
d__Bacteri	k__norank_	p__Depenc	c__Babelia	o__Babelia	f__unclassi	g__unclass	s__unclassi	OTU2442
d__Bacteri	k__norank_	p__Actinob	c__Actinob	o__norank_	f__norank_	g__norank_	s__uncultu	OTU1953
d__Bacteri	k__norank_	p__Proteol	c__Gamma	o__Legione	f__Legione	g__Legione	s__unclassi	OTU2501
d__Bacteri	k__norank_	p__Proteol	c__Gamma	o__Pseudo	f__Moraxe	g__Acineto	s__Acineto	OTU158
d__Bacteri	k__norank_	p__Proteol	c__Gamma	o__Betaprc	f__Burkhol	g__unclass	s__unclassi	OTU1228
d__Bacteri	k__norank_	p__Proteol	c__Gamma	o__Legione	f__Legione	g__Legione	s__unclassi	OTU2188
d__Bacteri	k__norank_	p__Proteol	c__Gamma	o__Pseudo	f__Pseudor	g__Pseudo	s__unclassi	OTU2788
d__Bacteri	k__norank_	p__Proteol	c__Alphapr	o__Caulob	f__Cauloba	g__norank_	s__unclassi	OTU1045
d__Bacteri	k__norank_	p__Firmicu	c__Clostrid	o__Clostrid	f__Family_	g__Anaero	s__uncultu	OTU1260
d__Bacteri	k__norank_	p__Proteol	c__Gamma	o__Pseudo	f__Moraxe	g__Psychrc	s__Psychro	OTU2516
d__Bacteri	k__norank_	p__Firmicu	c__Clostrid	o__Clostrid	f__Lachnos	g__unclass	s__unclassi	OTU2702
d__Bacteri	k__norank_	p__Depenc	c__Babelia	o__Babelia	f__unclassi	g__unclass	s__unclassi	OTU2701
d__Bacteri	k__norank_	p__Lentisp	c__Lentispl	o__Victival	f__vadinBE	g__norank_	s__uncultu	OTU1651
d__Bacteri	k__norank_	p__Bacterc	c__Bactero	o__Sphingo	f__Sphingo	g__Pedoba	s__uncultu	OTU1442
d__Bacteri	k__norank_	p__Depenc	c__Babelia	o__Babelia	f__unclassi	g__unclass	s__unclassi	OTU2706
d__Bacteri	k__norank_	p__Chlamy	c__Chlamy	o__Chlamy	f__Parachl	g__Candid	s__metage	OTU2699
d__Bacteri	k__norank_	p__Omnitr	c__norank_	o__norank_	f__norank_	g__norank_	s__uncultu	OTU1717
d__Bacteri	k__norank_	p__Depenc	c__Babelia	o__Babelia	f__Vermipl	g__norank_	s__uncultu	OTU2690
d__Bacteri	k__norank_	p__Firmicu	c__Bacilli	o__Bacillal	f__Alicyclol	g__Tumeba	s__uncultu	OTU1378
d__Bacteri	k__norank_	p__Bacterc	c__Bactero	o__Sphingo	f__Sphingo	g__Sphingo	s__Sphingo	OTU1468
d__Bacteri	k__norank_	p__Depenc	c__Babelia	o__Babelia	f__Vermipl	g__norank_	s__uncultu	OTU2695
d__Bacteri	k__norank_	p__Depenc	c__Babelia	o__Babelia	f__norank_	g__norank_	s__uncultu	OTU2186
d__Bacteri	k__norank_	p__Firmicu	c__Bacilli	o__Bacillal	f__Bacillac	g__Bacillus	s__unclassi	OTU128
d__Bacteri	k__norank_	p__Firmicu	c__Clostrid	o__Clostrid	f__Rumino	g__Ruminc	s__unclassi	OTU1799

d__Bacteri	k__norank_	p__Bacterc	c__Bactero	o__Cytoph	f__Hymenc	g__Nibriba	s__uncultu	OTU1007
d__Bacteri	k__norank_	p__Depenc	c__Babelia	o__Babelia	f__unclassi	g__unclass	s__unclassi	OTU2491
d__Bacteri	k__norank_	p__Acidoba	c__Subgrou	o__norank_	f__norank_	g__norank_	s__unclassi	OTU2667
d__Bacteri	k__norank_	p__Acidoba	c__Subgrou	o__norank_	f__norank_	g__norank_	s__unclassi	OTU2669
d__Bacteri	k__norank_	p__Proteol	c__Alphapr	o__norank_	f__norank_	g__norank_	s__unclassi	OTU2061
d__Bacteri	k__norank_	p__Chlamy	c__Chlamy	o__Chlamy	f__unclassi	g__unclass	s__unclassi	OTU2364
d__Bacteri	k__norank_	p__Bacterc	c__Rhodotl	o__Rhodot	f__Rhodotl	g__unclass	s__unclassi	OTU1334
d__Bacteri	k__norank_	p__Acidoba	c__Acidoba	o__Solibac	f__Solibact	g__GOUTB	s__unclassi	OTU1104
d__Bacteri	k__norank_	p__Bacterc	c__Bactero	o__Bacterc	f__Prevote	g__Prevote	s__unclassi	OTU2260
d__Bacteri	k__norank_	p__Depenc	c__Babelia	o__Babelia	f__norank_	g__norank_	s__uncultu	OTU2121
d__Bacteri	k__norank_	p__Cyanob	c__Oxypho	o__Chlorop	f__norank_	g__norank_	s__Virgulin	OTU2398
d__Bacteri	k__norank_	p__Depenc	c__Babelia	o__Babelia	f__Vermipl	g__norank_	s__uncultu	OTU2216
d__Bacteri	k__norank_	p__Firmicu	c__Bacilli	o__Bacillal	f__Paeniba	g__Paeniba	s__Paeniba	OTU1164
d__Bacteri	k__norank_	p__Firmicu	c__Clostrid	o__Clostrid	f__Peptost	g__Proteoc	s__uncultu	OTU2791
d__Bacteri	k__norank_	p__Firmicu	c__Clostrid	o__Clostrid	f__Lachnos	g__Lachnos	s__uncultu	OTU1849
d__Bacteri	k__norank_	p__Proteol	c__Gamma	o__unclass	f__unclassi	g__unclass	s__unclassi	OTU2369
d__Bacteri	k__norank_	p__Proteol	c__Alphapr	o__Holosp	f__Holospc	g__norank_	s__unclassi	OTU2036
d__Bacteri	k__norank_	p__Proteol	c__Gamma	o__Betapro	f__Burkhol	g__Limnob	s__unclassi	OTU1704
d__Bacteri	k__norank_	p__Actinol	c__Actinob	o__Microtr	f__Ilumato	g__Ilumatc	s__uncultu	OTU1568
d__Bacteri	k__norank_	p__Proteol	c__Gamma	o__Diploric	f__Diploric	g__Aquicel	s__uncultu	OTU1679
d__Bacteri	k__norank_	p__Patescil	c__Sacchar	o__Sacchar	f__norank_	g__norank_	s__uncultu	OTU1858
d__Bacteri	k__norank_	p__Proteol	c__Gamma	o__Diploric	f__Diploric	g__norank_	s__unclassi	OTU2033
d__Bacteri	k__norank_	p__Firmicu	c__Clostrid	o__Clostrid	f__Clostridi	g__unclass	s__unclassi	OTU2028
d__Bacteri	k__norank_	p__Bacterc	c__Bactero	o__Flavoba	f__Flavoba	g__Flavoba	s__Flavoba	OTU1351
d__Bacteri	k__norank_	p__Acidoba	c__Blastoca	o__Blastoc	f__Blastoca	g__unclass	s__unclassi	OTU227
d__Bacteri	k__norank_	p__Bacterc	c__Bactero	o__Flavoba	f__Weekse	g__Chrysec	s__Candida	OTU60
d__Bacteri	k__norank_	p__Verrucc	c__Verrucc	o__norank_	f__norank_	g__norank_	s__metage	OTU1159
d__Bacteri	k__norank_	p__Proteol	c__Alphapr	o__Acetob	f__Acetoba	g__Acidiph	s__uncultu	OTU1302
d__Bacteri	k__norank_	p__Depenc	c__Babelia	o__Babelia	f__Vermipl	g__norank_	s__uncultu	OTU2408
d__Bacteri	k__norank_	p__Acidoba	c__Subgrou	o__norank_	f__norank_	g__norank_	s__unclassi	OTU773
d__Bacteri	k__norank_	p__Proteol	c__Gamma	o__Diploric	f__Diploric	g__norank_	s__metage	OTU2611
d__Bacteri	k__norank_	p__Firmicu	c__Clostrid	o__Clostrid	f__Syntrop	g__Syntrop	s__uncultu	OTU727
d__Bacteri	k__norank_	p__Proteol	c__unclassi	o__unclass	f__unclassi	g__unclass	s__unclassi	OTU2465
d__Bacteri	k__norank_	p__Depenc	c__Babelia	o__Babelia	f__Vermipl	g__norank_	s__uncultu	OTU1673
d__Bacteri	k__norank_	p__Cyanob	c__Sericytc	o__norank_	f__norank_	g__norank_	s__Hyalope	OTU1383
d__Bacteri	k__norank_	p__Proteol	c__Alphapr	o__unclass	f__unclassi	g__unclass	s__unclassi	OTU2047
d__Bacteri	k__norank_	p__Depenc	c__Babelia	o__Babelia	f__unclassi	g__unclass	s__unclassi	OTU2291
d__Bacteri	k__norank_	p__Proteol	c__Gamma	o__211ds2	f__norank_	g__norank_	s__uncultu	OTU3653
d__Bacteri	k__norank_	p__Chlamy	c__Chlamy	o__Chlamy	f__Simkani	g__norank_	s__unclassi	OTU3522
d__Bacteri	k__norank_	p__Proteol	c__Gamma	o__Betapro	f__Burkhol	g__Achrom	s__unclassi	OTU1503
d__Bacteri	k__norank_	p__Bacterc	c__Bactero	o__Bacterc	f__Muribac	g__unclass	s__unclassi	OTU2664
d__Bacteri	k__norank_	p__Proteol	c__Deltapr	o__Myxocc	f__Polyang	g__Pajaroe	s__uncultu	OTU1043
d__Bacteri	k__norank_	p__Patescil	c__Parcuba	o__Candida	f__norank_	g__norank_	s__unclassi	OTU456
d__Bacteri	k__norank_	p__Bacterc	c__Bactero	o__Flavoba	f__Crocinit	g__Fluviico	s__unclassi	OTU1132
d__Bacteri	k__norank_	p__Bacterc	c__Bactero	o__Bacterc	f__Tannere	g__Tannere	s__uncultu	OTU817
d__Bacteri	k__norank_	p__Bacterc	c__Bactero	o__Sphingo	f__Sphingo	g__Sphingo	s__Sphingo	OTU1444
d__Bacteri	k__norank_	p__Proteol	c__Gamma	o__Legione	f__Legione	g__Legione	s__uncultu	OTU1583

d__Bacteri	k__norank_	p__Firmicu	c__Negativ	o__Selenor	f__Veillone	g__Selenor	s__unclassi	OTU695
d__Bacteri	k__norank_	p__Halana	c__Halanae	o__Halana	f__Halobac	g__norank_	s__uncultu	OTU1120
d__Bacteri	k__norank_	p__Acidoba	c__Acidoba	o__Solibac	f__Solibact	g__Paludib	s__uncultu	OTU1567
d__Bacteri	k__norank_	p__Cyanob	c__Oxypho	o__Chlorop	f__norank_	g__norank_	s__unclassi	OTU897
d__Bacteri	k__norank_	p__Proteob	c__Deltapr	o__Syntrop	f__Syntrop	g__Desulfo	s__unclassi	OTU169
d__Bacteri	k__norank_	p__Deinoc	c__Deinocc	o__Therma	f__Therma	g__Thermu	s__Thermu	OTU803
d__Bacteri	k__norank_	p__Proteob	c__Alphapr	o__Holosp	f__Holospo	g__norank_	s__uncultu	OTU2090
d__Bacteri	k__norank_	p__Chlorof	c__KD4-96	o__norank_	f__norank_	g__norank_	s__unclassi	OTU2290
d__Bacteri	k__norank_	p__unclass	c__unclassi	o__unclass	f__unclassi	g__unclass	s__unclassi	OTU2008
d__Bacteri	k__norank_	p__Proteob	c__Alphapr	o__Caulob	f__Cauloba	g__Asticca	s__uncultu	OTU280
d__Bacteri	k__norank_	p__Firmicu	c__Clostrid	o__Clostrid	f__Family_	g__norank_	s__uncultu	OTU1574
d__Bacteri	k__norank_	p__Proteob	c__Gamma	o__Betapr	f__Burkhol	g__Lautrop	s__uncultu	OTU783
d__Bacteri	k__norank_	p__Proteob	c__Gamma	o__Betapr	f__Burkhol	g__Malikia	s__uncultu	OTU416
d__Bacteri	k__norank_	p__Bacter	c__Bactero	o__Flavoba	f__Flavoba	g__Flavoba	s__unclassi	OTU1461
d__Bacteri	k__norank_	p__Firmicu	c__Clostrid	o__Clostrid	f__Syntrop	g__norank_	s__unclassi	OTU1151
d__Bacteri	k__norank_	p__Firmicu	c__Clostrid	o__Clostrid	f__Lachnos	g__Lachno	s__Clostrid	OTU51
d__Bacteri	k__norank_	p__Proteob	c__Deltapr	o__Myxoc	f__Phaselic	g__Phaseli	s__unclassi	OTU2017
d__Bacteri	k__norank_	p__Proteob	c__Gamma	o__Oceanc	f__Sacchar	g__unclass	s__unclassi	OTU2765
d__Bacteri	k__norank_	p__Proteob	c__Deltapr	o__Sva048	f__norank_	g__norank_	s__uncultu	OTU2563
d__Bacteri	k__norank_	p__Proteob	c__Alphapr	o__Rhizobi	f__Devosia	g__Devosia	s__metage	OTU195
d__Bacteri	k__norank_	p__Firmicu	c__Clostrid	o__Clostrid	f__Lachnos	g__Anaero	s__Anaero	OTU2039
d__Bacteri	k__norank_	p__Proteob	c__Gamma	o__Betapr	f__Burkhol	g__Hydrog	s__uncultu	OTU3196
d__Bacteri	k__norank_	p__Proteob	c__Alphapr	o__Paraca	f__Paracae	g__Candida	s__uncultu	OTU2242
d__Bacteri	k__norank_	p__Bacter	c__Bactero	o__Bacter	f__Rikenell	g__Rikenel	s__uncultu	OTU2838
d__Bacteri	k__norank_	p__Proteob	c__Gamma	o__Betapr	f__Burkhol	g__Comam	s__Comam	OTU717
d__Bacteri	k__norank_	p__Deinoc	c__Deinocc	o__Deinoc	f__Deinocc	g__Deinoc	s__Deinocc	OTU1368
d__Bacteri	k__norank_	p__Acidoba	c__Subgro	o__norank_	f__norank_	g__norank_	s__unclassi	OTU3507
d__Bacteri	k__norank_	p__Proteob	c__Gamma	o__Diploric	f__Diploric	g__Aquicel	s__unclassi	OTU2592
d__Bacteri	k__norank_	p__Depend	c__Babelia	o__Babelia	f__Vermip	g__norank_	s__uncultu	OTU1413
d__Bacteri	k__norank_	p__Deinoc	c__Deinocc	o__Deinoc	f__Deinocc	g__Deinoc	s__unclassi	OTU1506
d__Bacteri	k__norank_	p__Proteob	c__Gamma	o__R7C24	f__norank_	g__norank_	s__uncultu	OTU1527
d__Bacteri	k__norank_	p__Depend	c__Babelia	o__Babelia	f__Vermip	g__norank_	s__unclassi	OTU1809
d__Bacteri	k__norank_	p__Firmicu	c__Clostrid	o__Clostrid	f__Rumino	g__GCA-90	s__unclassi	OTU1264
d__Bacteri	k__norank_	p__Proteob	c__Alphapr	o__Rhodob	f__Rhodob	g__Paraco	s__uncultu	OTU7
d__Bacteri	k__norank_	p__Chlamy	c__Chlamy	o__Chlamy	f__Parachl	g__Candida	s__metage	OTU2050
d__Bacteri	k__norank_	p__Proteob	c__Alphapr	o__Rhodos	f__Magnet	g__Magnet	s__uncultu	OTU2053
d__Bacteri	k__norank_	p__Firmicu	c__Clostrid	o__Clostrid	f__Peptocc	g__Thermi	s__uncultu	OTU1013
d__Bacteri	k__norank_	p__Proteob	c__Gamma	o__Xantho	f__Xanthor	g__Lysobac	s__unclassi	OTU2761
d__Bacteri	k__norank_	p__Proteob	c__Gamma	o__Legione	f__Legione	g__Legione	s__uncultu	OTU2760
d__Bacteri	k__norank_	p__Proteob	c__Gamma	o__JTB23	f__norank_	g__norank_	s__metage	OTU3626
d__Bacteri	k__norank_	p__Chlamy	c__Chlamy	o__Chlamy	f__Parachl	g__unclass	s__unclassi	OTU2147
d__Bacteri	k__norank_	p__Acidoba	c__Acidoba	o__Acidoba	f__norank_	g__norank_	s__uncultu	OTU2917
d__Bacteri	k__norank_	p__Proteob	c__Gamma	o__Betapr	f__Nitroso	g__Ellin606	s__uncultu	OTU887
d__Bacteri	k__norank_	p__Chlamy	c__Chlamy	o__Chlamy	f__Parachl	g__unclass	s__unclassi	OTU2148
d__Bacteri	k__norank_	p__Proteob	c__Gamma	o__Pseudo	f__Pseudor	g__Pseudo	s__unclassi	OTU1943
d__Bacteri	k__norank_	p__Chlorof	c__Ktedon	o__C0119	f__norank_	g__norank_	s__uncultu	OTU1894
d__Bacteri	k__norank_	p__Chlorof	c__Anaerol	o__Anaero	f__Anaerol	g__norank_	s__uncultu	OTU2534

d__Bacteri	k__norank_	p__Bacterc	c__Bactero	o__Bacterc	f__Tannere	g__Parabac	s__Parabac	OTU2773
d__Bacteri	k__norank_	p__Proteob	c__Deltapr	o__PB19	f__norank_	g__norank_	s__metage	OTU423
d__Bacteri	k__norank_	p__Verrucc	c__Verrucc	o__Chthon	f__Chthoni	g__LD29	s__uncultu	OTU1945
d__Bacteri	k__norank_	p__Proteob	c__Gamma	o__Diploric	f__Diploric	g__norank_	s__unclassi	OTU1786
d__Bacteri	k__norank_	p__Proteob	c__Gamma	o__Diploric	f__Diploric	g__Aquicel	s__uncultu	OTU2810
d__Bacteri	k__norank_	p__Cyanob	c__Melaina	o__Gastrar	f__norank_	g__norank_	s__uncultu	OTU1505
d__Bacteri	k__norank_	p__Firmicu	c__Erysipel	o__Erysipe	f__Erysipel	g__norank_	s__unclassi	OTU2629
d__Bacteri	k__norank_	p__Depenc	c__Babelia	o__Babelia	f__unclassi	g__unclass	s__unclassi	OTU2670
d__Bacteri	k__norank_	p__Actinob	c__Actinob	o__Propior	f__Propion	g__norank_	s__metage	OTU1168
d__Bacteri	k__norank_	p__Proteob	c__Gamma	o__Betaprc	f__Burkhol	g__Polynuc	s__Polynuc	OTU3147
d__Bacteri	k__norank_	p__Proteob	c__Alphapr	o__Paracae	f__Paracae	g__Candida	s__uncultu	OTU3200
d__Bacteri	k__norank_	p__Proteob	c__Deltapr	o__Syntrop	f__Syntrop	g__Smithel	s__uncultu	OTU443
d__Bacteri	k__norank_	p__Proteob	c__Gamma	o__Legione	f__Legione	g__Legione	s__uncultu	OTU2341
d__Bacteri	k__norank_	p__Firmicu	c__Clostrid	o__Clostrid	f__Christer	g__Christer	s__unclassi	OTU1394
d__Bacteri	k__norank_	p__Actinob	c__Actinob	o__Microtr	f__Ilumato	g__CL500-2	s__uncultu	OTU1995
d__Bacteri	k__norank_	p__Proteob	c__Alphapr	o__Azospir	f__Azospiri	g__norank_	s__metage	OTU1118
d__Bacteri	k__norank_	p__Proteob	c__Deltapr	o__Desulfu	f__Geobac	g__Geothe	s__unclassi	OTU2469
d__Bacteri	k__norank_	p__Patescil	c__Microge	o__Candida	f__norank_	g__norank_	s__uncultu	OTU1715
d__Bacteri	k__norank_	p__Bacterc	c__Bactero	o__Cytoph	f__Spirosor	g__Lacihab	s__uncultu	OTU2505
d__Bacteri	k__norank_	p__Acidoba	c__Subgrou	o__norank_	f__norank_	g__norank_	s__unclassi	OTU3703
d__Bacteri	k__norank_	p__Elusimi	c__Elusimi	o__Lineage	f__norank_	g__norank_	s__uncultu	OTU1061
d__Bacteri	k__norank_	p__Depenc	c__Babelia	o__Babelia	f__UBA124	g__norank_	s__uncultu	OTU2367
d__Bacteri	k__norank_	p__Proteob	c__Alphapr	o__unclass	f__unclassi	g__unclass	s__unclassi	OTU1950
d__Bacteri	k__norank_	p__Proteob	c__Alphapr	o__unclass	f__unclassi	g__unclass	s__unclassi	OTU1951
d__Bacteri	k__norank_	p__Bacterc	c__Bactero	o__Bacterc	f__Bactero	g__Bacterc	s__unclassi	OTU1080
d__Bacteri	k__norank_	p__Depenc	c__Babelia	o__Babelia	f__unclassi	g__unclass	s__unclassi	OTU1835
d__Bacteri	k__norank_	p__Proteob	c__Gamma	o__Coxiella	f__Coxiella	g__Coxiella	s__uncultu	OTU2795
d__Bacteri	k__norank_	p__Proteob	c__Gamma	o__Methyl	f__Methylc	g__unclass	s__unclassi	OTU2324
d__Bacteri	k__norank_	p__Firmicu	c__Clostrid	o__Clostrid	f__Peptost	g__Filifactc	s__unclassi	OTU1977
d__Bacteri	k__norank_	p__Proteob	c__Gamma	o__Compe	f__Compet	g__Candida	s__uncultu	OTU1218
d__Bacteri	k__norank_	p__Proteob	c__Gamma	o__norank_	f__norank_	g__norank_	s__unclassi	OTU1738
d__Bacteri	k__norank_	p__Bacterc	c__Bactero	o__Flavoba	f__Weekse	g__Chrysec	s__Chrysec	OTU1123
d__Bacteri	k__norank_	p__Proteob	c__Gamma	o__Diploric	f__Diploric	g__Aquicel	s__uncultu	OTU2049
d__Bacteri	k__norank_	p__Proteob	c__Gamma	o__Coxiella	f__Coxiella	g__Coxiella	s__uncultu	OTU2405
d__Bacteri	k__norank_	p__Depenc	c__Babelia	o__Babelia	f__Vermipl	g__norank_	s__uncultu	OTU2335
d__Bacteri	k__norank_	p__Bacterc	c__Bactero	o__Flavoba	f__Weekse	g__Chrysec	s__unclassi	OTU2259
d__Bacteri	k__norank_	p__Deinoc	c__Deinocc	o__Deinoc	f__Deinocc	g__Deinoc	s__Deinocc	OTU1380
d__Bacteri	k__norank_	p__Depenc	c__Babelia	o__Babelia	f__UBA124	g__norank_	s__uncultu	OTU2564
d__Bacteri	k__norank_	p__Proteob	c__Gamma	o__Legione	f__Legione	g__Legione	s__unclassi	OTU2757
d__Bacteri	k__norank_	p__Proteob	c__Gamma	o__Cellvibr	f__Halieace	g__OM60N	s__uncultu	OTU2814
d__Bacteri	k__norank_	p__Proteob	c__Gamma	o__Xantho	f__Xanthor	g__Lysobac	s__Lysobac	OTU666
d__Bacteri	k__norank_	p__Actinob	c__Actinob	o__Propior	f__Nocardi	g__Nocardi	s__unclassi	OTU1321
d__Bacteri	k__norank_	p__Proteob	c__Gamma	o__Diploric	f__Diploric	g__norank_	s__unclassi	OTU2132
d__Bacteri	k__norank_	p__Firmicu	c__Clostrid	o__Clostrid	f__Rumino	g__Ruminc	s__uncultu	OTU1089
d__Bacteri	k__norank_	p__Proteob	c__Alphapr	o__Reyran	f__Reyrane	g__norank_	s__unclassi	OTU848
d__Bacteri	k__norank_	p__Proteob	c__Gamma	o__Diploric	f__Diploric	g__Aquicel	s__uncultu	OTU2680
d__Bacteri	k__norank_	p__Proteob	c__Alphapr	o__Rhodob	f__Rhodob	g__Loktane	s__Loktane	OTU4022

d__Bacteri	k__norank_	p__Proteot	c__Alphapr	o__Rhodob	f__Rhodob	g__Gemmc	s__unclassi	OTU857
d__Bacteri	k__norank_	p__Firmicu	c__Bacilli	o__Bacillal	f__Bacillac	g__Bacillus	s__unclassi	OTU1000
d__Bacteri	k__norank_	p__Gemma	c__Gemma	o__Gemma	f__Gemma	g__Gemma	s__unclassi	OTU1350
d__Bacteri	k__norank_	p__Cyanob	c__Oxypho	o__Chlorop	f__norank_	g__norank_	s__unclassi	OTU2054
d__Bacteri	k__norank_	p__Chlamy	c__Chlamy	o__Chlamy	f__Simkani	g__norank_	s__unclassi	OTU1895
d__Bacteri	k__norank_	p__Firmicu	c__Bacilli	o__Bacillal	f__Bacillac	g__Anoxyb	s__unclassi	OTU43
d__Bacteri	k__norank_	p__Chlorof	c__JG30-KF	o__norank_	f__norank_	g__norank_	s__unclassi	OTU1648
d__Bacteri	k__norank_	p__Bacterc	c__Bactero	o__Bacterc	f__unclassi	g__unclass	s__unclassi	OTU2059
d__Bacteri	k__norank_	p__Chlorof	c__Anaerol	o__Anaero	f__Anaerol	g__Anaerol	s__unclassi	OTU2330
d__Bacteri	k__norank_	p__Proteot	c__Gamma	o__Betaprc	f__Burkhol	g__GKS98_	s__unclassi	OTU454
d__Bacteri	k__norank_	p__Epsilon	c__Campyl	o__Campyl	f__Campyl	g__Campyl	s__Campyl	OTU2156
d__Bacteri	k__norank_	p__Actinot	c__Actinob	o__Microtr	f__norank_	g__norank_	s__uncultu	OTU1389
d__Bacteri	k__norank_	p__Acidoba	c__Holoph	o__Holoph	f__Holopha	g__marine_	s__uncultu	OTU2481
d__Bacteri	k__norank_	p__Firmicu	c__Clostrid	o__Clostrid	f__Lachnos	g__norank_	s__unclassi	OTU44
d__Bacteri	k__norank_	p__Chlamy	c__Chlamy	o__Chlamy	f__Parachla	g__unclass	s__unclassi	OTU2779
d__Bacteri	k__norank_	p__Actinot	c__Actinob	o__Solirubi	f__Solirubr	g__Solirubi	s__unclassi	OTU1092
d__Bacteri	k__norank_	p__Actinot	c__Actinob	o__Frankia	f__Sporicht	g__hgcI_cla	s__uncultu	OTU982
d__Bacteri	k__norank_	p__Proteot	c__Alphapr	o__Rhodob	f__Rhodob	g__HIMB11	s__uncultu	OTU2024
d__Bacteri	k__norank_	p__Proteot	c__Gamma	o__Legione	f__Legione	g__Legione	s__unclassi	OTU1921
d__Bacteri	k__norank_	p__Bacterc	c__Bactero	o__Chitino	f__Saprosp	g__Candida	s__uncultu	OTU972
d__Bacteri	k__norank_	p__Proteot	c__Gamma	o__Betaprc	f__Burkhol	g__unclass	s__unclassi	OTU1510
d__Bacteri	k__norank_	p__Depenc	c__Babelia	o__Babelia	f__Vermipl	g__norank_	s__unclassi	OTU2421
d__Bacteri	k__norank_	p__Depenc	c__Babelia	o__Babelia	f__Babeliac	g__norank_	s__uncultu	OTU2523
d__Bacteri	k__norank_	p__Cyanob	c__Oxypho	o__Synech	f__Cyanobi	g__Cyanob	s__uncultu	OTU1654
d__Bacteri	k__norank_	p__Proteot	c__Gamma	o__Diploric	f__Diploric	g__Aquicel	s__uncultu	OTU2313
d__Bacteri	k__norank_	p__Proteot	c__Alphapr	o__Sphingc	f__Sphingo	g__Sphingc	s__unclassi	OTU380
d__Bacteri	k__norank_	p__Nitrosp	c__Nitrosp	o__norank_	f__norank_	g__Nitrosp	s__uncultu	OTU3857
d__Bacteri	k__norank_	p__Proteot	c__Gamma	o__Betaprc	f__Burkhol	g__CM1G0	s__uncultu	OTU2182
d__Bacteri	k__norank_	p__Firmicu	c__Bacilli	o__Lactoba	f__Enteroc	g__Enteroc	s__unclassi	OTU2410
d__Bacteri	k__norank_	p__Proteot	c__Gamma	o__unclass	f__unclassi	g__unclass	s__unclassi	OTU873
d__Bacteri	k__norank_	p__Proteot	c__Alphapr	o__Sphingc	f__Sphingo	g__Sphingc	s__Sphingc	OTU1349
d__Bacteri	k__norank_	p__Proteot	c__Alphapr	o__Acetob	f__Acetoba	g__Acidoce	s__unclassi	OTU1578
d__Bacteri	k__norank_	p__Firmicu	c__Bacilli	o__Lactoba	f__Aerococ	g__Aerococ	s__Aerococ	OTU1331
d__Bacteri	k__norank_	p__Proteot	c__Gamma	o__Betaprc	f__Methylc	g__Candida	s__unclassi	OTU2771
d__Bacteri	k__norank_	p__Firmicu	c__Clostrid	o__Clostrid	f__Lachnos	g__Tyzzere	s__unclassi	OTU1186
d__Bacteri	k__norank_	p__Depenc	c__Babelia	o__Babelia	f__norank_	g__norank_	s__uncultu	OTU2190
d__Bacteri	k__norank_	p__Proteot	c__Deltapr	o__Oligofle	f__Oligofle	g__norank_	s__unclassi	OTU2249
d__Bacteri	k__norank_	p__Proteot	c__Gamma	o__Pseudo	f__Pseudor	g__Pseudo	s__unclassi	OTU1409
d__Bacteri	k__norank_	p__Proteot	c__Gamma	o__Diploric	f__Diploric	g__Aquicel	s__uncultu	OTU2267
d__Bacteri	k__norank_	p__Proteot	c__Gamma	o__Diploric	f__Diploric	g__Aquicel	s__uncultu	OTU2266
d__Bacteri	k__norank_	p__Firmicu	c__Bacilli	o__Bacillal	f__Planoco	g__Paenisp	s__uncultu	OTU1221
d__Bacteri	k__norank_	p__unclass	c__unclassi	o__unclass	f__unclassi	g__unclass	s__unclassi	OTU1342
d__Bacteri	k__norank_	p__Armatir	c__Fimbriir	o__Fimbriir	f__Fimbriir	g__norank_	s__unclassi	OTU1340
d__Bacteri	k__norank_	p__Chlamy	c__Chlamy	o__Chlamy	f__unclassi	g__unclass	s__unclassi	OTU1688
d__Bacteri	k__norank_	p__Cyanob	c__Oxypho	o__unclass	f__unclassi	g__unclass	s__unclassi	OTU2854
d__Bacteri	k__norank_	p__Depenc	c__Babelia	o__Babelia	f__Babeliac	g__norank_	s__uncultu	OTU2708
d__Bacteri	k__norank_	p__Proteot	c__Gamma	o__Vibrion	f__Vibriona	g__Vibrio	s__unclassi	OTU2452

d__Bacteri	k__norank_	p__Proteol	c__Alphapr	o__Holosp	f__Holospo	g__norank_	s__unclassi	OTU1637
d__Bacteri	k__norank_	p__Bacterc	c__Bactero	o__Bacterc	f__Muribac	g__norank_	s__uncultu	OTU2504
d__Bacteri	k__norank_	p__Proteol	c__Gamma	o__Compe	f__Compet	g__Candida	s__uncultu	OTU2451
d__Bacteri	k__norank_	p__Chlorof	c__Anaerol	o__SBR103	f__A4b	g__norank_	s__unclassi	OTU1014
d__Bacteri	k__norank_	p__Acidoba	c__Subgrou	o__norank_	f__norank_	g__norank_	s__unclassi	OTU2479
d__Bacteri	k__norank_	p__Depenc	c__Babelia	o__Babelia	f__Vermipl	g__norank_	s__uncultu	OTU2203
d__Bacteri	k__norank_	p__Actinob	c__Actinob	o__Actinor	f__Actinom	g__Actinon	s__Actinom	OTU801
d__Bacteri	k__norank_	p__Proteol	c__Gamma	o__Betapro	f__Neisseri	g__norank_	s__unclassi	OTU2001
d__Bacteri	k__norank_	p__Proteol	c__Alphapr	o__Rhizobi	f__Beijerin	g__Methylo	s__Methylo	OTU17
d__Bacteri	k__norank_	p__Firmicu	c__Bacilli	o__Bacillal	f__Paeniba	g__Paeniba	s__Paeniba	OTU822
d__Bacteri	k__norank_	p__Proteol	c__Gamma	o__Diploric	f__Diploric	g__norank_	s__unclassi	OTU2142
d__Bacteri	k__norank_	p__Gemma	c__Gemma	o__Gemma	f__Gemma	g__norank_	s__uncultu	OTU2424
d__Bacteri	k__norank_	p__Proteol	c__Alphapr	o__Cauloba	f__Cauloba	g__Brevund	s__Brevund	OTU851
d__Bacteri	k__norank_	p__Proteol	c__Gamma	o__Betapro	f__Chitinib	g__Deefgea	s__Deefgea	OTU1198
d__Bacteri	k__norank_	p__Firmicu	c__Bacilli	o__Bacillal	f__Alicyclol	g__Alicyclo	s__Alicyclo	OTU1008
d__Bacteri	k__norank_	p__Bacterc	c__Bactero	o__Flavoba	f__Flavoba	g__Flavoba	s__Flavoba	OTU1140
d__Bacteri	k__norank_	p__Depenc	c__Babelia	o__Babelia	f__unclassi	g__unclass	s__unclassi	OTU2118
d__Bacteri	k__norank_	p__Actinob	c__Actinob	o__Actinor	f__norank_	g__norank_	s__unclassi	OTU1991
d__Bacteri	k__norank_	p__Proteol	c__Gamma	o__Xantho	f__Xanthor	g__Lysobac	s__unclassi	OTU2584
d__Bacteri	k__norank_	p__Firmicu	c__Clostrid	o__Clostrid	f__Rumino	g__Sacchar	s__unclassi	OTU1659
d__Bacteri	k__norank_	p__Proteol	c__Deltapr	o__Myxocc	f__Blfdi19	g__norank_	s__uncultu	OTU965
d__Bacteri	k__norank_	p__Firmicu	c__Negativ	o__Selenor	f__Veillone	g__norank_	s__low_GC	OTU106
d__Bacteri	k__norank_	p__Depenc	c__Babelia	o__Babelia	f__Vermipl	g__norank_	s__uncultu	OTU1716
d__Bacteri	k__norank_	p__Proteol	c__Deltapr	o__Myxocc	f__Blfdi19	g__norank_	s__uncultu	OTU2587
d__Bacteri	k__norank_	p__Firmicu	c__Bacilli	o__Bacillal	f__Bacillac	g__Bacillus	s__Bacillus	OTU1508
d__Bacteri	k__norank_	p__Firmicu	c__Clostrid	o__Clostrid	f__Gracilib	g__Lutispo	s__uncultu	OTU1205
d__Bacteri	k__norank_	p__Proteol	c__Deltapr	o__Desulfo	f__Desulfo	g__Desulfo	s__unclassi	OTU2138
d__Bacteri	k__norank_	p__Fusoba	c__Fusobac	o__Fusoba	f__Leptotri	g__Leptotr	s__unclassi	OTU1117
d__Bacteri	k__norank_	p__Bacterc	c__Bactero	o__Chitino	f__Chitinop	g__norank_	s__unclassi	OTU1357
d__Bacteri	k__norank_	p__Armatir	c__norank_	o__norank_	f__norank_	g__norank_	s__uncultu	OTU1937
d__Bacteri	k__norank_	p__Acidoba	c__Holophi	o__Subgro	f__norank_	g__norank_	s__uncultu	OTU1216
d__Bacteri	k__norank_	p__Depenc	c__Babelia	o__Babelia	f__UBA124	g__norank_	s__uncultu	OTU2632
d__Bacteri	k__norank_	p__Proteol	c__Deltapr	o__unclass	f__unclassi	g__unclass	s__unclassi	OTU2646
d__Bacteri	k__norank_	p__Bacterc	c__Bactero	o__Flavoba	f__Flavoba	g__Flavoba	s__unclassi	OTU1347
d__Bacteri	k__norank_	p__Proteol	c__Alphapr	o__Rhizobi	f__Hyphom	g__unclass	s__unclassi	OTU2878
d__Bacteri	k__norank_	p__Nitrosp	c__Nitrospi	o__norank_	f__norank_	g__Nitrosp	s__uncultu	OTU1752
d__Bacteri	k__norank_	p__Proteol	c__Gamma	o__Diploric	f__Diploric	g__norank_	s__unclassi	OTU1794
d__Bacteri	k__norank_	p__Depenc	c__Babelia	o__Babelia	f__UBA124	g__norank_	s__uncultu	OTU2552
d__Bacteri	k__norank_	p__Depenc	c__Babelia	o__Babelia	f__Vermipl	g__norank_	s__unclassi	OTU2634
d__Bacteri	k__norank_	p__Firmicu	c__Clostrid	o__Clostrid	f__Lachnos	g__Shuttle	s__metage	OTU1628
d__Bacteri	k__norank_	p__Actinob	c__Actinob	o__Propior	f__Nocardi	g__Nocardi	s__unclassi	OTU1451
d__Bacteri	k__norank_	p__Bacterc	c__Bactero	o__Bacterc	f__norank_	g__norank_	s__metage	OTU1702
d__Bacteri	k__norank_	p__Proteol	c__Alphapr	o__Rhizobi	f__Hyphom	g__Pedomi	s__uncultu	OTU176
d__Bacteri	k__norank_	p__Bacterc	c__Bactero	o__Cytoph	f__unclassi	g__unclass	s__unclassi	OTU2010
d__Bacteri	k__norank_	p__Proteol	c__Gamma	o__Alteron	f__Alterom	g__Rheinhe	s__Rheinhe	OTU833
d__Bacteri	k__norank_	p__Nitrosp	c__Thermo	o__norank_	f__norank_	g__norank_	s__uncultu	OTU2126
d__Bacteri	k__norank_	p__Chlamy	c__Chlamy	o__Chlamy	f__Parachla	g__unclass	s__unclassi	OTU1770

d__Bacteri	k__norank_	p__Proteob	c__Gamma	o__Diploric	f__Diploric	g__Aquicel	s__unclassi	OTU2776
d__Bacteri	k__norank_	p__Proteob	c__Gamma	o__Betapro	f__Neisseri	g__norank_	s__metage	OTU2530
d__Bacteri	k__norank_	p__Chlorof	c__Chlorof	o__Thermo	f__JG30-KF	g__norank_	s__metage	OTU1058
d__Bacteri	k__norank_	p__Patescil	c__Sacchar	o__Sacchar	f__norank_	g__norank_	s__unclassi	OTU2733
d__Bacteri	k__norank_	p__Bactero	c__Bactero	o__Flavoba	f__Flavoba	g__Flavoba	s__unclassi	OTU2319
d__Bacteri	k__norank_	p__Firmicu	c__Clostrid	o__Clostrid	f__Clostridi	g__Clostrid	s__unclassi	OTU537
d__Bacteri	k__norank_	p__Margul	c__norank_	o__norank_	f__norank_	g__norank_	s__unclassi	OTU2097
d__Bacteri	k__norank_	p__Bactero	c__Bactero	o__Bactero	f__Prevote	g__unclass	s__unclassi	OTU2072
d__Bacteri	k__norank_	p__Proteob	c__Gamma	o__Compet	f__Compet	g__Candida	s__unclassi	OTU167
d__Bacteri	k__norank_	p__Proteob	c__Gamma	o__Legione	f__Legione	g__Legione	s__uncultu	OTU2921
d__Bacteri	k__norank_	p__Acidoba	c__Subgrou	o__norank_	f__norank_	g__norank_	s__metage	OTU1229
d__Bacteri	k__norank_	p__Firmicu	c__Negativ	o__Selenor	f__Veillone	g__Selenor	s__uncultu	OTU1401
d__Bacteri	k__norank_	p__Proteob	c__Gamma	o__Diploric	f__Diploric	g__Aquicel	s__metage	OTU1744
d__Bacteri	k__norank_	p__Proteob	c__Gamma	o__Cellvibr	f__Porticoc	g__C1-B04	s__uncultu	OTU2780
d__Bacteri	k__norank_	p__Proteob	c__Gamma	o__Betapro	f__Burkhol	g__Cupriav	s__Cupriav	OTU967
d__Bacteri	k__norank_	p__Actinob	c__Actinob	o__Propior	f__Nocardi	g__Nocardi	s__unclassi	OTU1074
d__Bacteri	k__norank_	p__Chlamy	c__Chlamy	o__Chlamy	f__unclassi	g__unclass	s__unclassi	OTU2580
d__Bacteri	k__norank_	p__Firmicu	c__Bacilli	o__Bacillal	f__Paeniba	g__Cohnell	s__unclassi	OTU1509
d__Bacteri	k__norank_	p__Chlamy	c__Chlamy	o__Chlamy	f__Parachla	g__unclass	s__unclassi	OTU2396
d__Bacteri	k__norank_	p__Depenc	c__Babelia	o__Babelia	f__UBA124	g__norank_	s__unclassi	OTU1726
d__Bacteri	k__norank_	p__Firmicu	c__Clostrid	o__Clostrid	f__Rumino	g__Ruminic	s__unclassi	OTU1573
d__Bacteri	k__norank_	p__Proteob	c__Gamma	o__Betapro	f__Neisseri	g__norank_	s__uncultu	OTU2806
d__Bacteri	k__norank_	p__Firmicu	c__Erysipel	o__Erysipe	f__Erysipel	g__Turiciba	s__uncultu	OTU752
d__Bacteri	k__norank_	p__Bactero	c__Bactero	o__Bactero	f__unclassi	g__unclass	s__unclassi	OTU2835
d__Bacteri	k__norank_	p__Proteob	c__Alphapr	o__Rhizobi	f__Rhizobia	g__norank_	s__metage	OTU1375
d__Bacteri	k__norank_	p__Firmicu	c__Bacilli	o__Lactoba	f__Lactoba	g__Lactoba	s__Lactoba	OTU2734
d__Bacteri	k__norank_	p__Cyanob	c__Oxypho	o__Chlorop	f__norank_	g__norank_	s__unclassi	OTU2843
d__Bacteri	k__norank_	p__Patescil	c__Sacchar	o__Sacchar	f__norank_	g__norank_	s__wastew	OTU3398
d__Bacteri	k__norank_	p__Bactero	c__Bactero	o__Bactero	f__Muribac	g__norank_	s__uncultu	OTU2449
d__Bacteri	k__norank_	p__Proteob	c__Deltapr	o__Bdellov	f__Bacteric	g__Peredib	s__uncultu	OTU1857
d__Bacteri	k__norank_	p__Proteob	c__Gamma	o__unclass	f__unclassi	g__unclass	s__unclassi	OTU2295
d__Bacteri	k__norank_	p__Proteob	c__Alphapr	o__Sphingo	f__Sphingo	g__Porphyr	s__uncultu	OTU356
d__Bacteri	k__norank_	p__Proteob	c__Gamma	o__Cellvibr	f__Cellvibri	g__Cellvibr	s__uncultu	OTU1015
d__Bacteri	k__norank_	p__Chlamy	c__Chlamy	o__Chlamy	f__Parachla	g__Neochla	s__unclassi	OTU2225
d__Bacteri	k__norank_	p__Acidoba	c__Subgrou	o__norank_	f__norank_	g__norank_	s__unclassi	OTU286
d__Bacteri	k__norank_	p__Depenc	c__Babelia	o__Babelia	f__unclassi	g__unclass	s__unclassi	OTU2846
d__Bacteri	k__norank_	p__Chlamy	c__Chlamy	o__Chlamy	f__Simkani	g__Candida	s__uncultu	OTU1672
d__Bacteri	k__norank_	p__Bactero	c__Bactero	o__Cytopha	f__Spirosor	g__Pseudar	s__uncultu	OTU3257
d__Bacteri	k__norank_	p__Proteob	c__Gamma	o__Diploric	f__Diploric	g__Aquicel	s__unclassi	OTU2837
d__Bacteri	k__norank_	p__Proteob	c__Alphapr	o__Rhizobi	f__Rhizobia	g__Mesorh	s__unclassi	OTU3169
d__Bacteri	k__norank_	p__Proteob	c__Gamma	o__Diploric	f__Diploric	g__Aquicel	s__unclassi	OTU3194
d__Bacteri	k__norank_	p__Chlamy	c__Chlamy	o__Chlamy	f__unclassi	g__unclass	s__unclassi	OTU1854
d__Bacteri	k__norank_	p__Chlamy	c__Chlamy	o__Chlamy	f__unclassi	g__unclass	s__unclassi	OTU1855
d__Bacteri	k__norank_	p__Chlorof	c__SL56_m	o__norank_	f__norank_	g__norank_	s__uncultu	OTU2833
d__Bacteri	k__norank_	p__Proteob	c__Gamma	o__Xantho	f__Xanthor	g__Pseudo	s__uncultu	OTU1208
d__Bacteri	k__norank_	p__Proteob	c__Gamma	o__Coxiella	f__Coxiella	g__Coxiella	s__uncultu	OTU2149
d__Bacteri	k__norank_	p__Firmicu	c__Clostrid	o__Clostrid	f__Clostridi	g__Clostrid	s__Clostrid	OTU766

d__Bacteri	k__norank_	p__Bacterc	c__Bactero	o__Chitino	f__Chitinop	g__Flavihu	s__unclassi	OTU1139
d__Bacteri	k__norank_	p__Proteok	c__Gamma	o__unclass	f__unclassi	g__unclass	s__unclassi	OTU2164
d__Bacteri	k__norank_	p__Proteok	c__Gamma	o__unclass	f__unclassi	g__unclass	s__unclassi	OTU2162
d__Bacteri	k__norank_	p__Cyanob	c__Oxypho	o__Chlorop	f__norank_	g__norank_	s__uncultu	OTU2171
d__Bacteri	k__norank_	p__Deinoc	c__Deinocc	o__Deinoc	f__Trueper	g__Trueper	s__unclassi	OTU2058
d__Bacteri	k__norank_	p__Depenc	c__Babelia	o__Babelia	f__Vermipl	g__norank_	s__uncultu	OTU2651
d__Bacteri	k__norank_	p__Spiroch	c__Spiroch	o__Spiroch	f__Spiroch	g__norank_	s__unclassi	OTU2427
d__Bacteri	k__norank_	p__Depenc	c__Babelia	o__Babelia	f__unclassi	g__unclass	s__unclassi	OTU2591
d__Bacteri	k__norank_	p__Proteok	c__Gamma	o__Legione	f__Legione	g__Legione	s__unclassi	OTU2749
d__Bacteri	k__norank_	p__Depenc	c__Babelia	o__Babelia	f__unclassi	g__unclass	s__unclassi	OTU2597
d__Bacteri	k__norank_	p__Proteok	c__Gamma	o__Gamma	f__unclassi	g__Candida	s__unclassi	OTU2322
d__Bacteri	k__norank_	p__Proteok	c__Gamma	o__Xantho	f__Xanthor	g__Arenim	s__unclassi	OTU3654
d__Bacteri	k__norank_	p__Proteok	c__Gamma	o__Betaprc	f__Rhodoc	g__Zoogloe	s__unclassi	OTU898
d__Bacteri	k__norank_	p__Proteok	c__Alphapr	o__Rickett	f__Ricketts	g__Candida	s__uncultu	OTU3158
d__Bacteri	k__norank_	p__Proteok	c__Gamma	o__Diploric	f__Diploric	g__norank_	s__uncultu	OTU1523
d__Bacteri	k__norank_	p__Patescil	c__Sacchar	o__Sacchar	f__Sacchar	g__norank_	s__unclassi	OTU657
d__Bacteri	k__norank_	p__unclass	c__unclassi	o__unclass	f__unclassi	g__unclass	s__unclassi	OTU1800
d__Bacteri	k__norank_	p__Proteok	c__Gamma	o__Diploric	f__Diploric	g__Aquicel	s__unclassi	OTU2455
d__Bacteri	k__norank_	p__Proteok	c__Alphapr	o__norank_	f__norank_	g__norank_	s__unclassi	OTU1563
d__Bacteri	k__norank_	p__Depenc	c__Babelia	o__Babelia	f__UBA124	g__norank_	s__uncultu	OTU2426
d__Bacteri	k__norank_	p__Bacterc	c__Bactero	o__Chitino	f__Saprosp	g__norank_	s__uncultu	OTU1149
d__Bacteri	k__norank_	p__Proteok	c__Gamma	o__Gamma	f__unclassi	g__Candida	s__uncultu	OTU2600
d__Bacteri	k__norank_	p__Firmicu	c__Clostrid	o__Clostrid	f__Rumino	g__Fournie	s__uncultu	OTU2951
d__Bacteri	k__norank_	p__Depenc	c__Babelia	o__Babelia	f__UBA124	g__norank_	s__uncultu	OTU2438
d__Bacteri	k__norank_	p__Plancto	c__Phycisp	o__Phycisp	f__Phycispl	g__CL500-3	s__uncultu	OTU1899
d__Bacteri	k__norank_	p__Actinok	c__Actinob	o__Actinor	f__norank_	g__norank_	s__unclassi	OTU1686
d__Bacteri	k__norank_	p__Proteok	c__Gamma	o__Betaprc	f__unclassi	g__unclass	s__unclassi	OTU2555
d__Bacteri	k__norank_	p__Bacterc	c__Bactero	o__Chitino	f__Chitinop	g__Sedimir	s__unclassi	OTU296
d__Bacteri	k__norank_	p__Chlorof	c__Chlorof	o__Thermc	f__JG30-KF	g__norank_	s__uncultu	OTU1602
d__Bacteri	k__norank_	p__Proteok	c__Gamma	o__Betaprc	f__Burkhol	g__Pauciba	s__unclassi	OTU268
d__Bacteri	k__norank_	p__Teneric	c__Mollicu	o__Halopla	f__Halopla	g__Halopla	s__unclassi	OTU1190
d__Bacteri	k__norank_	p__Firmicu	c__Clostrid	o__Clostrid	f__Rumino	g__Ruminic	s__unclassi	OTU2845
d__Bacteri	k__norank_	p__Depenc	c__Babelia	o__Babelia	f__Vermipl	g__norank_	s__unclassi	OTU2707
d__Bacteri	k__norank_	p__Proteok	c__Alphapr	o__Rhodov	f__Fodinicu	g__norank_	s__metage	OTU1657
d__Bacteri	k__norank_	p__Proteok	c__Gamma	o__Betaprc	f__Burkhol	g__Bordete	s__Bordete	OTU2890
d__Bacteri	k__norank_	p__Firmicu	c__Bacilli	o__Lactoba	f__Leucon	g__Weissel	s__Weissel	OTU736
d__Bacteri	k__norank_	p__Depenc	c__Babelia	o__Babelia	f__Vermipl	g__norank_	s__unclassi	OTU2419
d__Bacteri	k__norank_	p__Proteok	c__Gamma	o__Diploric	f__Diploric	g__Aquicel	s__unclassi	OTU1836
d__Bacteri	k__norank_	p__Chlorof	c__Dehaloc	o__S085	f__norank_	g__norank_	s__unclassi	OTU1745
d__Bacteri	k__norank_	p__Proteok	c__Alphapr	o__Rhizobi	f__Beijerin	g__Methyl	s__Methyl	OTU122
d__Bacteri	k__norank_	p__Proteok	c__Gamma	o__Betaprc	f__Gallione	g__Gallione	s__unclassi	OTU924
d__Bacteri	k__norank_	p__Proteok	c__Deltapr	o__Bdellov	f__Bdellovi	g__OM27_	s__uncultu	OTU3417
d__Bacteri	k__norank_	p__Bacterc	c__Bactero	o__Bacterc	f__Rikenell	g__Alistipe	s__uncultu	OTU2635
d__Bacteri	k__norank_	p__Proteok	c__Deltapr	o__Syntrop	f__Syntrop	g__Syntrop	s__unclassi	OTU2688
d__Bacteri	k__norank_	p__GAL15	c__norank_	o__norank_	f__norank_	g__norank_	s__uncultu	OTU2337
d__Bacteri	k__norank_	p__GAL15	c__norank_	o__norank_	f__norank_	g__norank_	s__uncultu	OTU2339
d__Bacteri	k__norank_	p__Proteok	c__Alphapr	o__Rhizobi	f__Rhizobia	g__Allorhiz	s__Pararhiz	OTU200

d__Bacteri	k__norank_	p__Bactero	c__Bactero	o__Chitino	f__Saprosp	g__norank_	s__metage	OTU1077
d__Bacteri	k__norank_	p__Proteob	c__Alphapr	o__Rickett	f__SM2D12	g__norank_	s__unident	OTU3
d__Bacteri	k__norank_	p__Actinob	c__Actinob	o__norank_	f__norank_	g__norank_	s__uncultu	OTU1607
d__Bacteri	k__norank_	p__Verruco	c__Verruco	o__Opituta	f__Opituta	g__Opitutu	s__unclassi	OTU3634
d__Bacteri	k__norank_	p__Proteob	c__Gamma	o__Betapro	f__Burkhol	g__unclass	s__unclassi	OTU3985
d__Bacteri	k__norank_	p__Proteob	c__Gamma	o__Diploric	f__Diploric	g__norank_	s__unclassi	OTU2467
d__Bacteri	k__norank_	p__Bactero	c__Bactero	o__Bactero	f__Muribac	g__norank_	s__uncultu	OTU2071
d__Bacteri	k__norank_	p__Proteob	c__Deltapr	o__Bdellov	f__Bdellovi	g__OM27_	s__uncultu	OTU1779
d__Bacteri	k__norank_	p__Depend	c__Babelia	o__Babelia	f__Vermiph	g__norank_	s__uncultu	OTU2528
d__Bacteri	k__norank_	p__Proteob	c__Gamma	o__Betapro	f__Nitroso	g__MND1	s__unclassi	OTU2492
d__Bacteri	k__norank_	p__Depend	c__Babelia	o__Babelia	f__Vermiph	g__norank_	s__uncultu	OTU2524
d__Bacteri	k__norank_	p__Proteob	c__Deltapr	o__Bdellov	f__Bdellovi	g__Bdellov	s__uncultu	OTU3301
d__Bacteri	k__norank_	p__Bactero	c__Bactero	o__Cytoph	f__Microsc	g__unclass	s__unclassi	OTU3352
d__Bacteri	k__norank_	p__Chlamy	c__Chlamy	o__Chlamy	f__cvE6	g__norank_	s__unclassi	OTU2329
d__Bacteri	k__norank_	p__Proteob	c__Gamma	o__KF-JG30	f__norank_	g__norank_	s__uncultu	OTU2599
d__Bacteri	k__norank_	p__Actinob	c__Actinob	o__PeM15	f__norank_	g__norank_	s__uncultu	OTU2834
d__Bacteri	k__norank_	p__Proteob	c__Gamma	o__Acidithi	f__Acidithi	g__Acidithi	s__Acidithi	OTU4118
d__Bacteri	k__norank_	p__Firmicu	c__Clostrid	o__Clostrid	f__Helioba	g__Hydrog	s__uncultu	OTU1493
d__Bacteri	k__norank_	p__Proteob	c__Gamma	o__Betapro	f__Burkhol	g__norank_	s__uncultu	OTU2461
d__Bacteri	k__norank_	p__Depend	c__Babelia	o__Babelia	f__Vermiph	g__norank_	s__uncultu	OTU2349
d__Bacteri	k__norank_	p__Acidoba	c__Thermo	o__Thermo	f__Thermo	g__Subgrou	s__unclassi	OTU2425
d__Bacteri	k__norank_	p__Proteob	c__Alphapr	o__Sphingo	f__Sphingo	g__Sphingo	s__uncultu	OTU3565
d__Bacteri	k__norank_	p__Gemma	c__Gemma	o__norank_	f__norank_	g__norank_	s__uncultu	OTU2794
d__Bacteri	k__norank_	p__Bactero	c__Bactero	o__Cytoph	f__Hymeno	g__Adhaeri	s__uncultu	OTU1480
d__Bacteri	k__norank_	p__Bactero	c__Bactero	o__Flavoba	f__Weekse	g__Chishui	s__Chishui	OTU2615
d__Bacteri	k__norank_	p__Firmicu	c__Clostrid	o__Clostrid	f__Peptocc	g__norank_	s__uncultu	OTU1286
d__Bacteri	k__norank_	p__Actinob	c__Actinob	o__Propion	f__Nocardi	g__Nocardi	s__unclassi	OTU1489
d__Bacteri	k__norank_	p__Proteob	c__Alphapr	o__Paracae	f__Paracae	g__Candida	s__uncultu	OTU2000
d__Bacteri	k__norank_	p__Depend	c__Babelia	o__Babelia	f__UBA124	g__norank_	s__uncultu	OTU2169
d__Bacteri	k__norank_	p__Firmicu	c__Clostrid	o__Clostrid	f__Clostridi	g__Protein	s__uncultu	OTU1548
d__Bacteri	k__norank_	p__Proteob	c__Deltapr	o__Myxocc	f__norank_	g__norank_	s__unclassi	OTU1887
d__Bacteri	k__norank_	p__Firmicu	c__Clostrid	o__Clostrid	f__Christer	g__Christer	s__unclassi	OTU1245
d__Bacteri	k__norank_	p__Depend	c__Babelia	o__Babelia	f__UBA124	g__norank_	s__uncultu	OTU2167
d__Bacteri	k__norank_	p__Depend	c__Babelia	o__Babelia	f__unclassi	g__unclass	s__unclassi	OTU2107
d__Bacteri	k__norank_	p__Proteob	c__Deltapr	o__Myxocc	f__P3OB-42	g__norank_	s__uncultu	OTU2262
d__Bacteri	k__norank_	p__Proteob	c__Gamma	o__Oceano	f__Halomo	g__Halomo	s__Halomo	OTU2576
d__Bacteri	k__norank_	p__Proteob	c__Gamma	o__norank_	f__norank_	g__norank_	s__uncultu	OTU2191
d__Bacteri	k__norank_	p__Chlamy	c__Chlamy	o__Chlamy	f__Parachla	g__unclass	s__unclassi	OTU2235
d__Bacteri	k__norank_	p__Proteob	c__Deltapr	o__Bdellov	f__Bacterio	g__Peredib	s__metage	OTU1309
d__Bacteri	k__norank_	p__Cyanob	c__Oxypho	o__Chlorop	f__norank_	g__norank_	s__unclassi	OTU1708
d__Bacteri	k__norank_	p__Acidoba	c__Subgrou	o__norank_	f__norank_	g__norank_	s__uncultu	OTU946
d__Bacteri	k__norank_	p__Proteob	c__Alphapr	o__Micavib	f__norank_	g__norank_	s__uncultu	OTU1360
d__Bacteri	k__norank_	p__Chlorof	c__Dehaloc	o__S085	f__norank_	g__norank_	s__uncultu	OTU1869
d__Bacteri	k__norank_	p__Proteob	c__Gamma	o__Diploric	f__Diploric	g__norank_	s__unclassi	OTU1415
d__Bacteri	k__norank_	p__Firmicu	c__Clostrid	o__Clostrid	f__Clostridi	g__Clostrid	s__Clostrid	OTU630
d__Bacteri	k__norank_	p__Bactero	c__Bactero	o__Chitino	f__Chitinop	g__Sedimir	s__uncultu	OTU1111
d__Bacteri	k__norank_	p__Depend	c__Babelia	o__Babelia	f__Vermiph	g__norank_	s__unclassi	OTU1720

d__Bacteri	k__norank_	p__Chlamy	c__Chlamy	o__Chlamy	f__cvE6	g__norank_	s__uncultu	OTU2462
d__Bacteri	k__norank_	p__Proteob	c__Deltapr	o__Myxocc	f__Haliangi	g__Haliang	s__unclassi	OTU1816
d__Bacteri	k__norank_	p__Bactero	c__Rhodoth	o__Rhodot	f__Rhodoth	g__norank_	s__uncultu	OTU1841
d__Bacteri	k__norank_	p__Proteob	c__Gamma	o__Diploric	f__Diploric	g__Aquicel	s__uncultu	OTU2693
d__Bacteri	k__norank_	p__Verruco	c__Verruco	o__Chthon	f__Chthoni	g__Chthon	s__unclassi	OTU1261
d__Bacteri	k__norank_	p__Actinob	c__Actinob	o__Frankia	f__Sporicht	g__hgcI_cla	s__uncultu	OTU859
d__Bacteri	k__norank_	p__Bactero	c__Bactero	o__SM1A0	f__norank_	g__norank_	s__metage	OTU1056
d__Bacteri	k__norank_	p__Bactero	c__Bactero	o__Cytopha	f__Spirosor	g__Arcicella	s__uncultu	OTU1632
d__Bacteri	k__norank_	p__Gemma	c__Gemma	o__Gemma	f__Gemma	g__norank_	s__unclassi	OTU1600
d__Bacteri	k__norank_	p__Firmicu	c__Clostrid	o__Clostrid	f__Peptocc	g__Thermin	s__uncultu	OTU1048
d__Bacteri	k__norank_	p__Proteob	c__Gamma	o__Legione	f__Legione	g__Legione	s__unclassi	OTU2323
d__Bacteri	k__norank_	p__Bactero	c__Bactero	o__Sphingo	f__Sphingo	g__Pedoba	s__uncultu	OTU345
d__Bacteri	k__norank_	p__Actinob	c__Actinob	o__Frankia	f__Sporicht	g__hgcI_cla	s__uncultu	OTU948
d__Bacteri	k__norank_	p__Cyanob	c__Melaina	o__Gastran	f__norank_	g__norank_	s__unclassi	OTU999
d__Bacteri	k__norank_	p__Proteob	c__Deltapr	o__Bdellov	f__Bacteric	g__Bacterio	s__uncultu	OTU2046
d__Bacteri	k__norank_	p__Proteob	c__Gamma	o__Salinisp	f__Solimon	g__Nevskia	s__Nevskia	OTU2731
d__Bacteri	k__norank_	p__Firmicu	c__Negativ	o__Selenor	f__Veillone	g__Selenor	s__unclassi	OTU1882
d__Bacteri	k__norank_	p__Bactero	c__Bactero	o__Bactero	f__Rikenell	g__Rikenel	s__uncultu	OTU2318
d__Bacteri	k__norank_	p__Bactero	c__Ignavib	o__SJA-28	f__norank_	g__norank_	s__uncultu	OTU1987
d__Bacteri	k__norank_	p__Chlorof	c__Dehaloc	o__SAR202	f__norank_	g__norank_	s__hydroth	OTU1954
d__Bacteri	k__norank_	p__Proteob	c__Gamma	o__Xantho	f__Rhodan	g__Metallib	s__uncultu	OTU4122
d__Bacteri	k__norank_	p__Proteob	c__Alphapr	o__Acetob	f__Acetoba	g__Roseom	s__unclassi	OTU2088
d__Bacteri	k__norank_	p__Actinob	c__Actinob	o__Solirubr	f__67-14	g__norank_	s__Solirubr	OTU1536
d__Bacteri	k__norank_	p__Proteob	c__Deltapr	o__Bdellov	f__Bdellovi	g__Bdellov	s__uncultu	OTU550
d__Bacteri	k__norank_	p__Proteob	c__Alphapr	o__Ricketts	f__Ricketts	g__norank_	s__metage	OTU2280
d__Bacteri	k__norank_	p__Bactero	c__Bactero	o__Flavoba	f__Flavoba	g__Flavoba	s__unclassi	OTU2309
d__Bacteri	k__norank_	p__Bactero	c__Bactero	o__Cytopha	f__Microsc	g__Flexibac	s__unclassi	OTU1769
d__Bacteri	k__norank_	p__Bactero	c__Bactero	o__Bactero	f__Bactero	g__Bactero	s__unclassi	OTU1693
d__Bacteri	k__norank_	p__Patescib	c__Sacchar	o__Sacchar	f__unclassi	g__unclass	s__unclassi	OTU254
d__Bacteri	k__norank_	p__Proteob	c__Deltapr	o__Bradym	f__norank_	g__norank_	s__uncultu	OTU1754
d__Bacteri	k__norank_	p__Latescib	c__Latescib	o__norank_	f__norank_	g__norank_	s__unclassi	OTU2043
d__Bacteri	k__norank_	p__Chlamy	c__Chlamy	o__Chlamy	f__Parachla	g__Neochla	s__unclassi	OTU1793
d__Bacteri	k__norank_	p__Bactero	c__Bactero	o__Chitino	f__Chitinop	g__Flaviaes	s__uncultu	OTU1320
d__Bacteri	k__norank_	p__Fusoba	c__Fusobac	o__Fusoba	f__Fusobac	g__Cetobac	s__uncultu	OTU1285
d__Bacteri	k__norank_	p__Depend	c__Babelia	o__Babelia	f__Vermiph	g__norank_	s__unclassi	OTU2400
d__Bacteri	k__norank_	p__Proteob	c__Gamma	o__Betapro	f__Nitrosor	g__IS-44	s__uncultu	OTU2189
d__Bacteri	k__norank_	p__Proteob	c__Deltapr	o__Bdellov	f__Bdellovi	g__Bdellov	s__Bdellovi	OTU3202
d__Bacteri	k__norank_	p__Proteob	c__Gamma	o__KI89A_	f__norank_	g__norank_	s__unclassi	OTU3470
d__Bacteri	k__norank_	p__Chlamy	c__Chlamy	o__Chlamy	f__Parachla	g__Neochla	s__metage	OTU3014
d__Bacteri	k__norank_	p__Proteob	c__Gamma	o__unclass	f__unclassi	g__unclass	s__unclassi	OTU2115
d__Bacteri	k__norank_	p__Chlamy	c__Chlamy	o__Chlamy	f__Parachla	g__Neochla	s__uncultu	OTU2458
d__Bacteri	k__norank_	p__Bactero	c__Bactero	o__Bactero	f__Rikenell	g__Alistipe	s__Tidjanib	OTU1083
d__Bacteri	k__norank_	p__Armatin	c__Armatin	o__Armatin	f__Armatin	g__Armatin	s__uncultu	OTU1781
d__Bacteri	k__norank_	p__Gemma	c__Gemma	o__norank_	f__norank_	g__norank_	s__unclassi	OTU2187
d__Bacteri	k__norank_	p__Proteob	c__Deltapr	o__Myxocc	f__Nannoc	g__Nannoc	s__Nannoc	OTU1589
d__Bacteri	k__norank_	p__Proteob	c__Gamma	o__Pasteur	f__Pasteur	g__Haemop	s__Haemop	OTU791
d__Bacteri	k__norank_	p__Proteob	c__Deltapr	o__unclass	f__unclassi	g__unclass	s__unclassi	OTU2825

d__Bacteri	k__norank_	p__Firmicu	c__Clostrid	o__Clostrid	f__Lachnos	g__unclass	s__unclassi	OTU47
d__Bacteri	k__norank_	p__Bacterc	c__Bactero	o__Bacterc	f__Bactero	g__Bactero	s__Bactero	OTU2431
d__Bacteri	k__norank_	p__Firmicu	c__Bacilli	o__Bacillal	f__Thermo	g__Planifilu	s__uncultu	OTU1412
d__Bacteri	k__norank_	p__Proteol	c__Deltapr	o__Bdellov	f__Bacteric	g__Peredib	s__unclassi	OTU1639
d__Bacteri	k__norank_	p__Chlorof	c__JG30-KF	o__norank_	f__norank_	g__norank_	s__uncultu	OTU1986
d__Bacteri	k__norank_	p__Bacterc	c__Bactero	o__Sphingo	f__KD3-93	g__norank_	s__metage	OTU4025
d__Bacteri	k__norank_	p__Firmicu	c__Bacilli	o__Bacillal	f__Thermo	g__Planifilu	s__Planifilu	OTU997
d__Bacteri	k__norank_	p__Proteol	c__Gamma	o__Diploric	f__Diploric	g__Aquicel	s__unclassi	OTU1911
d__Bacteri	k__norank_	p__Proteol	c__Gamma	o__Alteron	f__Alterom	g__Rheinhe	s__Rheinhe	OTU249
d__Bacteri	k__norank_	p__Bacterc	c__Bactero	o__Flavoba	f__Flavoba	g__Capnoc	s__Capnoc	OTU770
d__Bacteri	k__norank_	p__Proteol	c__Gamma	o__Diploric	f__Diploric	g__Aquicel	s__uncultu	OTU2206
d__Bacteri	k__norank_	p__Cyanob	c__Sericytc	o__norank_	f__norank_	g__norank_	s__uncultu	OTU2803
d__Bacteri	k__norank_	p__Actinol	c__Actinob	o__IMCC26	f__norank_	g__norank_	s__uncultu	OTU1791
d__Bacteri	k__norank_	p__Firmicu	c__Clostrid	o__Clostrid	f__Rumino	g__Eubacte	s__gut_me	OTU2485
d__Bacteri	k__norank_	p__Proteol	c__Gamma	o__Legione	f__Legione	g__Legione	s__unclassi	OTU1661
d__Bacteri	k__norank_	p__Proteol	c__Gamma	o__Enterol	f__Enterob	g__Yersinia	s__Yersinia	OTU2168
d__Bacteri	k__norank_	p__Proteol	c__Gamma	o__Steroid	f__Steroidc	g__Steroid	s__uncultu	OTU3758
d__Bacteri	k__norank_	p__Firmicu	c__Erysipel	o__Erysipe	f__Erysipel	g__Faecalit	s__uncultu	OTU918
d__Bacteri	k__norank_	p__Chlorof	c__Anaerol	o__Caldilin	f__Caldiline	g__norank_	s__unclassi	OTU1188
d__Bacteri	k__norank_	p__Firmicu	c__Clostrid	o__Clostrid	f__Rumino	g__Ruminic	s__unclassi	OTU1003
d__Bacteri	k__norank_	p__Proteol	c__Gamma	o__Diploric	f__Diploric	g__Aquicel	s__unclassi	OTU2930
d__Bacteri	k__norank_	p__Proteol	c__Gamma	o__Betaprc	f__Ferrova	g__Ferrovu	s__uncultu	OTU1236
d__Bacteri	k__norank_	p__Firmicu	c__Clostrid	o__Clostrid	f__Peptocc	g__Desulfo	s__uncultu	OTU1434
d__Bacteri	k__norank_	p__Depenc	c__Babelia	o__Babelia	f__Vermipl	g__norank_	s__uncultu	OTU1653
d__Bacteri	k__norank_	p__Depenc	c__Babelia	o__Babelia	f__Vermipl	g__norank_	s__uncultu	OTU1655
d__Bacteri	k__norank_	p__Firmicu	c__Clostrid	o__Clostrid	f__Rumino	g__Ruminic	s__uncultu	OTU1020
d__Bacteri	k__norank_	p__Proteol	c__Gamma	o__Pseudo	f__Moraxe	g__Alkanin	s__uncultu	OTU1591
d__Bacteri	k__norank_	p__Proteol	c__Gamma	o__Diploric	f__Diploric	g__norank_	s__uncultu	OTU1884
d__Bacteri	k__norank_	p__Proteol	c__Alphapr	o__Sneathi	f__Sneathi	g__AT-s3-4	s__uncultu	OTU2315
d__Bacteri	k__norank_	p__Proteol	c__Gamma	o__Gamma	f__unclassi	g__Candida	s__uncultu	OTU1202
d__Bacteri	k__norank_	p__Patescil	c__Sacchar	o__Sacchar	f__Sacchar	g__norank_	s__uncultu	OTU1484
d__Bacteri	k__norank_	p__Bacterc	c__Bactero	o__Cytoph	f__Spiroso	g__Dyadob	s__metage	OTU1143
d__Bacteri	k__norank_	p__Proteol	c__Alphapr	o__Sphingo	f__Sphingo	g__Sphingo	s__Sphingo	OTU1313
d__Bacteri	k__norank_	p__Proteol	c__Alphapr	o__Sphingo	f__Sphingo	g__Alterery	s__Alterery	OTU234
d__Bacteri	k__norank_	p__Proteol	c__Gamma	o__Oceanc	f__Pseudol	g__Pseudo	s__uncultu	OTU2281
d__Bacteri	k__norank_	p__Firmicu	c__Bacilli	o__Bacillal	f__Paeniba	g__Breviba	s__unclassi	OTU1547
d__Bacteri	k__norank_	p__Acidoba	c__Subgrou	o__norank_	f__norank_	g__norank_	s__uncultu	OTU2697
d__Bacteri	k__norank_	p__Depenc	c__Babelia	o__Babelia	f__Vermipl	g__norank_	s__uncultu	OTU1784
d__Bacteri	k__norank_	p__Proteol	c__Alphapr	o__Microp	f__Micrope	g__norank_	s__metage	OTU3963
d__Bacteri	k__norank_	p__Proteol	c__Alphapr	o__Rhodol	f__Rhodob	g__unclass	s__unclassi	OTU260
d__Bacteri	k__norank_	p__Acidoba	c__Acidoba	o__Solibac	f__Solibact	g__unclass	s__unclassi	OTU1988
d__Bacteri	k__norank_	p__Actinol	c__Actinob	o__Microtr	f__Ilumato	g__CL500-2	s__metage	OTU1464
d__Bacteri	k__norank_	p__Proteol	c__Alphapr	o__Rhizobi	f__Beijerin	g__Methylc	s__Methylc	OTU20
d__Bacteri	k__norank_	p__Firmicu	c__Clostrid	o__Clostrid	f__Clostridi	g__Clostrid	s__Clostrid	OTU1466
d__Bacteri	k__norank_	p__Proteol	c__Deltapr	o__Myxocc	f__Blfdi19	g__norank_	s__uncultu	OTU2556
d__Bacteri	k__norank_	p__Depenc	c__Babelia	o__Babelia	f__UBA124	g__norank_	s__uncultu	OTU2432
d__Bacteri	k__norank_	p__Proteol	c__Gamma	o__Betaprc	f__Burkhol	g__Delftia	s__Delftia_	OTU1513

d__Bacteri	k__norank_	p__Proteob	c__Gamma	o__Betapr	f__Nitroso	g__MND1	s__unclassi	OTU2394
d__Bacteri	k__norank_	p__Actinob	c__Actinob	o__Coryne	f__Coryneb	g__Coryne	s__uncultu	OTU1201
d__Bacteri	k__norank_	p__Firmicu	c__Clostrid	o__Clostrid	f__Lachnos	g__Lachno	s__metage	OTU50
d__Bacteri	k__norank_	p__Proteob	c__Gamma	o__Pseudo	f__Moraxe	g__unclass	s__unclassi	OTU1530
d__Bacteri	k__norank_	p__Bacterc	c__Bactero	o__Bacterc	f__Prevote	g__Prevote	s__unclassi	OTU1917
d__Bacteri	k__norank_	p__Proteob	c__Deltapr	o__Myxocc	f__Blfdi19	g__norank_	s__uncultu	OTU1608
d__Bacteri	k__norank_	p__Proteob	c__Gamma	o__Gamma	f__unclassi	g__norank_	s__metage	OTU2812
d__Bacteri	k__norank_	p__Acidoba	c__unclassi	o__unclass	f__unclassi	g__unclass	s__unclassi	OTU2296
d__Bacteri	k__norank_	p__Firmicu	c__Bacilli	o__Bacillal	f__Paeniba	g__Paeniba	s__Paeniba	OTU1181
d__Bacteri	k__norank_	p__Firmicu	c__Clostrid	o__Clostrid	f__Lachnos	g__Marvinl	s__unclassi	OTU2022
d__Bacteri	k__norank_	p__Firmicu	c__Bacilli	o__Bacillal	f__Planoco	g__Paenisp	s__uncultu	OTU545
d__Bacteri	k__norank_	p__Bacterc	c__Bactero	o__Chitino	f__Chitinop	g__Dinghui	s__unclassi	OTU1650
d__Bacteri	k__norank_	p__Lentisp	c__Lentispl	o__Victival	f__unclassi	g__unclass	s__unclassi	OTU1280
d__Bacteri	k__norank_	p__Firmicu	c__Clostrid	o__Clostrid	f__unclassi	g__unclass	s__unclassi	OTU1327
d__Bacteri	k__norank_	p__Bacterc	c__Bactero	o__Bacterc	f__unclassi	g__unclass	s__unclassi	OTU2325
d__Bacteri	k__norank_	p__Firmicu	c__Bacilli	o__Bacillal	f__Paeniba	g__Paeniba	s__unclassi	OTU1390
d__Bacteri	k__norank_	p__Proteob	c__Deltapr	o__Myxocc	f__Blfdi19	g__norank_	s__uncultu	OTU4042
d__Bacteri	k__norank_	p__unclass	c__unclassi	o__unclass	f__unclassi	g__unclass	s__unclassi	OTU1572
d__Bacteri	k__norank_	p__Teneric	c__Mollicu	o__Izimapl	f__norank_	g__norank_	s__uncultu	OTU2435
d__Bacteri	k__norank_	p__Plancto	c__Phycisp	o__Phycisp	f__Phycispl	g__CL500-3	s__uncultu	OTU2739
d__Bacteri	k__norank_	p__Proteob	c__Gamma	o__Betapr	f__Burkhol	g__unclass	s__unclassi	OTU2256
d__Bacteri	k__norank_	p__Chlorof	c__Anaerol	o__SBR103	f__norank_	g__norank_	s__unclassi	OTU2123
d__Bacteri	k__norank_	p__Chlamy	c__Chlamy	o__Chlamy	f__Parachla	g__Neochla	s__uncultu	OTU2604
d__Bacteri	k__norank_	p__Proteob	c__Gamma	o__Alteron	f__Psychro	g__Psychro	s__Psychro	OTU693
d__Bacteri	k__norank_	p__Chlamy	c__Chlamy	o__Chlamy	f__Parachla	g__Neochla	s__unclassi	OTU1414
d__Bacteri	k__norank_	p__Proteob	c__Alphapr	o__unclass	f__unclassi	g__unclass	s__unclassi	OTU1668
d__Bacteri	k__norank_	p__Proteob	c__Gamma	o__Pseudo	f__Pseudor	g__Pseudo	s__unclassi	OTU2350
d__Bacteri	k__norank_	p__Acidoba	c__Holopha	o__Holoph	f__Holopha	g__unclass	s__unclassi	OTU2500
d__Bacteri	k__norank_	p__Proteob	c__Gamma	o__Pisciricl	f__Piscirick	g__Candida	s__metage	OTU1934
d__Bacteri	k__norank_	p__Firmicu	c__Clostrid	o__Clostrid	f__Rumino	g__GCA-90	s__uncultu	OTU1647
d__Bacteri	k__norank_	p__Chlorof	c__Anaerol	o__SBR103	f__norank_	g__norank_	s__unclassi	OTU1617
d__Bacteri	k__norank_	p__Depenc	c__Babelia	o__Babelia	f__UBA124	g__norank_	s__unclassi	OTU2605
d__Bacteri	k__norank_	p__Firmicu	c__Bacilli	o__Bacillal	f__Paeniba	g__Paeniba	s__unclassi	OTU1067
d__Bacteri	k__norank_	p__Firmicu	c__Bacilli	o__Bacillal	f__Paeniba	g__Paeniba	s__unclassi	OTU1063
d__Bacteri	k__norank_	p__Proteob	c__unclassi	o__unclass	f__unclassi	g__unclass	s__unclassi	OTU1295
d__Bacteri	k__norank_	p__Proteob	c__Gamma	o__Betapr	f__Leeiacea	g__norank_	s__uncultu	OTU1707
d__Bacteri	k__norank_	p__Firmicu	c__Negativ	o__Selenor	f__Veillone	g__Megasp	s__uncultu	OTU2470
d__Bacteri	k__norank_	p__Firmicu	c__Bacilli	o__Bacillal	f__Paeniba	g__Paeniba	s__unclassi	OTU1069
d__Bacteri	k__norank_	p__Bacterc	c__Ignavib	o__Kryptor	f__BSV26	g__norank_	s__uncultu	OTU1873
d__Bacteri	k__norank_	p__Proteob	c__Alphapr	o__Cauloba	f__Cauloba	g__norank_	s__uncultu	OTU3001
d__Bacteri	k__norank_	p__Actinob	c__Actinob	o__Microc	f__Microba	g__Lysinim	s__metage	OTU2879
d__Bacteri	k__norank_	p__Cyanob	c__Oxypho	o__Chlorop	f__norank_	g__norank_	s__unclassi	OTU1774
d__Bacteri	k__norank_	p__Proteob	c__Alphapr	o__Paracae	f__Paracae	g__Candida	s__unclassi	OTU1560
d__Bacteri	k__norank_	p__Firmicu	c__Clostrid	o__Clostrid	f__Gracilib	g__Lutispo	s__uncultu	OTU1537
d__Bacteri	k__norank_	p__Actinob	c__Actinob	o__Propior	f__Nocardi	g__Nocardi	s__unclassi	OTU1496
d__Bacteri	k__norank_	p__Proteob	c__Alphapr	o__Acetoba	f__Acetoba	g__unclass	s__unclassi	OTU4111
d__Bacteri	k__norank_	p__Proteob	c__Gamma	o__Coxiella	f__Coxiella	g__Coxiella	s__unclassi	OTU2112

d__Bacteri	k__norank_	p__Proteol	c__Gamma	o__Piscirick	f__Piscirick	g__Candida	s__metage	OTU1734
d__Bacteri	k__norank_	p__Patescil	c__Sacchar	o__Sacchar	f__norank_	g__norank_	s__Candida	OTU1197
d__Bacteri	k__norank_	p__Proteol	c__Alphapr	o__Rhodos	f__Magnet	g__norank_	s__uncultu	OTU2013
d__Bacteri	k__norank_	p__Proteol	c__Deltapr	o__Myxocc	f__Archang	g__Anaero	s__unclassi	OTU1255
d__Bacteri	k__norank_	p__Chlorof	c__Anaerol	o__unclass	f__unclassi	g__unclass	s__unclassi	OTU2821
d__Bacteri	k__norank_	p__Depenc	c__Babelia	o__Babelia	f__Vermipl	g__norank_	s__uncultu	OTU2790
d__Bacteri	k__norank_	p__Proteol	c__Gamma	o__Betapro	f__Gallione	g__unclass	s__unclassi	OTU2533
d__Bacteri	k__norank_	p__Proteol	c__Gamma	o__Betapro	f__Burkhol	g__Massilia	s__unclassi	OTU598
d__Bacteri	k__norank_	p__Firmicu	c__Clostrid	o__Clostrid	f__Christer	g__unclass	s__unclassi	OTU1467
d__Bacteri	k__norank_	p__Proteol	c__Alphapr	o__Sphingo	f__Sphingo	g__Alterery	s__uncultu	OTU3940
d__Bacteri	k__norank_	p__Proteol	c__Gamma	o__Coxiella	f__Coxiella	g__Coxiella	s__uncultu	OTU2135
d__Bacteri	k__norank_	p__Firmicu	c__Clostrid	o__Clostrid	f__Lachnos	g__Epulopi	s__Niamey	OTU1499
d__Bacteri	k__norank_	p__Depenc	c__Babelia	o__Babelia	f__unclassi	g__unclass	s__unclassi	OTU1697
d__Bacteri	k__norank_	p__Depenc	c__Babelia	o__Babelia	f__Babelia	g__norank_	s__uncultu	OTU1817
d__Bacteri	k__norank_	p__Plancto	c__Plancto	o__Gemma	f__Gemma	g__norank_	s__uncultu	OTU49
d__Bacteri	k__norank_	p__Proteol	c__Gamma	o__Pseudo	f__Pseudor	g__Pseudo	s__Pseudo	OTU1751
d__Bacteri	k__norank_	p__Depenc	c__Babelia	o__Babelia	f__Vermipl	g__norank_	s__uncultu	OTU2316
d__Bacteri	k__norank_	p__Firmicu	c__Negativ	o__Selenor	f__Veillone	g__unclass	s__unclassi	OTU1240
d__Bacteri	k__norank_	p__Proteol	c__Gamma	o__Legione	f__Legione	g__Legione	s__unclassi	OTU655
d__Bacteri	k__norank_	p__Bacterc	c__Bactero	o__Bacterc	f__Muribac	g__norank_	s__uncultu	OTU2276
d__Bacteri	k__norank_	p__Proteol	c__Alphapr	o__Rhodob	f__Rhodob	g__Defluvii	s__unclassi	OTU1373
d__Bacteri	k__norank_	p__Actinob	c__Actinob	o__Actinor	f__Actinom	g__Actinon	s__Actinon	OTU1283
d__Bacteri	k__norank_	p__Patescil	c__WS6_D	o__norank_	f__norank_	g__norank_	s__unclassi	OTU3998
d__Bacteri	k__norank_	p__Firmicu	c__Clostrid	o__Clostrid	f__Rumino	g__Ruminc	s__unclassi	OTU1086
d__Bacteri	k__norank_	p__Proteol	c__Deltapr	o__Desulfo	f__Desulfol	g__Desulfo	s__unclassi	OTU1052
d__Bacteri	k__norank_	p__Patescil	c__Sacchar	o__Sacchar	f__Sacchar	g__norank_	s__uncultu	OTU815
d__Bacteri	k__norank_	p__Proteol	c__Gamma	o__Oceanc	f__Oleiphil	g__Oleiphil	s__uncultu	OTU3747
d__Bacteri	k__norank_	p__Proteol	c__Gamma	o__Legione	f__Legione	g__Legione	s__unclassi	OTU2305
d__Bacteri	k__norank_	p__Actinob	c__Actinob	o__Pseudo	f__Pseudor	g__Sacchar	s__unclassi	OTU892
d__Bacteri	k__norank_	p__Proteol	c__Gamma	o__Pseudo	f__unclassi	g__unclass	s__unclassi	OTU2334
d__Bacteri	k__norank_	p__Depenc	c__Babelia	o__Babelia	f__unclassi	g__unclass	s__unclassi	OTU1967
d__Bacteri	k__norank_	p__Proteol	c__Alphapr	o__Rhizobi	f__Devosia	g__Devosia	s__unclassi	OTU3110
d__Bacteri	k__norank_	p__Firmicu	c__Clostrid	o__Clostrid	f__Lachnos	g__Blautia	s__Rumino	OTU629
d__Bacteri	k__norank_	p__Chlamy	c__Chlamy	o__Chlamy	f__Parachla	g__Neochla	s__metage	OTU1695
d__Bacteri	k__norank_	p__Nitrosp	c__Nitrosp	o__norank_	f__norank_	g__Nitrosp	s__Nitrospi	OTU1091
d__Bacteri	k__norank_	p__Firmicu	c__Bacilli	o__Lactoba	f__Lactoba	g__Lactoba	s__unclassi	OTU912
d__Bacteri	k__norank_	p__Proteol	c__Gamma	o__Betapro	f__Burkhol	g__Massilia	s__Massilia	OTU881
d__Bacteri	k__norank_	p__Chlorof	c__KD4-96	o__norank_	f__norank_	g__norank_	s__unclassi	OTU4048
d__Bacteri	k__norank_	p__Proteol	c__Gamma	o__Pseudo	f__Moraxe	g__Alkanin	s__unclassi	OTU2025
d__Bacteri	k__norank_	p__Rokuba	c__NC10	o__Rokuba	f__norank_	g__norank_	s__uncultu	OTU3482
d__Bacteri	k__norank_	p__Chlamy	c__Chlamy	o__Chlamy	f__unclassi	g__unclass	s__unclassi	OTU2106
d__Bacteri	k__norank_	p__Cyanob	c__Melaina	o__Gastrar	f__norank_	g__norank_	s__uncultu	OTU1552
d__Bacteri	k__norank_	p__Proteol	c__Alphapr	o__Sphingo	f__Sphingo	g__Sphingo	s__Sphingo	OTU679
d__Bacteri	k__norank_	p__Bacterc	c__Bactero	o__unclass	f__unclassi	g__unclass	s__unclassi	OTU1227
d__Bacteri	k__norank_	p__Bacterc	c__Bactero	o__Bacterc	f__F082	g__norank_	s__uncultu	OTU2852
d__Bacteri	k__norank_	p__Proteol	c__Gamma	o__unclass	f__unclassi	g__unclass	s__unclassi	OTU2620
d__Bacteri	k__norank_	p__Chlamy	c__Chlamy	o__Chlamy	f__Parachla	g__unclass	s__unclassi	OTU2710

d__Bacteri	k__norank_	p__Depenc	c__Babelia	o__Babelia	f__Babelia	g__norank_	s__uncultu	OTU2109
d__Bacteri	k__norank_	p__Proteol	c__Alphapr	o__Rhizobi	f__Methylo	g__norank_	s__unclassi	OTU2994
d__Bacteri	k__norank_	p__Proteol	c__Alphapr	o__Ricketts	f__Mitocho	g__norank_	s__uncultu	OTU2160
d__Bacteri	k__norank_	p__Verrucc	c__Verrucc	o__norank_	f__norank_	g__norank_	s__metage	OTU1075
d__Bacteri	k__norank_	p__Depenc	c__Babelia	o__Babelia	f__Babelia	g__norank_	s__uncultu	OTU1422
d__Bacteri	k__norank_	p__Proteol	c__Gamma	o__Pseudo	f__Moraxe	g__Cavicell	s__uncultu	OTU1269
d__Bacteri	k__norank_	p__Proteol	c__Alphapr	o__Sneathi	f__Sneathi	g__norank_	s__uncultu	OTU2048
d__Bacteri	k__norank_	p__Proteol	c__Deltapr	o__Myxocc	f__Nannoc	g__Nannoc	s__uncultu	OTU2348
d__Bacteri	k__norank_	p__Proteol	c__Gamma	o__Coxiella	f__Coxiella	g__Coxiella	s__unclassi	OTU2807
d__Bacteri	k__norank_	p__Bacterc	c__Bactero	o__Flavoba	f__Weekse	g__Chrysec	s__Chrysec	OTU1479
d__Bacteri	k__norank_	p__Proteol	c__Alphapr	o__Azospir	f__norank_	g__norank_	s__uncultu	OTU1831
d__Bacteri	k__norank_	p__Chlorof	c__TK10	o__norank_	f__norank_	g__norank_	s__uncultu	OTU2006
d__Bacteri	k__norank_	p__Firmicu	c__Clostrid	o__Clostrid	f__Lachnos	g__Blautia	s__unclassi	OTU1665
d__Bacteri	k__norank_	p__Proteol	c__Gamma	o__SAR86_	f__norank_	g__norank_	s__uncultu	OTU2454
d__Bacteri	k__norank_	p__Chlamy	c__Chlamy	o__Chlamy	f__cvE6	g__norank_	s__metage	OTU2389
d__Bacteri	k__norank_	p__Depenc	c__Babelia	o__Babelia	f__Vermipl	g__norank_	s__uncultu	OTU1681
d__Bacteri	k__norank_	p__Cyanob	c__Oxypho	o__Synech	f__Cyanobi	g__Cyanob	s__Synech	OTU1788
d__Bacteri	k__norank_	p__Verrucc	c__Verrucc	o__Verrucc	f__Rubrital	g__Luteolil	s__unclassi	OTU1410
d__Bacteri	k__norank_	p__Cyanob	c__Oxypho	o__Chlorop	f__norank_	g__norank_	s__unclassi	OTU1999
d__Bacteri	k__norank_	p__Proteol	c__Alphapr	o__Rhizobi	f__Xanthol	g__unclass	s__unclassi	OTU2
d__Bacteri	k__norank_	p__Proteol	c__Gamma	o__Diploric	f__Diploric	g__norank_	s__unclassi	OTU2755
d__Bacteri	k__norank_	p__Depenc	c__Babelia	o__Babelia	f__Vermipl	g__norank_	s__unclassi	OTU2278
d__Bacteri	k__norank_	p__Cyanob	c__Oxypho	o__Oxyphc	f__unclassi	g__Calothr	s__uncultu	OTU2035
d__Bacteri	k__norank_	p__Firmicu	c__Bacilli	o__Bacillal	f__Family_	g__Exiguol	s__unclassi	OTU2517
d__Bacteri	k__norank_	p__Depenc	c__Babelia	o__Babelia	f__Vermipl	g__norank_	s__unclassi	OTU2270
d__Bacteri	k__norank_	p__Actinol	c__Actinob	o__Gaiellal	f__Gaiellac	g__Gaiella	s__unclassi	OTU3189
d__Bacteri	k__norank_	p__Chlorof	c__KD4-96	o__norank_	f__norank_	g__norank_	s__uncultu	OTU729
d__Bacteri	k__norank_	p__Bacterc	c__Bactero	o__Chitino	f__Saprosp	g__norank_	s__uncultu	OTU1878
d__Bacteri	k__norank_	p__Acidoba	c__Subgrou	o__norank_	f__norank_	g__norank_	s__uncultu	OTU2654
d__Bacteri	k__norank_	p__Bacterc	c__Bactero	o__Bacterc	f__Prevote	g__Prevote	s__uncultu	OTU1141
d__Bacteri	k__norank_	p__Bacterc	c__Bactero	o__Bacterc	f__Bactero	g__Bactero	s__unclassi	OTU2227
d__Bacteri	k__norank_	p__Acidoba	c__Blastoc	o__DS-100	f__norank_	g__norank_	s__uncultu	OTU2005
d__Bacteri	k__norank_	p__Proteol	c__Alphapr	o__Rhodol	f__Rhodob	g__unclass	s__unclassi	OTU499
d__Bacteri	k__norank_	p__Verrucc	c__Verrucc	o__Verrucc	f__Verruco	g__norank_	s__metage	OTU1265
d__Bacteri	k__norank_	p__Proteol	c__Deltapr	o__SAR324	f__norank_	g__norank_	s__unclassi	OTU1832
d__Bacteri	k__norank_	p__Proteol	c__Gamma	o__Diploric	f__Diploric	g__unclass	s__unclassi	OTU2211
d__Bacteri	k__norank_	p__Bacterc	c__Bactero	o__Cytoph	f__Bernard	g__Bernarc	s__Bernard	OTU1445
d__Bacteri	k__norank_	p__Firmicu	c__Bacilli	o__Bacillal	f__Thermo	g__Thermc	s__Thermo	OTU2079
d__Bacteri	k__norank_	p__Depenc	c__Babelia	o__Babelia	f__Vermipl	g__norank_	s__uncultu	OTU2819
d__Bacteri	k__norank_	p__Chlorof	c__Anaerol	o__SBR103	f__A4b	g__norank_	s__metage	OTU1924
d__Bacteri	k__norank_	p__Proteol	c__Deltapr	o__Bdellov	f__Bdellovi	g__Bdellov	s__uncultu	OTU3364
d__Bacteri	k__norank_	p__Proteol	c__Alphapr	o__Sneathi	f__Sneathi	g__AT-s3-4	s__metage	OTU1979
d__Bacteri	k__norank_	p__Proteol	c__Deltapr	o__MBNT1	f__norank_	g__norank_	s__uncultu	OTU2736
d__Bacteri	k__norank_	p__Proteol	c__Gamma	o__Steroid	f__Steroid	g__norank_	s__unclassi	OTU1933
d__Bacteri	k__norank_	p__Proteol	c__Gamma	o__Gamma	f__unclassi	g__norank_	s__metage	OTU2289
d__Bacteri	k__norank_	p__Proteol	c__Alphapr	o__Rhodos	f__norank_	g__norank_	s__uncultu	OTU279
d__Bacteri	k__norank_	p__Acidoba	c__Acidoba	o__Solibac	f__Solibact	g__Bryoba	s__unclassi	OTU2040

d__Bacteri	k__norank_	p__Depenc	c__Babelia	o__Babelia	f__UBA124	g__norank_	s__uncultu	OTU2842
d__Bacteri	k__norank_	p__Proteol	c__Gamma	o__Betapro	f__Burkhol	g__Aquaba	s__unclassi	OTU1203
d__Bacteri	k__norank_	p__Chlamy	c__Chlamy	o__Chlamy	f__Parachla	g__Neochla	s__metage	OTU2801
d__Bacteri	k__norank_	p__Armatir	c__norank_	o__norank_	f__norank_	g__norank_	s__uncultu	OTU949
d__Bacteri	k__norank_	p__Chlamy	c__Chlamy	o__Chlamy	f__cvE6	g__norank_	s__unclassi	OTU2288
d__Bacteri	k__norank_	p__Deinoco	c__Deinoco	o__Deinoco	f__Deinocc	g__Deinoco	s__Deinocc	OTU1502
d__Bacteri	k__norank_	p__Proteol	c__Gamma	o__Betapro	f__Burkhol	g__Parasut	s__uncultu	OTU142
d__Bacteri	k__norank_	p__Proteol	c__Gamma	o__Pseudo	f__Pseudor	g__Pseudo	s__Pseudoi	OTU365
d__Bacteri	k__norank_	p__Firmicu	c__Clostrid	o__Clostrid	f__Rumino	g__Ruminc	s__unclassi	OTU1121
d__Bacteri	k__norank_	p__Actinol	c__Actinob	o__Microm	f__Microm	g__unclass	s__unclassi	OTU1262
d__Bacteri	k__norank_	p__Firmicu	c__Negativ	o__Selenor	f__Veillone	g__Dialiste	s__Dialistel	OTU2402
d__Bacteri	k__norank_	p__Proteol	c__Gamma	o__Diploric	f__Diploric	g__norank_	s__unclassi	OTU1624
d__Bacteri	k__norank_	p__Proteol	c__Gamma	o__Legione	f__Legione	g__norank_	s__Legione	OTU2822
d__Bacteri	k__norank_	p__Firmicu	c__Clostrid	o__Clostrid	f__Lachnos	g__Lachno	s__unclassi	OTU2885
d__Bacteri	k__norank_	p__Chlamy	c__Chlamy	o__Chlamy	f__cvE6	g__norank_	s__unclassi	OTU1864
d__Bacteri	k__norank_	p__Proteol	c__Gamma	o__Betapro	f__Burkhol	g__Suttere	s__unclassi	OTU1259
d__Bacteri	k__norank_	p__Actinol	c__Actinob	o__Microc	f__Microba	g__Candida	s__uncultu	OTU3068
d__Bacteri	k__norank_	p__Chlamy	c__Chlamy	o__Chlamy	f__Parachla	g__Neochla	s__metage	OTU2314
d__Bacteri	k__norank_	p__Proteol	c__Gamma	o__Betapro	f__Burkhol	g__Limnob	s__uncultu	OTU978
d__Bacteri	k__norank_	p__Depenc	c__Babelia	o__Babelia	f__norank_	g__norank_	s__unclassi	OTU1730
d__Bacteri	k__norank_	p__Actinol	c__Actinob	o__Microc	f__Micrococ	g__unclass	s__unclassi	OTU1974
d__Bacteri	k__norank_	p__Chlamy	c__Chlamy	o__Chlamy	f__Parachla	g__Candida	s__metage	OTU1687
d__Bacteri	k__norank_	p__Actinol	c__Actinob	o__Frankia	f__norank_	g__norank_	s__uncultu	OTU289
d__Bacteri	k__norank_	p__Bacterc	c__Bactero	o__Cytoph	f__Spirosor	g__Dyadob	s__uncultu	OTU1462
d__Bacteri	k__norank_	p__Proteol	c__Gamma	o__Betapro	f__Chromo	g__Vogesel	s__uncultu	OTU1145
d__Bacteri	k__norank_	p__unclass	c__unclassi	o__unclass	f__unclassi	g__unclass	s__unclassi	OTU2570
d__Bacteri	k__norank_	p__Proteol	c__Alphapr	o__Ricketts	f__Mitocho	g__norank_	s__uncultu	OTU2085
d__Bacteri	k__norank_	p__Firmicu	c__Bacilli	o__Lactoba	f__Lactoba	g__Lactoba	s__unclassi	OTU720
d__Bacteri	k__norank_	p__unclass	c__unclassi	o__unclass	f__unclassi	g__unclass	s__unclassi	OTU1102
d__Bacteri	k__norank_	p__Proteol	c__Gamma	o__Betapro	f__Burkhol	g__unclass	s__unclassi	OTU1355
d__Bacteri	k__norank_	p__Chlorof	c__Anaerol	o__Anaero	f__Anaerol	g__norank_	s__uncultu	OTU2741
d__Bacteri	k__norank_	p__Firmicu	c__Bacilli	o__Bacillal	f__Bacillac	g__Bacillus	s__Bacillus_	OTU1576
d__Bacteri	k__norank_	p__Plancto	c__Plancto	o__Gemma	f__Gemma	g__norank_	s__uncultu	OTU1570
d__Bacteri	k__norank_	p__Patescil	c__Sacchar	o__Sacchar	f__Sacchar	g__Candida	s__uncultu	OTU1025
d__Bacteri	k__norank_	p__Chlamy	c__Chlamy	o__Chlamy	f__unclassi	g__unclass	s__unclassi	OTU2116
d__Bacteri	k__norank_	p__Proteol	c__Alphapr	o__unclass	f__unclassi	g__unclass	s__unclassi	OTU1982
d__Bacteri	k__norank_	p__Firmicu	c__Clostrid	o__Clostrid	f__Peptost	g__Rombo	s__uncultu	OTU1437
d__Bacteri	k__norank_	p__Firmicu	c__Negativ	o__Selenor	f__Veillone	g__Sporom	s__unclassi	OTU1432
d__Bacteri	k__norank_	p__Bacterc	c__Bactero	o__Bacterc	f__Bactero	g__Bactero	s__Bactero	OTU112
d__Bacteri	k__norank_	p__Acidoba	c__Blastoca	o__Blastoc	f__Blastoca	g__Aridiba	s__uncultu	OTU1369
d__Bacteri	k__norank_	p__Depenc	c__Babelia	o__Babelia	f__unclassi	g__unclass	s__unclassi	OTU2480
d__Bacteri	k__norank_	p__Firmicu	c__Clostrid	o__Clostrid	f__Clostridi	g__unclass	s__unclassi	OTU1095
d__Bacteri	k__norank_	p__Proteol	c__Gamma	o__Betapro	f__Burkhol	g__Rhizoba	s__unclassi	OTU3578
d__Bacteri	k__norank_	p__Proteol	c__Gamma	o__Aeromo	f__Aeromo	g__Aeromo	s__unclassi	OTU2740
d__Bacteri	k__norank_	p__Proteol	c__Gamma	o__Betapro	f__Gallione	g__Gallione	s__uncultu	OTU958
d__Bacteri	k__norank_	p__Firmicu	c__Clostrid	o__Clostrid	f__Lachnos	g__XBB100	s__uncultu	OTU2068
d__Bacteri	k__norank_	p__Firmicu	c__Clostrid	o__Clostrid	f__Rumino	g__Ruminc	s__uncultu	OTU1177

d__Bacteri	k__norank_	p__Cyanob	c__Melaina	o__Obscuri	f__norank_	g__norank_	s__uncultu	OTU1235
d__Bacteri	k__norank_	p__Firmicu	c__Clostrid	o__Clostrid	f__Clostridi	g__Clostrid	s__Clostrid	OTU1207
d__Bacteri	k__norank_	p__Bacterc	c__Bactero	o__Cytoph	f__Microsc	g__Siphonc	s__uncultu	OTU1411
d__Bacteri	k__norank_	p__Firmicu	c__Bacilli	o__Bacillal	f__Paeniba	g__Paeniba	s__unclassi	OTU1478
d__Bacteri	k__norank_	p__Chlorof	c__KD4-96	o__norank_	f__norank_	g__norank_	s__uncultu	OTU3579
d__Bacteri	k__norank_	p__Proteob	c__Gamma	o__Betaprc	f__Nitrosor	g__MND1	s__uncultu	OTU1273
d__Bacteri	k__norank_	p__Firmicu	c__Bacilli	o__Bacillal	f__Paeniba	g__Paeniba	s__unclassi	OTU1473
d__Bacteri	k__norank_	p__Depenc	c__Babelia	o__Babelia	f__UBA124	g__norank_	s__unclassi	OTU2728
d__Bacteri	k__norank_	p__Firmicu	c__Clostrid	o__Clostrid	f__Lachnos	g__unclass	s__unclassi	OTU2034
d__Bacteri	k__norank_	p__Proteob	c__Gamma	o__Betaprc	f__Chitinib	g__Iodobac	s__unclassi	OTU2475
d__Bacteri	k__norank_	p__Proteob	c__Gamma	o__Legione	f__Legione	g__Legione	s__unclassi	OTU2384
d__Bacteri	k__norank_	p__Proteob	c__Alphapr	o__Rhizobi	f__Beijerin	g__Methyl	s__uncultu	OTU1005
d__Bacteri	k__norank_	p__Chlamy	c__Chlamy	o__Chlamy	f__Parachl	g__unclass	s__unclassi	OTU1746
d__Bacteri	k__norank_	p__Firmicu	c__Clostrid	o__Clostrid	f__Rumino	g__Ruminc	s__unclassi	OTU1172
d__Bacteri	k__norank_	p__Firmicu	c__Clostrid	o__Clostrid	f__Clostridi	g__Clostrid	s__metage	OTU1452
d__Bacteri	k__norank_	p__Acidob	c__Acidoba	o__Solibac	f__Solibact	g__Candida	s__uncultu	OTU1706
d__Bacteri	k__norank_	p__Proteob	c__Alphapr	o__Paracae	f__Paracae	g__norank_	s__uncultu	OTU1998
d__Bacteri	k__norank_	p__Proteob	c__Deltapr	o__Bdellov	f__Bdellovi	g__OM27_	s__uncultu	OTU1684
d__Bacteri	k__norank_	p__Bacterc	c__Bactero	o__Chitino	f__Saprosp	g__norank_	s__unclassi	OTU2539
d__Bacteri	k__norank_	p__Proteob	c__Deltapr	o__MBNT1	f__norank_	g__norank_	s__unclassi	OTU2243
d__Bacteri	k__norank_	p__Firmicu	c__Bacilli	o__Bacillal	f__Paeniba	g__Cohnell	s__uncultu	OTU764
d__Bacteri	k__norank_	p__Proteob	c__Gamma	o__Betaprc	f__Rhodoc	g__Methyl	s__uncultu	OTU680
d__Bacteri	k__norank_	p__Bacterc	c__Ignavib	o__OPB56	f__norank_	g__norank_	s__unclassi	OTU1667
d__Bacteri	k__norank_	p__Proteob	c__Gamma	o__Methyl	f__Methylc	g__norank_	s__unclassi	OTU1870
d__Bacteri	k__norank_	p__Gemma	c__Gemma	o__Gemma	f__Gemma	g__Gemma	s__unclassi	OTU1065
d__Bacteri	k__norank_	p__Proteob	c__Gamma	o__Betaprc	f__Nitrosor	g__MND1	s__metage	OTU3575
d__Bacteri	k__norank_	p__Chlamy	c__Chlamy	o__Chlamy	f__Simkani	g__norank_	s__unclassi	OTU2619
d__Bacteri	k__norank_	p__Depenc	c__Babelia	o__Babelia	f__Vermipl	g__norank_	s__uncultu	OTU2495
d__Bacteri	k__norank_	p__Cyanob	c__Oxypho	o__Chlorop	f__norank_	g__norank_	s__unclassi	OTU3448
d__Bacteri	k__norank_	p__Verrucc	c__Verrucc	o__Verrucc	f__Akkerm	g__Akkerm	s__uncultu	OTU1173
d__Bacteri	k__norank_	p__Depenc	c__Babelia	o__Babelia	f__UBA124	g__norank_	s__uncultu	OTU1830
d__Bacteri	k__norank_	p__Depenc	c__Babelia	o__Babelia	f__unclassi	g__unclass	s__unclassi	OTU2820
d__Bacteri	k__norank_	p__Proteob	c__Gamma	o__Pseudo	f__Moraxe	g__Acineto	s__unclassi	OTU1823
d__Bacteri	k__norank_	p__Cyanob	c__Oxypho	o__Chlorop	f__norank_	g__norank_	s__metage	OTU1604
d__Bacteri	k__norank_	p__Depenc	c__Babelia	o__Babelia	f__Babelia	g__norank_	s__uncultu	OTU2593
d__Bacteri	k__norank_	p__Depenc	c__Babelia	o__Babelia	f__Vermipl	g__norank_	s__uncultu	OTU2254
d__Bacteri	k__norank_	p__Firmicu	c__Clostrid	o__Clostrid	f__Rumino	g__Rumini	s__unclassi	OTU1012
d__Bacteri	k__norank_	p__Proteob	c__Alphapr	o__Acetob	f__Acetoba	g__Acidiph	s__unclassi	OTU992
d__Bacteri	k__norank_	p__Actinob	c__Actinob	o__Microtr	f__Microtri	g__IMCC26	s__unclassi	OTU1435
d__Bacteri	k__norank_	p__Acidob	c__Subgrou	o__norank_	f__norank_	g__norank_	s__unclassi	OTU1257
d__Bacteri	k__norank_	p__Proteob	c__Gamma	o__unclass	f__unclassi	g__unclass	s__unclassi	OTU2610
d__Bacteri	k__norank_	p__Chlamy	c__Chlamy	o__Chlamy	f__cvE6	g__norank_	s__unclassi	OTU1666
d__Bacteri	k__norank_	p__Bacterc	c__Bactero	o__Flavoba	f__Flavoba	g__Flavoba	s__Flavoba	OTU309
d__Bacteri	k__norank_	p__Proteob	c__Gamma	o__Betaprc	f__Nitrosor	g__Ellin606	s__uncultu	OTU2642
d__Bacteri	k__norank_	p__Depenc	c__Babelia	o__Babelia	f__Vermipl	g__norank_	s__uncultu	OTU2312
d__Bacteri	k__norank_	p__Plancto	c__Phycisp	o__Phycisp	f__Phycispl	g__AKYG58	s__uncultu	OTU1763
d__Bacteri	k__norank_	p__Proteob	c__Gamma	o__Betaprc	f__Burkhol	g__Lautrop	s__uncultu	OTU2218

d__Bacteri	k__norank_	p__Proteob	c__Gamma	o__Diploric	f__Diploric	g__Aquicel	s__uncultu	OTU1985
d__Bacteri	k__norank_	p__Proteob	c__Gamma	o__unclass	f__unclassi	g__unclass	s__unclassi	OTU1664
d__Bacteri	k__norank_	p__Proteob	c__Deltapr	o__Myxocc	f__Polyang	g__Pajaroe	s__uncultu	OTU1670
d__Bacteri	k__norank_	p__Proteob	c__Deltapr	o__unclass	f__unclassi	g__unclass	s__unclassi	OTU2351
d__Bacteri	k__norank_	p__Depend	c__Babelia	o__Babelia	f__Vermiph	g__norank_	s__uncultu	OTU2671
d__Bacteri	k__norank_	p__Bacterc	c__Bactero	o__Sphingc	f__Sphingo	g__unclass	s__unclassi	OTU1082
d__Bacteri	k__norank_	p__Proteob	c__Gamma	o__Legione	f__Legione	g__Legione	s__unclassi	OTU2722
d__Bacteri	k__norank_	p__Proteob	c__Alphapr	o__Sphingc	f__Sphingo	g__Sphingc	s__uncultu	OTU197
d__Bacteri	k__norank_	p__Depend	c__Babelia	o__Babelia	f__Vermiph	g__norank_	s__uncultu	OTU2674
d__Bacteri	k__norank_	p__Actinob	c__Actinob	o__Micrococ	f__Microba	g__Rathayi	s__Rathayi	OTU1440
d__Bacteri	k__norank_	p__Proteob	c__Gamma	o__Steroid	f__Steroidc	g__Steroid	s__uncultu	OTU2565
d__Bacteri	k__norank_	p__Cyanob	c__Oxypho	o__Synech	f__Cyanobi	g__Synech	s__unident	OTU1936
d__Bacteri	k__norank_	p__Chlamy	c__Chlamy	o__Chlamy	f__cvE6	g__norank_	s__metage	OTU2070
d__Bacteri	k__norank_	p__Proteob	c__Gamma	o__Xantho	f__Xanthor	g__Lysobac	s__unclassi	OTU2665
d__Bacteri	k__norank_	p__Plancto	c__Plancto	o__Isospha	f__Isospha	g__norank_	s__uncultu	OTU2146
d__Bacteri	k__norank_	p__Firmicu	c__Negativ	o__Selenor	f__Veillone	g__Megasp	s__unclassi	OTU899
d__Bacteri	k__norank_	p__Proteob	c__Gamma	o__Diploric	f__Diploric	g__Aquicel	s__unclassi	OTU1820
d__Bacteri	k__norank_	p__Proteob	c__Gamma	o__Legione	f__Legione	g__Legione	s__uncultu	OTU1272
d__Bacteri	k__norank_	p__Chlorof	c__KD4-96	o__norank_	f__norank_	g__norank_	s__metage	OTU2096
d__Bacteri	k__norank_	p__Patescil	c__Microge	o__norank_	f__norank_	g__norank_	s__uncultu	OTU2172
d__Bacteri	k__norank_	p__Bacterc	c__Bactero	o__Bacterc	f__Prevote	g__Prevote	s__Prevote	OTU1362
d__Bacteri	k__norank_	p__Actinob	c__Actinob	o__Corynel	f__Mycoba	g__Mycoba	s__unclassi	OTU1981
d__Bacteri	k__norank_	p__Cyanob	c__Melaina	o__Gastrar	f__norank_	g__norank_	s__uncultu	OTU1076
d__Bacteri	k__norank_	p__Depend	c__Babelia	o__Babelia	f__Vermiph	g__norank_	s__uncultu	OTU98
d__Bacteri	k__norank_	p__Verrucc	c__Verrucc	o__Verrucc	f__Akkerm	g__Akkerm	s__unclassi	OTU2627
d__Bacteri	k__norank_	p__Actinob	c__Actinob	o__IMCC26	f__norank_	g__norank_	s__uncultu	OTU1900
d__Bacteri	k__norank_	p__Firmicu	c__Negativ	o__Selenor	f__Veillone	g__Pelosin	s__uncultu	OTU1270
d__Bacteri	k__norank_	p__Bacterc	c__Bactero	o__Cytoph	f__Spirosoı	g__Emticici	s__uncultu	OTU449
d__Bacteri	k__norank_	p__Firmicu	c__Bacilli	o__Bacillal	f__Paeniba	g__Breviba	s__unclassi	OTU1522
d__Bacteri	k__norank_	p__Proteob	c__Deltapr	o__Myxocc	f__Haliangi	g__Haliang	s__uncultu	OTU2130
d__Bacteri	k__norank_	p__Firmicu	c__Bacilli	o__Bacillal	f__Paeniba	g__Paeniba	s__Paeniba	OTU831
d__Bacteri	k__norank_	p__Proteob	c__Gamma	o__Diploric	f__Diploric	g__Aquicel	s__uncultu	OTU2581
d__Bacteri	k__norank_	p__Actinob	c__Actinob	o__Pseudo	f__Pseudor	g__Pseudo	s__Pseudo	OTU1011
d__Bacteri	k__norank_	p__Firmicu	c__Clostrid	o__Clostrid	f__Rumino	g__unclass	s__unclassi	OTU2026
d__Bacteri	k__norank_	p__Depend	c__Babelia	o__Babelia	f__unclassi	g__unclass	s__unclassi	OTU2307
d__Bacteri	k__norank_	p__Firmicu	c__Bacilli	o__Bacillal	f__Thermo	g__Thermc	s__unclassi	OTU1279
d__Bacteri	k__norank_	p__Firmicu	c__Negativ	o__Selenor	f__Veillone	g__Pelosin	s__uncultu	OTU1404
d__Bacteri	k__norank_	p__Bacterc	c__Bactero	o__Sphingc	f__Sphingo	g__Pedoba	s__unclassi	OTU950
d__Bacteri	k__norank_	p__Firmicu	c__Clostrid	o__Clostrid	f__Caldicop	g__Caldico	s__unclassi	OTU1175
d__Bacteri	k__norank_	p__Proteob	c__Gamma	o__Betapr	f__Rhodoc	g__norank_	s__unclassi	OTU1242
d__Bacteri	k__norank_	p__Proteob	c__Gamma	o__Xantho	f__Rhodan	g__Dokdor	s__uncultu	OTU164
d__Bacteri	k__norank_	p__Bacterc	c__Bactero	o__Bacterc	f__Bactero	g__Bactero	s__Bactero	OTU1821
d__Bacteri	k__norank_	p__Proteob	c__Gamma	o__Betapr	f__Burkhol	g__Polaron	s__unclassi	OTU293
d__Bacteri	k__norank_	p__Chlorof	c__Anaerol	o__RBG-13	f__norank_	g__norank_	s__unclassi	OTU1805
d__Bacteri	k__norank_	p__Bacterc	c__Bactero	o__Cytoph	f__Spirosoı	g__Runella	s__Runella	OTU1078
d__Bacteri	k__norank_	p__Gemma	c__Gemma	o__Gemma	f__Gemma	g__norank_	s__metage	OTU3505
d__Bacteri	k__norank_	p__Firmicu	c__Erysipel	o__Erysipe	f__Erysipel	g__Erysipel	s__uncultu	OTU105

d__Bacteri	k__norank_	p__Firmicu	c__Clostrid	o__Clostrid	f__Clostridi	g__Oxobac	s__unclassi	OTU1097
d__Bacteri	k__norank_	p__Proteol	c__Gamma	o__Enterol	f__Enterob	g__Plesiom	s__Plesiom	OTU1206
d__Bacteri	k__norank_	p__Firmicu	c__Clostrid	o__Clostrid	f__Clostridi	g__Fontice	s__unclassi	OTU1180
d__Bacteri	k__norank_	p__Proteol	c__Alphapr	o__Acetob	f__Acetoba	g__Roseom	s__uncultu	OTU183
d__Bacteri	k__norank_	p__Cyanob	c__Oxypho	o__Chlorop	f__norank_	g__norank_	s__unclassi	OTU1741
d__Bacteri	k__norank_	p__Bacterc	c__Bactero	o__Flavoba	f__Flavoba	g__Flavoba	s__Flavoba	OTU1128
d__Bacteri	k__norank_	p__Bacterc	c__Bactero	o__Flavoba	f__Flavoba	g__Flavoba	s__unclassi	OTU3533
d__Bacteri	k__norank_	p__Proteol	c__Gamma	o__Betaprc	f__Burkhol	g__Polaron	s__unclassi	OTU3652
d__Bacteri	k__norank_	p__unclass	c__unclassi	o__unclass	f__unclassi	g__unclass	s__unclassi	OTU2143
d__Bacteri	k__norank_	p__Proteol	c__Alphapr	o__Sphingc	f__Sphingo	g__Sandara	s__uncultu	OTU1367
d__Bacteri	k__norank_	p__Chlorof	c__Chlorof	o__Thermc	f__JG30-KF	g__norank_	s__bacteriu	OTU152
d__Bacteri	k__norank_	p__Proteol	c__Deltapr	o__unclass	f__unclassi	g__unclass	s__unclassi	OTU2624
d__Bacteri	k__norank_	p__Proteol	c__Gamma	o__Diploric	f__Diploric	g__norank_	s__uncultu	OTU1875
d__Bacteri	k__norank_	p__Fibroba	c__Fibroba	o__Fibroba	f__Fibroba	g__norank_	s__uncultu	OTU2032
d__Bacteri	k__norank_	p__Proteol	c__Gamma	o__Legione	f__Legione	g__Legione	s__uncultu	OTU2659
d__Bacteri	k__norank_	p__Chlamy	c__Chlamy	o__Chlamy	f__Simkani	g__Candida	s__unclassi	OTU2751
d__Bacteri	k__norank_	p__Bacterc	c__Bactero	o__Cytoph	f__Hymenc	g__Adhaeri	s__uncultu	OTU1019
d__Bacteri	k__norank_	p__Proteol	c__Alphapr	o__Rhizobi	f__Hyphom	g__unclass	s__unclassi	OTU178
d__Bacteri	k__norank_	p__Acidoba	c__Subgrou	o__norank_	f__norank_	g__norank_	s__uncultu	OTU1428
d__Bacteri	k__norank_	p__Fibroba	c__Chitiniv	o__norank_	f__norank_	g__norank_	s__uncultu	OTU1847
d__Bacteri	k__norank_	p__Proteol	c__Gamma	o__Pseudo	f__Moraxe	g__Agitoco	s__metage	OTU2375
d__Bacteri	k__norank_	p__Proteol	c__Deltapr	o__Myxocc	f__Haliangi	g__Haliang	s__unclassi	OTU2849
d__Bacteri	k__norank_	p__unclass	c__unclassi	o__unclass	f__unclassi	g__unclass	s__unclassi	OTU1941
d__Bacteri	k__norank_	p__Bacterc	c__Bactero	o__Bacterc	f__Bactero	g__Bactero	s__Bactero	OTU2526
d__Bacteri	k__norank_	p__Depenc	c__Babelia	o__Babelia	f__unclassi	g__unclass	s__unclassi	OTU2129
d__Bacteri	k__norank_	p__Proteol	c__Alphapr	o__Reyran	f__Reyrane	g__Reyrane	s__unclassi	OTU455
d__Bacteri	k__norank_	p__Bacterc	c__Bactero	o__Flavoba	f__Flavoba	g__Flavoba	s__unclassi	OTU1124
d__Bacteri	k__norank_	p__Depenc	c__Babelia	o__Babelia	f__unclassi	g__unclass	s__unclassi	OTU1848
d__Bacteri	k__norank_	p__Depenc	c__Babelia	o__Babelia	f__Vermipl	g__norank_	s__uncultu	OTU1856
d__Bacteri	k__norank_	p__Proteol	c__Gamma	o__unclass	f__unclassi	g__unclass	s__unclassi	OTU2792
d__Bacteri	k__norank_	p__Proteol	c__Gamma	o__Legione	f__Legione	g__Legione	s__unclassi	OTU1853
d__Bacteri	k__norank_	p__Proteol	c__Alphapr	o__Cauloba	f__Cauloba	g__Brevun	s__metage	OTU198
d__Bacteri	k__norank_	p__Plancto	c__BD7-11	o__norank_	f__norank_	g__norank_	s__uncultu	OTU2805
d__Bacteri	k__norank_	p__Proteol	c__Alphapr	o__Rhizobi	f__KF-JG30	g__norank_	s__uncultu	OTU4034
d__Bacteri	k__norank_	p__Proteol	c__Gamma	o__Betaprc	f__Neisseri	g__Neisser	s__Neisseri	OTU1237
d__Bacteri	k__norank_	p__Cyanob	c__Oxypho	o__Chlorop	f__norank_	g__norank_	s__Phalacr	OTU1029
d__Bacteri	k__norank_	p__Proteol	c__Gamma	o__SAR86_	f__norank_	g__norank_	s__unclassi	OTU2095
d__Bacteri	k__norank_	p__Bacterc	c__Bactero	o__Chitino	f__Saprosp	g__norank_	s__uncultu	OTU71
d__Bacteri	k__norank_	p__Proteol	c__Gamma	o__Legione	f__Legione	g__Legione	s__unclassi	OTU2711
d__Bacteri	k__norank_	p__Bacterc	c__Bactero	o__Bacterc	f__Muribac	g__norank_	s__unclassi	OTU137
d__Bacteri	k__norank_	p__Depenc	c__Babelia	o__Babelia	f__Vermipl	g__norank_	s__uncultu	OTU2661
d__Bacteri	k__norank_	p__Proteol	c__Deltapr	o__Oligofle	f__Oligofle	g__norank_	s__unclassi	OTU2716
d__Bacteri	k__norank_	p__Proteol	c__Gamma	o__Diploric	f__Diploric	g__Aquicel	s__uncultu	OTU2830
d__Bacteri	k__norank_	p__Proteol	c__Gamma	o__Betaprc	f__Burkhol	g__norank_	s__Alcalige	OTU1400
d__Bacteri	k__norank_	p__Proteol	c__Gamma	o__unclass	f__unclassi	g__unclass	s__unclassi	OTU2263
d__Bacteri	k__norank_	p__Firmicu	c__Clostrid	o__Clostrid	f__Lachnos	g__Lachno	s__uncultu	OTU42
d__Bacteri	k__norank_	p__Proteol	c__Gamma	o__Diploric	f__Diploric	g__norank_	s__metage	OTU2681

d__Bacteria	k__norank_	p__Depend	c__Babelia	o__Babelia	f__unclassi	g__unclass	s__unclassi	OTU1949
d__Bacteria	k__norank_	p__Proteob	c__Alphapr	o__norank_	f__norank_	g__norank_	s__unclassi	OTU2015
d__Bacteria	k__norank_	p__Proteob	c__Alphapr	o__Rhizobi	f__A0839	g__norank_	s__uncultu	OTU156
d__Bacteria	k__norank_	p__Bacterc	c__Bactero	o__Bacterc	f__Dysgonc	g__norank_	s__uncultu	OTU878
d__Bacteria	k__norank_	p__Bacterc	c__Bactero	o__Flavoba	f__Flavoba	g__Flavoba	s__unclassi	OTU3311
d__Bacteria	k__norank_	p__Verrucc	c__Verrucc	o__Pedosp	f__Pedosph	g__norank_	s__unclassi	OTU3150
d__Bacteria	k__norank_	p__Bacterc	c__Bactero	o__Cytopha	f__Hymenc	g__Hymen	s__unclassi	OTU2493
d__Bacteria	k__norank_	p__Proteob	c__Alphapr	o__Rhizobi	f__Amb-16	g__norank_	s__uncultu	OTU4120
d__Bacteria	k__norank_	p__Chlamy	c__Chlamy	o__Chlamy	f__cvE6	g__norank_	s__metage	OTU1696
d__Bacteria	k__norank_	p__Depend	c__Babelia	o__Babelia	f__Vermiph	g__norank_	s__uncultu	OTU2065
d__Bacteria	k__norank_	p__Firmicu	c__Clostrid	o__Clostrid	f__Peptocc	g__Peloton	s__uncultu	OTU1106
d__Bacteria	k__norank_	p__Proteob	c__Gamma	o__unclass	f__unclassi	g__unclass	s__unclassi	OTU2649
d__Bacteria	k__norank_	p__Depend	c__Babelia	o__Babelia	f__unclassi	g__unclass	s__unclassi	OTU2498
d__Bacteria	k__norank_	p__Firmicu	c__Clostrid	o__Clostrid	f__Rumino	g__Ruminc	s__metage	OTU1902
d__Bacteria	k__norank_	p__Proteob	c__Gamma	o__unclass	f__unclassi	g__unclass	s__unclassi	OTU2078
d__Bacteria	k__norank_	p__Proteob	c__Gamma	o__Betaprc	f__Rhodocy	g__Azoarcu	s__unclassi	OTU3252
d__Bacteria	k__norank_	p__Bacterc	c__Bactero	o__Bacterc	f__Prevote	g__unclass	s__unclassi	OTU2306
d__Bacteria	k__norank_	p__Proteob	c__Gamma	o__Diploric	f__Diploric	g__Aquicel	s__uncultu	OTU2715
d__Bacteria	k__norank_	p__Proteob	c__Gamma	o__Betaprc	f__Rhodocy	g__Candida	s__unclassi	OTU755
d__Bacteria	k__norank_	p__Proteob	c__Gamma	o__Pseudo	f__Moraxe	g__Enhydrc	s__Moraxe	OTU53
d__Bacteria	k__norank_	p__Proteob	c__Gamma	o__Legione	f__Legione	g__Legione	s__unclassi	OTU2662
d__Bacteria	k__norank_	p__Proteob	c__Gamma	o__Diploric	f__Diploric	g__Aquicel	s__uncultu	OTU2719
d__Bacteria	k__norank_	p__Proteob	c__Gamma	o__Diploric	f__Diploric	g__norank_	s__uncultu	OTU1980
d__Bacteria	k__norank_	p__Proteob	c__Deltapr	o__Bdellov	f__Bacteric	g__Peredib	s__unclassi	OTU664
d__Bacteria	k__norank_	p__Proteob	c__Alphapr	o__Caulob	f__Cauloba	g__unclass	s__unclassi	OTU479
d__Bacteria	k__norank_	p__Proteob	c__Gamma	o__Diploric	f__Diploric	g__Aquicel	s__unclassi	OTU2796
d__Bacteria	k__norank_	p__unclass	c__unclassi	o__unclass	f__unclassi	g__unclass	s__unclassi	OTU1393
d__Bacteria	k__norank_	p__Proteob	c__Deltapr	o__Desulfc	f__Desulfol	g__norank_	s__unclassi	OTU569
d__Bacteria	k__norank_	p__Patescil	c__Gracilib	o__norank_	f__norank_	g__norank_	s__metage	OTU2075
d__Bacteria	k__norank_	p__Firmicu	c__Negativ	o__Selenor	f__Veillone	g__Zymoph	s__Propion	OTU745
d__Bacteria	k__norank_	p__Proteob	c__Alphapr	o__Rhizobi	f__Beijerin	g__Microvi	s__unclassi	OTU35
d__Bacteria	k__norank_	p__Proteob	c__Alphapr	o__Sphingc	f__Sphingo	g__Blastor	s__uncultu	OTU1051
d__Bacteria	k__norank_	p__Bacterc	c__Bactero	o__Bacterc	f__Prevote	g__Prevote	s__uncultu	OTU1626
d__Bacteria	k__norank_	p__Bacterc	c__Bactero	o__Bacterc	f__Prevote	g__Prevote	s__unclassi	OTU1457
d__Bacteria	k__norank_	p__Proteob	c__Gamma	o__unclass	f__unclassi	g__unclass	s__unclassi	OTU2003
d__Bacteria	k__norank_	p__Proteob	c__Gamma	o__Beggiat	f__Beggiat	g__norank_	s__unclassi	OTU2566
d__Bacteria	k__norank_	p__Proteob	c__Alphapr	o__unclass	f__unclassi	g__unclass	s__unclassi	OTU2194
d__Bacteria	k__norank_	p__Chlorof	c__Anaerol	o__SBR103	f__A4b	g__norank_	s__uncultu	OTU1174
d__Bacteria	k__norank_	p__Actinob	c__Actinob	o__Microtr	f__norank_	g__norank_	s__metage	OTU961
d__Bacteria	k__norank_	p__Proteob	c__Gamma	o__Betaprc	f__Burkhol	g__norank_	s__unclassi	OTU2418
d__Bacteria	k__norank_	p__Gemma	c__Gemma	o__Gemma	f__Gemma	g__norank_	s__unclassi	OTU860
d__Bacteria	k__norank_	p__Proteob	c__Deltapr	o__Sva048	f__norank_	g__norank_	s__uncultu	OTU2308
d__Bacteria	k__norank_	p__Proteob	c__Gamma	o__Betaprc	f__Burkhol	g__Limnoh	s__uncultu	OTU453
d__Bacteria	k__norank_	p__Lentisp	c__Lentispl	o__Victival	f__Victivall	g__norank_	s__uncultu	OTU1914
d__Bacteria	k__norank_	p__Proteob	c__Alphapr	o__Sphingc	f__Sphingo	g__Sphingc	s__Sphingc	OTU1365
d__Bacteria	k__norank_	p__Proteob	c__Gamma	o__Diploric	f__Diploric	g__Aquicel	s__unclassi	OTU2213
d__Bacteria	k__norank_	p__Bacterc	c__Bactero	o__Chitino	f__Saprosp	g__norank_	s__metage	OTU1131

d__Bacteri	k__norank_	p__Verruco	c__Verruco	o__Verruco	f__Rubrital	g__Luteolib	s__uncultu	OTU1263
d__Bacteri	k__norank_	p__Chlorof	c__Ktedon	o__Ktedon	f__JG30-KF	g__norank_	s__uncultu	OTU1993
d__Bacteri	k__norank_	p__Depend	c__Babelia	o__Babelia	f__Babelia	g__norank_	s__uncultu	OTU2457
d__Bacteri	k__norank_	p__Firmicu	c__Clostrid	o__Clostrid	f__Christen	g__norank_	s__unclassi	OTU1337
d__Bacteri	k__norank_	p__Chlorof	c__Anaerol	o__SBR103	f__A4b	g__norank_	s__uncultu	OTU2723
d__Bacteri	k__norank_	p__Actinob	c__Actinob	o__Corynel	f__Nocardi	g__Gordon	s__Gordon	OTU1551
d__Bacteri	k__norank_	p__Proteob	c__Alphapr	o__Acetob	f__Acetoba	g__Rhodov	s__uncultu	OTU557
d__Bacteri	k__norank_	p__Depend	c__Babelia	o__Babelia	f__Vermipl	g__norank_	s__uncultu	OTU2488
d__Bacteri	k__norank_	p__Proteob	c__Gamma	o__Betapro	f__Burkhol	g__unclass	s__unclassi	OTU1436
d__Bacteri	k__norank_	p__Chlamy	c__Chlamy	o__Chlamy	f__Parachla	g__Neochla	s__unclassi	OTU2471
d__Bacteri	k__norank_	p__Acidoba	c__Subgrou	o__norank_	f__norank_	g__norank_	s__unclassi	OTU2631
d__Bacteri	k__norank_	p__Proteob	c__Gamma	o__Cardiob	f__Wohlfah	g__Ignatzsc	s__Ignatzsc	OTU1712
d__Bacteri	k__norank_	p__Firmicu	c__Bacilli	o__Bacillal	f__Bacillace	g__Geobac	s__Geobac	OTU1682
d__Bacteri	k__norank_	p__Plancto	c__Plancto	o__Gemma	f__Gemma	g__norank_	s__uncultu	OTU1636
d__Bacteri	k__norank_	p__Firmicu	c__Clostrid	o__Clostrid	f__Lachnos	g__Acetitor	s__uncultu	OTU1946
d__Bacteri	k__norank_	p__Latescib	c__Latescib	o__norank_	f__norank_	g__norank_	s__unclassi	OTU2170
d__Bacteri	k__norank_	p__Proteob	c__Alphapr	o__Acetob	f__Acetoba	g__Roseom	s__Roseom	OTU538
d__Bacteri	k__norank_	p__Proteob	c__Gamma	o__Betapro	f__Rhodocy	g__unclass	s__unclassi	OTU2595
d__Bacteri	k__norank_	p__Proteob	c__Alphapr	o__unclass	f__unclassi	g__unclass	s__unclassi	OTU2110
d__Bacteri	k__norank_	p__Proteob	c__Gamma	o__Betapro	f__TRA3-20	g__norank_	s__metage	OTU3651
d__Bacteri	k__norank_	p__Proteob	c__Gamma	o__Betapro	f__TRA3-20	g__norank_	s__metage	OTU3650
d__Bacteri	k__norank_	p__Proteob	c__Gamma	o__Betapro	f__Nitrosor	g__mle1-7	s__uncultu	OTU2297
d__Bacteri	k__norank_	p__Proteob	c__Gamma	o__Xantho	f__Rhodan	g__unclass	s__unclassi	OTU1315
d__Bacteri	k__norank_	p__Proteob	c__Gamma	o__Diploric	f__Diploric	g__Aquicel	s__unclassi	OTU2531
d__Bacteri	k__norank_	p__Proteob	c__Gamma	o__Betapro	f__Burkhol	g__unclass	s__unclassi	OTU3879
d__Bacteri	k__norank_	p__Proteob	c__Gamma	o__Betapro	f__Methylo	g__norank_	s__uncultu	OTU3635
d__Bacteri	k__norank_	p__Proteob	c__Gamma	o__Diploric	f__Diploric	g__Aquicel	s__unclassi	OTU548
d__Bacteri	k__norank_	p__Actinob	c__Actinob	o__Microtr	f__Ilumato	g__CL500-2	s__unclassi	OTU2677
d__Bacteri	k__norank_	p__Bactero	c__Bactero	o__Chitino	f__Chitinop	g__Terrimo	s__unclassi	OTU2698
d__Bacteri	k__norank_	p__Firmicu	c__Clostrid	o__Clostrid	f__Peptoco	g__Thermin	s__uncultu	OTU1158
d__Bacteri	k__norank_	p__Actinob	c__Actinob	o__Microtr	f__Ilumato	g__CL500-2	s__unclassi	OTU3279
d__Bacteri	k__norank_	p__Bactero	c__Bactero	o__Flavoba	f__Flavoba	g__unclass	s__unclassi	OTU1727
d__Bacteri	k__norank_	p__Firmicu	c__Clostrid	o__Clostrid	f__Family_	g__norank_	s__unclassi	OTU1183
d__Bacteri	k__norank_	p__Chlorof	c__Anaerol	o__Anaero	f__Anaerol	g__norank_	s__uncultu	OTU2177
d__Bacteri	k__norank_	p__Proteob	c__Gamma	o__PLTA13	f__norank_	g__norank_	s__unclassi	OTU986
d__Bacteri	k__norank_	p__Spiroch	c__Leptosp	o__Leptosp	f__Leptosp	g__Leptosp	s__Leptosp	OTU1160
d__Bacteri	k__norank_	p__Chlorof	c__Dehaloc	o__S085	f__norank_	g__norank_	s__uncultu	OTU1905
d__Bacteri	k__norank_	p__Depend	c__Babelia	o__Babelia	f__unclassi	g__unclass	s__unclassi	OTU2152
d__Bacteri	k__norank_	p__Bactero	c__Bactero	o__Bactero	f__Tannere	g__Parabac	s__Parabac	OTU2625
d__Bacteri	k__norank_	p__Bactero	c__Rhodotl	o__Rhodot	f__Rhodoth	g__norank_	s__uncultu	OTU2286
d__Bacteri	k__norank_	p__Cyanob	c__Melaina	o__Gastran	f__norank_	g__norank_	s__uncultu	OTU1200
d__Bacteri	k__norank_	p__Acidoba	c__Subgrou	o__norank_	f__norank_	g__norank_	s__unclassi	OTU4031
d__Bacteri	k__norank_	p__Proteob	c__Deltapr	o__Oligofle	f__Oligofle	g__norank_	s__unclassi	OTU3599
d__Bacteri	k__norank_	p__Actinob	c__Actinob	o__Microtr	f__Ilumato	g__CL500-2	s__uncultu	OTU1992
d__Bacteri	k__norank_	p__Proteob	c__Gamma	o__Betapro	f__SC-I-84	g__norank_	s__unclassi	OTU2064
d__Bacteri	k__norank_	p__Acidoba	c__Subgrou	o__unclass	f__unclassi	g__Luteital	s__uncultu	OTU1199
d__Bacteri	k__norank_	p__Proteob	c__Gamma	o__Gamma	f__unclassi	g__Acidiba	s__unclassi	OTU2694

d__Bacteri	k__norank_	p__Firmicu	c__Bacilli	o__Bacillal	f__Thermo	g__Laceyel	s__Laceyell	OTU1329
d__Bacteri	k__norank_	p__Chlamy	c__Chlamy	o__Chlamy	f__Parachl	g__Neochl	s__metage	OTU2660
d__Bacteri	k__norank_	p__Firmicu	c__Clostrid	o__Clostrid	f__Rumino	g__Rumino	s__uncultu	OTU2102
d__Bacteri	k__norank_	p__Chlorof	c__Chlorof	o__Thermo	f__JG30-KF	g__norank_	s__uncultu	OTU1785
d__Bacteri	k__norank_	p__Depend	c__Babelia	o__Babelia	f__Vermipl	g__norank_	s__unclassi	OTU2853
d__Bacteri	k__norank_	p__Bacterc	c__Bactero	o__Flavoba	f__Flavoba	g__Flavoba	s__Flavoba	OTU1459
d__Bacteri	k__norank_	p__Chlorof	c__KD4-96	o__norank_	f__norank_	g__norank_	s__unclassi	OTU2158
d__Bacteri	k__norank_	p__Depend	c__Babelia	o__Babelia	f__Vermipl	g__norank_	s__unclassi	OTU2678
d__Bacteri	k__norank_	p__Actinob	c__Actinob	o__Strepto	f__Strepto	g__Strepto	s__Strepto	OTU26
d__Bacteri	k__norank_	p__Actinob	c__Actinob	o__norank_	f__norank_	g__norank_	s__uncultu	OTU2881
d__Bacteri	k__norank_	p__Chlamy	c__Chlamy	o__Chlamy	f__Parachl	g__Candida	s__metage	OTU3771
d__Bacteri	k__norank_	p__Proteob	c__Gamma	o__Legione	f__Legione	g__Legione	s__uncultu	OTU1797
d__Bacteri	k__norank_	p__Proteob	c__Alphapr	o__Rhizobi	f__Rhizobia	g__unclass	s__unclassi	OTU702
d__Bacteri	k__norank_	p__Depend	c__Babelia	o__Babelia	f__Vermipl	g__norank_	s__uncultu	OTU1756
d__Bacteri	k__norank_	p__Proteob	c__Gamma	o__Betapro	f__Burkhol	g__Limnoh	s__uncultu	OTU2756
d__Bacteri	k__norank_	p__Bacterc	c__Bactero	o__Flavoba	f__Crocinit	g__Fluviico	s__uncultu	OTU1364
d__Bacteri	k__norank_	p__Chlamy	c__Chlamy	o__Chlamy	f__Parachl	g__norank_	s__unclassi	OTU1731
d__Bacteri	k__norank_	p__Chlorof	c__Gitt-GS-	o__norank_	f__norank_	g__norank_	s__uncultu	OTU1554
d__Bacteri	k__norank_	p__Proteob	c__unclassi	o__unclass	f__unclassi	g__unclass	s__unclassi	OTU2403
d__Bacteri	k__norank_	p__Depend	c__Babelia	o__Babelia	f__unclassi	g__unclass	s__unclassi	OTU2544
d__Bacteri	k__norank_	p__Actinob	c__Actinob	o__Microco	f__Microcc	g__Arthrob	s__Arthrob	OTU1059
d__Bacteri	k__norank_	p__Depend	c__Babelia	o__Babelia	f__Vermipl	g__norank_	s__uncultu	OTU2255
d__Bacteri	k__norank_	p__Proteob	c__Deltapr	o__Myxoco	f__P3OB-4	g__norank_	s__unclassi	OTU2365
d__Bacteri	k__norank_	p__Proteob	c__Alphapr	o__Rhodos	f__norank_	g__norank_	s__unident	OTU19
d__Bacteri	k__norank_	p__Firmicu	c__Clostrid	o__Clostrid	f__Lachnos	g__Oribact	s__Oribact	OTU1339
d__Bacteri	k__norank_	p__Bacterc	c__Bactero	o__Flavoba	f__Flavoba	g__NS4_m	s__unclassi	OTU2274
d__Bacteri	k__norank_	p__Proteob	c__Alphapr	o__Azospir	f__Azospiri	g__Niveisp	s__uncultu	OTU1565
d__Bacteri	k__norank_	p__Proteob	c__Gamma	o__Legione	f__Legione	g__Legione	s__unclassi	OTU2441
d__Bacteri	k__norank_	p__Armatir	c__Armatir	o__Armatir	f__norank_	g__norank_	s__uncultu	OTU1344
d__Bacteri	k__norank_	p__Depend	c__Babelia	o__Babelia	f__UBA124	g__norank_	s__uncultu	OTU2546
d__Bacteri	k__norank_	p__Proteob	c__Alphapr	o__Caulob	f__Cauloba	g__unclass	s__unclassi	OTU62
d__Bacteri	k__norank_	p__Depend	c__Babelia	o__Babelia	f__unclassi	g__unclass	s__unclassi	OTU2317
d__Bacteri	k__norank_	p__Proteob	c__Alphapr	o__Sphingo	f__Sphingo	g__Sphingo	s__uncultu	OTU468
d__Bacteri	k__norank_	p__Proteob	c__Gamma	o__Enterob	f__Enterob	g__Enterob	s__unclassi	OTU109
d__Bacteri	k__norank_	p__Depend	c__Babelia	o__Babelia	f__Babelia	g__norank_	s__uncultu	OTU2506
d__Bacteri	k__norank_	p__Chlamy	c__Chlamy	o__Chlamy	f__Parachl	g__Neochl	s__uncultu	OTU2823
d__Bacteri	k__norank_	p__Depend	c__Babelia	o__Babelia	f__Vermipl	g__norank_	s__uncultu	OTU1703
d__Bacteri	k__norank_	p__Firmicu	c__Bacilli	o__Bacillal	f__Paeniba	g__Paeniba	s__unclassi	OTU1042
d__Bacteri	k__norank_	p__Depend	c__Babelia	o__Babelia	f__Vermipl	g__norank_	s__uncultu	OTU2689
d__Bacteri	k__norank_	p__Firmicu	c__Bacilli	o__Bacillal	f__Paeniba	g__Paeniba	s__unclassi	OTU1044
d__Bacteri	k__norank_	p__Actinob	c__Actinob	o__unclass	f__unclassi	g__unclass	s__unclassi	OTU1481
d__Bacteri	k__norank_	p__Proteob	c__Gamma	o__Coxiella	f__Coxiella	g__Coxiella	s__unclassi	OTU2484
d__Bacteri	k__norank_	p__Firmicu	c__Clostrid	o__Clostrid	f__Lachnos	g__Agathol	s__metage	OTU2343
d__Bacteri	k__norank_	p__Proteob	c__Deltapr	o__Bdellov	f__Bacteric	g__Peredib	s__unclassi	OTU2429
d__Bacteri	k__norank_	p__Firmicu	c__Clostrid	o__Clostrid	f__unclassi	g__unclass	s__unclassi	OTU1017
d__Bacteri	k__norank_	p__Firmicu	c__Bacilli	o__Bacillal	f__Paeniba	g__Paeniba	s__unclassi	OTU1282
d__Bacteri	k__norank_	p__Chlamy	c__Chlamy	o__Chlamy	f__Parachl	g__unclass	s__unclassi	OTU2445

d__Bacteri	k__norank_	p__Firmicu	c__Negativ	o__Selenor	f__Veillone	g__Veillone	s__unclassi	OTU99
d__Bacteri	k__norank_	p__Proteol	c__Gamma	o__Betapro	f__Burkhol	g__Aquaba	s__Aquaba	OTU739
d__Bacteri	k__norank_	p__Bacterc	c__Bactero	o__Bacterc	f__Rikenell	g__Alistipe	s__uncultu	OTU1689
d__Bacteri	k__norank_	p__Proteol	c__Gamma	o__Betapro	f__Rhodocy	g__norank_	s__unclassi	OTU1538
d__Bacteri	k__norank_	p__Proteol	c__Deltapr	o__Myxocc	f__Haliangi	g__Haliang	s__uncultu	OTU2416
d__Bacteri	k__norank_	p__Proteol	c__Gamma	o__Diploric	f__Diploric	g__Aquicel	s__uncultu	OTU559
d__Bacteri	k__norank_	p__Firmicu	c__Bacilli	o__Bacillal	f__Bacillac	g__Bacillus	s__unclassi	OTU1575
d__Bacteri	k__norank_	p__Proteol	c__Deltapr	o__Myxocc	f__Haliangi	g__Haliang	s__metage	OTU905
d__Bacteri	k__norank_	p__Proteol	c__Gamma	o__Legione	f__Legione	g__Legione	s__unclassi	OTU2285
d__Bacteri	k__norank_	p__Proteol	c__Alphapr	o__Reyran	f__Reyrane	g__Reyran	s__unclassi	OTU201
d__Bacteri	k__norank_	p__Acidob	c__Thermo	o__Thermc	f__Thermo	g__Subgro	s__uncultu	OTU2440
d__Bacteri	k__norank_	p__Firmicu	c__Clostrid	o__Clostrid	f__Clostridi	g__Clostrid	s__uncultu	OTU1545
d__Bacteri	k__norank_	p__Proteol	c__Gamma	o__Diploric	f__Diploric	g__Aquicel	s__unclassi	OTU1947
d__Bacteri	k__norank_	p__Actinol	c__Actinob	o__Corynel	f__Nocardi	g__Rhodoc	s__Rhodoc	OTU33
d__Bacteri	k__norank_	p__Proteol	c__Gamma	o__Xantho	f__Xanthor	g__Stenotr	s__Pseudo	OTU104
d__Bacteri	k__norank_	p__Proteol	c__Gamma	o__Diploric	f__Diploric	g__Aquicel	s__unclassi	OTU2185
d__Bacteri	k__norank_	p__Patescil	c__Sacchar	o__Sacchar	f__norank_	g__norank_	s__unclassi	OTU1196
d__Bacteri	k__norank_	p__Bacterc	c__Bactero	o__Bacterc	f__Prevote	g__Prevote	s__unclassi	OTU2356
d__Bacteri	k__norank_	p__Proteol	c__Gamma	o__Betapro	f__Burkhol	g__Rhodof	s__unclassi	OTU2673
d__Bacteri	k__norank_	p__Proteol	c__Alphapr	o__Rhizobi	f__Hyphon	g__Hyphon	s__metage	OTU370
d__Bacteri	k__norank_	p__Proteol	c__Gamma	o__Diploric	f__Diploric	g__Aquicel	s__unclassi	OTU2302
d__Bacteri	k__norank_	p__Firmicu	c__Bacilli	o__Bacillal	f__Paeniba	g__norank_	s__uncultu	OTU1293
d__Bacteri	k__norank_	p__Proteol	c__Deltapr	o__unclass	f__unclassi	g__unclass	s__unclassi	OTU1610
d__Bacteri	k__norank_	p__Proteol	c__Gamma	o__Diploric	f__Diploric	g__Aquicel	s__uncultu	OTU2233
d__Bacteri	k__norank_	p__Proteol	c__Gamma	o__Betapro	f__unclassi	g__unclass	s__unclassi	OTU1594
d__Bacteri	k__norank_	p__Chlorof	c__Anaerol	o__SBR103	f__A4b	g__norank_	s__uncultu	OTU483
d__Bacteri	k__norank_	p__Chlamy	c__Chlamy	o__Chlamy	f__unclassi	g__unclass	s__unclassi	OTU2381
d__Bacteri	k__norank_	p__Depenc	c__Babelia	o__Babelia	f__unclassi	g__unclass	s__unclassi	OTU2809
d__Bacteri	k__norank_	p__Depenc	c__Babelia	o__Babelia	f__UBA124	g__norank_	s__uncultu	OTU1764
d__Bacteri	k__norank_	p__Depenc	c__Babelia	o__Babelia	f__unclassi	g__unclass	s__unclassi	OTU1771
d__Bacteri	k__norank_	p__Depenc	c__Babelia	o__Babelia	f__norank_	g__norank_	s__unclassi	OTU2197
d__Bacteri	k__norank_	p__Proteol	c__Deltapr	o__Myxocc	f__BIrii41	g__norank_	s__metage	OTU604
d__Bacteri	k__norank_	p__Proteol	c__Gamma	o__Betapro	f__A21b	g__norank_	s__unclassi	OTU2377
d__Bacteri	k__norank_	p__Proteol	c__Alphapr	o__Rickett	f__Mitocho	g__norank_	s__unclassi	OTU1645
d__Bacteri	k__norank_	p__Firmicu	c__Clostrid	o__Clostrid	f__Rumino	g__Ruminic	s__unclassi	OTU1031
d__Bacteri	k__norank_	p__Chlorof	c__Chlorofl	o__Chlorof	f__Roseifle	g__norank_	s__freshwa	OTU2029
d__Bacteri	k__norank_	p__Epsilon	c__Campyl	o__Campyl	f__Arcobac	g__Arcobac	s__Arcobac	OTU1989
d__Bacteri	k__norank_	p__Chlamy	c__Chlamy	o__Chlamy	f__Simkani	g__norank_	s__unclassi	OTU2151
d__Bacteri	k__norank_	p__Gemma	c__Gemma	o__Gemma	f__Gemma	g__Gemma	s__Gemma	OTU3572
d__Bacteri	k__norank_	p__Bacterc	c__Ignavib	o__OPB56	f__norank_	g__norank_	s__unclassi	OTU1247
d__Bacteri	k__norank_	p__Chlamy	c__Chlamy	o__Chlamy	f__unclassi	g__unclass	s__unclassi	OTU1811
d__Bacteri	k__norank_	p__Patescil	c__Sacchar	o__Sacchar	f__Sacchar	g__norank_	s__uncultu	OTU1443
d__Bacteri	k__norank_	p__Chlamy	c__Chlamy	o__Chlamy	f__unclassi	g__unclass	s__unclassi	OTU2253
d__Bacteri	k__norank_	p__Firmicu	c__Clostrid	o__Clostrid	f__Lachnos	g__Epulopi	s__uncultu	OTU1533
d__Bacteri	k__norank_	p__Proteol	c__Gamma	o__Diploric	f__Diploric	g__Aquicel	s__uncultu	OTU1964
d__Bacteri	k__norank_	p__Proteol	c__Gamma	o__Diploric	f__Diploric	g__norank_	s__unclassi	OTU1885
d__Bacteri	k__norank_	p__Proteol	c__Gamma	o__Diploric	f__Diploric	g__Aquicel	s__uncultu	OTU2094

d__Bacteri	k__norank_	p__Actinob	c__Actinob	o__Propion	f__Nocardi	g__Nocardi	s__uncultu	OTU710
d__Bacteri	k__norank_	p__Proteob	c__Deltapr	o__Myxocc	f__Haliangi	g__Haliang	s__unclassi	OTU2340
d__Bacteri	k__norank_	p__Proteob	c__Gamma	o__Betaprc	f__Burkhol	g__Azohyd	s__uncultu	OTU891
d__Bacteri	k__norank_	p__Bacterc	c__Bactero	o__Sphingc	f__Sphingo	g__Pedoba	s__Pedoba	OTU1458
d__Bacteri	k__norank_	p__Acidoba	c__Subgrou	o__unclass	f__unclassi	g__unclass	s__unclassi	OTU2686
d__Bacteri	k__norank_	p__Bacterc	c__Bactero	o__Chitino	f__Chitinop	g__Sedimir	s__uncultu	OTU295
d__Bacteri	k__norank_	p__Depenc	c__Babelia	o__Babelia	f__UBA124	g__norank_	s__uncultu	OTU1962
d__Bacteri	k__norank_	p__Firmicu	c__Clostrid	o__Clostrid	f__Christer	g__Christer	s__uncultu	OTU1539
d__Bacteri	k__norank_	p__Chlorof	c__KD4-96	o__norank_	f__norank_	g__norank_	s__unclassi	OTU2082
d__Bacteri	k__norank_	p__Proteob	c__Alphapr	o__Ricketts	f__Mitocho	g__norank_	s__uncultu	OTU1678
d__Bacteri	k__norank_	p__Proteob	c__Gamma	o__unclass	f__unclassi	g__unclass	s__unclassi	OTU1898
d__Bacteri	k__norank_	p__Proteob	c__Gamma	o__Legione	f__Legione	g__Legione	s__uncultu	OTU2511
d__Bacteri	k__norank_	p__Depenc	c__Babelia	o__Babelia	f__unclassi	g__unclass	s__unclassi	OTU2378
d__Bacteri	k__norank_	p__Firmicu	c__Negativ	o__Selenor	f__Acidami	g__Succinic	s__uncultu	OTU1861
d__Bacteri	k__norank_	p__Proteob	c__Gamma	o__unclass	f__unclassi	g__unclass	s__unclassi	OTU1891
d__Bacteri	k__norank_	p__Bacterc	c__Bactero	o__Chitino	f__Chitinop	g__Ferrugir	s__uncultu	OTU1455
d__Bacteri	k__norank_	p__Firmicu	c__Clostrid	o__Clostrid	f__Eubacte	g__Acetoba	s__uncultu	OTU2742
d__Bacteri	k__norank_	p__Firmicu	c__Clostrid	o__Clostrid	f__Lachnos	g__norank_	s__unclassi	OTU1090
d__Bacteri	k__norank_	p__Proteob	c__Deltapr	o__Myxocc	f__mle1-27	g__norank_	s__unclassi	OTU1733
d__Bacteri	k__norank_	p__Bacterc	c__Bactero	o__Flavoba	f__Flavoba	g__Flavoba	s__unclassi	OTU1361
d__Bacteri	k__norank_	p__Proteob	c__Gamma	o__Oceanc	f__Halomo	g__Halomc	s__unclassi	OTU1222
d__Bacteri	k__norank_	p__Proteob	c__Gamma	o__Diploric	f__Diploric	g__norank_	s__unclassi	OTU2737
d__Bacteri	k__norank_	p__Proteob	c__Gamma	o__Alteron	f__Shewan	g__Shewar	s__Shewan	OTU2496
d__Bacteri	k__norank_	p__Proteob	c__Alphapr	o__Rhizobi	f__norank_	g__norank_	s__unclassi	OTU1972
d__Bacteri	k__norank_	p__Depenc	c__Babelia	o__Babelia	f__Vermipl	g__norank_	s__uncultu	OTU2145
d__Bacteri	k__norank_	p__unclass	c__unclassi	o__unclass	f__unclassi	g__unclass	s__unclassi	OTU1387
d__Bacteri	k__norank_	p__Gemma	c__Gemma	o__norank_	f__norank_	g__norank_	s__uncultu	OTU1680
d__Bacteri	k__norank_	p__Bacterc	c__Bactero	o__Cytoph	f__Spirosor	g__Lacihab	s__uncultu	OTU3045
d__Bacteri	k__norank_	p__Depenc	c__Babelia	o__Babelia	f__Vermipl	g__norank_	s__unclassi	OTU2691
d__Bacteri	k__norank_	p__Depenc	c__Babelia	o__Babelia	f__Vermipl	g__norank_	s__uncultu	OTU2494
d__Bacteri	k__norank_	p__unclass	c__unclassi	o__unclass	f__unclassi	g__unclass	s__unclassi	OTU1388
d__Bacteri	k__norank_	p__Depenc	c__Babelia	o__Babelia	f__Vermipl	g__norank_	s__unclassi	OTU2692
d__Bacteri	k__norank_	p__Depenc	c__Babelia	o__Babelia	f__unclassi	g__unclass	s__unclassi	OTU2209
d__Bacteri	k__norank_	p__Proteob	c__Gamma	o__Diploric	f__Diploric	g__Aquicel	s__unclassi	OTU2549
d__Bacteri	k__norank_	p__Firmicu	c__Negativ	o__Selenor	f__Acidami	g__Acidam	s__uncultu	OTU2372
d__Bacteri	k__norank_	p__Proteob	c__Gamma	o__KI89A_	f__norank_	g__norank_	s__unclassi	OTU2512
d__Bacteri	k__norank_	p__Firmicu	c__Bacilli	o__Bacillal	f__Planoco	g__Chrysec	s__uncultu	OTU2183
d__Bacteri	k__norank_	p__Cyanob	c__Oxypho	o__Synech	f__Cyanobi	g__Cyanob	s__unident	OTU1613
d__Bacteri	k__norank_	p__Cyanob	c__Oxypho	o__Chlorop	f__norank_	g__norank_	s__unclassi	OTU956
d__Bacteri	k__norank_	p__Proteob	c__Alphapr	o__SAR11_	f__Clade_II	g__norank_	s__uncultu	OTU870
d__Bacteri	k__norank_	p__Gemma	c__Gemma	o__Gemma	f__Gemma	g__Gemma	s__uncultu	OTU1382
d__Bacteri	k__norank_	p__Chlamy	c__Chlamy	o__Chlamy	f__Criblam	g__norank_	s__uncultu	OTU2192
d__Bacteri	k__norank_	p__Cyanob	c__Melaina	o__Gastrar	f__norank_	g__norank_	s__uncultu	OTU1211
d__Bacteri	k__norank_	p__Chlorof	c__Gitt-GS-	o__norank_	f__norank_	g__norank_	s__uncultu	OTU3945
d__Bacteri	k__norank_	p__Bacterc	c__Bactero	o__unclass	f__unclassi	g__unclass	s__unclassi	OTU301
d__Bacteri	k__norank_	p__Acidoba	c__Holopha	o__Holoph	f__Holopha	g__Holoph	s__uncultu	OTU2223
d__Bacteri	k__norank_	p__Actinob	c__Actinob	o__Microc	f__Dermab	g__Brachyb	s__unclassi	OTU1555

d__Bacteri	k__norank_	p__Firmicu	c__Clostrid	o__Clostrid	f__Rumino	g__unclass	s__unclassi	OTU2101
d__Bacteri	k__norank_	p__Chlamy	c__Chlamy	o__Chlamy	f__unclassi	g__unclass	s__unclassi	OTU2713
d__Bacteri	k__norank_	p__Proteol	c__Gamma	o__Xantho	f__Xanthor	g__Thermo	s__uncultu	OTU1220
d__Bacteri	k__norank_	p__Actinol	c__Actinob	o__Corioba	f__Atopobi	g__Olsenel	s__unclassi	OTU1026
d__Bacteri	k__norank_	p__Proteol	c__Gamma	o__Gamma	f__unclassi	g__Candida	s__uncultu	OTU2478
d__Bacteri	k__norank_	p__Patescil	c__Parcuba	o__Candida	f__norank_	g__norank_	s__unclassi	OTU987
d__Bacteri	k__norank_	p__Proteol	c__Alphapr	o__Ricketts	f__Ricketts	g__Ricketts	s__unclassi	OTU2099
d__Bacteri	k__norank_	p__Cyanob	c__Melaina	o__Obscuri	f__norank_	g__norank_	s__uncultu	OTU998
d__Bacteri	k__norank_	p__Proteol	c__Alphapr	o__Rhodol	f__Rhodob	g__unclass	s__unclassi	OTU341
d__Bacteri	k__norank_	p__Firmicu	c__Clostrid	o__Clostrid	f__Lachnos	g__norank_	s__uncultu	OTU2817
d__Bacteri	k__norank_	p__Proteol	c__Gamma	o__Gamma	f__unclassi	g__Candida	s__uncultu	OTU1970
d__Bacteri	k__norank_	p__Verruco	c__Verruco	o__Chthon	f__Chthoni	g__Chthon	s__unclassi	OTU3263
d__Bacteri	k__norank_	p__Proteol	c__Gamma	o__Legione	f__Legione	g__Legione	s__uncultu	OTU1137
d__Bacteri	k__norank_	p__Bactero	c__Bactero	o__Bactero	f__Prevote	g__unclass	s__unclassi	OTU2300
d__Bacteri	k__norank_	p__Depenc	c__Babelia	o__Babelia	f__unclassi	g__unclass	s__unclassi	OTU2785
d__Bacteri	k__norank_	p__Proteol	c__Alphapr	o__Rhizobi	f__Rhizobia	g__unclass	s__unclassi	OTU2002
d__Bacteri	k__norank_	p__Depenc	c__Babelia	o__Babelia	f__unclassi	g__unclass	s__unclassi	OTU2787
d__Bacteri	k__norank_	p__Firmicu	c__Clostrid	o__Clostrid	f__Lachnos	g__Lachnos	s__uncultu	OTU883
d__Bacteri	k__norank_	p__Bactero	c__Bactero	o__Flavoba	f__Flavoba	g__Flavoba	s__unclassi	OTU2585
d__Bacteri	k__norank_	p__Proteol	c__Gamma	o__Betapro	f__TRA3-20	g__norank_	s__unclassi	OTU3788
d__Bacteri	k__norank_	p__Proteol	c__Gamma	o__Legione	f__Legione	g__Legione	s__uncultu	OTU2474
d__Bacteri	k__norank_	p__Cyanob	c__Oxypho	o__Chlorop	f__norank_	g__norank_	s__Epipyxis	OTU1868
d__Bacteri	k__norank_	p__Proteol	c__Alphapr	o__Rhizobi	f__Hyphom	g__Hyphon	s__unclassi	OTU3094
d__Bacteri	k__norank_	p__Proteol	c__Alphapr	o__Caulob	f__Cauloba	g__Caulob	s__unclassi	OTU501
d__Bacteri	k__norank_	p__Bactero	c__Bactero	o__Flavoba	f__Weekse	g__Chrysec	s__Chrysec	OTU1867
d__Bacteri	k__norank_	p__Proteol	c__Gamma	o__Xantho	f__Xanthor	g__Thermo	s__uncultu	OTU3681
d__Bacteri	k__norank_	p__Proteol	c__Alphapr	o__Elsteral	f__norank_	g__norank_	s__metage	OTU3971
d__Bacteri	k__norank_	p__Proteol	c__Gamma	o__Xantho	f__Xanthor	g__Stenotr	s__unclassi	OTU147
d__Bacteri	k__norank_	p__Chlamy	c__Chlamy	o__Chlamy	f__Parachla	g__Neochla	s__metage	OTU2277
d__Bacteri	k__norank_	p__Proteol	c__Gamma	o__Betapro	f__Burkhol	g__unclass	s__unclassi	OTU2735
d__Bacteri	k__norank_	p__Proteol	c__Gamma	o__Betapro	f__Rhodoc	g__Sterolib	s__mine_d	OTU1239
d__Bacteri	k__norank_	p__Bactero	c__Bactero	o__Bactero	f__Tannere	g__Paraba	s__uncultu	OTU1629
d__Bacteri	k__norank_	p__Deinoc	c__Deinoc	o__Deinoc	f__Deinocc	g__Deinoc	s__Deinocc	OTU2144
d__Bacteri	k__norank_	p__Proteol	c__Alphapr	o__Acetob	f__Acetoba	g__norank_	s__unclassi	OTU3244
d__Bacteri	k__norank_	p__Proteol	c__Alphapr	o__Rhizobi	f__Devosia	g__Devosia	s__unclassi	OTU22
d__Bacteri	k__norank_	p__Depenc	c__Babelia	o__Babelia	f__unclassi	g__unclass	s__unclassi	OTU1952
d__Bacteri	k__norank_	p__Spiroch	c__Leptosp	o__Leptosp	f__Leptosp	g__RBG-16	s__unclassi	OTU1161
d__Bacteri	k__norank_	p__Proteol	c__Gamma	o__Diploric	f__Diploric	g__Aquicel	s__unclassi	OTU761
d__Bacteri	k__norank_	p__Bactero	c__Bactero	o__Sphingo	f__Sphingo	g__Pedoba	s__unclassi	OTU1341
d__Bacteri	k__norank_	p__Firmicu	c__Clostrid	o__Clostrid	f__Family_	g__Gallicol	s__Peptost	OTU1606
d__Bacteri	k__norank_	p__Patescil	c__Sacchar	o__Sacchar	f__norank_	g__norank_	s__unclassi	OTU1860
d__Bacteri	k__norank_	p__Proteol	c__Gamma	o__Betapro	f__Burkhol	g__Massilia	s__unclassi	OTU927
d__Bacteri	k__norank_	p__Depenc	c__Babelia	o__Babelia	f__unclassi	g__unclass	s__unclassi	OTU1795
d__Bacteri	k__norank_	p__Depenc	c__Babelia	o__Babelia	f__unclassi	g__unclass	s__unclassi	OTU1790
d__Bacteri	k__norank_	p__Depenc	c__Babelia	o__Babelia	f__Vermipl	g__norank_	s__unclassi	OTU8
d__Bacteri	k__norank_	p__Depenc	c__Babelia	o__Babelia	f__Vermipl	g__norank_	s__uncultu	OTU2056
d__Bacteri	k__norank_	p__Chlorof	c__AD3	o__norank_	f__norank_	g__norank_	s__uncultu	OTU2855

d__Bacteri	k__norank_	p__Rokuba	c__NC10	o__Methyl	f__Methylo	g__Sh765B	s__ uncultu	OTU2968
d__Bacteri	k__norank_	p__Firmicu	c__Bacilli	o__Bacillal	f__Paeniba	g__Breviba	s__unclassi	OTU832
d__Bacteri	k__norank_	p__Cyanob	c__Oxypho	o__Chloro	f__norank_	g__norank_	s__Picochl	OTU1825
d__Bacteri	k__norank_	p__Synergi	c__Synergi	o__Synergi	f__Synergi	g__Fretiba	s__uncultu	OTU1558
d__Bacteri	k__norank_	p__Proteol	c__Gamma	o__Legione	f__Legione	g__Legione	s__uncultu	OTU114
d__Bacteri	k__norank_	p__Firmicu	c__Clostrid	o__Clostrid	f__Lachnos	g__Lachno	s__uncultu	OTU1144
d__Bacteri	k__norank_	p__Proteol	c__Gamma	o__Cellvibr	f__Cellvibri	g__Cellvibr	s__unclassi	OTU3616
d__Bacteri	k__norank_	p__Chlorof	c__Anaerol	o__norank_	f__norank_	g__norank_	s__uncultu	OTU2510
d__Bacteri	k__norank_	p__Proteol	c__Deltapr	o__PB19	f__norank_	g__norank_	s__metage	OTU4094
d__Bacteri	k__norank_	p__Firmicu	c__Clostrid	o__Clostrid	f__Rumino	g__unclass	s__unclassi	OTU1122
d__Bacteri	k__norank_	p__Depenc	c__Babelia	o__Babelia	f__unclassi	g__unclass	s__unclassi	OTU2577
d__Bacteri	k__norank_	p__Rokuba	c__NC10	o__Rokuba	f__norank_	g__norank_	s__uncultu	OTU1423
d__Bacteri	k__norank_	p__Depenc	c__Babelia	o__Babelia	f__unclassi	g__unclass	s__unclassi	OTU2574
d__Bacteri	k__norank_	p__Bacterc	c__Bactero	o__Bacterc	f__Prevote	g__Prevote	s__unclassi	OTU1677
d__Bacteri	k__norank_	p__Depenc	c__Babelia	o__Babelia	f__Vermipl	g__norank_	s__uncultu	OTU2125
d__Bacteri	k__norank_	p__Depenc	c__Babelia	o__Babelia	f__Vermipl	g__norank_	s__uncultu	OTU2124
d__Bacteri	k__norank_	p__Firmicu	c__Bacilli	o__Bacillal	f__Planoco	g__unclass	s__unclassi	OTU1919
d__Bacteri	k__norank_	p__Firmicu	c__Clostrid	o__Clostrid	f__Helioba	g__Hydrog	s__uncultu	OTU599
d__Bacteri	k__norank_	p__Acidob	c__Blastoc	o__Blastoc	f__Blastoc	g__Blastoc	s__uncultu	OTU34
d__Bacteri	k__norank_	p__Bacterc	c__Rhodot	o__Rhodot	f__Rhodotl	g__norank_	s__unclassi	OTU1725
d__Bacteri	k__norank_	p__Acidob	c__Blastoc	o__Blastoc	f__Blastoc	g__Blastoc	s__unclassi	OTU277
d__Bacteri	k__norank_	p__Proteol	c__Gamma	o__Cellvibr	f__Porticoc	g__C1-B04	s__uncultu	OTU2311
d__Bacteri	k__norank_	p__Bacterc	c__Bactero	o__Chitino	f__Chitino	g__norank_	s__uncultu	OTU1345
d__Bacteri	k__norank_	p__Proteol	c__Alphapr	o__Rhizobi	f__Rhizobi	g__Phreatc	s__uncultu	OTU1301
d__Bacteri	k__norank_	p__Firmicu	c__Bacilli	o__Lactoba	f__Lactoba	g__Lactoba	s__unclassi	OTU1620
d__Bacteri	k__norank_	p__Depenc	c__Babelia	o__Babelia	f__norank_	g__norank_	s__uncultu	OTU2483
d__Bacteri	k__norank_	p__Proteol	c__Gamma	o__Xantho	f__Rhodan	g__Tahibac	s__unclassi	OTU1217
d__Bacteri	k__norank_	p__Chlorof	c__Dehalo	o__S085	f__norank_	g__norank_	s__uncultu	OTU2108
d__Bacteri	k__norank_	p__Firmicu	c__Clostrid	o__Clostrid	f__Lachnos	g__Eubacte	s__uncultu	OTU2042
d__Bacteri	k__norank_	p__Firmicu	c__Clostrid	o__Clostrid	f__Lachnos	g__Lachno	s__unclassi	OTU45
d__Bacteri	k__norank_	p__Firmicu	c__Clostrid	o__Clostrid	f__Peptost	g__unclass	s__unclassi	OTU1343
d__Bacteri	k__norank_	p__Proteol	c__Gamma	o__Diploric	f__Diploric	g__norank_	s__uncultu	OTU2230
d__Bacteri	k__norank_	p__Firmicu	c__Bacilli	o__Bacillal	f__Bacillac	g__Fictibac	s__uncultu	OTU97
d__Bacteri	k__norank_	p__Firmicu	c__Clostrid	o__Clostrid	f__Peptost	g__Sporace	s__uncultu	OTU36
d__Bacteri	k__norank_	p__Nitrosp	c__Nitrosp	o__norank_	f__norank_	g__Nitrosp	s__uncultu	OTU2561
d__Bacteri	k__norank_	p__Proteol	c__Deltapr	o__Oligofle	f__Oligofle	g__Silvanig	s__metage	OTU2136
d__Bacteri	k__norank_	p__Verrucc	c__Verrucc	o__Pedosp	f__Pedospl	g__unclass	s__unclassi	OTU1929
d__Bacteri	k__norank_	p__Depenc	c__Babelia	o__Babelia	f__unclassi	g__unclass	s__unclassi	OTU2214
d__Bacteri	k__norank_	p__Actinol	c__Actinob	o__Gaiellal	f__norank_	g__norank_	s__uncultu	OTU3266
d__Bacteri	k__norank_	p__Proteol	c__Gamma	o__Betapr	f__Burkhol	g__Hydrog	s__unclassi	OTU3807
d__Bacteri	k__norank_	p__Firmicu	c__Clostrid	o__Clostrid	f__Family_	g__Symbio	s__uncultu	OTU1531
d__Bacteri	k__norank_	p__Gemma	c__Gemma	o__Gemma	f__Gemma	g__Gemma	s__unclassi	OTU1324
d__Bacteri	k__norank_	p__Firmicu	c__Clostrid	o__Clostrid	f__Family_	g__unclass	s__unclassi	OTU1590
d__Bacteri	k__norank_	p__Proteol	c__Alphapr	o__Paraca	f__Paracae	g__Candid	s__uncultu	OTU1948
d__Bacteri	k__norank_	p__Proteol	c__Gamma	o__Salinisp	f__Solimon	g__Nevskia	s__uncultu	OTU3926
d__Bacteri	k__norank_	p__Bacterc	c__Bactero	o__Bacterc	f__Prevote	g__Allopre	s__Allopre	OTU1116
d__Bacteri	k__norank_	p__Proteol	c__Gamma	o__Diploric	f__Diploric	g__norank_	s__uncultu	OTU1859

d__Bacteri	k__norank_	p__Proteob	c__Deltapr	o__MBNT1	f__norank_	g__norank_	s__unclassi	OTU2477
d__Bacteri	k__norank_	p__Proteob	c__Gamma	o__Diploric	f__Diploric	g__norank_	s__unclassi	OTU2650
d__Bacteri	k__norank_	p__Firmicu	c__Bacilli	o__Lactoba	f__Strepto	g__Strepto	s__unclassi	OTU2355
d__Bacteri	k__norank_	p__Proteob	c__Gamma	o__Legione	f__Legione	g__Legione	s__unclassi	OTU1839
d__Bacteri	k__norank_	p__Proteob	c__Gamma	o__Betapro	f__SC-I-84	g__norank_	s__unclassi	OTU2601
d__Bacteri	k__norank_	p__Actinob	c__Actinob	o__Corynel	f__Coryneb	g__Corynel	s__Coryneb	OTU5
d__Bacteri	k__norank_	p__Proteob	c__Gamma	o__Diploric	f__Diploric	g__Aquicel	s__unclassi	OTU2685
d__Bacteri	k__norank_	p__Depenc	c__Babelia	o__Babelia	f__Vermiph	g__norank_	s__unclassi	OTU2538
d__Bacteri	k__norank_	p__Actinob	c__Actinob	o__Microc	f__Microba	g__MWH-T	s__uncultu	OTU4041
d__Bacteri	k__norank_	p__Firmicu	c__Clostrid	o__Clostrid	f__Rumino	g__Rumino	s__unclassi	OTU29
d__Bacteri	k__norank_	p__Proteob	c__Gamma	o__Diploric	f__Diploric	g__Aquicel	s__unclassi	OTU2371
d__Bacteri	k__norank_	p__Chlamy	c__Chlamy	o__Chlamy	f__Parachla	g__unclass	s__unclassi	OTU1931
d__Bacteri	k__norank_	p__Proteob	c__Gamma	o__Betapro	f__Nitrosor	g__mle1-7	s__uncultu	OTU1685
d__Bacteri	k__norank_	p__Proteob	c__Gamma	o__Pseudo	f__Pseudor	g__Pseudo	s__Pseudor	OTU711
d__Bacteri	k__norank_	p__Proteob	c__Gamma	o__Betapro	f__Neisseri	g__Neisser	s__Neisseri	OTU712
d__Bacteri	k__norank_	p__Chlamy	c__Chlamy	o__Chlamy	f__unclassi	g__unclass	s__unclassi	OTU2392
d__Bacteri	k__norank_	p__Bacterc	c__Bactero	o__Bacterc	f__Prevote	g__Prevote	s__unclassi	OTU1085
d__Bacteri	k__norank_	p__Proteob	c__Alphapr	o__Rhodob	f__Rhodob	g__unclass	s__unclassi	OTU2856
d__Bacteri	k__norank_	p__Proteob	c__Gamma	o__Betapro	f__Nitrosor	g__norank_	s__metage	OTU3683
d__Bacteri	k__norank_	p__Depenc	c__Babelia	o__Babelia	f__norank_	g__norank_	s__metage	OTU2603
d__Bacteri	k__norank_	p__Proteob	c__Gamma	o__Legione	f__Legione	g__Legione	s__uncultu	OTU1529
d__Bacteri	k__norank_	p__Depenc	c__Babelia	o__Babelia	f__Babelia	g__norank_	s__uncultu	OTU2813
d__Bacteri	k__norank_	p__Proteob	c__Gamma	o__Betapro	f__Burkhol	g__Caenim	s__unclassi	OTU3628
d__Bacteri	k__norank_	p__Firmicu	c__Bacilli	o__Bacillal	f__Thermo	g__Planifilu	s__uncultu	OTU1407
d__Bacteri	k__norank_	p__Rokuba	c__NC10	o__Rokuba	f__norank_	g__norank_	s__uncultu	OTU2893
d__Bacteri	k__norank_	p__Bacterc	c__Bactero	o__Flavoba	f__Flavoba	g__Flavoba	s__Flavoba	OTU126
d__Bacteri	k__norank_	p__Proteob	c__Gamma	o__Betapro	f__Burkhol	g__Cupriav	s__Cupriav	OTU921
d__Bacteri	k__norank_	p__Epsilon	c__Campyl	o__Campyl	f__Sulfuros	g__Sulfuros	s__uncultu	OTU723
d__Bacteri	k__norank_	p__Bacterc	c__Ignavib	o__OPB56	f__norank_	g__norank_	s__metage	OTU136
d__Bacteri	k__norank_	p__Cyanob	c__Melaina	o__Gastrar	f__norank_	g__norank_	s__unclassi	OTU1395
d__Bacteri	k__norank_	p__Firmicu	c__Clostrid	o__Clostrid	f__Lachnos	g__Lachnos	s__unclassi	OTU89
d__Bacteri	k__norank_	p__Depenc	c__Babelia	o__Babelia	f__Vermiph	g__norank_	s__unclassi	OTU2668
d__Bacteri	k__norank_	p__Cyanob	c__Oxypho	o__Chlorop	f__norank_	g__norank_	s__unclassi	OTU1582
d__Bacteri	k__norank_	p__Firmicu	c__Clostrid	o__Clostrid	f__Rumino	g__Rumino	s__uncultu	OTU2851
d__Bacteri	k__norank_	p__Depenc	c__Babelia	o__Babelia	f__Vermiph	g__norank_	s__uncultu	OTU1749
d__Bacteri	k__norank_	p__Latescib	c__Latescib	o__norank_	f__norank_	g__norank_	s__unclassi	OTU2727
d__Bacteri	k__norank_	p__Proteob	c__Deltapr	o__Myxocc	f__Polyang	g__Pajaroe	s__unclassi	OTU1938
d__Bacteri	k__norank_	p__Proteob	c__Gamma	o__Betapro	f__Neisseri	g__norank_	s__uncultu	OTU2417
d__Bacteri	k__norank_	p__Acidoba	c__Subgrou	o__norank_	f__norank_	g__norank_	s__metage	OTU3317
d__Bacteri	k__norank_	p__Plancto	c__Plancto	o__Gemma	f__Gemma	g__norank_	s__bacteriu	OTU966
d__Bacteri	k__norank_	p__Firmicu	c__Clostrid	o__Clostrid	f__Lachnos	g__Tyzzere	s__unclassi	OTU1761
d__Bacteri	k__norank_	p__Proteob	c__Gamma	o__Diploric	f__Diploric	g__norank_	s__unclassi	OTU2507
d__Bacteri	k__norank_	p__Proteob	c__Gamma	o__Diploric	f__Diploric	g__norank_	s__unclassi	OTU1705
d__Bacteri	k__norank_	p__Depenc	c__Babelia	o__Babelia	f__unclassi	g__unclass	s__unclassi	OTU2368
d__Bacteri	k__norank_	p__Proteob	c__Alphapr	o__Rhodos	f__Rhodop	g__Defluvii	s__uncultu	OTU3135
d__Bacteri	k__norank_	p__Actinob	c__Actinob	o__Microtr	f__Microtri	g__Sva099	s__uncultu	OTU1691
d__Bacteri	k__norank_	p__Patescil	c__Microge	o__norank_	f__norank_	g__norank_	s__uncultu	OTU1880

d__Bacteri	k__norank_	p__Proteob	c__Gamma	o__Diploric	f__Diploric	g__norank_	s__unclassi	OTU2113
d__Bacteri	k__norank_	p__Depend	c__Babelia	o__Babelia	f__Vermipl	g__norank_	s__unclassi	OTU2207
d__Bacteri	k__norank_	p__Proteob	c__Deltapr	o__SAR324	f__norank_	g__norank_	s__unclassi	OTU1757
d__Bacteri	k__norank_	p__Bacterc	c__Bactero	o__Chitino	f__Chitinop	g__Dinghui	s__metage	OTU1865
d__Bacteri	k__norank_	p__Proteob	c__Gamma	o__Oceanc	f__Sacchar	g__Oleibac	s__uncultu	OTU2232
d__Bacteri	k__norank_	p__Actinob	c__Actinob	o__Microtr	f__Ilumato	g__CL500-2	s__unclassi	OTU1016
d__Bacteri	k__norank_	p__Depend	c__Babelia	o__Babelia	f__unclassi	g__unclass	s__unclassi	OTU2567
d__Bacteri	k__norank_	p__Depend	c__Babelia	o__Babelia	f__unclassi	g__unclass	s__unclassi	OTU2568
d__Bacteri	k__norank_	p__Depend	c__Babelia	o__Babelia	f__unclassi	g__unclass	s__unclassi	OTU2569
d__Bacteri	k__norank_	p__Proteob	c__Gamma	o__Legione	f__Legione	g__Legione	s__unclassi	OTU2594
d__Bacteri	k__norank_	p__Actinob	c__Actinob	o__unclass	f__unclassi	g__unclass	s__unclassi	OTU1601
d__Bacteri	k__norank_	p__Acidoba	c__Blastoca	o__Pyrinon	f__Pyrinom	g__RB41	s__unclassi	OTU2238
d__Bacteri	k__norank_	p__Plancto	c__Plancto	o__Gemma	f__Gemma	g__norank_	s__uncultu	OTU3855
d__Bacteri	k__norank_	p__Firmicu	c__Bacilli	o__Bacillal	f__Paeniba	g__Paeniba	s__unclassi	OTU1113
d__Bacteri	k__norank_	p__Proteob	c__Gamma	o__Betapro	f__T34	g__norank_	s__uncultu	OTU2802
d__Bacteri	k__norank_	p__Nitrosp	c__Thermo	o__norank_	f__norank_	g__norank_	s__uncultu	OTU1674
d__Bacteri	k__norank_	p__Omnitr	c__norank_	o__norank_	f__norank_	g__norank_	s__unclassi	OTU1789
d__Bacteri	k__norank_	p__Bacterc	c__Bactero	o__Flavoba	f__Flavoba	g__Flavoba	s__unclassi	OTU390
d__Bacteri	k__norank_	p__Bacterc	c__Bactero	o__Cytopha	f__Hymenc	g__Hymen	s__unclassi	OTU1418
d__Bacteri	k__norank_	p__Chlorof	c__unclassi	o__unclass	f__unclassi	g__unclass	s__unclassi	OTU2030
d__Bacteri	k__norank_	p__Bacterc	c__Bactero	o__Chitino	f__Chitinop	g__Terrimc	s__unclassi	OTU939
d__Bacteri	k__norank_	p__Bacterc	c__Bactero	o__Bacterc	f__Rikenell	g__Rikenel	s__uncultu	OTU1630
d__Bacteri	k__norank_	p__Actinob	c__Actinob	o__Corynel	f__Mycoba	g__Mycoba	s__Mycoba	OTU1209
d__Bacteri	k__norank_	p__Firmicu	c__Bacilli	o__Bacillal	f__Paeniba	g__Aneurir	s__uncultu	OTU1379
d__Bacteri	k__norank_	p__Chlamy	c__Chlamy	o__Chlamy	f__Simkani	g__norank_	s__uncultu	OTU1918
d__Bacteri	k__norank_	p__Chlorof	c__Anaerol	o__Anaero	f__Anaerol	g__norank_	s__uncultu	OTU2518
d__Bacteri	k__norank_	p__Chlorof	c__Anaerol	o__Anaero	f__Anaerol	g__unclass	s__unclassi	OTU2074
d__Bacteri	k__norank_	p__Actinob	c__Actinob	o__Propior	f__Nocardi	g__Aeromi	s__uncultu	OTU15
d__Bacteri	k__norank_	p__Depend	c__Babelia	o__Babelia	f__Vermipl	g__norank_	s__uncultu	OTU1957
d__Bacteri	k__norank_	p__Firmicu	c__Clostrid	o__Clostrid	f__Rumino	g__Ruminc	s__uncultu	OTU1192
d__Bacteri	k__norank_	p__Proteob	c__Alphapr	o__Acetob	f__Acetoba	g__norank_	s__metage	OTU3022
d__Bacteri	k__norank_	p__Proteob	c__Gamma	o__Gamma	f__unclassi	g__Candida	s__uncultu	OTU1796
d__Bacteri	k__norank_	p__Firmicu	c__Bacilli	o__Bacillal	f__Thermo	g__unclass	s__unclassi	OTU1577
d__Bacteri	k__norank_	p__Nitrosp	c__Nitrosp	o__norank_	f__norank_	g__Nitrosp	s__uncultu	OTU3672
d__Bacteri	k__norank_	p__Firmicu	c__Bacilli	o__Bacillal	f__Paeniba	g__Paeniba	s__unclassi	OTU1939
d__Bacteri	k__norank_	p__Proteob	c__Gamma	o__Betapro	f__TRA3-20	g__norank_	s__metage	OTU3781
d__Bacteri	k__norank_	p__Cyanob	c__Melaina	o__Gastran	f__norank_	g__norank_	s__uncultu	OTU1456
d__Bacteri	k__norank_	p__Cyanob	c__Oxypho	o__Chlorop	f__norank_	g__norank_	s__unclassi	OTU1926
d__Bacteri	k__norank_	p__Proteob	c__Alphapr	o__Rhizobi	f__Xanthob	g__Rhodop	s__unclassi	OTU240
d__Bacteri	k__norank_	p__Firmicu	c__Clostrid	o__Clostrid	f__Rumino	g__unclass	s__unclassi	OTU1037
d__Bacteri	k__norank_	p__Chlorof	c__Dehaloc	o__S085	f__norank_	g__norank_	s__metage	OTU3905
d__Bacteri	k__norank_	p__Chlamy	c__Chlamy	o__Chlamy	f__cvE6	g__norank_	s__uncultu	OTU2271
d__Bacteri	k__norank_	p__Acidoba	c__Subgrou	o__norank_	f__norank_	g__norank_	s__unclassi	OTU496
d__Bacteri	k__norank_	p__Chlamy	c__Chlamy	o__Chlamy	f__unclassi	g__unclass	s__unclassi	OTU2245
d__Bacteri	k__norank_	p__Proteob	c__Alphapr	o__Sphingo	f__Sphingo	g__Polymo	s__uncultu	OTU367
d__Bacteri	k__norank_	p__unclass	c__unclassi	o__unclass	f__unclassi	g__unclass	s__unclassi	OTU1454
d__Bacteri	k__norank_	p__Proteob	c__Gamma	o__Pseudo	f__Moraxe	g__Acineto	s__unclassi	OTU2675

d__Bacteri	k__norank_	p__Acidoba	c__Subgrou	o__norank_	f__norank_	g__norank_	s__unclassi	OTU2509
d__Bacteri	k__norank_	p__Firmicu	c__Bacilli	o__Lactoba	f__Streptoc	g__Lactoco	s__unclassi	OTU2208
d__Bacteri	k__norank_	p__Proteob	c__Gamma	o__Betapro	f__Burkhol	g__GKS98_	s__uncultu	OTU199
d__Bacteri	k__norank_	p__Firmicu	c__Clostrid	o__Clostrid	f__Rumino	g__unclass	s__unclassi	OTU2828
d__Bacteri	k__norank_	p__Acidoba	c__Subgrou	o__norank_	f__norank_	g__norank_	s__unclassi	OTU2502
d__Bacteri	k__norank_	p__Depend	c__Babelia	o__Babelia	f__Vermiph	g__norank_	s__uncultu	OTU2607
d__Bacteri	k__norank_	p__Actinob	c__Actinob	o__Bifidob	f__Bifidoba	g__Bifidob	s__Bifidoba	OTU2413
d__Bacteri	k__norank_	p__Proteob	c__Gamma	o__Betapro	f__Nitrosor	g__966-1	s__unclassi	OTU3280
d__Bacteri	k__norank_	p__Firmicu	c__Clostrid	o__Clostrid	f__Family_	g__norank_	s__uncultu	OTU1585
d__Bacteri	k__norank_	p__Acidoba	c__Blastoca	o__DS-100	f__norank_	g__norank_	s__uncultu	OTU1896
d__Bacteri	k__norank_	p__Proteob	c__Gamma	o__Betapro	f__Burkhol	g__Noviher	s__unclassi	OTU481
d__Bacteri	k__norank_	p__Plancto	c__unclassi	o__unclass	f__unclassi	g__unclass	s__unclassi	OTU2155
d__Bacteri	k__norank_	p__Depend	c__Babelia	o__Babelia	f__unclassi	g__unclass	s__unclassi	OTU1892
d__Bacteri	k__norank_	p__Chlamy	c__Chlamy	o__Chlamy	f__cvE6	g__norank_	s__uncultu	OTU1690
d__Bacteri	k__norank_	p__Plancto	c__Phycisp	o__Phycisp	f__Phycispl	g__SM1A0	s__metage	OTU523
d__Bacteri	k__norank_	p__Chlamy	c__Chlamy	o__Chlamy	f__cvE6	g__norank_	s__metage	OTU1844
d__Bacteri	k__norank_	p__Proteob	c__Gamma	o__Acidifer	f__Acidifer	g__Sulfurifi	s__unclassi	OTU1976
d__Bacteri	k__norank_	p__Bacterc	c__Bactero	o__Chitino	f__Chitinop	g__unclass	s__unclassi	OTU1062
d__Bacteri	k__norank_	p__Bacterc	c__Bactero	o__Cytopha	f__unclassi	g__unclass	s__unclassi	OTU1760
d__Bacteri	k__norank_	p__Proteob	c__Gamma	o__Diploric	f__Diploric	g__Aquicel	s__uncultu	OTU1772
d__Bacteri	k__norank_	p__Actinob	c__Actinob	o__Microco	f__Micrococ	g__Arthrob	s__Arthrob	OTU1427
d__Bacteri	k__norank_	p__Proteob	c__Alphapr	o__Acetoba	f__Acetoba	g__norank_	s__metage	OTU3728
d__Bacteri	k__norank_	p__Proteob	c__Gamma	o__Gamma	f__unclassi	g__Candida	s__uncultu	OTU1213
d__Bacteri	k__norank_	p__Bacterc	c__Bactero	o__Chitino	f__Chitinop	g__Paraseg	s__Paraseg	OTU1352
d__Bacteri	k__norank_	p__Bacterc	c__Bactero	o__Cytopha	f__Hymenc	g__Hymeno	s__Hymenc	OTU1446
d__Bacteri	k__norank_	p__Bacterc	c__Bactero	o__Sphingo	f__NS11-12	g__norank_	s__uncultu	OTU307
d__Bacteri	k__norank_	p__Proteob	c__Gamma	o__Enterob	f__Enterob	g__Escheric	s__unclassi	OTU1633
d__Bacteri	k__norank_	p__Bacterc	c__Bactero	o__Flavoba	f__Flavoba	g__Flavoba	s__unclassi	OTU78
d__Bacteri	k__norank_	p__Firmicu	c__Bacilli	o__Bacillale	f__Thermo	g__Kropper	s__unclassi	OTU1326
d__Bacteri	k__norank_	p__Proteob	c__Alphapr	o__Acetoba	f__Acetoba	g__Rubrite	s__uncultu	OTU247
d__Bacteri	k__norank_	p__Proteob	c__Gamma	o__Gamma	f__unclassi	g__Candida	s__uncultu	OTU2326
d__Bacteri	k__norank_	p__Proteob	c__Gamma	o__unclass	f__unclassi	g__unclass	s__unclassi	OTU2460
d__Bacteri	k__norank_	p__Bacterc	c__Bactero	o__Chitino	f__Saprosp	g__norank_	s__uncultu	OTU1072
d__Bacteri	k__norank_	p__Cyanob	c__Oxypho	o__Chlorop	f__norank_	g__norank_	s__Auxeno	OTU1472
d__Bacteri	k__norank_	p__Proteob	c__Gamma	o__Diploric	f__Diploric	g__norank_	s__unclassi	OTU2712
d__Bacteri	k__norank_	p__Chlamy	c__Chlamy	o__Chlamy	f__Simkani	g__norank_	s__unclassi	OTU1956
d__Bacteri	k__norank_	p__Proteob	c__Gamma	o__Diploric	f__Diploric	g__norank_	s__unclassi	OTU1843
d__Bacteri	k__norank_	p__Bacterc	c__Bactero	o__Bacterc	f__Prevote	g__Prevote	s__uncultu	OTU2543
d__Bacteri	k__norank_	p__Proteob	c__Deltapr	o__Myxocc	f__Archang	g__Anaero	s__unclassi	OTU2342
d__Bacteri	k__norank_	p__Proteob	c__Deltapr	o__Desulfu	f__norank_	g__norank_	s__uncultu	OTU2239
d__Bacteri	k__norank_	p__Depend	c__Babelia	o__Babelia	f__Vermiph	g__norank_	s__metage	OTU2579
d__Bacteri	k__norank_	p__Proteob	c__Deltapr	o__Desulfo	f__Desulfol	g__Desulfa	s__uncultu	OTU2676
d__Bacteri	k__norank_	p__Proteob	c__Gamma	o__Legione	f__Legione	g__Legione	s__unclassi	OTU1732
d__Bacteri	k__norank_	p__Chlorof	c__OLB14	o__norank_	f__norank_	g__norank_	s__uncultu	OTU1268
d__Bacteri	k__norank_	p__Proteob	c__Gamma	o__Vibrion	f__Vibriona	g__Photob	s__unclassi	OTU2748
d__Bacteri	k__norank_	p__Proteob	c__Gamma	o__Legione	f__Legione	g__Legione	s__unclassi	OTU2423
d__Bacteri	k__norank_	p__Bacterc	c__Bactero	o__Sphingo	f__env.OPS	g__norank_	s__unclassi	OTU2657

d__Bacteri	k__norank_	p__Firmicu	c__Bacilli	o__Bacillal	f__Alicyclol	g__Acidiba	s__unclassi	OTU889
d__Bacteri	k__norank_	p__Firmicu	c__BRH-c2(	o__norank_	f__norank_	g__norank_	s__uncultu	OTU1071
d__Bacteri	k__norank_	p__Bacterc	c__Bactero	o__Bacterc	f__unclassi	g__unclass	s__unclassi	OTU1619
d__Bacteri	k__norank_	p__Chlamy	c__Chlamy	o__Chlamy	f__Simkani	g__norank_	s__unclassi	OTU1656
d__Bacteri	k__norank_	p__Firmicu	c__Clostrid	o__Clostrid	f__Family_	g__norank_	s__uncultu	OTU2705
d__Bacteri	k__norank_	p__Proteol	c__Alphapr	o__Sphingc	f__Sphingo	g__Sphingc	s__unclassi	OTU1417
d__Bacteri	k__norank_	p__Bacterc	c__Bactero	o__Cytoph	f__Hymenc	g__Rufibac	s__uncultu	OTU1288
d__Bacteri	k__norank_	p__Verrucc	c__Verrucc	o__Verrucc	f__Akkerm	g__Akkerm	s__unclassi	OTU1663
d__Bacteri	k__norank_	p__Proteol	c__Gamma	o__Betaprc	f__unclassi	g__unclass	s__unclassi	OTU989
d__Bacteri	k__norank_	p__Proteol	c__Alphapr	o__unclass	f__unclassi	g__unclass	s__unclassi	OTU330
d__Bacteri	k__norank_	p__Chlamy	c__Chlamy	o__Chlamy	f__Parachl	g__Neochl	s__unclassi	OTU2596
d__Bacteri	k__norank_	p__Proteol	c__Deltapr	o__Bdellov	f__Bdellovi	g__Bdellov	s__unclassi	OTU506
d__Bacteri	k__norank_	p__Firmicu	c__Clostrid	o__Clostrid	f__Rumino	g__Ruminic	s__uncultu	OTU1049
d__Bacteri	k__norank_	p__Chlamy	c__Chlamy	o__Chlamy	f__unclassi	g__unclass	s__unclassi	OTU1759
d__Bacteri	k__norank_	p__Depenc	c__Babelia	o__Babelia	f__unclassi	g__unclass	s__unclassi	OTU2229
d__Bacteri	k__norank_	p__Proteol	c__Alphapr	o__Punicei	f__SAR116_	g__norank_	s__unclassi	OTU2379
d__Bacteri	k__norank_	p__Actinol	c__Actinob	o__Microc	f__Microcc	g__Paenart	s__unclassi	OTU1032
d__Bacteri	k__norank_	p__Proteol	c__Alphapr	o__Paracae	f__Paracae	g__Candid	s__metage	OTU238
d__Bacteri	k__norank_	p__Cyanob	c__Oxypho	o__Chlorop	f__norank_	g__norank_	s__unclassi	OTU1806
d__Bacteri	k__norank_	p__Proteol	c__Gamma	o__Betaprc	f__Hydroge	g__Thiobac	s__unclassi	OTU1511
d__Bacteri	k__norank_	p__Proteol	c__Alphapr	o__Rickett	f__SM2D12	g__norank_	s__uncultu	OTU1096
d__Bacteri	k__norank_	p__Proteol	c__Alphapr	o__Sphingc	f__Sphingo	g__Novosp	s__Novosp	OTU728
d__Bacteri	k__norank_	p__Actinol	c__Actinob	o__Nitrilirù	f__Nitrilirù	g__unclass	s__unclassi	OTU1562
d__Bacteri	k__norank_	p__Chlamy	c__Chlamy	o__Chlamy	f__cvE6	g__norank_	s__unclassi	OTU2758
d__Bacteri	k__norank_	p__Proteol	c__Deltapr	o__Myxocc	f__Nannoc	g__unclass	s__unclassi	OTU2114
d__Bacteri	k__norank_	p__Verrucc	c__Verrucc	o__Chthon	f__Chthoni	g__Chthon	s__unclassi	OTU3346
d__Bacteri	k__norank_	p__Proteol	c__Gamma	o__Betaprc	f__Burkhol	g__Massili	s__unclassi	OTU129
d__Bacteri	k__norank_	p__Depenc	c__Babelia	o__Babelia	f__Vermipl	g__norank_	s__unclassi	OTU2303
d__Bacteri	k__norank_	p__Proteol	c__Gamma	o__Salinisp	f__Solimon	g__Hydroc	s__Hydroc	OTU2117
d__Bacteri	k__norank_	p__Firmicu	c__Clostrid	o__Clostrid	f__Rumino	g__Rumino	s__unclassi	OTU1371
d__Bacteri	k__norank_	p__Proteol	c__Alphapr	o__Rickett	f__S25-593	g__norank_	s__unclassi	OTU2137
d__Bacteri	k__norank_	p__Proteol	c__Gamma	o__Betaprc	f__Burkhol	g__Caenim	s__unclassi	OTU2360
d__Bacteri	k__norank_	p__Depenc	c__Babelia	o__Babelia	f__Vermipl	g__norank_	s__uncultu	OTU1827
d__Bacteri	k__norank_	p__Depenc	c__Babelia	o__Babelia	f__unclassi	g__unclass	s__unclassi	OTU2770
d__Bacteri	k__norank_	p__Depenc	c__Babelia	o__Babelia	f__Vermipl	g__norank_	s__uncultu	OTU1824
d__Bacteri	k__norank_	p__Bacterc	c__Bactero	o__Sphingc	f__AKYH76	g__norank_	s__uncultu	OTU2656
d__Bacteri	k__norank_	p__Chlamy	c__Chlamy	o__Chlamy	f__Simkani	g__norank_	s__metage	OTU2464
d__Bacteri	k__norank_	p__Proteol	c__Gamma	o__Enterol	f__Enterob	g__Proteus	s__unclassi	OTU2804
d__Bacteri	k__norank_	p__Proteol	c__Gamma	o__Ectothi	f__Ectothic	g__norank_	s__uncultu	OTU2621
d__Bacteri	k__norank_	p__Bacterc	c__Bactero	o__Cytoph	f__Hymenc	g__Adhaeri	s__uncultu	OTU1439
d__Bacteri	k__norank_	p__Actinol	c__Actinob	o__Actinor	f__norank_	g__norank_	s__uncultu	OTU1912
d__Bacteri	k__norank_	p__Firmicu	c__Clostrid	o__Clostrid	f__Lachnos	g__unclass	s__unclassi	OTU1463
d__Bacteri	k__norank_	p__Proteol	c__Alphapr	o__Rhodol	f__Rhodob	g__Pseudo	s__uncultu	OTU3701
d__Bacteri	k__norank_	p__Bacterc	c__Bactero	o__Bacterc	f__Marinifi	g__Marinif	s__unclassi	OTU2644
d__Bacteri	k__norank_	p__Depenc	c__Babelia	o__Babelia	f__Vermipl	g__norank_	s__unclassi	OTU2157
d__Bacteri	k__norank_	p__Bacterc	c__Bactero	o__Cytoph	f__Microsc	g__OLB12	s__uncultu	OTU1035
d__Bacteri	k__norank_	p__Proteol	c__Deltapr	o__Myxocc	f__Vulgatit	g__Vulgatil	s__unclassi	OTU2826

d__Bacteri	k__norank	p__Nitrosp	c__Nitrosp	o__norank	f__norank_	g__Nitrosp	s__metage	OTU146
d__Bacteri	k__norank	p__Fusoba	c__Fusobac	o__Fusoba	f__Fusobac	g__Fusoba	s__unclassi	OTU760
d__Bacteri	k__norank	p__Proteol	c__Alphapr	o__Sphingo	f__Sphingo	g__Sphingo	s__unclassi	OTU39
d__Bacteri	k__norank	p__Cyanob	c__Oxypho	o__Chlorop	f__norank_	g__norank	s__unclassi	OTU1027
d__Bacteri	k__norank	p__Proteol	c__Deltapr	o__Myxocc	f__Polyang	g__Pajaroe	s__metage	OTU2387
d__Bacteri	k__norank	p__Firmicu	c__Bacilli	o__Bacillal	f__Family_	g__Exiguob	s__Exiguob	OTU96
d__Bacteri	k__norank	p__Firmicu	c__Bacilli	o__Bacillal	f__Bacillac	g__unclass	s__unclassi	OTU1571
d__Bacteri	k__norank	p__Proteol	c__Deltapr	o__unclass	f__unclassi	g__unclass	s__unclassi	OTU2829
d__Bacteri	k__norank	p__Proteol	c__Gamma	o__Legione	f__Legione	g__Legione	s__uncultu	OTU2220
d__Bacteri	k__norank	p__Proteol	c__Gamma	o__Betapro	f__Burkhol	g__Schlege	s__uncultu	OTU738
d__Bacteri	k__norank	p__Firmicu	c__Clostrid	o__Clostrid	f__Syntrop	g__Dethiol	s__uncultu	OTU2031
d__Bacteri	k__norank	p__Firmicu	c__Clostrid	o__Clostrid	f__Lachnos	g__Lachnos	s__unclassi	OTU2844
d__Bacteri	k__norank	p__Depenc	c__Babelia	o__Babelia	f__unclassi	g__unclass	s__unclassi	OTU2683
d__Bacteri	k__norank	p__Firmicu	c__Clostrid	o__Clostrid	f__Rumino	g__Faecalib	s__unclassi	OTU30
d__Bacteri	k__norank	p__Proteol	c__Alphapr	o__Punicei	f__SAR116_	g__Candida	s__uncultu	OTU1627
d__Bacteri	k__norank	p__Bacterc	c__Bactero	o__Bacterc	f__Rikenell	g__Rikenel	s__uncultu	OTU2652
d__Bacteri	k__norank	p__Depenc	c__Babelia	o__Babelia	f__UBA124	g__norank	s__uncultu	OTU2241
d__Bacteri	k__norank	p__Cyanob	c__Oxypho	o__Chlorop	f__norank_	g__norank	s__Dinobry	OTU1877
d__Bacteri	k__norank	p__Chlamy	c__Chlamy	o__Chlamy	f__cvE6	g__norank	s__unclassi	OTU1829
d__Bacteri	k__norank	p__Proteol	c__Gamma	o__Betapro	f__Burkhol	g__Acidovo	s__Acidovo	OTU133
d__Bacteri	k__norank	p__Proteol	c__Deltapr	o__Myxocc	f__BIrii41	g__norank	s__uncultu	OTU1910
d__Bacteri	k__norank	p__Proteol	c__Deltapr	o__MBNT1	f__norank_	g__norank	s__uncultu	OTU3657
d__Bacteri	k__norank	p__Depenc	c__Babelia	o__Babelia	f__Vermipl	g__norank	s__unclassi	OTU2092
d__Bacteri	k__norank	p__Proteol	c__Gamma	o__Betapro	f__Burkhol	g__AAP99	s__uncultu	OTU4
d__Bacteri	k__norank	p__Depenc	c__Babelia	o__Babelia	f__Vermipl	g__norank	s__unclassi	OTU2091
d__Bacteri	k__norank	p__Bacterc	c__Bactero	o__Bacterc	f__Dysgonc	g__Protein	s__anaerol	OTU1169
d__Bacteri	k__norank	p__Cyanob	c__Melaina	o__Gastrar	f__norank_	g__norank	s__unclassi	OTU1564
d__Bacteri	k__norank	p__Proteol	c__Gamma	o__Betapro	f__Methylo	g__unclass	s__unclassi	OTU2943
d__Bacteri	k__norank	p__Verruco	c__Verruco	o__Opituta	f__Puniceio	g__MB11C	s__uncultu	OTU2589
d__Bacteri	k__norank	p__Acidoba	c__Subgrou	o__norank	f__norank_	g__norank	s__uncultu	OTU2636
d__Bacteri	k__norank	p__Firmicu	c__Clostrid	o__Clostrid	f__Clostridi	g__Oxobac	s__uncultu	OTU1312
d__Bacteri	k__norank	p__Firmicu	c__Clostrid	o__Clostrid	f__Rumino	g__unclass	s__unclassi	OTU1490
d__Bacteri	k__norank	p__Chlamy	c__Chlamy	o__Chlamy	f__Simkani	g__norank	s__metage	OTU2127
d__Bacteri	k__norank	p__Epsilon	c__Campyl	o__Campyl	f__Campylo	g__Campyl	s__Campylo	OTU52
d__Bacteri	k__norank	p__Proteol	c__Alphapr	o__Rhizobi	f__Beijerin	g__unclass	s__unclassi	OTU1370
d__Bacteri	k__norank	p__Cyanob	c__Sericytc	o__norank	f__norank_	g__norank	s__uncultu	OTU1398
d__Bacteri	k__norank	p__Proteol	c__Gamma	o__Betapro	f__Burkhol	g__Rhodof	s__unclassi	OTU2720
d__Bacteri	k__norank	p__Proteol	c__Alphapr	o__Rhizobi	f__Devosia	g__norank	s__metage	OTU3034
d__Bacteri	k__norank	p__Actinob	c__Actinob	o__Actinor	f__Actinom	g__Actinon	s__unclassi	OTU1125
d__Bacteri	k__norank	p__Proteol	c__Alphapr	o__Paracae	f__Paracae	g__norank	s__uncultu	OTU1984
d__Bacteri	k__norank	p__Firmicu	c__Clostrid	o__Clostrid	f__Peptocc	g__unclass	s__unclassi	OTU2199
d__Bacteri	k__norank	p__Firmicu	c__Bacilli	o__Bacillal	f__Planoco	g__Lysiniba	s__uncultu	OTU676
d__Bacteri	k__norank	p__Depenc	c__Babelia	o__Babelia	f__Vermipl	g__norank	s__uncultu	OTU2700
d__Bacteri	k__norank	p__Bacterc	c__Bactero	o__Chitino	f__Sapros p	g__norank	s__uncultu	OTU321
d__Bacteri	k__norank	p__Proteol	c__Alphapr	o__Sphingo	f__Sphingo	g__Sphingo	s__unclassi	OTU182
d__Bacteri	k__norank	p__Chlamy	c__Chlamy	o__Chlamy	f__Simkani	g__norank	s__unclassi	OTU2687
d__Bacteri	k__norank	p__Proteol	c__Gamma	o__unclass	f__unclassi	g__unclass	s__unclassi	OTU1635

d__Bacteri	k__norank_	p__Firmicu	c__Clostrid	o__Clostrid	f__Family_	g__Parvim	s__unclassi	OTU1888
d__Bacteri	k__norank_	p__Bacter	c__Bactero	o__Bacter	f__Bactero	g__Bacter	s__uncultu	OTU943
d__Bacteri	k__norank_	p__Fusoba	c__Fusoba	o__Fusoba	f__Fusobac	g__Fusoba	s__unclassi	OTU1546
d__Bacteri	k__norank_	p__Firmicu	c__Bacilli	o__Bacillal	f__Paeniba	g__Paeniba	s__Paeniba	OTU1486
d__Bacteri	k__norank_	p__Bacter	c__Bactero	o__Chitino	f__Chitino	g__Ferrugi	s__metage	OTU1079
d__Bacteri	k__norank_	p__Proteol	c__Gamma	o__Betapr	f__Burkhol	g__unclass	s__unclassi	OTU932
d__Bacteri	k__norank_	p__Cyanob	c__Oxypho	o__Chloro	f__norank_	g__norank_	s__Monod	OTU2420
d__Bacteri	k__norank_	p__Depend	c__Babelia	o__Babelia	f__Vermip	g__norank_	s__uncultu	OTU2141
d__Bacteri	k__norank_	p__Firmicu	c__Bacilli	o__Bacillal	f__Paeniba	g__Paeniba	s__unclassi	OTU1101
d__Bacteri	k__norank_	p__Firmicu	c__Bacilli	o__Bacillal	f__Paeniba	g__Paeniba	s__unclassi	OTU1103
d__Bacteri	k__norank_	p__Proteol	c__Gamma	o__Legione	f__Legione	g__Legione	s__uncultu	OTU91
d__Bacteri	k__norank_	p__Chlamy	c__Chlamy	o__Chlamy	f__unclassi	g__unclass	s__unclassi	OTU2640
d__Bacteri	k__norank_	p__Proteol	c__Gamma	o__Entero	f__Enterob	g__Edward	s__Edward	OTU1254
d__Bacteri	k__norank_	p__Acidob	c__Holoph	o__Subgro	f__norank_	g__norank_	s__uncultu	OTU2989
d__Bacteri	k__norank_	p__Proteol	c__Gamma	o__unclass	f__unclassi	g__unclass	s__unclassi	OTU2212
d__Bacteri	k__norank_	p__Proteol	c__Gamma	o__Xantho	f__Xanthor	g__Lysobac	s__unclassi	OTU1431
d__Bacteri	k__norank_	p__Bacter	c__Bactero	o__Flavoba	f__Weekse	g__Chryse	s__uncultu	OTU1316
d__Bacteri	k__norank_	p__Actinol	c__Actinob	o__Coryne	f__Nocardi	g__William	s__unclassi	OTU1055
d__Bacteri	k__norank_	p__Proteol	c__Gamma	o__Diploric	f__Diploric	g__norank_	s__unclassi	OTU2098
d__Bacteri	k__norank_	p__Proteol	c__Gamma	o__Legione	f__Legione	g__Legione	s__unclassi	OTU2105
d__Bacteri	k__norank_	p__Bacter	c__Bactero	o__Flavoba	f__Weekse	g__Cloacib	s__uncultu	OTU148
d__Bacteri	k__norank_	p__Depend	c__Babelia	o__Babelia	f__Vermip	g__norank_	s__unclassi	OTU2542
d__Bacteri	k__norank_	p__Proteol	c__Gamma	o__Compe	f__Compet	g__Candid	s__uncultu	OTU685
d__Bacteri	k__norank_	p__Proteol	c__Gamma	o__CCD24	f__norank_	g__norank_	s__uncultu	OTU2745
d__Bacteri	k__norank_	p__Proteol	c__Gamma	o__Diploric	f__Diploric	g__Aquicel	s__unclassi	OTU2696
d__Bacteri	k__norank_	p__unclass	c__unclassi	o__unclass	f__unclassi	g__unclass	s__unclassi	OTU1514
d__Bacteri	k__norank_	p__Proteol	c__Gamma	o__Betapr	f__Methylo	g__Methyl	s__unclassi	OTU3649
d__Bacteri	k__norank_	p__Bacter	c__Bactero	o__Cytoph	f__Hymeno	g__Pontiba	s__unclassi	OTU1660
d__Bacteri	k__norank_	p__Proteol	c__Alphapr	o__Rickett	f__Mitocho	g__norank_	s__metage	OTU1611
d__Bacteri	k__norank_	p__Firmicu	c__Bacilli	o__Bacillal	f__Bacillac	g__Oceano	s__uncultu	OTU1224
d__Bacteri	k__norank_	p__Proteol	c__Gamma	o__Cellvibr	f__Cellvibri	g__norank_	s__unclassi	OTU2004
d__Bacteri	k__norank_	p__Firmicu	c__Clostrid	o__Clostrid	f__Clostridi	g__Clostrid	s__unclassi	OTU2037
d__Bacteri	k__norank_	p__Proteol	c__Gamma	o__unclass	f__unclassi	g__unclass	s__unclassi	OTU2076
d__Bacteri	k__norank_	p__Proteol	c__Alphapr	o__Acetob	f__Acetoba	g__Acidoce	s__unclassi	OTU994
d__Bacteri	k__norank_	p__Actinol	c__Actinob	o__Bifidob	f__Bifidoba	g__Bifidob	s__unclassi	OTU1834
d__Bacteri	k__norank_	p__Proteol	c__Gamma	o__Diploric	f__Diploric	g__norank_	s__uncultu	OTU2282
d__Bacteri	k__norank_	p__Proteol	c__Gamma	o__Coxiella	f__Coxiella	g__Coxiella	s__uncultu	OTU1923
d__Bacteri	k__norank_	p__Proteol	c__Alphapr	o__Sphingo	f__Sphingo	g__norank_	s__unclassi	OTU1212
d__Bacteri	k__norank_	p__Actinol	c__Actinob	o__Actinor	f__Actinom	g__Actinon	s__unclassi	OTU1193
d__Bacteri	k__norank_	p__Proteol	c__Alphapr	o__Rhizobi	f__Beijerin	g__norank_	s__unclassi	OTU398
d__Bacteri	k__norank_	p__Actinol	c__Actinob	o__Coryne	f__Nocardi	g__Rhodoc	s__Rhodoc	OTU1556
d__Bacteri	k__norank_	p__Proteol	c__Gamma	o__Diploric	f__Diploric	g__Aquicel	s__uncultu	OTU2547
d__Bacteri	k__norank_	p__Depend	c__Babelia	o__Babelia	f__Babelia	g__norank_	s__uncultu	OTU2793
d__Bacteri	k__norank_	p__Proteol	c__Alphapr	o__Rhodos	f__AEGEAN	g__norank_	s__unclassi	OTU1961
d__Bacteri	k__norank_	p__Firmicu	c__Clostrid	o__Clostrid	f__Family_	g__Tissiere	s__uncultu	OTU2083
d__Bacteri	k__norank_	p__Proteol	c__Gamma	o__Betapr	f__unclassi	g__unclass	s__unclassi	OTU1597
d__Bacteri	k__norank_	p__Actinol	c__Actinob	o__Coryne	f__Dietziac	g__Dietzia	s__Dietzia_	OTU1154

d__Bacteri	k__norank_	p__Proteob	c__Alphapr	o__Rhodob	f__Rhodob	g__Rubellir	s__uncultu	OTU25
d__Bacteri	k__norank_	p__Proteob	c__Alphapr	o__norank_	f__norank_	g__norank_	s__uncultu	OTU3898
d__Bacteri	k__norank_	p__Chlorof	c__Chlorof	o__Thermo	f__Thermo	g__unclass	s__unclassi	OTU1586
d__Bacteri	k__norank_	p__Firmicu	c__Bacilli	o__Bacillal	f__Bacillac	g__Gracilib	s__unclassi	OTU1406
d__Bacteri	k__norank_	p__Teneric	c__Mollicu	o__Izimapl	f__norank_	g__norank_	s__unclassi	OTU1515
d__Bacteri	k__norank_	p__Chlorof	c__JG30-KF	o__norank_	f__norank_	g__norank_	s__unclassi	OTU2052
d__Bacteri	k__norank_	p__Firmicu	c__Bacilli	o__Bacillal	f__Bacillac	g__Bacillus	s__Bacillus_	OTU1792
d__Bacteri	k__norank_	p__Firmicu	c__Clostrid	o__Clostrid	f__Lachnos	g__Tyzzere	s__Galloisi	OTU1424
d__Bacteri	k__norank_	p__Firmicu	c__Clostrid	o__Clostrid	f__Family_	g__Tissiere	s__uncultu	OTU1287
d__Bacteri	k__norank_	p__Depend	c__Babelia	o__Babelia	f__unclassi	g__unclass	s__unclassi	OTU1883
d__Bacteri	k__norank_	p__Firmicu	c__Clostrid	o__Clostrid	f__Peptocc	g__norank_	s__unclassi	OTU1187
d__Bacteri	k__norank_	p__Proteob	c__Gamma	o__Oceano	f__Halomo	g__Halomo	s__unclassi	OTU1747
d__Bacteri	k__norank_	p__Proteob	c__Deltapr	o__Oligofle	f__Oligofle	g__Silvanig	s__unclassi	OTU2195
d__Bacteri	k__norank_	p__Proteob	c__Gamma	o__Enterob	f__Enterob	g__Provide	s__unclassi	OTU2321
d__Bacteri	k__norank_	p__Proteob	c__Gamma	o__Betapro	f__Methylo	g__Methylo	s__unclassi	OTU3812
d__Bacteri	k__norank_	p__Bactero	c__Bactero	o__Chitino	f__Chitinop	g__Flavisol	s__uncultu	OTU1292
d__Bacteri	k__norank_	p__Proteob	c__Alphapr	o__Sphingo	f__Sphingo	g__Sphingo	s__unident	OTU518
d__Bacteri	k__norank_	p__Patescil	c__Sacchar	o__Sacchar	f__norank_	g__norank_	s__uncultu	OTU1402
d__Bacteri	k__norank_	p__Verruco	c__Verruco	o__Verruco	f__Rubrital	g__Luteolib	s__uncultu	OTU3791
d__Bacteri	k__norank_	p__Firmicu	c__Clostrid	o__Clostrid	f__Peptost	g__Filifacto	s__Filifacto	OTU1544
d__Bacteri	k__norank_	p__Bactero	c__Bactero	o__Flavoba	f__Flavoba	g__Aquibac	s__uncultu	OTU1634
d__Bacteri	k__norank_	p__Chlamy	c__Chlamy	o__Chlamy	f__cvE6	g__norank_	s__uncultu	OTU2578
d__Bacteri	k__norank_	p__Proteob	c__Gamma	o__Diploric	f__Diploric	g__Aquicel	s__uncultu	OTU1766
d__Bacteri	k__norank_	p__Proteob	c__Gamma	o__Oceano	f__Pseudol	g__Pseudo	s__metage	OTU1130
d__Bacteri	k__norank_	p__Firmicu	c__Clostrid	o__Clostrid	f__Rumino	g__Rumino	s__metage	OTU2508
d__Bacteri	k__norank_	p__Chlamy	c__Chlamy	o__Chlamy	f__Parachla	g__Neochla	s__metage	OTU990
d__Bacteri	k__norank_	p__Proteob	c__Gamma	o__Betapro	f__Rhodoc	g__Sulfurit	s__unclassi	OTU684
d__Bacteri	k__norank_	p__Bactero	c__Bactero	o__Chitino	f__Saprosp	g__Phaeod	s__uncultu	OTU1470
d__Bacteri	k__norank_	p__Proteob	c__Gamma	o__Legione	f__Legione	g__Legione	s__uncultu	OTU2525
d__Bacteri	k__norank_	p__Proteob	c__Gamma	o__Xantho	f__Xanthor	g__Luteimo	s__unclassi	OTU704
d__Bacteri	k__norank_	p__Bactero	c__Bactero	o__Chitino	f__Saprosp	g__unclass	s__unclassi	OTU2014
d__Bacteri	k__norank_	p__Depend	c__Babelia	o__Babelia	f__unclassi	g__unclass	s__unclassi	OTU2184
d__Bacteri	k__norank_	p__Bactero	c__Bactero	o__Bactero	f__Barnesi	g__norank_	s__uncultu	OTU1024
d__Bacteri	k__norank_	p__Proteob	c__Alphapr	o__Ricketts	f__Anaplas	g__Wolbac	s__unclassi	OTU1944
d__Bacteri	k__norank_	p__Proteob	c__Deltapr	o__Bdellov	f__Bdellovi	g__OM27_	s__uncultu	OTU2027
d__Bacteri	k__norank_	p__Depend	c__Babelia	o__Babelia	f__Vermipl	g__norank_	s__unclassi	OTU2222
d__Bacteri	k__norank_	p__Synergi	c__Synergi	o__Synergi	f__Synergis	g__Fretibac	s__unclassi	OTU1425
d__Bacteri	k__norank_	p__Bactero	c__Bactero	o__Flavoba	f__Flavoba	g__Flavoba	s__unclassi	OTU1353
d__Bacteri	k__norank_	p__Proteob	c__Gamma	o__Betapro	f__Nitrosor	g__Ellin606	s__uncultu	OTU1845
d__Bacteri	k__norank_	p__Proteob	c__Gamma	o__unclass	f__unclassi	g__unclass	s__unclassi	OTU1778
d__Bacteri	k__norank_	p__Firmicu	c__Bacilli	o__Bacillal	f__Staphylo	g__Staphyl	s__unclassi	OTU111
d__Bacteri	k__norank_	p__Depend	c__Babelia	o__Babelia	f__unclassi	g__unclass	s__unclassi	OTU2422
d__Bacteri	k__norank_	p__Actinob	c__Actinob	o__Corynel	f__Coryneb	g__Corynel	s__Coryneb	OTU1994
d__Bacteri	k__norank_	p__Bactero	c__Bactero	o__Bactero	f__Tannere	g__Macellil	s__uncultu	OTU1233
d__Bacteri	k__norank_	p__Proteob	c__Gamma	o__Pseudo	f__Moraxe	g__Alkanin	s__uncultu	OTU2513
d__Bacteri	k__norank_	p__Proteob	c__Gamma	o__Oceano	f__Pseudol	g__Pseudo	s__uncultu	OTU2264
d__Bacteri	k__norank_	p__Depend	c__Babelia	o__Babelia	f__UBA124	g__norank_	s__unclassi	OTU2827

d__Bacteri	k__norank_	p__Synergi	c__Synergis	o__Synergi	f__Synergis	g__Synergi	s__uncultu	OTU2704
d__Bacteri	k__norank_	p__Firmicu	c__Clostrid	o__Clostrid	f__Clostridi	g__unclass	s__unclassi	OTU544
d__Bacteri	k__norank_	p__Depenc	c__Babelia	o__Babelia	f__Vermipl	g__norank_	s__uncultu	OTU2357
d__Bacteri	k__norank_	p__Cyanob	c__Sericytc	o__norank_	f__norank_	g__norank_	s__uncultu	OTU1603
d__Bacteri	k__norank_	p__Chlamy	c__Chlamy	o__Chlamy	f__Parachla	g__unclass	s__unclassi	OTU2412
d__Bacteri	k__norank_	p__Proteol	c__Deltapr	o__Myxocc	f__Haliangi	g__Haliang	s__unclassi	OTU3102
d__Bacteri	k__norank_	p__Chlamy	c__Chlamy	o__Chlamy	f__Parachla	g__Candida	s__metage	OTU1416
d__Bacteri	k__norank_	p__Proteol	c__Alphapr	o__Rhizobi	f__Rhizobia	g__Phreatc	s__unclassi	OTU1960
d__Bacteri	k__norank_	p__Proteol	c__Gamma	o__Betaprc	f__Burkhol	g__Comam	s__Ottowia	OTU1534
d__Bacteri	k__norank_	p__Acidoba	c__Acidoba	o__Subgroı	f__norank_	g__norank_	s__uncultu	OTU2486
d__Bacteri	k__norank_	p__Proteol	c__Gamma	o__Legione	f__Legione	g__Legione	s__unclassi	OTU2320
d__Bacteri	k__norank_	p__Depenc	c__Babelia	o__Babelia	f__Vermipl	g__norank_	s__uncultu	OTU1838
d__Bacteri	k__norank_	p__Depenc	c__Babelia	o__Babelia	f__unclassi	g__unclass	s__unclassi	OTU2166
d__Bacteri	k__norank_	p__Chlamy	c__Chlamy	o__Chlamy	f__unclassi	g__unclass	s__unclassi	OTU2466
d__Bacteri	k__norank_	p__Proteol	c__Gamma	o__Alteron	f__Pseudoa	g__Pseudo	s__Pseudoa	OTU2752
d__Bacteri	k__norank_	p__Proteol	c__Gamma	o__Betaprc	f__Nitrosor	g__Ellin606	s__uncultu	OTU2444
d__Bacteri	k__norank_	p__Cyanob	c__Oxypho	o__Chlorop	f__norank_	g__norank_	s__unclassi	OTU1497
d__Bacteri	k__norank_	p__Depenc	c__Babelia	o__Babelia	f__unclassi	g__unclass	s__unclassi	OTU2590
d__Bacteri	k__norank_	p__Firmicu	c__Bacilli	o__Bacillal	f__Bacillac	g__Oceano	s__unclassi	OTU1526
d__Bacteri	k__norank_	p__Firmicu	c__Bacilli	o__Bacillal	f__Bacillac	g__Bacillus	s__unclassi	OTU1550
d__Bacteri	k__norank_	p__Proteol	c__Gamma	o__Xantho	f__Xanthor	g__Silanim	s__uncultu	OTU608
d__Bacteri	k__norank_	p__Proteol	c__Gamma	o__Betaprc	f__Burkhol	g__unclass	s__unclassi	OTU2759
d__Bacteri	k__norank_	p__Fusoba	c__Fusoba	o__Fusoba	f__Leptotri	g__Leptotr	s__unclassi	OTU698
d__Bacteri	k__norank_	p__unclass	c__unclassi	o__unclass	f__unclassi	g__unclass	s__unclassi	OTU1862
d__Bacteri	k__norank_	p__Bacterc	c__Bactero	o__Cytoph	f__Microsc	g__OLB12	s__uncultu	OTU1022
d__Bacteri	k__norank_	p__Chlorof	c__Chlorof	o__Chlorof	f__Roseifle	g__norank_	s__unclassi	OTU597
d__Bacteri	k__norank_	p__Depenc	c__Babelia	o__Babelia	f__unclassi	g__unclass	s__unclassi	OTU2163
d__Bacteri	k__norank_	p__Firmicu	c__Bacilli	o__Lactoba	f__Carnoba	g__Carnob	s__Carnoba	OTU2514
d__Bacteri	k__norank_	p__Firmicu	c__Clostrid	o__Clostrid	f__Clostridi	g__Fontice	s__unclassi	OTU1018

HS1	HS7	HS8	HS2	HS3	HS4	HS5	HS6	SUM
0	0	0	0	0	0	0	3	3
0	0	0	0	0	0	0	6	6
0	2	0	0	0	0	0	7	9
0	0	0	0	0	0	0	5	5
0	0	90	0	18	164	0	0	272
0	0	3	0	0	0	0	0	3
1	2	283	0	36	82	0	8	412
0	0	0	0	0	56	0	27	83
0	0	0	0	0	0	0	3	3
0	1	0	0	0	0	0	2	3
0	0	0	0	0	0	0	9	9
0	1	0	0	0	0	0	0	1
0	0	0	0	0	0	0	19	19
0	0	0	0	0	0	0	7	7
0	0	3	0	31	0	0	0	34
0	0	1	0	0	0	0	6	7
0	0	0	0	0	0	0	19	19
0	0	62	0	6	18	0	2	88
0	0	1	0	3	0	0	0	4
0	0	40	0	0	0	0	28	68
0	0	0	0	0	0	0	6	6
0	0	0	0	0	0	0	12	12
0	0	0	0	0	0	0	7	7
0	0	0	0	0	0	1	5	6
0	0	0	0	3	0	0	0	3
0	0	0	0	0	0	0	6	6
0	1	15	0	4	7	0	0	27
0	0	0	0	0	0	0	6	6
0	0	0	0	0	0	0	9	9
0	0	46	0	0	0	0	0	46
0	0	0	0	0	6	0	0	6
0	0	0	0	5	0	0	0	5
0	0	0	0	0	0	0	7	7
0	3	0	0	0	0	0	53	56
0	2	263	0	119	0	0	20	404
0	0	2	0	4	0	0	0	6
0	0	0	0	0	0	0	4	4
0	0	20	0	0	0	0	0	20
0	0	0	0	0	0	0	10	10
0	0	41	0	0	0	0	1	42
0	0	0	0	0	0	0	13	13
0	0	0	0	0	0	0	4	4
0	0	0	0	0	35	0	0	35
0	0	0	0	0	0	0	3	3
0	0	14	0	0	0	0	0	14
0	0	0	0	1	5	0	6	12

0	0	0	0	1	0	0	0	1
0	0	0	0	0	5	0	0	5
0	0	0	0	0	0	0	4	4
0	0	0	0	0	0	0	27	27
0	1	0	0	0	0	0	38	39
0	0	0	0	0	0	0	2	2
0	0	0	0	0	0	0	32	32
0	3	56	1	25	1197	0	61	1343
0	0	0	0	0	0	0	1	1
0	5	184	0	10	0	0	3	202
0	0	0	0	0	0	0	2	2
0	0	0	0	0	0	0	7	7
0	0	0	0	0	0	0	6	6
0	0	0	0	0	0	0	9	9
0	0	0	0	0	0	0	6	6
0	0	7	0	0	0	0	0	7
0	1	0	0	0	0	0	1	2
0	0	0	0	0	0	0	1	1
0	0	0	0	0	0	0	5	5
0	0	0	0	0	0	0	6	6
0	1	10	1	74	0	0	0	86
0	0	204	0	0	129	0	295	628
0	0	0	0	0	0	0	12	12
0	0	0	0	0	0	0	19	19
0	0	0	0	0	0	0	10	10
0	0	0	0	0	0	0	1	1
0	0	0	0	0	0	0	4	4
0	0	0	0	0	0	0	4	4
1	15	611	2	68	671	2	45	1415
0	0	0	0	0	0	0	4	4
0	0	4	0	1	15	0	0	20
0	0	0	0	0	0	0	6	6
0	0	0	0	0	0	0	9	9
0	0	0	0	0	0	0	6	6
0	0	25	0	0	0	0	0	25
0	0	0	0	0	14	0	0	14
0	0	0	0	0	0	0	16	16
0	0	0	0	0	0	0	7	7
0	0	45	0	1	0	0	1	47
0	1	0	1	20	140	0	18	180
0	27842	85	0	0	0	17	0	27944
0	0	0	0	0	0	0	2	2
0	0	0	0	0	0	0	12	12
0	0	0	0	0	0	0	17	17
0	0	0	0	0	0	0	2	2
0	0	0	0	0	0	0	10	10
0	0	16	0	0	0	0	1	17

6222	0	16	16015	6	63	710	51	23083
0	0	0	0	0	0	0	5	5
0	0	0	0	0	0	0	3	3
0	0	0	0	0	0	0	4	4
1	0	0	1	0	0	0	0	2
0	0	0	0	0	380	0	1	381
0	0	58	0	0	0	0	0	58
0	0	0	0	0	34	0	0	34
0	0	1	0	0	0	0	4	5
0	0	0	0	0	0	0	4	4
0	0	0	0	0	0	0	8	8
0	0	0	0	0	0	0	25	25
0	0	67	0	0	0	0	0	67
268	0	0	112	0	0	0	0	380
52	0	0	16	10	0	0	0	78
0	0	0	0	0	0	0	1	1
0	0	0	0	0	5	0	0	5
0	0	0	0	0	0	0	157	157
0	0	3	0	0	0	0	0	3
0	1	0	0	0	0	0	0	1
0	0	0	0	0	0	0	1	1
0	0	0	0	0	0	0	9	9
0	0	0	0	0	0	0	9	9
0	0	0	0	0	0	0	5	5
0	0	0	0	0	0	0	10	10
0	0	5	0	0	0	0	0	5
0	0	0	0	0	0	0	4	4
0	0	0	0	0	0	0	9	9
0	0	14	0	0	0	0	0	14
0	0	0	0	0	0	0	7	7
0	0	0	0	0	0	0	2	2
0	1	0	0	0	0	0	0	1
0	0	0	0	0	0	0	13	13
0	0	26	0	1	0	0	0	27
0	1	35	0	11	26	0	4	77
0	0	0	0	0	107	0	27	134
0	2	0	0	0	0	0	43	45
0	0	0	0	0	0	0	12	12
0	0	39	0	0	1	0	0	40
0	0	0	0	0	0	0	1	1
0	4	0	0	0	9	0	0	13
0	0	0	0	0	0	0	20	20
0	0	23	0	0	0	0	0	23
0	0	0	1	0	0	0	0	1
0	1	0	0	0	0	0	23	24
0	0	3	0	0	0	0	0	3
0	0	11	1	2	0	0	0	14

0	0	5	0	0	0	0	0	5
0	0	92	0	8	51	0	3	154
0	0	17	0	0	0	0	0	17
0	0	15	0	0	0	0	0	15
0	0	25	0	0	0	0	0	25
0	0	8	0	0	0	0	0	8
0	0	0	0	0	0	0	4	4
0	0	0	0	0	0	0	5	5
0	0	0	0	0	0	0	49	49
0	0	0	0	0	0	0	39	39
0	0	0	0	0	0	0	3	3
0	0	0	0	0	0	0	10	10
0	0	0	0	0	0	0	12	12
0	3	0	0	0	0	0	7	10
0	0	0	0	0	0	0	18	18
0	0	0	0	0	0	0	5	5
0	0	25	0	0	0	0	0	25
0	0	0	0	0	0	0	6	6
0	0	0	0	0	33	0	0	33
0	0	0	0	0	0	0	9	9
0	0	0	0	0	0	0	8	8
0	0	0	0	0	0	0	26	26
0	1	18	0	1	0	0	0	20
0	0	0	0	0	0	0	10	10
0	0	22	0	19	0	0	0	41
0	0	0	0	0	0	0	3	3
0	0	0	0	0	0	0	3	3
0	0	0	0	0	0	0	8	8
0	0	0	0	0	0	0	4	4
0	0	0	0	0	0	0	6	6
0	0	0	0	0	0	0	7	7
0	0	0	0	0	4	1	0	5
0	0	0	0	0	46	0	0	46
0	0	0	0	2	0	0	0	2
0	0	0	0	0	20	0	0	20
0	1	0	0	0	0	0	223	224
0	0	16	1	1	23	0	0	41
0	1442	90	0	29	146	53037	255	54999
0	0	0	0	0	0	0	7	7
0	0	0	0	0	0	0	12	12
0	1	293	0	151	0	0	12	457
0	0	0	0	0	0	0	5	5
0	0	0	0	0	0	1	9	10
0	0	0	0	5	8	0	21	34
0	0	3	0	0	0	0	0	3
0	0	0	0	0	0	0	11	11
0	0	23	0	1	0	0	0	24

0	0	0	0	0	18	0	0	18
0	1	0	0	0	0	0	8	9
0	0	0	0	3	0	0	0	3
0	0	0	0	0	0	0	4	4
0	0	0	0	0	0	0	17	17
0	0	0	0	0	0	0	2	2
0	1	0	0	0	0	0	45	46
0	2	0	0	0	0	0	9	11
0	0	4	0	1	26	0	0	31
0	0	0	0	0	0	0	33	33
0	4	305	1	7	185	0	12	514
0	1	0	0	0	0	0	2	3
0	0	0	0	0	0	0	31	31
0	0	0	0	0	0	0	3	3
854	3	18	8	3	13	0	0	899
0	1	0	0	0	0	0	10	11
0	0	0	0	0	24	0	11	35
0	0	4	0	6	36	0	0	46
0	37	1294	9	2387	0	0	64	3791
0	1	0	0	0	0	0	0	1
0	0	0	0	5	0	0	0	5
0	1	0	0	0	0	0	0	1
0	0	8	0	0	0	0	0	8
0	0	0	0	0	0	0	4	4
0	0	30	0	0	0	0	0	30
0	0	52	0	0	25	0	0	77
0	0	0	0	0	0	0	25	25
0	0	0	0	0	0	0	2	2
0	0	0	0	0	0	0	15	15
0	0	164	0	44	82	1	64	355
0	0	0	0	0	0	0	6	6
0	0	0	0	0	0	0	18	18
0	0	20	0	0	0	0	0	20
0	0	0	0	0	0	0	3	3
0	0	0	0	0	0	0	5	5
0	0	0	0	0	111	0	20	131
0	0	0	0	0	0	0	2	2
0	0	0	0	0	0	0	6	6
0	0	0	0	0	0	0	4	4
0	0	1	0	0	0	0	0	1
0	3	0	0	0	0	0	0	3
0	1	0	0	0	0	0	2	3
0	0	0	0	0	0	0	14	14
0	0	0	0	0	0	0	13	13
0	1	0	0	0	0	0	72	73
0	0	4	0	3	7	0	0	14
0	0	0	0	0	3	0	0	3

0	0	0	0	0	0	0	12	12
0	0	6	0	0	0	0	0	6
0	0	0	0	0	0	0	3	3
0	1	1	0	0	0	0	0	2
0	0	0	0	0	15	0	20	35
0	0	0	0	0	0	0	42	42
0	0	0	0	0	0	0	93	93
0	0	0	0	2	0	0	0	2
0	0	0	0	0	0	0	5	5
3	1	212	1	42	42	0	2	303
0	0	0	0	0	0	0	6	6
0	0	0	0	0	0	0	21	21
0	0	0	0	0	0	0	5	5
0	0	0	0	0	0	0	20	20
0	0	0	0	0	0	0	5	5
0	0	0	0	0	0	0	3	3
0	0	0	0	0	0	0	4	4
0	0	0	0	0	0	0	9	9
0	0	0	0	0	0	0	2	2
0	0	0	0	0	0	0	5	5
0	0	12	0	0	0	0	1	13
0	0	69	0	1	0	0	0	70
0	0	0	0	0	1	0	0	1
0	0	21	0	0	0	0	0	21
0	0	0	0	0	0	0	10	10
0	0	0	0	0	0	0	13	13
0	0	20	0	0	0	0	0	20
0	2	0	0	0	0	0	10	12
0	0	0	0	0	0	0	15	15
0	3	0	0	0	0	0	0	3
0	0	0	0	0	0	0	10	10
0	0	0	0	0	0	0	7	7
0	0	12	0	0	0	0	0	12
0	0	0	0	0	0	0	25	25
0	0	19	0	5	3	0	0	27
0	0	0	0	0	0	0	6	6
0	1	41	0	20	0	0	0	62
0	0	0	0	1	227	0	16	244
0	0	0	0	0	0	0	6	6
0	0	0	0	3	0	0	3	6
0	1	0	0	0	0	0	0	1
0	0	0	0	0	0	0	10	10
0	0	0	0	0	0	0	36	36
0	1	0	0	0	0	0	8	9
0	0	0	0	0	0	0	14	14
0	0	0	0	0	1	0	10	11
0	0	0	0	0	0	0	11	11

0	0	0	0	0	0	0	11	11
0	0	0	0	0	0	0	2	2
0	0	0	0	0	0	0	3	3
0	0	0	0	0	0	0	3	3
0	0	0	0	0	0	0	2	2
0	0	0	0	0	0	0	9	9
0	0	0	0	0	0	0	6	6
0	0	0	0	0	0	0	4	4
0	1	0	0	0	0	0	23	24
0	0	0	0	0	0	0	12	12
0	0	0	0	0	0	0	6	6
0	0	0	0	0	0	0	6	6
0	0	12	0	0	0	0	0	12
0	0	0	0	0	0	0	30	30
0	1	360	2	270	158	0	0	791
0	0	2	0	0	0	0	18	20
0	0	0	0	0	0	0	7	7
0	0	50	0	18	1	0	1	70
0	3	0	0	0	0	0	2	5
0	0	4	0	0	0	0	6	10
0	0	1	0	0	0	0	6	7
0	0	0	0	0	49	0	0	49
0	1	0	0	0	0	0	19	20
0	0	56	0	0	0	0	18	74
0	0	50	0	37	0	0	0	87
0	0	0	0	0	0	0	14	14
0	1	0	0	0	0	0	13	14
0	0	6	0	3	0	0	0	9
0	0	0	0	0	0	0	10	10
0	0	0	0	0	0	0	5	5
0	0	0	0	0	0	0	26	26
0	0	0	0	0	0	0	4	4
0	0	0	0	0	0	0	9	9
0	0	0	0	6	0	0	0	6
0	2	0	0	0	0	0	0	2
0	0	15	0	0	0	0	0	15
0	0	4	0	1	0	0	0	5
0	0	0	0	0	0	0	5	5
0	0	0	0	0	0	0	8	8
0	0	0	0	0	0	0	3	3
0	0	0	0	0	0	0	11	11
0	0	0	0	0	0	0	3	3
0	0	0	0	0	0	0	4	4
0	0	0	0	0	6	0	0	6
0	0	0	0	0	0	0	8	8
0	0	26	0	0	0	0	0	26
0	0	66	0	13	8	0	0	87

0	0	0	0	0	0	0	6	6
0	0	0	0	0	0	0	12	12
0	0	0	0	0	0	0	313	313
0	1	82	0	94	0	0	0	177
1	0	0	0	0	3	0	0	4
0	0	0	0	0	0	0	44	44
0	0	0	0	0	0	0	5	5
0	0	0	0	0	0	0	7	7
0	0	1	0	3	0	0	0	4
0	0	5	0	0	51	0	0	56
0	0	0	0	2	0	0	0	2
0	0	0	0	0	0	0	9	9
0	0	0	0	0	0	0	16	16
0	0	0	0	0	0	0	4	4
0	0	0	0	0	0	0	4	4
0	0	0	0	0	0	0	5	5
0	0	0	0	0	0	0	9	9
0	0	105	0	0	0	0	0	105
0	0	56	0	1	59	0	6	122
0	0	0	0	0	0	0	18	18
0	0	5	0	0	0	0	0	5
0	0	0	0	0	0	0	17	17
0	1	0	0	0	0	0	0	1
0	0	0	0	0	0	0	2	2
0	0	0	0	0	0	0	5	5
0	0	0	0	0	0	0	6	6
0	0	7	0	0	0	2	298	307
0	0	52	0	85	25	0	0	162
2	4	210	0	76	218	0	19	529
0	0	2	0	0	0	0	0	2
0	0	77	0	1	0	0	0	78
0	0	0	0	0	0	0	62	62
0	0	7	0	0	0	0	0	7
0	0	27	0	0	0	0	49	76
0	0	0	0	0	0	0	3	3
0	0	0	0	0	0	0	10	10
0	0	0	0	0	0	0	4	4
0	0	0	0	0	0	0	3	3
0	0	0	0	0	0	0	16	16
0	0	0	0	0	0	0	7	7
0	0	0	0	0	0	0	11	11
0	0	0	0	0	0	0	2	2
0	0	0	0	0	25	0	0	25
0	0	13	0	7	0	0	0	20
0	0	0	0	0	0	0	9	9
0	0	0	0	0	0	0	11	11
0	0	0	0	0	0	0	9	9

0	0	0	0	0	0	0	4	4
0	0	4	0	10	0	0	0	14
0	0	11	0	0	16	0	0	27
0	0	0	0	0	0	0	5	5
0	0	0	0	0	0	0	14	14
0	0	14	0	0	0	0	0	14
0	0	0	0	0	0	0	4	4
0	0	38	0	0	0	0	0	38
0	0	9	0	3	0	0	0	12
0	0	0	0	0	0	0	42	42
0	0	0	0	0	0	0	3	3
0	0	0	0	0	0	0	5	5
0	1	20	0	0	0	0	0	21
0	0	0	0	0	0	0	3	3
0	0	0	0	0	0	0	2	2
0	0	6	0	0	0	0	0	6
0	0	0	0	0	0	0	3	3
0	0	0	0	0	0	1	0	1
0	0	0	0	0	0	0	15	15
0	0	0	0	0	0	0	6	6
0	0	11	0	0	15	0	0	26
0	0	0	0	0	0	0	28	28
0	0	0	0	0	0	1	0	1
0	0	31	0	5	12	0	11	59
0	0	16	0	10	0	0	0	26
0	0	0	0	0	73	0	0	73
0	0	0	0	0	0	0	12	12
0	1	28	0	0	0	0	0	29
0	0	0	0	0	0	0	10	10
0	0	0	0	0	0	0	3	3
0	0	0	0	1	0	0	0	1
0	0	0	0	0	0	0	10	10
0	0	4	0	0	0	0	0	4
0	0	8	0	0	0	0	2	10
0	0	0	0	0	0	0	3	3
0	0	0	0	1	0	0	0	1
0	0	0	0	2	0	0	0	2
0	0	11	0	11	0	0	0	22
0	0	3	0	0	0	0	39	42
0	0	0	1	8	0	0	0	9
0	1	0	0	0	0	0	2	3
0	0	0	0	0	0	0	11	11
0	0	0	0	0	0	0	11	11
0	2	0	0	0	0	0	6	8
0	0	0	0	0	7	0	0	7
0	0	8	0	1	3	0	0	12
0	1	0	0	0	0	0	122	123

0	0	0	0	0	0	0	6	6
0	0	0	0	12	0	1395	13	1420
0	2	0	0	0	1	0	182	185
0	0	0	0	0	0	0	24	24
0	0	37	0	0	0	0	0	37
0	0	0	0	0	0	0	11	11
0	5	0	0	0	0	0	78	83
0	0	0	0	0	0	0	9	9
0	0	0	0	0	0	0	8	8
0	0	0	0	0	0	0	3	3
0	0	0	0	0	0	0	3	3
0	0	0	0	0	14	0	0	14
0	0	0	0	0	0	0	31	31
0	0	0	0	5	542	0	3	550
0	0	38	0	0	0	0	0	38
0	0	0	0	0	0	1	27	28
0	0	0	0	0	0	0	5	5
5	1	223	2	350	175	0	27	783
0	0	6	0	1	0	0	0	7
0	0	0	0	0	0	0	2	2
0	0	0	0	0	0	0	49	49
0	0	0	0	0	0	0	8	8
0	0	0	0	0	0	0	8	8
0	0	56	0	7	0	0	0	63
0	0	0	0	0	0	0	3	3
0	0	0	0	0	0	0	7	7
0	0	0	0	0	0	0	9	9
0	0	2	0	0	19	0	0	21
0	0	0	21	0	0	0	0	21
0	0	0	0	0	0	0	10	10
0	0	0	0	0	0	0	13	13
0	0	0	0	0	0	0	2	2
0	0	0	0	0	0	0	2	2
0	0	0	0	0	0	0	10	10
0	0	0	0	0	0	0	4	4
0	0	0	0	0	0	0	7	7
0	0	5	0	0	0	0	0	5
0	0	0	0	6	0	0	3	9
1	9	194	0	73	362	0	33	672
0	0	0	0	0	0	0	4	4
0	1	8	0	31	32	0	0	72
0	0	0	0	0	0	0	4	4
0	0	0	0	0	0	0	5	5
0	0	8	0	0	0	0	0	8
0	0	18	0	0	0	0	0	18
0	0	12	0	4	0	0	0	16
0	0	0	0	4	0	0	0	4

0	0	0	0	0	0	0	3	3
0	0	3	0	0	0	0	5	8
0	0	8	0	5	3	0	1	17
0	0	0	0	0	50	0	0	50
0	0	0	0	2	0	0	0	2
0	0	0	0	0	0	0	15	15
0	4	0	0	0	0	0	1	5
0	0	3	0	1	0	0	0	4
0	0	51	0	0	0	0	78	129
0	0	0	0	0	0	0	8	8
0	0	0	0	3	0	0	0	3
0	0	7	0	0	0	1	61	69
0	1	1	0	0	0	0	1	3
0	0	17	0	1	0	0	0	18
0	0	0	0	0	0	0	3	3
0	0	0	0	0	0	0	4	4
0	0	0	0	0	0	0	19	19
0	0	0	0	0	0	0	6	6
0	0	3	0	4	0	0	0	7
0	0	13	0	7	0	0	1	21
0	0	0	0	0	0	0	7	7
0	0	0	0	0	0	0	1	1
0	0	0	0	0	0	0	11	11
0	0	0	1	0	0	0	0	1
0	0	0	0	0	0	0	9	9
0	2	23	0	15	0	0	1	41
0	0	0	0	0	0	0	21	21
0	0	0	0	0	0	0	5	5
0	0	0	0	0	0	0	2	2
0	0	0	0	0	21	0	8	29
1	0	0	0	0	0	0	11	12
0	0	0	0	0	0	0	10	10
0	0	0	0	1	0	0	0	1
0	2	10	0	0	0	0	0	12
0	0	0	0	0	0	0	6	6
0	0	0	0	0	0	0	15	15
0	0	8	0	0	0	0	0	8
0	0	0	0	0	4	0	0	4
0	0	0	0	0	0	0	222	222
1	2	208	0	0	107	0	30	348
0	0	0	0	0	0	0	1	1
0	0	0	0	0	0	0	6	6
0	0	0	0	0	0	0	24	24
0	5	0	0	0	0	0	42	47
0	0	0	0	0	0	0	3	3
0	0	20	0	0	0	0	4	24
0	0	25	0	0	0	0	0	25

0	0	9	0	0	0	0	4	13
0	0	25	0	0	0	0	0	25
0	0	0	0	0	0	0	5	5
0	0	0	0	0	0	0	17	17
0	4895	12	0	0	10	366	0	5283
0	6	262	0	2	42	0	7	319
0	0	0	0	0	0	0	13	13
0	1	0	0	0	0	0	0	1
0	0	0	0	0	0	0	5	5
0	0	0	0	0	0	0	14	14
0	0	0	0	0	0	0	3	3
0	0	48	0	0	9	0	11	68
0	0	0	0	0	0	0	17	17
0	0	0	0	0	0	0	23	23
0	0	21	0	0	0	0	0	21
0	0	8	0	0	0	0	0	8
0	0	42	0	0	0	0	0	42
0	1	8	0	6	0	0	8	23
0	0	34	0	0	0	0	0	34
0	0	0	0	0	0	0	19	19
0	0	0	0	0	0	0	2	2
0	0	0	0	0	0	0	6	6
0	0	52	0	0	0	0	5	57
0	0	0	0	0	0	0	16	16
0	0	0	0	0	0	0	6	6
0	0	0	0	5	3	0	0	8
1	0	0	0	0	0	0	10	11
0	0	0	0	0	0	0	8	8
0	0	0	0	0	0	0	3	3
0	0	0	0	4	0	0	0	4
0	0	0	0	0	0	0	7	7
0	0	2	0	0	0	0	0	2
0	0	0	0	0	0	0	6	6
0	0	0	0	0	0	0	14	14
0	0	0	0	0	0	0	3	3
2	2	291	2	94	111	0	2	504
0	1	0	0	0	169	0	45	215
0	0	0	0	6	0	0	0	6
0	0	0	0	0	0	0	7	7
0	0	0	0	0	0	0	2	2
0	0	3	0	0	0	0	5	8
0	0	12	0	3	117	0	9	141
0	0	0	0	0	0	0	7	7
0	0	28	0	3	8	0	5	44
0	0	0	0	0	0	0	9	9
0	0	0	0	0	0	0	6	6
0	0	0	0	0	0	0	24	24

0	0	0	0	0	0	0	3	3
0	0	0	0	0	0	0	3	3
0	0	0	0	0	561	0	4	565
0	0	7	0	0	0	0	0	7
0	0	0	0	0	0	0	11	11
0	0	20	0	17	1	0	0	38
0	0	0	0	0	0	0	3	3
0	0	2	0	0	0	0	0	2
0	0	0	0	0	67	0	0	67
0	0	0	0	0	0	0	7	7
0	0	0	0	0	0	0	182	182
0	0	0	0	0	0	0	23	23
0	0	0	0	0	12	0	0	12
0	0	0	0	0	0	0	4	4
0	79	2492	2	573	1976	0	781	5903
0	1	95	0	16	37	0	2	151
0	0	0	0	0	0	0	7	7
0	0	0	0	8	0	0	0	8
0	0	0	0	0	0	0	180	180
0	0	0	0	0	0	0	2	2
0	0	0	0	0	0	0	7	7
0	0	0	0	0	0	0	2	2
1	0	73	10	35	44	0	2	165
0	0	0	0	0	0	0	2	2
0	0	32	0	2	0	0	31	65
0	0	0	0	0	0	0	2	2
0	1	0	0	0	0	0	0	1
0	0	0	0	0	0	0	19	19
0	0	0	0	0	0	0	11	11
0	0	0	2	2	0	0	1	5
0	2	0	0	0	0	0	0	2
0	0	0	0	0	0	0	6	6
0	0	20	0	2	19	0	3	44
0	0	0	0	0	0	0	7	7
0	2	166	1	156	107	0	0	432
0	0	0	0	0	0	0	7	7
0	0	0	0	0	0	0	12	12
0	0	0	0	0	0	0	6	6
0	0	0	0	0	0	0	11	11
0	1	0	0	0	0	0	5	6
0	0	0	0	0	10	0	0	10
0	0	0	0	8	0	0	14	22
0	0	0	0	0	0	0	34	34
0	2	0	0	0	0	0	3	5
0	0	17	0	0	0	0	0	17
2	1	23	0	27	96	0	12	161
0	0	0	0	0	0	0	7	7

0	0	5	0	0	0	0	0	5
0	0	0	0	0	0	0	4	4
0	0	0	0	0	0	0	98	98
0	0	0	0	0	0	0	6	6
0	0	0	0	0	0	0	10	10
0	0	0	0	3	0	0	0	3
0	0	0	0	3	0	0	0	3
0	0	0	0	0	0	0	4	4
0	0	69	0	0	0	0	0	69
0	0	0	0	0	0	0	4	4
0	0	0	0	0	0	0	18	18
0	0	3	0	0	0	0	0	3
0	0	0	0	0	43	0	0	43
257	0	0	136	0	393	0	0	786
0	0	0	0	0	0	0	6	6
0	0	96	0	1	177	0	9	283
0	1	0	0	0	0	0	0	1
0	0	0	0	0	0	0	5	5
140	0	0	4	0	0	0	0	144
0	0	10	0	0	57	0	0	67
0	0	8	0	0	0	0	0	8
0	0	0	0	0	0	0	1	1
0	0	0	0	0	59	0	1	60
0	0	0	0	0	0	0	48	48
0	0	0	0	0	0	0	2	2
0	0	0	0	0	2	0	0	2
0	0	0	0	0	0	0	8	8
0	0	1	0	6	0	0	9	16
0	0	0	0	0	0	0	15	15
0	1	0	0	0	0	0	13	14
0	0	0	0	0	0	0	37	37
0	0	67	0	1	0	0	0	68
0	0	14	0	0	0	0	0	14
0	0	0	0	0	0	0	11	11
0	0	0	0	0	0	0	6	6
0	0	55	0	4	28	0	0	87
0	0	0	0	0	14	0	0	14
0	0	0	0	0	43	0	0	43
0	0	0	0	0	0	0	2	2
0	0	7	0	0	0	0	0	7
0	0	0	0	0	0	0	3	3
0	0	0	0	4	9	0	0	13
0	0	32	0	0	0	0	0	32
0	0	0	0	0	0	0	21	21
0	0	0	0	0	0	0	6	6
0	0	0	0	0	0	0	8	8
0	0	12	0	4	7	0	7	30

0	0	14	0	5	0	0	0	19
0	0	0	0	0	0	0	5	5
1	0	0	0	7	0	0	0	8
0	0	0	0	0	0	0	10	10
0	0	0	0	3	0	0	0	3
0	0	0	0	0	0	0	12	12
0	0	0	0	0	0	0	15	15
0	0	0	0	0	0	0	10	10
0	0	0	0	0	76	0	2	78
0	0	0	0	0	0	0	12	12
0	13	429	0	194	548	0	48	1232
0	0	0	0	0	0	0	3	3
0	0	0	0	0	0	0	23	23
0	0	0	0	0	0	0	4	4
0	1	5	0	17	113	0	24	160
11	10	1550	10	567	1009	1	97	3255
0	0	0	0	0	0	0	2	2
0	0	0	0	0	0	0	4	4
0	0	0	0	0	0	0	15	15
0	0	0	0	0	0	0	19	19
0	0	0	0	0	0	0	23	23
1	0	0	0	0	0	0	10	11
0	0	0	0	0	0	0	3	3
0	1	0	0	0	0	0	13	14
0	0	4	0	0	0	0	0	4
0	0	0	0	0	0	0	13	13
0	0	0	0	0	0	0	5	5
0	0	0	0	0	93	0	0	93
0	0	0	0	0	0	0	6	6
0	1	0	0	0	0	0	33	34
0	0	0	0	0	0	0	3	3
0	0	0	0	0	0	0	5	5
0	1	0	0	0	0	0	40	41
0	0	0	0	0	0	0	22	22
0	0	0	0	0	0	0	13	13
0	0	0	0	0	0	0	4	4
0	1	0	0	0	0	0	1	2
0	0	0	0	10	0	0	0	10
0	0	0	0	0	0	0	9	9
0	0	0	0	0	0	0	7	7
0	0	0	0	0	0	0	12	12
0	0	0	0	0	0	0	10	10
0	0	4	0	0	0	0	0	4
0	0	0	0	0	0	0	3	3
0	0	12	0	6	0	0	16	34
0	0	0	0	0	0	0	2	2
0	0	0	0	0	0	0	6	6

0	0	0	0	0	18	0	0	18
0	0	14	0	0	0	0	0	14
0	0	0	0	0	0	0	10	10
0	0	0	0	0	0	0	9	9
0	0	0	0	0	0	0	4	4
0	0	5	0	1	0	0	0	6
0	1	265	0	71	23	0	0	360
0	2	0	0	0	0	0	0	2
0	1	69	0	19	0	0	0	89
0	0	0	0	0	0	0	12	12
0	1	0	0	0	0	0	8	9
0	0	0	0	0	0	0	4	4
0	0	42	0	52	2	0	0	96
0	0	0	0	0	0	0	3	3
0	0	0	0	0	0	0	20	20
0	0	30	0	0	0	0	0	30
0	0	0	0	0	0	0	4	4
0	0	0	0	0	0	0	12	12
0	0	11	0	0	0	0	3	14
0	0	0	0	0	0	0	14	14
0	0	0	0	0	0	0	12	12
0	0	0	0	0	19	0	0	19
0	0	0	0	0	0	0	2	2
1	0	0	0	0	0	0	16	17
0	0	4	0	4	0	0	0	8
0	0	0	0	0	0	0	3	3
0	0	40	0	2	0	0	66	108
0	0	0	0	4	0	0	0	4
0	0	0	0	0	0	0	22	22
0	2	8	0	0	0	0	0	10
0	0	0	0	3	0	0	0	3
0	0	0	0	0	0	0	10	10
0	0	0	0	0	0	0	17	17
0	0	0	0	0	0	0	7	7
0	1	0	0	0	0	0	2	3
0	0	0	0	0	0	0	2	2
0	0	0	0	0	0	0	7	7
0	0	0	0	0	0	0	3	3
0	0	0	0	0	0	0	2	2
0	0	0	0	0	0	0	7	7
0	0	25	0	0	36	0	0	61
0	2	178	3	72	437	0	0	692
1	0	0	0	0	0	0	7	8
0	0	0	0	0	0	0	22	22
0	0	0	0	0	6	0	0	6
0	0	0	0	0	0	0	8	8
0	0	0	0	0	0	0	6	6

0	0	0	0	0	0	0	4	4
0	0	0	0	0	0	0	4	4
0	0	0	0	0	0	0	7	7
0	0	4	0	0	0	0	0	4
0	0	0	0	0	0	0	6	6
0	0	0	0	0	0	0	3	3
0	0	0	0	0	0	0	5	5
0	0	0	0	0	0	0	18	18
0	0	50	0	0	0	0	0	50
0	2	0	0	0	0	0	1	3
0	0	2	0	0	0	0	0	2
0	0	19	0	0	0	0	0	19
0	0	0	0	3	0	0	21	24
0	0	0	0	0	0	0	13	13
0	0	0	0	0	0	0	2	2
0	0	0	0	0	0	0	1	1
0	0	0	0	0	0	0	96	96
0	0	67	0	2	0	0	0	69
0	0	0	0	0	0	0	6	6
0	0	0	0	0	0	0	21	21
0	0	14	0	1	4	0	9	28
0	0	13	0	0	0	0	0	13
0	0	0	0	0	0	0	3	3
0	0	0	0	0	0	0	2	2
0	0	0	0	0	0	0	4	4
0	0	0	0	0	0	0	29	29
0	0	0	0	0	0	0	12	12
0	0	0	0	0	0	0	9	9
0	0	0	0	0	0	0	8	8
0	0	170	0	59	0	0	0	229
0	0	0	0	0	0	0	1	1
0	0	0	0	0	0	0	8	8
0	3	155	0	19	253	0	3	433
0	0	0	0	0	0	0	15	15
0	0	0	0	0	0	0	10	10
0	0	0	0	0	32	0	0	32
0	0	0	0	0	0	0	7	7
0	0	0	0	0	0	0	5	5
0	0	0	0	0	0	0	8	8
0	0	0	0	0	0	0	7	7
0	0	5	0	0	13	0	0	18
0	0	25	0	2	0	0	0	27
0	0	1	0	5	0	0	0	6
0	0	0	0	0	0	0	3	3
0	0	0	0	0	0	0	18	18
0	0	21	0	0	0	0	0	21
0	0	0	0	0	0	0	28	28

0	0	0	0	0	0	0	24	24
0	0	0	0	0	0	0	6	6
0	0	0	0	0	0	0	14	14
0	0	0	0	0	0	0	2	2
0	2	15	0	0	0	0	1	18
0	2	0	0	0	0	1	13	16
0	0	0	0	0	0	0	14	14
0	0	0	0	3	0	0	0	3
0	0	41	0	2	0	0	9	52
0	0	0	0	0	0	0	3	3
0	1	146	0	28	57	0	11	243
0	0	21	0	26	1	0	0	48
48151	14592	102	39538	27894	528	12	117	130934
0	0	0	0	0	0	0	5	5
0	0	0	0	0	0	0	2	2
0	0	0	0	0	0	0	14	14
0	0	0	1	1	0	0	0	2
0	0	0	0	0	0	0	12	12
0	0	15	0	1	21	0	0	37
0	2	0	0	0	0	0	188	190
0	0	0	0	0	0	0	12	12
0	0	0	0	0	0	0	10	10
0	0	0	0	0	0	0	9	9
0	0	14	0	1	0	0	0	15
0	0	0	0	0	0	69	0	69
0	0	0	0	0	0	0	19	19
0	0	3	0	1	0	0	0	4
0	0	0	0	0	0	0	3	3
0	0	0	0	0	0	0	14	14
0	0	0	0	0	0	0	9	9
0	0	0	0	0	0	0	6	6
0	0	0	0	0	0	0	2	2
0	1	0	0	0	0	0	4	5
0	0	0	0	0	0	0	2	2
0	0	0	0	0	0	0	5	5
0	2	0	0	0	0	0	91	93
0	0	0	0	0	0	0	10	10
0	0	0	0	0	0	0	5	5
0	0	5	0	0	0	0	0	5
0	0	0	0	0	0	0	25	25
0	1	0	0	0	0	0	0	1
0	0	0	0	0	0	0	2	2
1	3	437	0	208	0	0	0	649
0	0	0	0	0	0	0	1	1
0	0	0	0	6	0	0	0	6
0	0	0	0	0	0	0	27	27
0	0	0	0	0	0	0	6	6

0	0	16	0	0	0	0	7	23
0	1	96	0	2	280	0	55	434
0	1	0	0	0	0	0	7	8
0	0	0	0	0	0	0	17	17
0	0	0	0	0	0	0	289	289
0	0	0	0	0	0	0	14	14
0	1	207	0	35	0	1	0	244
0	0	27	0	0	0	0	0	27
0	0	0	0	0	0	0	12	12
0	0	185	0	5	15	0	0	205
0	0	0	0	0	0	0	19	19
0	0	0	0	0	0	0	9	9
0	0	0	0	0	30	0	0	30
0	0	32	0	0	0	0	3	35
0	0	0	0	0	0	0	4	4
0	0	0	0	0	0	0	4	4
0	0	0	0	0	0	0	13	13
0	0	0	0	0	48	0	0	48
1	0	0	0	0	0	0	18	19
0	0	3	0	0	22	0	2	27
0	0	0	0	0	0	0	1	1
0	1	0	0	0	0	0	5	6
0	2	0	0	0	0	0	8	10
0	0	0	0	0	0	0	98	98
0	0	0	0	0	0	0	17	17
0	0	0	0	0	0	0	94	94
8	85	2732	9	2545	1790	0	622	7791
0	1	60	0	16	0	0	0	77
0	0	0	0	0	0	0	13	13
0	0	0	0	0	0	0	8	8
0	0	18	0	0	0	0	0	18
0	0	97	0	0	0	0	4	101
0	0	0	0	0	16	0	28	44
0	0	0	0	0	0	0	7	7
0	0	0	0	0	0	0	7	7
0	0	0	0	0	0	0	10	10
0	0	0	0	0	3	0	1	4
0	0	0	0	0	0	0	4	4
0	0	0	0	0	0	0	3	3
0	0	0	0	0	0	0	4	4
0	0	0	0	0	0	0	25	25
0	0	8	0	5	0	0	0	13
0	0	38	1	6	36	0	24	105
0	0	0	0	0	0	0	8	8
0	0	0	0	0	0	0	8	8
0	0	156	0	11	61	0	5	233
0	0	0	0	0	0	0	3	3

0	1	3	0	0	8	0	0	12
0	0	0	0	0	0	0	12	12
0	0	0	0	0	0	0	13	13
0	0	0	0	0	0	0	2	2
0	0	0	0	0	0	0	2	2
0	1	0	0	0	0	0	26	27
0	0	15	0	16	0	0	0	31
0	0	28	0	0	0	0	0	28
0	0	0	0	0	0	0	3	3
0	1	0	0	0	0	0	8	9
0	0	53	0	0	0	0	65	118
0	0	0	0	0	0	0	8	8
0	0	49	0	1	2	0	0	52
0	1	0	0	0	0	0	11	12
0	0	0	0	0	17	0	14	31
0	0	0	0	0	0	0	3	3
0	1	0	0	0	0	0	20	21
0	0	0	0	0	0	0	2	2
0	0	0	0	0	53	0	0	53
0	0	0	0	0	0	0	5	5
0	0	0	0	0	0	0	13	13
0	0	0	0	0	0	0	2	2
0	0	0	0	0	0	0	13	13
0	0	12	0	88	0	0	0	100
0	0	10	0	2	0	0	0	12
0	0	49	0	21	120	0	4	194
0	0	2	0	0	0	0	0	2
0	0	0	141	0	0	0	0	141
0	4	0	0	0	0	0	23	27
0	0	0	0	0	0	0	4	4
0	0	0	0	0	0	0	11	11
0	0	7	0	0	21	0	0	28
0	0	0	0	0	0	0	8	8
0	0	0	0	0	0	0	11	11
0	0	18	0	2	0	0	3	23
0	0	0	0	0	0	0	22	22
0	0	0	0	0	0	0	4	4
0	0	0	0	0	0	0	1	1
0	1	0	0	0	0	0	0	1
0	0	0	1	4	36	0	0	41
0	0	0	0	0	0	0	12	12
0	0	10	0	0	0	0	0	10
0	0	0	0	0	0	0	1	1
0	0	37	0	0	0	0	1	38
0	0	2	0	0	0	0	0	2
0	1	0	0	5	17	0	0	23
0	1	0	0	0	0	0	52	53

0	0	54	0	30	0	0	0	84
0	0	2	0	0	0	0	0	2
0	1	0	0	0	127	0	11	139
0	0	0	0	0	0	0	14	14
0	0	0	0	0	0	0	2	2
0	30	1274	0	0	0	0	0	1304
0	1	0	0	0	0	0	29	30
0	0	0	0	0	0	0	14	14
0	0	0	0	0	0	0	14	14
0	0	9	0	1	22	0	0	32
0	0	0	0	0	27	0	0	27
0	0	0	0	0	0	0	10	10
0	1	0	0	1	0	0	108	110
0	1	2	0	10	34	0	0	47
0	0	5	0	0	0	0	0	5
0	3	65	0	3	3	0	21	95
0	0	0	0	0	0	0	3	3
0	0	0	0	0	0	0	16	16
0	0	0	0	0	0	0	6	6
0	1	41	0	9	64	0	3	118
0	0	0	0	0	0	0	37	37
0	0	4	0	0	0	0	6	10
0	0	0	0	0	0	0	4	4
0	0	0	0	0	0	0	13	13
0	0	18	0	4	105	0	3	130
0	0	11	2	3	0	0	0	16
0	1	0	0	0	0	0	0	1
0	0	0	0	0	0	0	31	31
0	0	0	0	4	0	0	0	4
0	0	0	1	0	118	0	5	124
0	0	0	0	0	7	0	0	7
0	0	0	0	0	0	0	5	5
0	0	6	0	0	0	0	0	6
1	1	246	2	42	737	1	10	1040
0	0	0	0	0	0	0	13	13
0	0	0	0	0	0	0	22	22
0	0	17	0	1	2	0	0	20
0	0	0	0	0	0	0	15	15
0	0	0	0	0	0	0	12	12
0	0	14	0	0	0	0	0	14
0	0	0	0	0	0	0	4	4
0	0	0	0	0	0	0	1	1
0	0	0	0	0	0	0	31	31
0	0	0	0	0	0	0	4	4
0	1	0	0	7	50	0	79	137
0	0	0	0	0	0	0	17	17
0	0	0	0	0	0	0	16	16

0	0	0	0	0	0	0	56	56
0	0	0	0	0	0	0	1	1
0	0	0	0	0	0	0	15	15
0	0	0	0	0	0	0	5	5
0	0	0	0	0	0	0	14	14
0	1	55	0	6	273	0	4	339
0	0	0	0	0	0	0	4	4
0	0	0	0	0	0	0	4	4
0	0	13	0	0	0	0	0	13
0	0	0	0	0	102	0	0	102
0	0	0	0	0	0	0	4	4
0	1	0	0	0	0	0	0	1
0	0	0	0	0	0	0	484	484
0	0	2	0	3	0	0	0	5
0	0	0	0	0	0	0	13	13
0	0	4	0	0	0	0	0	4
0	0	0	0	0	0	0	12	12
0	0	0	0	0	0	0	16	16
0	0	0	0	0	0	0	12	12
0	0	0	0	0	0	0	4	4
0	0	21	0	0	0	0	0	21
0	0	0	0	0	0	0	39	39
0	0	0	0	0	0	0	10	10
0	0	0	0	0	0	0	5	5
0	0	36	0	0	0	0	0	36
0	0	0	0	0	0	0	4	4
0	0	0	0	0	0	0	2	2
0	0	0	0	0	0	0	8	8
0	0	0	0	0	0	0	5	5
0	0	50	0	0	0	0	0	50
0	0	0	0	0	0	0	3	3
0	0	31	0	0	19	0	0	50
0	0	0	0	0	0	0	7	7
0	1	0	0	0	0	0	19	20
0	0	0	0	0	0	0	12	12
0	0	8	0	58	19	0	11	96
0	2	51	1	39	0	0	31	124
0	0	0	0	0	0	0	18	18
0	0	0	0	0	0	0	8	8
0	0	0	0	0	0	0	8	8
0	0	8	0	0	3	0	0	11
0	0	76	0	31	123	0	0	230
0	0	0	0	0	0	0	9	9
0	0	18	0	0	0	0	0	18
0	2	111	0	8	0	0	46	167
0	0	0	0	0	0	0	14	14
0	0	0	0	0	0	0	6	6

0	3	70	0	20	0	0	37	130
0	1	0	0	0	0	0	0	1
0	0	13	1	29	0	0	0	43
0	0	0	0	0	0	0	15	15
0	0	0	0	0	0	0	6	6
4	5	295	5	181	1164	0	331	1985
0	0	0	0	0	0	0	19	19
0	0	0	0	0	0	0	4	4
0	0	0	0	0	0	0	14	14
0	0	0	0	0	0	0	310	310
0	0	0	0	0	0	0	7	7
0	0	0	0	11	22	0	0	33
0	0	0	0	0	0	0	13	13
0	0	0	0	0	0	0	20	20
0	0	0	0	0	0	0	5	5
0	0	15	0	0	0	0	0	15
0	0	36	0	0	0	0	70	106
0	0	0	0	0	0	0	89	89
0	0	0	0	0	0	0	1	1
0	0	0	0	0	0	0	4	4
0	0	0	0	0	36	0	0	36
0	0	0	0	0	0	0	11	11
0	0	0	0	0	0	0	69	69
0	0	0	0	0	0	2	85	87
0	0	0	0	0	0	0	6	6
0	0	38	0	7	5	0	24	74
0	0	0	0	0	0	0	12	12
0	0	0	0	0	0	0	4	4
0	0	0	0	0	0	0	10	10
0	0	0	0	0	0	0	3	3
0	0	7	0	1	91	0	3	102
0	0	0	0	0	0	489	0	489
0	0	0	0	6	0	0	1	7
0	0	0	0	0	0	0	15	15
0	0	3	0	0	0	0	0	3
0	0	0	0	0	0	0	10	10
0	0	0	0	0	0	0	4	4
0	37	763	0	8706	1783	1	102	11392
0	1	0	0	0	0	0	7	8
0	0	0	0	0	0	0	10	10
1	4	90	1	12	129	0	11	248
0	0	0	0	3	0	0	0	3
0	0	0	0	4	0	0	0	4
0	0	0	0	0	0	0	6	6
0	0	0	0	0	0	0	4	4
0	0	0	0	0	0	0	4	4
0	0	0	0	0	0	0	23	23

0	0	0	0	0	0	0	14	14
0	0	0	0	0	0	0	10	10
0	0	0	0	0	0	0	11	11
0	0	32	0	0	0	0	0	32
0	0	0	0	0	0	0	10	10
0	0	0	0	0	0	0	38	38
0	0	36	0	25	0	0	5	66
0	0	0	0	0	0	0	6	6
0	0	55	0	16	47	0	6	124
0	0	6	0	0	0	0	1	7
0	0	0	0	0	0	0	13	13
0	0	0	0	0	0	0	29	29
1	0	39	0	43	210	0	9	302
0	2	46	0	11	0	0	0	59
0	2560	1	0	0	0	0	0	2561
0	0	31	0	0	0	0	0	31
0	0	0	0	0	0	0	8	8
0	0	0	0	0	0	0	29	29
0	0	0	0	0	29	0	16	45
0	0	0	0	0	0	0	4	4
0	0	0	0	0	0	0	14	14
0	0	2	0	0	0	0	0	2
0	1	0	0	0	0	0	5	6
0	0	0	0	0	0	0	6	6
0	0	0	0	0	8	0	0	8
0	1	50	0	2	8	0	0	61
0	0	0	0	0	0	0	4	4
0	0	8	0	0	0	0	0	8
0	0	2	0	13	0	0	0	15
0	0	0	0	0	0	0	4	4
0	0	68	0	0	0	0	19	87
0	2	0	0	0	0	0	56	58
0	0	0	0	0	0	0	16	16
0	0	12	0	25	0	0	0	37
0	0	0	0	0	0	0	7	7
0	0	0	0	0	0	0	33	33
0	0	0	0	0	0	0	4	4
0	0	0	0	0	0	0	20	20
0	0	0	0	0	0	0	5	5
0	0	0	0	0	0	0	7	7
0	0	0	0	0	37	0	0	37
0	0	0	0	0	0	0	8	8
0	0	0	0	0	0	0	1	1
0	0	0	0	0	0	0	20	20
0	1	449	0	168	0	0	16	634
0	0	0	0	0	0	0	2	2
0	0	0	0	0	0	0	2	2

0	0	0	0	0	0	0	11	11
0	0	0	0	0	0	0	6	6
0	0	56	0	0	0	0	0	56
0	0	0	0	0	0	0	6	6
0	0	0	0	0	0	0	2	2
0	4	271	2	21	126	0	44	468
0	0	0	0	0	0	0	5	5
0	0	0	0	0	0	0	6	6
0	0	290	0	0	0	0	4	294
0	1	0	0	0	0	0	0	1
0	0	61	0	0	0	0	0	61
0	0	0	0	4	0	0	0	4
0	0	0	0	0	0	0	8	8
0	0	0	0	0	0	0	2	2
0	1	11	1	21	0	0	0	34
0	0	2	0	0	0	0	0	2
0	0	0	0	0	0	0	3	3
0	0	19	0	6	10	0	0	35
0	1	0	0	1	0	0	4	6
0	0	0	0	0	0	0	4	4
0	0	7	0	0	40	0	3	50
0	0	0	0	0	0	0	2	2
6	14	555	3	314	1424	0	49	2365
0	0	0	0	0	0	0	2	2
0	0	101	0	10	10	0	28	149
0	0	0	0	0	0	0	9	9
0	0	0	0	0	0	0	3	3
0	0	0	0	0	0	0	3	3
0	0	0	0	0	0	0	5	5
0	0	0	0	0	0	0	10	10
0	1	0	0	0	0	0	7	8
0	0	90	0	31	19	0	8	148
0	1	152	1	42	143	0	39	378
0	0	0	0	0	0	0	4	4
0	0	0	0	0	0	0	15	15
0	0	0	0	0	0	0	3	3
0	0	0	0	0	0	0	3	3
0	0	0	0	0	0	0	3	3
0	0	0	0	0	0	0	5	5
0	0	13	0	7	0	0	0	20
0	3	0	0	0	0	0	3	6
0	0	0	0	0	0	0	6	6
0	0	0	0	0	0	0	6	6
0	0	0	0	0	0	0	17	17
0	0	44	0	0	0	0	0	44
0	0	0	0	0	0	0	3	3
0	0	71	0	0	0	0	0	71

0	0	25	0	0	0	0	0	25
0	0	0	0	0	0	0	4	4
0	0	0	0	0	0	0	7	7
0	0	0	0	0	0	0	7	7
0	0	0	0	0	0	0	24	24
0	0	0	0	0	0	0	5	5
0	0	0	0	0	0	0	9	9
0	0	0	0	0	0	0	9	9
0	0	0	0	0	0	0	15	15
0	0	0	0	0	0	0	3	3
0	0	0	0	0	0	0	5	5
0	0	0	0	0	0	0	15	15
1	0	8	0	0	7	0	1	17
0	0	40	0	0	0	0	5	45
0	0	0	0	0	55	0	0	55
0	0	6	0	1	0	0	0	7
0	0	0	0	0	0	0	10	10
0	0	0	0	0	0	0	1	1
0	0	0	0	0	5	0	3	8
0	0	0	0	0	0	0	12	12
0	0	30	0	0	0	0	0	30
0	0	0	0	0	0	0	31	31
0	1	0	0	0	0	0	0	1
0	0	0	0	0	0	0	72	72
0	0	0	0	0	0	0	6	6
0	0	0	0	0	0	0	13	13
0	0	0	0	0	0	0	11	11
0	0	0	0	2	0	0	0	2
0	0	0	0	0	0	0	8	8
0	0	0	0	1	0	0	0	1
0	0	6	0	0	0	0	0	6
0	0	0	0	0	0	0	4	4
0	0	0	0	0	0	0	15	15
0	0	0	0	0	0	0	9	9
0	0	12	0	8	0	0	0	20
2	0	0	0	2	0	0	21	25
0	2	0	0	0	0	0	10	12
0	0	0	0	0	0	0	10	10
0	0	0	0	0	0	0	7	7
1	1	187	0	19	74	0	23	305
0	0	10	0	1	0	0	0	11
0	0	0	0	0	0	0	1	1
0	0	0	0	0	0	0	7	7
0	0	0	0	0	0	0	15	15
0	0	0	0	0	0	0	7	7
0	0	0	0	0	0	0	22	22
0	0	0	0	2	0	0	0	2

0	0	30	0	0	0	0	0	30
0	0	0	0	0	98	0	0	98
0	0	0	0	0	0	0	11	11
0	0	0	1	0	0	0	0	1
1	0	61	0	23	10	0	9	104
0	0	0	0	0	0	0	5	5
0	0	0	0	0	0	0	12	12
0	0	0	0	0	0	0	12	12
0	0	0	0	0	0	0	5	5
0	0	0	0	0	0	0	6	6
0	1	0	0	0	0	0	4	5
0	0	0	0	3	0	0	0	3
0	0	0	0	0	0	0	16	16
0	0	0	0	0	0	0	11	11
0	0	0	0	0	0	0	16	16
0	0	0	0	0	0	0	253	253
7	0	0	27	0	826	0	0	860
0	0	5	0	2	21	0	0	28
0	0	0	0	0	0	0	16	16
0	0	0	0	0	0	0	19	19
0	0	0	0	0	0	0	11	11
0	0	0	0	0	39	0	0	39
0	0	0	0	0	0	0	17	17
0	0	0	0	1	78	0	0	79
0	0	0	0	0	0	0	4	4
0	0	10	0	0	0	0	0	10
0	1	182	1	41	211	0	17	453
0	0	0	0	0	0	0	4	4
0	1	0	0	0	0	0	26	27
0	0	0	0	0	7	0	0	7
0	0	0	0	0	0	0	17	17
0	0	14	0	0	0	0	0	14
0	0	0	0	0	0	0	71	71
0	0	0	0	0	0	0	9	9
0	0	0	0	0	0	0	3	3
0	0	0	0	0	0	0	17	17
0	0	0	0	0	0	0	4	4
0	0	0	0	0	0	0	7	7
0	0	0	0	10	0	0	0	10
0	0	0	0	0	0	0	6	6
0	0	0	0	0	0	0	3	3
0	0	0	0	6	0	0	0	6
0	0	0	0	0	0	0	21	21
0	0	0	0	3	0	0	0	3
0	0	51	0	0	0	0	0	51
0	0	6	0	0	0	0	14	20
0	0	0	0	0	0	0	8	8

0	0	0	0	0	0	0	4	4
0	0	0	0	0	0	0	12	12
0	0	0	0	0	0	0	19	19
0	0	0	0	0	0	0	16	16
0	0	1	0	1	0	0	0	2
0	0	50	0	0	0	0	107	157
0	0	6	0	0	0	0	0	6
0	0	0	0	0	0	0	3	3
0	0	0	0	0	0	0	31	31
0	0	2	0	0	0	0	0	2
0	2	0	0	0	0	0	68	70
2	0	31	1	0	0	0	0	34
0	0	0	0	0	0	0	29	29
0	4	0	0	0	0	0	0	4
0	0	0	0	0	0	0	9	9
0	0	0	0	0	0	0	6	6
0	0	0	0	0	0	0	19	19
0	0	0	0	0	0	0	21	21
0	0	0	0	0	0	0	10	10
0	0	0	0	0	0	0	3	3
127	0	0	26	0	0	81	1	235
0	0	0	0	0	0	0	8	8
0	0	0	0	0	5	0	0	5
0	0	0	0	3	0	0	0	3
0	0	4	0	0	0	0	12	16
0	0	0	0	0	0	0	26	26
0	0	0	0	0	0	0	2	2
0	0	0	0	0	0	0	11	11
0	0	0	0	0	2	0	0	2
0	0	0	0	0	0	0	3	3
0	0	0	0	0	0	0	9	9
0	0	0	0	0	0	0	5	5
0	0	59	0	124	2	0	0	185
0	1	270	1	24	11	0	0	307
0	0	0	0	0	0	0	31	31
0	0	0	0	0	0	0	16	16
0	0	0	0	0	0	0	1	1
1	0	0	0	0	0	0	0	1
0	0	0	0	0	0	0	1	1
0	0	0	0	0	0	0	15	15
0	0	0	0	0	0	0	11	11
0	0	51	0	0	0	0	0	51
0	0	0	0	0	0	0	86	86
0	0	0	0	0	0	0	6	6
0	0	0	0	0	0	0	7	7
0	0	0	0	0	0	0	114	114
0	0	0	0	0	0	0	19	19

0	0	0	0	0	0	0	46	46
0	0	0	0	0	0	0	1	1
0	0	0	0	5	0	0	1	6
0	0	0	0	0	0	0	2	2
0	0	0	0	0	0	0	5	5
0	0	0	0	0	0	0	2	2
0	1	0	0	0	0	0	0	1
0	0	0	0	0	0	0	7	7
0	0	0	0	0	3	0	0	3
0	0	0	0	1	0	0	0	1
0	0	0	0	0	0	0	2	2
0	0	0	0	0	0	0	12	12
0	0	0	0	0	0	0	5	5
0	0	0	0	0	0	0	22	22
0	0	0	0	0	0	0	2	2
0	0	0	0	0	1	0	160	161
0	0	0	0	0	0	0	2	2
0	0	0	2	0	0	0	0	2
0	0	30	0	0	0	0	0	30
0	0	0	0	0	0	0	2	2
0	0	0	0	0	0	0	6	6
0	0	13	0	0	0	0	0	13
0	0	0	0	0	22	0	0	22
0	1	0	0	0	0	0	7	8
0	1	0	0	0	0	0	18	19
0	0	10	0	0	0	0	0	10
0	0	0	0	0	0	0	2	2
0	0	0	0	0	0	0	2	2
0	0	0	0	0	0	0	4	4
0	0	44	0	0	0	0	0	44
0	0	2	0	0	13	0	0	15
0	0	9	0	0	14	0	0	23
0	0	137	0	5	0	0	13	155
0	0	0	0	0	15	0	2	17
0	0	0	0	0	0	0	8	8
0	0	0	0	4	9	0	0	13
0	0	0	0	0	0	0	64	64
0	0	0	0	0	0	0	3	3
0	0	0	0	0	0	0	1	1
0	0	50	0	5	0	0	90	145
0	0	0	0	0	0	0	10	10
0	0	33	0	0	70	0	25	128
0	0	73	0	9	3	0	9	94
1	0	227	0	5	116	0	6	355
0	0	0	0	0	0	0	17	17
0	0	0	0	0	0	0	4	4
0	1	57	0	23	76	0	38	195

0	0	0	0	0	0	0	13	13
0	0	70	0	10	0	0	0	80
0	0	0	0	0	0	0	10	10
0	0	0	0	0	125	0	29	154
0	0	0	0	0	0	0	5	5
0	0	0	0	0	0	0	4	4
0	0	0	0	0	0	0	19	19
0	0	0	0	0	0	0	5	5
0	0	6	0	0	0	0	0	6
0	0	0	0	0	0	0	2	2
1	2	66	0	4	24	0	7	104
0	0	0	0	0	0	0	5	5
0	0	26	0	0	0	0	0	26
0	1	0	0	7	0	0	0	8
0	0	0	0	0	0	0	22	22
0	0	0	0	2	0	0	0	2
0	0	0	0	0	0	0	8	8
0	0	0	0	0	3	0	0	3
0	0	0	0	0	0	0	7	7
0	0	0	0	0	0	0	2	2
0	0	0	0	0	0	0	21	21
0	0	0	0	0	0	0	20	20
0	1	0	0	0	0	0	2	3
0	1	28	0	0	0	0	0	29
0	0	0	0	4	0	0	0	4
0	0	0	0	0	0	0	2	2
0	5	128	0	1160	234	0	591	2118
0	0	0	0	0	0	0	4	4
0	0	0	0	0	0	0	5	5
0	0	0	0	0	0	0	5	5
0	0	0	0	0	0	0	8	8
0	0	0	0	0	0	0	7	7
0	0	12	0	0	0	0	0	12
0	0	10	0	0	0	0	0	10
11	0	0	2	0	0	0	0	13
0	0	0	0	0	0	0	3	3
0	0	0	0	0	0	0	21	21
0	0	5	0	0	0	0	0	5
0	0	0	0	0	0	0	18	18
0	0	1	0	0	0	0	0	1
0	0	62	0	25	85	0	1	173
0	0	9	0	0	0	0	11	20
0	0	0	0	0	51	0	0	51
0	0	0	0	0	39	0	0	39
0	0	16	0	9	53	0	0	78
0	2	0	0	0	0	0	0	2
0	1	0	0	0	0	0	7	8

0	0	0	0	0	0	0	12	12
0	0	12	0	0	0	0	0	12
0	0	0	0	0	0	0	11	11
0	0	32	0	0	0	0	0	32
0	0	0	0	0	0	0	22	22
0	0	0	0	0	0	0	4	4
0	0	0	0	0	0	0	8	8
0	19	810	0	192	148	0	335	1504
0	0	12	0	0	53	0	4	69
0	0	0	0	1	1	0	0	2
0	0	0	0	0	0	0	3	3
0	0	0	0	0	35	0	0	35
0	1	0	0	0	0	0	5	6
0	0	0	0	0	0	0	2	2
0	0	0	0	0	120	2	376	498
0	0	0	0	0	0	0	5	5
0	0	0	0	0	0	0	3	3
0	0	16	0	6	0	0	0	22
0	0	86	0	0	67	0	0	153
0	0	0	0	0	0	0	10	10
0	0	5	0	9	0	0	0	14
0	0	16	0	0	0	0	1	17
0	0	0	0	0	0	1	0	1
0	0	5	0	0	0	0	0	5
0	0	24	0	0	0	0	0	24
0	0	54	0	2	0	0	24	80
0	1	0	0	0	0	0	15	16
0	0	0	0	0	0	0	4	4
0	0	16	0	0	10	0	0	26
0	0	0	0	0	0	0	7	7
0	0	0	0	0	0	0	2	2
0	0	34	0	0	0	0	0	34
0	0	0	0	0	15	0	57	72
0	0	0	0	0	0	0	7	7
0	0	21	0	0	0	0	0	21
0	0	0	0	0	0	0	14	14
0	3	106	0	7	0	0	1	117
1	0	0	0	0	0	0	0	1
0	0	0	0	0	0	0	1048	1048
0	0	0	0	0	0	0	1	1
0	0	0	0	0	0	0	5	5
0	0	3	1	0	12	0	0	16
0	4	71	0	110	1	0	2	188
0	0	27	0	0	0	0	0	27
0	0	0	0	0	0	0	2	2
0	0	0	0	0	0	0	14	14
0	0	0	0	0	0	0	5	5

0	0	0	0	0	0	0	2	2
0	0	0	0	0	0	0	3	3
0	0	0	0	0	0	0	2	2
0	0	8	0	0	0	0	0	8
0	0	0	0	0	7	0	0	7
0	0	28	0	0	0	0	0	28
0	0	0	0	0	0	0	2	2
0	0	0	0	0	0	0	2	2
0	0	0	0	0	0	0	4	4
0	2	36	0	14	1304	0	51	1407
0	0	0	0	0	0	0	7	7
0	0	0	0	0	0	0	21	21
0	0	0	0	1	0	0	6	7
0	0	0	0	0	0	0	18	18
0	0	0	0	0	0	0	11	11
0	0	0	0	0	0	0	2	2
0	0	0	0	0	0	0	10	10
0	0	1	1	5	0	0	0	7
0	0	0	0	0	0	0	15	15
1	0	0	0	2	0	0	14	17
0	1	0	0	0	0	0	7	8
0	0	0	0	0	0	0	6	6
0	0	0	0	0	0	0	1	1
0	0	0	0	0	0	0	7	7
0	1	0	0	0	0	0	4	5
0	0	0	0	0	0	0	6	6
0	0	62	0	0	0	0	33	95
0	0	0	0	0	0	0	2	2
0	0	0	0	0	0	0	11	11
0	0	3	0	0	0	0	0	3
0	0	0	0	0	0	0	2	2
0	0	0	0	0	0	0	3	3
0	0	0	0	9	0	0	21	30
0	0	2	0	3	0	0	0	5
0	0	0	0	0	0	0	17	17
0	0	0	0	0	0	0	2	2
0	0	0	0	0	36	0	0	36
0	1	0	0	0	0	0	6	7
0	2	0	0	0	0	0	8	10
0	0	0	0	0	0	0	16	16
0	0	0	1	0	0	0	0	1
0	0	0	0	0	0	0	17	17
0	0	0	0	0	0	0	12	12
0	0	0	0	0	0	0	15	15
0	0	0	0	0	0	0	3	3
0	0	0	1	0	0	0	0	1
0	0	0	0	0	0	0	29	29

0	1	0	0	0	0	0	3	4
0	7	2906	0	426	6	0	55	3400
0	0	0	0	0	0	0	2	2
0	0	3	0	0	0	0	0	3
0	0	0	0	0	0	0	6	6
0	7	755	4	199	355	0	0	1320
0	0	0	0	0	0	0	472	472
0	0	0	0	0	49	0	1	50
0	0	6	0	0	0	0	0	6
0	2	64	0	1	4	0	8	79
0	0	0	0	3	0	0	17	20
0	0	0	0	0	0	0	5	5
0	1	0	0	0	0	0	3	4
0	0	0	0	0	0	0	1	1
0	1	0	0	0	0	0	7	8
0	0	26	0	0	0	0	0	26
0	1	0	0	0	0	0	0	1
0	0	0	0	0	0	0	4	4
0	0	0	0	0	0	0	15	15
0	0	0	0	0	0	0	5	5
0	0	0	0	0	0	0	12	12
0	0	0	0	0	0	0	4	4
0	0	0	0	0	0	0	5	5
0	0	0	0	9	22	0	0	31
0	0	2	0	2	4	0	0	8
0	0	0	0	0	0	0	2	2
0	0	0	0	0	0	0	4	4
0	0	45	0	0	0	0	0	45
0	0	26	0	0	0	0	0	26
0	0	0	0	4	40	0	24	68
0	0	0	0	0	0	0	24	24
0	0	56	0	13	24	0	14	107
0	0	0	0	0	79	0	0	79
0	0	5	0	1	0	0	0	6
0	0	0	0	0	0	0	8	8
0	0	0	0	0	0	0	3	3
17	141	3209	26	956	6434	1	114	10898
0	0	27	0	0	44	0	5	76
0	0	0	0	0	0	0	385	385
0	0	28	0	20	0	0	7	55
0	1	0	0	0	0	0	19	20
0	0	18	0	0	4	0	0	22
0	1	0	0	0	0	0	12	13
0	0	147	0	78	81	0	11166	11472
0	0	129	0	6	0	0	0	135
0	0	0	0	0	0	0	5	5
0	0	2	0	0	0	0	0	2

0	0	2	0	0	0	0	0	2
1	32	1877	3	147	1938	0	78	4076
0	0	0	0	5	0	0	0	5
0	0	0	0	0	5	0	0	5
1	0	0	0	0	0	0	13	14
0	0	25	0	0	0	0	0	25
0	0	0	0	0	8	0	0	8
1	1	7	0	0	0	0	8	17
0	0	0	0	0	0	0	3	3
0	1	0	0	0	0	0	142	143
0	1	0	0	0	0	0	65	66
0	516	67	0	0	0	0	1	584
0	0	0	0	0	0	0	2	2
0	0	7	0	0	0	0	0	7
14	5	433	21	512	4153	0	17	5155
0	0	0	0	0	0	0	3	3
0	0	22	0	0	0	0	20	42
0	0	0	0	0	0	0	4	4
0	0	0	0	0	0	0	21	21
0	0	0	0	0	0	0	4	4
0	0	12	0	0	31	0	2	45
0	3	116	0	5	60	0	20	204
0	0	0	0	0	0	0	22	22
0	0	0	0	0	0	0	10	10
0	0	156	0	199	0	0	0	355
0	0	0	0	0	0	0	11	11
0	0	0	0	0	0	0	12	12
0	3	0	0	0	0	0	56	59
0	0	0	0	0	0	0	14	14
0	0	1	0	1	0	0	0	2
0	0	0	0	0	0	0	2	2
0	0	0	0	0	0	0	4	4
0	0	22	0	0	0	0	56	78
0	0	0	0	0	0	0	14	14
0	0	0	0	0	0	0	3	3
0	0	0	0	0	0	0	6	6
0	2	6	0	0	0	0	0	8
0	3107	0	0	0	0	11	0	3118
0	0	3	0	4	11	0	0	18
0	0	19	0	0	0	0	0	19
0	0	0	0	0	0	0	20	20
0	0	0	0	0	0	0	4	4
0	0	0	0	0	38	0	0	38
0	0	0	0	0	0	0	13	13
0	0	0	0	0	0	0	4	4
0	0	0	0	0	0	0	27	27
0	0	0	0	0	0	0	11	11

0	0	0	0	0	0	0	5	5
0	0	0	0	0	0	0	4	4
0	0	0	0	0	0	0	2	2
0	0	0	0	0	0	0	4	4
0	0	0	0	0	0	0	5	5
0	0	21	0	0	0	0	0	21
0	0	0	0	0	0	0	11	11
0	0	88	0	2	58	0	75	223
0	0	0	0	0	0	0	2	2
0	0	0	0	0	26	0	0	26
0	0	0	0	0	0	0	13	13
0	0	0	0	0	0	0	37	37
0	0	0	0	0	0	0	8	8
0	0	0	0	0	0	0	6	6
0	0	0	0	0	0	0	4	4
0	2	106	0	0	271	0	7	386
0	0	0	0	0	0	0	2	2
0	0	5	0	0	0	0	0	5
0	0	0	0	0	0	0	24	24
0	0	0	0	0	0	0	11	11
0	0	0	0	4	0	0	0	4
1	0	0	0	0	0	0	1	2
0	0	8	0	5	0	0	0	13
0	1	0	0	0	0	0	15	16
0	0	0	0	0	0	0	11	11
0	0	0	0	0	0	0	9	9
0	2	36	0	12	24	0	8	82
0	0	0	0	1	0	0	4	5
0	0	0	0	8	90	0	0	98
0	0	0	0	0	0	0	5	5
0	0	0	0	0	9	0	0	9
0	0	0	0	0	0	0	6	6
0	0	20	0	2	0	0	0	22
0	0	0	0	0	0	0	4	4
0	0	0	0	0	0	1	27	28
0	0	25	0	2	0	0	0	27
0	0	0	0	5	0	0	0	5
0	0	0	0	0	0	0	2	2
0	0	13	0	0	0	0	0	13
0	0	27	0	13	0	0	6	46
0	0	63	0	0	0	0	0	63
0	0	45	0	0	0	0	1932	1977
0	0	0	0	0	0	0	8	8
0	0	0	0	0	0	0	22	22
0	0	31	0	0	0	0	0	31
1	0	0	0	0	0	0	5	6
0	0	0	0	0	0	0	66	66

0	0	7	0	0	0	0	0	7
0	0	17	0	0	0	0	0	17
0	0	8	0	0	0	0	0	8
0	0	0	0	4	0	0	0	4
0	0	0	0	0	0	0	2	2
0	0	17	0	0	0	0	0	17
0	0	0	0	0	0	0	2	2
0	0	0	0	0	0	0	110	110
0	0	0	0	0	0	0	14	14
0	0	28	0	8	0	0	0	36
0	0	0	0	2	0	0	0	2
0	0	0	0	0	0	0	4	4
0	0	0	0	0	0	0	4	4
0	0	0	0	0	0	0	11	11
0	0	0	0	0	0	0	11	11
0	0	0	0	0	0	0	6	6
2	0	13	0	5	0	0	0	20
0	0	0	0	0	0	0	10	10
0	0	0	0	0	73	0	0	73
0	0	0	0	0	0	0	2	2
0	0	0	0	0	0	0	24	24
0	0	0	0	0	0	0	5	5
0	0	0	0	0	0	0	28	28
0	0	0	0	0	0	0	8	8
0	0	0	0	0	0	0	3	3
0	0	1	0	0	0	0	192	193
0	0	6	0	0	0	0	0	6
0	0	0	0	0	0	0	2	2
0	0	0	0	0	0	0	11	11
0	1	0	0	0	0	0	6	7
0	0	0	0	0	0	6	59	65
0	2	221	1	101	293	0	36	654
0	0	0	0	0	0	0	9	9
0	0	0	0	0	0	0	2	2
0	0	48	0	6	0	0	0	54
0	0	15	0	0	0	0	3	18
0	0	0	0	0	0	0	13	13
0	0	0	0	0	0	0	16	16
0	0	0	0	0	0	0	4	4
0	1	0	0	0	0	0	0	1
0	0	0	0	0	0	0	6	6
0	0	0	0	0	0	0	15	15
0	0	0	0	0	0	0	7	7
0	0	0	0	1	0	0	0	1
0	0	0	0	0	0	0	3	3
0	0	6	0	0	0	0	7	13
0	0	0	0	0	0	0	5	5

0	0	0	0	0	0	0	14	14
0	0	0	0	0	0	0	15	15
0	0	0	0	0	0	0	35	35
0	0	0	0	0	22	0	0	22
0	0	0	0	0	0	1	90	91
0	0	0	0	0	0	0	4	4
0	0	0	0	0	0	0	1	1
0	0	0	0	0	0	0	1	1
0	0	0	0	0	0	0	5	5
0	0	0	0	0	0	0	1	1
0	0	32	0	0	0	0	0	32
0	0	0	0	0	0	0	9	9
0	0	0	0	0	0	0	10	10
0	0	0	0	0	0	0	21	21
0	0	0	0	0	0	0	4	4
0	0	0	0	0	0	0	12	12
0	0	0	0	0	0	0	11	11
0	0	0	0	0	0	0	8	8
0	0	20	0	0	0	0	0	20
0	0	51	0	43	9	0	4	107
0	0	0	0	0	0	0	10	10
0	0	0	0	0	0	0	4	4
0	0	0	0	0	0	0	1	1
1	3	595	1	174	0	0	0	774
0	0	0	0	0	6	0	0	6
0	0	0	0	0	0	0	7	7
0	0	0	0	5	0	0	0	5
0	1	0	0	0	0	0	0	1
0	0	0	0	0	0	0	30	30
0	1	0	0	0	0	0	0	1
0	1	51	0	11	41	0	1	105
0	4	568	0	163	245	0	142	1122
0	0	0	0	0	0	0	12	12
0	0	0	0	1	21	0	0	22
0	0	0	0	0	0	0	3	3
0	0	0	0	0	0	0	3	3
0	0	0	0	0	0	0	11	11
0	0	15	0	0	0	0	0	15
0	0	0	0	0	0	0	14	14
0	0	0	0	0	0	0	22	22
0	0	21	0	0	0	0	0	21
0	0	0	0	0	0	0	3	3
0	0	0	0	0	0	0	107	107
0	0	0	0	0	0	0	4	4
0	0	87	0	10	21	0	0	118
0	0	0	0	0	0	0	8	8
0	0	33	0	0	0	0	0	33

0	0	5	0	1	0	0	0	6
0	0	0	0	0	0	0	4	4
0	0	0	0	0	0	0	21	21
0	0	0	0	3	0	0	0	3
0	0	0	0	0	0	0	6	6
0	0	53	0	36	524	0	4	617
0	0	24	0	14	0	0	0	38
0	1	0	0	0	0	0	21	22
0	0	0	0	0	62	0	0	62
0	0	0	0	0	0	0	5	5
0	0	0	0	0	0	0	9	9
0	0	0	0	0	0	0	2	2
0	0	0	0	1	0	0	6	7
0	0	0	0	0	0	0	136	136
0	0	0	0	0	0	0	9	9
0	0	0	0	0	0	0	18	18
1	0	0	0	1	0	0	5	7
0	0	0	0	0	0	0	17	17
0	0	0	0	0	0	0	2	2
0	0	0	0	0	0	0	15	15
0	0	0	0	0	0	0	8	8
0	0	0	0	0	0	0	4	4
0	0	0	0	4	0	0	0	4
0	0	0	0	0	0	0	27	27
0	0	1	0	2	0	0	1	4
0	0	0	0	0	0	0	1	1
0	0	0	0	0	50	0	9	59
0	1	0	0	0	0	0	426	427
0	0	0	0	0	0	0	6	6
0	0	13	0	0	0	0	0	13
0	0	0	0	0	0	0	13	13
0	0	0	0	0	0	0	9	9
1	0	2	0	0	0	0	0	3
0	0	0	0	0	0	0	6	6
0	0	0	0	0	0	0	17	17
0	0	9	0	0	0	0	0	9
0	0	0	0	0	0	0	7	7
0	0	0	0	0	0	0	2	2
0	0	0	0	0	0	0	24	24
0	0	0	0	0	0	0	8	8
0	3	101	0	7	30	0	25	166
0	0	0	0	0	0	0	1	1
0	0	0	0	0	0	0	19	19
0	0	0	0	0	0	0	9	9
0	0	0	0	0	0	0	7	7
0	0	10	0	27	0	0	0	37
0	0	0	0	0	0	0	6	6

0	0	0	0	5	0	0	0	5
0	0	0	0	0	0	0	3	3
0	0	0	0	0	0	0	16	16
0	0	0	0	0	0	0	21	21
0	0	0	0	0	0	0	3	3
0	0	20	0	2	79	0	0	101
0	0	0	0	0	0	0	12	12
0	0	0	0	0	0	0	9	9
0	0	0	0	1	0	0	14	15
0	1	34	0	0	0	0	0	35
0	1	0	0	0	0	0	0	1
0	0	0	0	0	0	0	192	192
0	0	0	0	0	5	0	0	5
0	1	0	0	0	0	0	1	2
0	0	0	0	0	0	0	35	35
0	0	0	0	4	1	0	0	5
0	0	0	0	0	0	0	1	1
0	0	0	0	1	140	0	0	141
0	0	0	0	0	0	0	13	13
0	0	0	0	0	0	0	5	5
0	0	15	0	3	13	0	0	31
0	0	0	0	0	0	0	4	4
0	0	0	0	0	0	0	7	7
0	0	20	0	0	0	0	5	25
0	0	0	0	2	0	0	0	2
0	0	0	0	0	0	0	12	12
0	0	0	0	3	15	0	0	18
0	2	0	0	0	0	0	255	257
0	0	1	0	7	0	0	0	8
0	0	0	0	0	0	0	14	14
0	0	124	1	26	33	0	18	202
0	0	0	0	0	0	0	7	7
0	0	13	0	4	0	0	0	17
0	1	31	0	0	49	0	101	182
0	0	0	0	0	0	0	27	27
0	0	0	0	0	0	0	2	2
0	2	0	0	0	0	0	6	8
0	0	25	0	0	7	0	11	43
0	2	0	0	0	0	0	9	11
0	0	15	0	1	0	0	0	16
0	0	0	0	0	22	0	0	22
0	0	0	0	0	0	0	201	201
0	0	0	0	0	33	0	438	471
0	0	0	0	0	0	0	6	6
0	0	6	0	0	0	0	0	6
0	0	9	0	0	0	0	0	9
0	0	0	0	0	0	0	22	22

0	0	61	0	23	0	0	0	84
0	0	121	0	25	75	0	37	258
0	0	0	0	0	0	0	6	6
0	0	0	0	0	48	0	0	48
0	0	0	0	0	0	0	5	5
0	0	0	0	0	0	0	13	13
4	10	1666	13	286	1444	0	94	3517
0	0	1	0	0	0	0	0	1
0	0	0	0	0	0	0	3	3
0	0	7	0	6	27	0	11	51
0	0	0	0	0	0	0	7	7
0	2	19	0	2	231	0	4	258
0	0	0	0	0	0	0	9	9
2	5	87	0	50	414	0	72	630
1	10	61	0	22	0	0	11	105
0	0	0	0	0	0	0	18	18
0	2	52	0	0	0	0	69	123
0	0	0	0	0	0	0	2	2
0	1	9	0	0	26	0	211	247
0	0	13	0	6	0	0	5	24
0	0	0	0	0	0	0	6	6
0	0	18	0	0	0	0	0	18
0	0	0	0	0	0	0	5	5
0	1	0	0	0	0	0	24	25
0	0	0	0	0	0	0	6	6
0	0	0	0	0	0	0	4	4
0	0	0	0	0	0	0	26	26
0	0	0	0	0	0	0	7	7
0	0	0	0	0	0	0	8	8
0	1	0	0	0	0	0	12	13
0	0	0	0	0	0	0	5	5
0	0	0	0	0	0	0	2	2
1	0	0	0	0	0	0	10	11
0	0	0	0	0	0	0	2	2
0	1	32	0	7	0	0	0	40
0	0	0	0	0	0	0	22	22
0	0	0	0	0	0	0	25	25
0	0	0	0	0	0	0	2	2
0	0	4	0	0	0	0	0	4
0	0	31	0	0	0	0	0	31
0	0	0	0	0	0	0	3	3
0	0	0	0	0	38	0	6	44
0	0	0	0	0	0	0	3	3
0	0	0	0	0	5	0	0	5
0	0	0	0	0	0	0	3	3
0	0	0	0	0	0	0	2	2
0	0	0	0	0	0	0	9	9

0	0	121	0	11	159	0	0	291
0	0	0	0	0	1	0	8	9
1	3	226	0	37	31	0	20	318
0	0	1	0	0	68	0	6	75
0	0	0	0	0	0	0	21	21
0	0	0	0	0	0	0	13	13
0	0	0	0	0	0	0	2	2
0	0	0	0	0	57	0	0	57
3	0	0	0	0	0	0	12	15
0	0	0	0	0	0	0	2	2
0	0	0	0	0	0	0	3	3
0	1	0	0	0	0	1	133	135
0	0	0	0	0	0	0	4	4
0	0	0	0	0	0	0	3	3
0	0	0	0	0	0	0	4	4
0	0	0	0	0	16	0	0	16
0	0	0	0	0	0	0	19	19
0	0	92	0	0	2	0	0	94
0	0	0	0	0	0	0	6	6
0	0	22	0	3	0	0	0	25
0	0	32	0	5	0	0	0	37
0	0	0	0	0	0	0	14	14
0	0	0	0	3	0	0	175	178
0	0	0	0	0	0	0	9	9
0	0	0	0	0	0	0	3	3
0	0	0	0	2	0	0	0	2
0	0	0	0	0	0	0	8	8
0	0	3	0	0	0	0	0	3
0	2	0	0	0	0	0	29	31
0	0	0	0	0	0	1	363	364
0	0	0	0	4	0	0	0	4
0	1	0	0	0	0	0	4	5
0	1	0	0	0	0	0	9	10
0	0	0	0	0	0	0	8	8
0	0	0	0	0	0	0	56	56
0	0	0	0	0	0	0	8	8
0	2	28	0	0	129	0	12	171
0	0	0	0	0	0	0	8	8
0	2	193	1	16	251	0	117	580
0	0	0	0	0	0	0	22	22
0	0	0	0	4	0	0	0	4
0	0	0	0	0	0	0	7	7
0	0	3	0	0	4	0	3	10
0	0	0	0	0	0	0	10	10
0	0	0	0	0	0	0	1	1
0	0	0	0	0	0	0	13	13
0	0	0	0	3	24	0	0	27

0	0	0	0	0	0	0	5	5
0	0	0	0	0	0	0	12	12
0	2	80	0	15	0	0	2	99
0	0	21	0	0	0	0	0	21
0	0	0	0	0	0	0	9	9
0	3	0	0	0	0	0	2	5
0	0	0	0	2	0	0	63	65
0	1	0	0	0	0	0	10	11
0	0	87	0	4	0	0	7	98
0	1	1	0	0	0	0	3	5
0	0	0	0	0	0	0	3	3
0	0	0	0	0	0	0	2	2
0	0	13	0	0	0	0	0	13
0	0	0	0	0	0	0	8	8
0	0	0	0	0	0	0	3	3
0	0	0	0	0	0	0	10	10
0	0	0	0	0	0	0	4	4
0	0	0	0	0	0	0	3	3
0	0	0	0	0	0	0	26	26
0	0	0	0	0	0	0	16	16
0	0	0	0	0	0	0	5	5
0	0	0	0	0	0	0	50	50
0	0	0	0	12	0	0	12	24
0	0	19	0	6	0	0	0	25
0	0	0	0	0	0	0	10	10
0	0	0	0	1	0	0	17	18
0	0	0	0	0	0	0	19	19
1	15	132	0	291	65	0	0	504
0	0	0	0	0	0	0	4	4
0	0	1	0	0	21	0	26	48
0	0	2	0	0	0	0	0	2
0	0	0	0	0	0	0	16	16
0	0	0	0	0	0	0	3	3
0	0	0	0	0	0	0	19	19
0	0	1	0	10	47	0	0	58
0	0	0	0	0	0	0	5	5
0	0	53	0	0	0	0	0	53
0	1	0	0	0	0	0	6	7
0	1	0	0	12	0	0	0	13
0	0	0	0	0	0	0	12	12
0	0	0	0	0	0	0	7	7
1	13	1178	0	334	350	0	124	2000
0	0	0	0	1	0	0	7	8
0	0	0	0	0	0	0	5	5
0	0	0	0	0	0	1	34	35
0	0	0	0	0	0	0	3	3
0	1	0	0	0	0	0	8	9

1	2	0	0	0	0	0	0	3
0	1	80	0	20	288	0	24	413
0	0	0	0	0	0	0	10	10
0	0	0	0	0	48	0	0	48
0	1	0	0	0	0	0	17	18
0	0	14	0	0	0	1	0	15
0	0	4	0	2	0	0	0	6
0	0	0	0	0	0	0	18	18
0	0	0	0	0	0	0	3	3
0	0	3	0	0	0	0	0	3
0	0	0	0	0	0	0	4	4
0	1	0	0	0	38	0	2	41
0	0	0	0	0	0	0	12	12
0	0	0	0	0	0	0	5	5
0	0	0	0	0	0	0	27	27
0	0	0	0	0	0	0	6	6
0	0	0	0	1	40	0	20	61
0	0	0	0	0	1	0	0	1
0	0	0	0	0	0	0	18	18
0	0	0	0	0	0	0	16	16
0	0	2	0	0	0	0	0	2
0	0	0	0	0	0	0	6	6
1	1	89	0	54	0	0	0	145
0	5	439	1	118	15	0	8	586
0	0	0	0	0	0	0	14	14
0	0	0	0	0	0	0	98	98
0	0	70	0	12	0	0	0	82
0	0	0	0	0	0	0	19	19
0	0	0	0	1	0	0	26	27
0	0	0	0	0	0	0	77	77
3	77	1128	8	230	1792	0	50	3288
0	0	0	0	0	0	0	4	4
0	0	19	0	4	0	0	0	23
0	0	0	0	17	20	0	3	40
0	0	0	0	0	0	0	24	24
0	1	0	0	0	0	0	17	18
0	0	0	0	0	0	0	24	24
0	0	0	0	0	0	0	3	3
0	0	0	0	0	0	0	2	2
0	1	41	0	1	0	0	30	73
0	0	0	0	0	20	0	0	20
0	0	3	0	6	0	0	0	9
0	0	0	0	0	0	0	13	13
0	0	0	0	0	0	0	11	11
0	0	0	0	0	0	0	2	2
0	0	7	0	0	0	0	0	7
0	0	0	0	0	0	0	2	2

0	0	0	0	0	0	0	10	10
0	0	0	0	0	0	0	3	3
1	0	0	0	3	0	0	24	28
0	0	0	0	0	0	0	9	9
0	0	0	0	0	0	0	8	8
0	0	0	0	0	0	0	3	3
0	2	0	0	0	0	0	7	9
0	0	0	0	0	0	0	27	27
0	1	2	0	17	22	0	0	42
0	0	0	0	1	0	0	0	1
0	0	0	0	0	0	0	4	4
0	0	0	0	0	0	0	6	6
0	0	0	0	0	0	0	8	8
0	0	0	0	0	43	0	43	86
0	2	24	0	2	0	0	0	28
0	0	0	0	0	0	0	9	9
0	0	26	0	0	0	0	0	26
0	0	0	0	0	0	0	8	8
0	0	0	0	0	0	0	1	1
0	0	0	0	0	0	0	15	15
0	0	0	0	0	2	0	0	2
0	0	0	0	0	0	0	6	6
0	0	0	0	1	0	0	5	6
0	0	0	0	5	0	0	0	5
0	0	0	0	0	0	0	11	11
0	0	25	0	0	0	0	0	25
0	0	2	0	3	0	0	0	5
0	0	0	0	0	0	0	9	9
0	0	0	0	0	0	0	42	42
0	0	0	0	3	0	0	0	3
0	0	0	0	0	0	0	104	104
0	0	0	0	0	0	0	150	150
0	0	0	0	0	0	0	2	2
0	0	0	0	0	0	0	7	7
0	0	0	0	0	0	0	2	2
0	0	0	0	0	0	0	16	16
0	0	0	0	0	0	0	6	6
0	0	0	0	0	0	0	9	9
0	0	0	2	0	0	0	1	3
0	0	0	0	0	107	0	26	133
0	0	0	0	0	0	0	17	17
0	0	0	0	0	0	0	6	6
0	0	0	0	0	0	0	3	3
0	0	0	0	0	0	0	5	5
0	0	0	0	0	0	0	3	3
0	0	0	0	0	0	0	15	15
0	0	0	0	0	0	0	9	9

0	0	0	0	0	0	0	6	6
0	0	0	0	0	0	0	4	4
0	0	0	0	0	0	0	22	22
0	0	0	0	0	0	0	5	5
0	0	0	0	0	0	0	2	2
0	0	33	0	0	0	0	0	33
0	0	0	0	0	0	0	2	2
0	0	0	0	0	0	0	4	4
0	1	0	0	0	0	0	15	16
0	0	0	0	0	0	0	24	24
0	0	0	0	0	0	0	12	12
0	0	0	0	0	0	0	20	20
0	1	0	0	0	0	0	0	1
0	0	48	0	5	3	0	0	56
0	0	0	0	0	0	0	3	3
1	0	0	0	0	0	0	7	8
0	0	0	0	0	0	0	4	4
0	0	0	0	0	0	0	17	17
0	0	14	0	14	0	0	0	28
0	0	0	0	0	0	0	8	8
0	0	56	0	0	0	0	39	95
0	0	0	0	0	0	0	17	17
0	0	333	0	48	24	0	34	439
0	0	0	0	2	1	0	0	3
0	0	0	0	0	0	0	3	3
0	0	0	0	0	0	0	8	8
0	0	0	0	0	0	0	13	13
0	3	164	0	139	67	0	0	373
0	2	0	0	0	0	1	4	7
0	0	11	0	0	0	0	0	11
0	0	3	0	0	0	0	34	37
0	0	0	0	0	0	0	3	3
0	0	0	0	0	26	0	0	26
0	1	0	0	0	0	0	15	16
0	0	31	0	9	2	0	14	56
0	1	0	0	0	0	0	7	8
0	0	5	0	0	49	0	0	54
0	0	0	0	0	0	0	5	5
0	0	0	0	0	0	0	5	5
0	0	11	0	0	0	0	0	11
0	0	0	0	0	0	0	2	2
0	0	0	0	0	0	0	6	6
0	0	0	0	0	61	0	48	109
0	0	0	0	0	0	0	4	4
0	1	0	0	0	0	0	0	1
0	0	0	0	0	135	0	0	135
0	0	0	0	0	0	0	201	201

0	0	0	0	0	0	0	17	17
0	0	0	0	0	0	0	17	17
0	0	0	0	0	0	0	118	118
0	0	0	0	0	0	0	2	2
0	0	0	0	0	0	0	16	16
0	0	0	0	0	0	0	11	11
0	0	0	0	0	0	0	18	18
0	0	0	0	0	0	0	1	1
0	0	0	0	0	0	0	11	11
0	0	0	0	0	0	0	15	15
0	0	26	0	10	5	0	9	50
0	0	0	0	0	0	0	11	11
0	0	0	0	0	0	0	3	3
0	0	0	0	0	0	0	5	5
0	0	1	0	0	0	0	0	1
0	0	0	0	0	0	0	7	7
0	0	0	0	0	0	0	9	9
0	0	3	0	0	0	0	0	3
0	0	0	0	0	0	0	10	10
0	0	0	0	0	0	0	5	5
0	0	0	0	0	16	0	0	16
0	0	0	0	0	0	0	2	2
0	0	3	0	0	0	0	2	5
1	1	38	0	36	0	0	0	76
0	0	0	0	0	15	0	1	16
0	0	0	0	0	0	0	5	5
0	0	0	0	0	0	0	10	10
0	0	0	0	0	0	0	1	1
0	0	0	0	2	0	0	0	2
0	1	0	1	0	0	0	3	5
0	0	0	0	0	0	0	15	15
0	0	0	0	0	0	0	9	9
0	0	33	0	0	0	0	0	33
0	0	0	0	0	23	0	0	23
0	1	0	0	0	0	0	11	12
0	1	0	0	0	0	0	2	3
0	0	0	0	0	0	0	3	3
0	0	0	0	0	0	0	5	5
0	0	0	0	0	0	0	3	3
0	0	0	0	0	0	0	9	9
0	0	0	0	0	0	0	42	42
0	0	0	0	0	0	0	19	19
0	0	0	0	0	0	0	4	4
0	0	17	0	0	0	0	0	17
0	0	0	0	0	0	0	3	3
0	2	0	0	0	0	0	6	8
0	0	0	0	0	0	0	7	7

0	0	17	0	0	0	0	0	17
0	0	23	0	0	0	0	0	23
0	0	0	0	0	0	0	7	7
0	0	0	0	0	0	0	6	6
0	0	0	0	0	0	0	20	20
0	0	11	0	1	0	0	0	12
0	3	192	0	99	0	0	0	294
0	0	0	0	0	0	0	5	5
0	1	0	0	0	0	0	2	3
0	0	0	0	0	0	0	14	14
0	0	0	0	0	0	0	6	6
0	0	15	0	0	0	0	0	15
0	1	29	0	0	0	0	5	35
0	0	0	0	0	0	0	3	3
0	0	0	0	0	0	0	13	13
0	0	0	0	0	0	0	7	7
0	0	41	0	2	15	0	0	58
0	0	0	0	0	0	0	6	6
0	0	13	0	0	0	1	34	48
0	0	0	0	0	48	0	0	48
0	0	5	0	0	0	0	0	5
0	2	236	0	7	223	0	387	855
0	0	19	0	5	37	0	6	67
0	0	0	0	0	0	0	10	10
0	0	0	0	0	0	0	17	17
0	0	0	0	0	0	0	1	1
0	3	305	0	85	80	0	66	539
0	0	0	0	0	0	0	2	2
0	0	0	0	0	0	0	18	18
0	0	0	0	2	0	0	0	2
0	0	0	0	0	0	0	5	5
0	0	4	1	4	2	0	2	13
0	0	0	0	0	0	0	4	4
0	1	0	0	0	0	0	3	4
0	0	0	0	0	0	0	3	3
0	0	0	0	0	0	0	6	6
0	0	0	0	0	0	0	6	6
0	0	0	0	0	100	0	1	101
0	0	0	0	0	0	0	7	7
0	0	0	0	0	12	0	0	12
0	0	0	0	0	0	0	16	16
0	1	11	0	0	27	0	0	39
0	0	0	0	0	0	0	19	19
0	0	0	0	0	0	0	7	7
0	0	0	0	0	0	0	3	3
0	0	44	0	0	0	0	0	44
0	0	0	0	0	0	0	10	10

0	0	31	0	0	0	0	16	47
0	0	27	0	18	0	0	22	67
0	3	265	1	84	38	0	0	391
0	0	15	0	0	0	0	0	15
0	0	0	0	0	0	0	5	5
0	0	0	0	7	46	0	16	69
0	0	0	0	0	14	0	0	14
0	0	0	0	0	0	0	10	10
0	0	0	0	0	0	10	6259	6269
1	0	1	0	0	22	0	7	31
0	0	0	0	0	0	0	11	11
0	0	0	0	1	0	0	20	21
0	0	0	0	0	0	0	8	8
0	0	0	0	0	0	0	913	913
0	0	0	0	0	0	0	21	21
0	0	0	0	0	0	0	6	6
0	0	0	0	0	0	0	2	2
0	1	0	0	0	0	0	1043	1044
0	0	0	0	0	0	0	12	12
1	4	175	0	87	149	0	62	478
0	0	0	0	0	0	0	5	5
0	1	0	0	0	70	0	2	73
0	2	0	0	0	0	0	3	5
0	0	56	0	0	0	0	0	56
0	1	0	0	0	0	0	2	3
0	0	61	0	0	0	0	0	61
0	2	0	0	6	22	0	0	30
0	0	0	0	0	0	0	12	12
0	0	0	0	0	0	0	7	7
0	0	0	0	0	0	0	25	25
0	0	8	0	4	32	0	0	44
0	0	4	0	1	62	0	0	67
0	0	0	0	0	0	0	8	8
0	0	21	0	2	0	0	0	23
0	0	49	0	2	0	0	0	51
0	4	405	0	398	0	0	0	807
0	0	0	0	0	0	0	129	129
0	0	0	1	0	0	0	0	1
0	0	41	0	5	0	0	0	46
0	0	0	0	0	0	0	4	4
0	0	31	0	0	0	0	1	32
0	0	213	0	74	219	0	0	506
0	0	0	0	0	0	0	6	6
0	0	0	0	0	0	0	5	5
0	0	154	1	49	25	0	43	272
0	0	0	0	0	0	0	5	5
0	0	0	0	0	0	0	3	3

0	0	0	0	0	0	0	6	6
0	0	13	0	0	0	0	31	44
0	0	57	0	0	140	0	0	197
0	0	15	0	0	39	0	0	54
0	0	57	0	0	0	0	0	57
0	0	0	0	0	0	0	3	3
0	0	0	0	0	0	0	21	21
0	0	0	0	0	0	0	5	5
0	0	11	0	0	0	0	0	11
0	0	18	0	0	0	0	0	18
0	1	0	0	0	0	0	7	8
0	0	0	0	0	0	0	2	2
0	1	790	0	125	10	0	0	926
0	0	0	0	0	0	0	4	4
0	0	0	0	0	0	0	4	4
1	2	295	0	145	456	0	9	908
0	0	0	0	3	0	0	0	3
0	0	2	0	0	0	0	0	2
0	0	0	0	0	0	0	4	4
0	0	0	0	0	0	0	4	4
0	0	63	0	4	1	0	16	84
0	0	0	0	0	0	0	3	3
0	1	231	0	0	0	0	11	243
0	0	0	0	0	0	0	22	22
0	0	0	0	0	0	0	13	13
0	0	0	0	0	5	0	0	5
0	0	0	0	0	58	0	34	92
0	0	0	0	0	0	0	11	11
0	0	0	0	0	0	0	6	6
0	1	15	0	0	0	0	0	16
0	0	0	0	0	0	0	5	5
0	0	11	0	2	0	0	34	47
0	0	0	0	0	0	0	110	110
0	152	0	0	0	0	0	0	152
0	0	0	0	0	0	0	98	98
0	0	0	0	0	0	0	6	6
0	0	0	0	0	0	0	2	2
0	0	37	0	0	0	0	0	37
0	0	5	0	0	0	0	0	5
0	0	2	0	0	0	0	15	17
0	1	0	0	11	277	0	7	296
0	0	0	0	0	0	0	22	22
0	1	0	0	0	0	0	11	12
0	0	0	0	0	0	0	8	8
0	0	0	0	0	0	0	13	13
0	0	0	0	0	0	0	2	2
0	0	12	0	0	66	0	0	78

0	0	9	0	0	0	0	22	31
0	0	0	0	0	0	0	1	1
0	0	0	0	0	0	0	12	12
0	0	0	0	4	0	0	0	4
0	0	0	0	0	28	0	0	28
0	0	0	0	0	0	0	25	25
1	0	267	0	0	3362	0	34	3664
0	0	0	0	0	9	0	0	9
0	0	2	0	0	0	0	0	2
0	0	0	0	0	0	0	5	5
0	0	102	0	6	12	0	2	122
0	0	21	0	0	24	0	2	47
0	0	0	0	0	0	0	4	4
0	0	0	0	0	0	0	8	8
0	0	2	0	0	1	0	0	3
0	1	129	0	57	0	0	0	187
3	3	525	3	130	96	0	47	807
0	0	0	0	5	0	0	0	5
0	0	0	0	0	0	0	2	2
0	0	0	0	0	10	0	0	10
0	0	0	0	0	0	0	12	12
0	1	0	0	0	0	0	11	12
0	0	0	0	0	0	0	10	10
0	0	2	0	0	0	0	0	2
0	0	0	0	0	3	0	15	18
0	2	0	0	0	0	0	0	2
0	0	0	0	0	0	0	9	9
0	0	0	0	0	53	0	0	53
0	1	0	0	0	0	0	3	4
0	1	29	0	33	6	0	0	69
0	0	0	0	0	0	0	8	8
0	0	0	0	0	0	0	13	13
0	0	44	0	0	0	0	0	44
0	0	0	0	0	0	0	12	12
0	0	0	0	0	0	0	9	9
0	0	0	0	0	0	0	9	9
0	0	0	0	0	118	0	0	118
0	1	5	0	37	0	0	0	43
2	0	0	0	0	0	0	12	14
0	0	0	0	0	0	0	1	1
0	0	93	0	1	90	0	35	219
0	0	0	0	0	0	0	8	8
0	0	0	0	0	0	0	6	6
0	0	4	0	0	0	0	0	4
0	0	0	0	0	0	0	26	26
0	0	0	0	0	0	0	13	13
0	0	0	0	0	0	0	4	4

0	0	0	0	0	0	0	9	9
0	0	190	0	5	32	0	2	229
0	0	0	0	0	0	0	3	3
0	0	0	0	0	0	0	12	12
0	0	0	0	0	0	0	3	3
0	1	0	0	0	0	0	0	1
0	0	0	0	5	0	0	0	5
0	0	0	0	0	0	0	3	3
0	0	0	0	0	20	0	0	20
0	0	0	0	0	0	0	15	15
0	0	0	0	0	0	0	4	4
0	0	0	0	0	0	0	9	9
0	0	0	0	0	0	0	3	3
0	0	0	0	0	0	0	32	32
0	0	0	0	0	0	0	17	17
0	0	0	0	0	0	0	26	26
0	0	0	0	0	59	0	0	59
0	1	0	0	0	0	0	89	90
0	0	0	0	0	53	0	0	53
0	1	0	0	0	37	0	6	44
0	0	84	0	15	0	0	0	99
0	0	0	0	0	0	0	9	9
0	0	33	0	10	0	0	0	43
0	0	0	0	0	0	0	5	5
0	0	46	0	0	0	0	0	46
0	0	0	0	5	0	0	0	5
0	0	0	0	0	0	0	5	5
0	0	0	0	0	3	0	151	154
0	0	13	0	0	0	0	0	13
