# Supplementary material for: Composition and Functional Characteristics and Influencing Factors of Bacterioplankton Community in the Huangshui River, China
Source: Microorganisms. 2021 Oct 29;9(11):2260. doi: 10.3390/microorganisms9112260 (PMC8623840; doi:10.3390/microorganisms9112260)
Supplement: Supplementary file 1 [file microorganisms-09-02260-s001.zip › Table S2.pdf]

Table S2. Composition of the top 6 microorganism groups in each sampling at class level

	Gammaproteobacteria	Alphaproteobacteria	Clostridia	Bacilli	Bacterodia
HS1	13.59%	86.20%	0.09%	0.03%	0.02%
HS2	28.78%	70.91%	0.11%	0.04%	0.01%
HS3	29.19%	53.27%	3.69%	1.56%	2.14%
HS4	21.94%	8.68%	28.96%	17.12%	4.25%
HS5	3.92%	95.93%	0%	0%	0.01%
HS6	55.16%	5.52%	5.46%	2.04%	7.11%
HS7	0.73%	93.53%	0.51%	4.64%	0.05%
HS8	13.85%	33.29%	17.94%	7.71%	5.93%

Actinobacteria
0.01%
0.01%
1.42%
7.93%
0%
3.77%
0.05%
4.13%
