# Supplementary material for: Composition and Functional Characteristics and Influencing Factors of Bacterioplankton Community in the Huangshui River, China
Source: Microorganisms. 2021 Oct 29;9(11):2260. doi: 10.3390/microorganisms9112260 (PMC8623840; doi:10.3390/microorganisms9112260)
Supplement: Supplementary file 1 [file microorganisms-09-02260-s001.zip › Table S3.pdf]

Table S3. Composition of the top 6 microorganism groups in each sampling at genus level

	Acidiphillum	Acidocella	Metallibacerium	Acinetobacter	Pseudomonas	Aeromonas
HS1	86.16%	0%	11.36%	0.02%	0%	0%
HS2	70.87%	0%	28.43%	0.02%	0%	0%
HS3	49.52%	0%	0%	5.85%	18.15%	0%
HS4	0.92%	0.26%	0.12%	7.54%	4.15%	0.16%
HS5	0.03%	95.90%	1.44%	0%	0%	0%
HS6	0.20%	0.45%	0.09%	3.05%	1.56%	19.77%
HS7	31.58%	60.91%	0%	0.33%	0.08%	0%
HS8	0.19%	0.33%	0.02%	10%	2.01%	0.24%
