# Supplementary material for: Composition and Functional Characteristics and Influencing Factors of Bacterioplankton Community in the Huangshui River, China
Source: Microorganisms. 2021 Oct 29;9(11):2260. doi: 10.3390/microorganisms9112260 (PMC8623840; doi:10.3390/microorganisms9112260)
Supplement: Supplementary file 1 [file microorganisms-09-02260-s001.zip › Table S4.pdf]

Pathway le	Pathway le	Pathway le	Descriptior	HS1	HS2	HS3	HS5	HS4
Metabolisn	Global and	ko01230	Biosynthesi	4722575	4645306	4544721	2634222	3957260
Environmei	Membrane	ko02010	ABC transp	5713928	4939278	5316849	2419601	3286095
Metabolisn	Global and	ko01200	Carbon me	4986737	4700747	4468782	2670898	3307501
Environmei	Signal trans	ko02020	Two-compo	3169828	3203181	3511011	1734367	2411175
Cellular Prc	Cellular cor	ko02024	Quorum se	3855885	3340139	3123832	1883080	1893100
Metabolisn	Nucleotide	ko00230	Purine met	2835852	2778188	2636391	1402785	2289648
Genetic Inf	Translation	ko03010	Ribosome	2260468	2290189	2098737	1225361	2121581
Metabolisn	Energy met	ko00190	Oxidative p	2661530	2534805	2158153	1111704	1299192
Metabolisn	Nucleotide	ko00240	Pyrimidine	1827633	1797921	1777199	969540.1	1810242
Environmei	Membrane	ko03070	Bacterial se	2482155	2244956	1929743	1130549	761421.8
Metabolisn	Metabolisn	ko00860	Porphyrin a	2391273	2099662	1880711	1164371	1005089
Metabolisn	Carbohydra	ko00630	Glyoxylate	1834448	1706415	1732107	926158.4	1061489
Metabolisn	Carbohydra	ko00620	Pyruvate m	1555820	1510421	1514375	935564.3	1340164
Metabolisn	Amino acid	ko00260	Glycine, sei	1807430	1700406	1628239	930592.4	1053508
Metabolisn	Energy met	ko00720	Carbon fixa	1594088	1495905	1397091	862042.3	1098078
Metabolisn	Carbohydra	ko00010	Glycolysis /	1528355	1435551	1381296	719821.3	1232097
Metabolisn	Amino acid	ko00270	Cysteine ar	1531493	1515514	1316217	721926.5	1071194
Metabolisn	Energy met	ko00680	Methane m	1556631	1384927	1264019	858200.9	811772.8
Metabolisn	Carbohydra	ko00640	Propanoate	1419866	1320696	1311567	715068.3	948224.9
Metabolisn	Carbohydra	ko00650	Butanoate	1373695	1276558	1319820	792751	879449.5
Metabolisn	Global and	ko01212	Fatty acid r	1252929	1216279	1216688	795114.6	932042.4
Genetic Inf	Translation	ko00970	Aminoacyl-	1189965	1169527	1082269	652064.9	1012186
Metabolisn	Carbohydra	ko00520	Amino suga	1125526	1064428	1074639	524458.5	1118511
Metabolisn	Amino acid	ko00250	Alanine, as	1234920	1167244	1141890	602472.5	943946.3
Cellular Prc	Cell motilit	ko02040	Flagellar as	1109698	1176285	1052368	603512.9	861564.7
Metabolisn	Global and	ko01210	2-Oxocarbo	1064276	1050718	1016526	648245.7	783071.4
Metabolisn	Amino acid	ko00280	Valine, leuc	1109986	1059860	1102146	664798.1	767394.8
Metabolisn	Carbohydra	ko00020	Citrate cycl	1108093	1105092	1019004	562604.9	760692.4
Cellular Prc	Cell motilit	ko02030	Bacterial ch	1196608	1148126	1098590	541087.6	679137.2
Metabolisn	Amino acid	ko00330	Arginine ar	1174451	1085254	1101534	611916.4	663209.2
Metabolisn	Energy met	ko00920	Sulfur meta	1346209	1190228	1225999	390169.9	602551
Genetic Inf	Replication	ko03440	Homologou	954763.3	958707.9	940552.3	459809.2	852551
Metabolisn	Carbohydra	ko00030	Pentose ph	985843.5	919582.8	929261.1	491033.2	759318.3
Metabolisn	Lipid metal	ko00061	Fatty acid b	895338.1	888556.6	818083.9	551885.7	710823.6
Metabolisn	Carbohydra	ko00500	Starch and	835369	728456.9	798943.9	415137.9	1009242
Metabolisn	Amino acid	ko00400	Phenylalan	795516.8	821650.7	808858.5	457044.4	759700.1
Cellular Prc	Cellular cor	ko05111	Biofilm forr	1014409	1004262	950796.1	359116.8	442588.2
Metabolisn	Metabolisn	ko00480	Glutathione	1109310	1047432	900304.2	571246.5	376282.7
Genetic Inf	Replication	ko03430	Mismatch r	846350	821986.9	850730.9	385557.6	777103.2
Metabolisn	Amino acid	ko00360	Phenylalan	1054881	922261.2	1006218	462440.3	492474.1
Metabolisn	Glycan bios	ko00550	Peptidoglyc	752712.7	778868.4	714302	412164	795935.8
Metabolisn	Metabolisn	ko00770	Pantothena	916529.1	881582.2	789438.3	469119.1	575940.3
Metabolisn	Lipid metal	ko00564	Glyceropho	874522.6	838251.1	805259.7	427720.8	624225
Metabolisn	Lipid metal	ko00071	Fatty acid c	880467.9	805194.5	825538.9	503139.6	514748.6
Metabolisn	Metabolisn	ko00760	Nicotinate	860741.1	812420.9	779466.8	423152.1	548221.4
Metabolisn	Amino acid	ko00220	Arginine bi	857007.8	800377.7	837005.6	401560.2	563305.6

Genetic Inf	Folding, soı	ko03060	Protein exp	805222.7	789433.7	703274.3	406341.5	617328.6
Metabolisn	Amino acid	ko00380	Tryptophar	942483.3	897384.8	847151.6	464180.8	425655.3
Metabolisn	Carbohydra	ko00051	Fructose ar	984911.6	862191.8	806230.3	308023.7	523154.2
Genetic Inf	Replication	ko03030	DNA replica	757839.6	733996.7	737588.8	362102.6	654135
Metabolisn	Amino acid	ko00340	Histidine m	764808.3	757312.2	686708	399769.1	525731.7
Genetic Inf	Folding, soı	ko03018	RNA degrac	729052.9	727324.6	638655.1	362214.4	562141.4
Metabolisn	Metabolisn	ko00790	Folate bios	631872.6	672482.4	658480.4	327864.3	602876.5
Metabolisn	Energy met	ko00710	Carbon fixa	782187.9	727251.7	654597.3	361275.4	492847.7
Metabolisn	Xenobiotic:	ko00362	Benzoate d	713885.7	643843.1	904661.7	435957.1	452173.3
Cellular Prc	Cell growth	ko04112	Cell cycle -	702500	665046.1	636469.1	356822.9	531875.7
Metabolisn	Metabolisn	ko00900	Terpenoid I	706980.2	676967.9	633595.3	359756.7	545774.8
Metabolisn	Energy met	ko00910	Nitrogen m	722315	645658.9	726990.3	318983.1	463039.6
Metabolisn	Amino acid	ko00290	Valine, leuc	679411.9	670873.6	608754.6	421365.9	427379.1
Metabolisn	Metabolisn	ko00780	Biotin meta	610280.9	621835.8	571399.6	420132.3	457544.8
Metabolisn	Amino acid	ko00300	Lysine bios	556865.4	553907.7	562822.6	336443.7	556928.9
Human Dis	Drug resist	ko01501	beta-Lactar	557273.3	553945.1	557720.8	270419.9	458912.2
Metabolisn	Amino acid	ko00310	Lysine degr	600544.9	551081.2	586664.4	349953.6	393650.4
Metabolisn	Metabolisn	ko00670	One carbor	523033.7	534818.3	541333.3	275329.7	472347.2
Metabolisn	Amino acid	ko00350	Tyrosine m	689179.7	638007.4	570154	290417	310429.5
Metabolisn	Metabolisn	ko00730	Thiamine n	508811.7	497536.7	486569	251326.1	512794.8
Metabolisn	Glycan bios	ko00540	Lipopolysac	569291	578369.7	556038.1	302614.2	232853.7
Cellular Prc	Cellular cor	ko02025	Biofilm forr	610349.1	632523.4	614893.7	274270.6	254063.6
Metabolisn	Metabolisn	ko00450	Selenocom	576047.5	545855.7	487175.8	205570.7	394146.8
Human Dis	Drug resist	ko01503	Cationic an	522789.9	534229.4	486254.1	251117.9	318181.4
Metabolisn	Metabolisn	ko00410	beta-Alanir	555098.5	495774.3	519180.1	305503.1	327293.1
Genetic Inf	Replication	ko03410	Base excisi	444457	460965.1	482474.7	228459.9	445221.9
Metabolisn	Carbohydra	ko00052	Galactose r	453888.7	428863.4	433957.1	157884.8	518534.4
Cellular Prc	Cellular cor	ko02026	Biofilm forr	422958.2	399293.5	532946.6	230779.3	368014
Genetic Inf	Folding, soı	ko04122	Sulfur relay	455746.8	428984.3	475262.1	228275	371148.7
Metabolisn	Global and	ko01220	Degradatio	464457.7	396327.3	599890.8	259314.8	272535.9
Human Dis	Drug resist	ko01524	Platinum d	597466.5	538689.5	438051.6	306139.7	130988.2
Metabolisn	Xenobiotic:	ko00982	Drug metal	597191.5	538326.3	455577.5	261937.7	122473.5
Genetic Inf	Replication	ko03420	Nucleotide	379323.2	367346.3	398477.4	181133.7	390396.1
Human Dis	Cardiovasc	ko05418	Fluid shear	495744.7	471222.2	424887.3	243468.9	212169.8
Cellular Prc	Transport a	ko04146	Peroxisome	446421.5	415314	399933	259846.2	265428.4
Metabolisn	Energy met	ko00195	Photosynth	384436.6	379620.2	335728.5	202690.9	324663
Metabolisn	Xenobiotic:	ko00627	Aminobenz	510061.4	451643.3	434898.7	260559.2	193953.3
Metabolisn	Xenobiotic:	ko00980	Metabolisn	559231.5	507425.4	415882.3	240993.2	114599
Metabolisn	Lipid metal	ko00561	Glycerolipi	298039.3	293005	332934.5	157275.8	419206.8
Metabolisn	Biosynthes	ko00261	Monobacta	382516.8	378609.8	329373.3	200354.5	254990.4
Metabolisn	Carbohydra	ko00660	C5-Branche	340346.1	335673.8	330687.6	221821.5	248792.1
Environme	Membrane	ko02060	Phosphotra	394150.5	347539.6	308814.5	89119.91	377758.4
Human Dis	Infectious c	ko05134	Legionellos	436286.2	390563.1	323941	220960.9	178882.6
Metabolisn	Metabolisn	ko00130	Ubiquinone	287225	323858.4	330181.6	163871.3	260262.5
Metabolisn	Biosynthes	ko00521	Streptomyc	341266.3	335791.2	335643.5	182130.7	248682.8
Metabolisn	Metabolisn	ko00740	Riboflavin r	334224.4	322965.8	339734.3	177042.2	253089.7
Metabolisn	Lipid metal	ko01040	Biosynthesi	299947.8	303949.2	290918.1	261463.8	215602.9

Metabolisn	Metabolisn	ko00281	Geraniol de	331389.1	321821.8	380426.5	179303.7	175614.6
Metabolisn	Carbohydra	ko00040	Pentose an	356792.3	316359	283484.4	110104.8	254969
Metabolisn	Xenobiotics	ko00625	Chloroalkai	393522.1	346715.1	303463.1	192831.9	160355.1
Human Dis	Cancers: O	ko05230	Central car	330916.4	310630.3	287715.7	154342.5	236699.9
Metabolisn	Metabolisn	ko00750	Vitamin B6	329041.1	310243.2	291021.1	176415.4	172890.5
Human Dis	Cancers: O	ko05204	Chemical ca	408520.2	383799.1	313991.6	197949.5	90431.17
Metabolisn	Lipid metal	ko00072	Synthesis a	321539.1	297141.5	322249.6	177041.6	188095.5
Human Dis	Infectious c	ko05152	Tuberculos	361320.4	328732.4	277088.8	176829	155966.4
Metabolisn	Xenobiotics	ko00643	Styrene deg	397184.4	358588.2	323364	153662.8	95483.95
Organismal	Aging	ko04212	Longevity r	258792	261055.9	254643.8	135166.5	242163.7
Human Dis	Neurodege	ko05016	Huntington	361620.3	328109.8	266228.6	178035.1	110765.8
Human Dis	Neurodege	ko05010	Alzheimer's	361860.3	328121.8	269155	177967.2	103611.1
Human Dis	Drug resista	ko01502	Vancomyci	221766.3	230415.5	219199	113161.2	261251.8
Metabolisn	Carbohydra	ko00562	Inositol pho	313409.8	272767.7	252056.2	153515.4	138794.9
Metabolisn	Xenobiotics	ko00633	Nitrotoluer	377130.4	309387	248193.3	190676.9	89283.85
Organismal	Endocrine s	ko04922	Glucagon s	250828.6	236581.2	215489.3	132710.2	213554.7
Metabolisn	Xenobiotics	ko00983	Drug metal	178137.6	186782.3	189466.6	68458.68	267645.5
Environmer	Signal trans	ko04066	HIF-1 signa	250133.2	236361.8	208660.7	131570.8	174104.5
Metabolisn	Metabolisn	ko00460	Cyanoamin	210192.3	204968.9	219047.1	110794.3	181375.6
Metabolisn	Carbohydra	ko00053	Ascorbate a	242494.4	223192.9	234447.6	129894.7	155159.1
Organismal	Endocrine s	ko03320	PPAR signa	204138	192296.3	191183.6	132297.2	168924.7
Metabolisn	Metabolisn	ko00430	Taurine and	210038.4	204974.4	211604	109706.6	170848.1
Human Dis	Neurodege	ko05012	Parkinson's	318238	284481.8	218012.8	155247.5	44109.81
Metabolisn	Metabolisn	ko00440	Phosphona	307771.6	260507.8	215211.3	169286	59881.45
Human Dis	Endocrine a	ko04932	Non-alcoho	280625	253656.8	196327.8	155234.5	43708.81
Human Dis	Cancers: O	ko05200	Pathways ir	274773	241576	190491.1	132543.6	69600.99
Cellular Pro	Cell growth	ko04214	Apoptosis -	269180.3	228981.6	191633.7	152788	54475.9
Metabolisn	Xenobiotics	ko00930	Caprolactai	236367.1	210275.2	234058.8	107191.8	87394.46
Genetic Inf	Transcriptio	ko03020	RNA polym	173043.6	174233.2	157893	90848.81	162502.5
Human Dis	Drug resista	ko01523	Antifolate r	139942.2	155652.5	180835.2	71663.41	168600.6
Organismal	Environmer	ko04626	Plant-patho	168295.4	162100.8	157484.6	90206.95	131446.7
Metabolisn	Metabolisn	ko00523	Polyketide	173287.6	174111.6	164411.8	90857.71	111288.6
Metabolisn	Biosynthes	ko00401	Novobiocin	168301.3	161771.9	148028.7	90205.06	140725
Metabolisn	Metabolisn	ko00906	Carotenoid	263451	216289.5	177796	126791.7	35894.99
Metabolisn	Xenobiotics	ko00361	Chlorocyclo	199029.3	179542.2	207652.5	129258.1	71241.99
Metabolisn	Xenobiotics	ko00364	Fluorobenz	231304.3	197835.4	206495.5	107599.2	45382.91
Metabolisn	Biosynthes	ko00960	Tropane, pi	172732.5	173920.6	150532.6	68806.23	127925.1
Environmer	Signal trans	ko04070	Phosphatid	167872.6	161626	155255.6	90179.83	101962.1
Human Dis	Neurodege	ko05014	Amyotroph	193867.2	167234.3	156779.1	108764	79721.25
Organismal	Immune sy	ko04621	NOD-like re	130100.5	130907.7	133740.1	68209.84	140609.8
Metabolisn	Metabolisn	ko00903	Limonene a	123182.2	117792	160457.6	107223.9	130960.7
Metabolisn	Xenobiotics	ko00791	Atrazine de	226308.2	185450	164476.4	108072.5	43183.68
Organismal	Aging	ko04213	Longevity r	97120.45	112228.7	126882	67723.47	162518.3
Metabolisn	Xenobiotics	ko00626	Naphthaler	194295.4	167176.1	141560.3	66806.13	69417.71
Metabolisn	Metabolisn	ko00471	D-Glutamir	87254.2	87504	121946.7	47705.35	151456.4
Metabolisn	Metabolisn	ko00473	D-Alanine r	124272.7	118136	116879.3	67501.04	122043.4
Metabolisn	Metabolisn	ko00830	Retinol me	194233.4	167088.2	137528.2	65741.16	47656.07

Organismal	Nervous sy	ko04727	GABAergic	119468.8	105617.7	156222.3	65747.34	103037.8
Environme	Signal trans	ko04016	MAPK signa	92772.81	99845.91	113922.4	88039.33	112778.9
Organismal	Nervous sy	ko04724	Glutamater	118943.3	105488.9	155950.6	65747.34	100234.2
Metabolism	Biosynthes	ko00950	Isoquinolin	156524.9	136294.9	145230.5	64630.17	78445.15
Cellular Pro	Cell growth	ko04210	Apoptosis	188352.8	154597.1	135016.1	87132.4	26098.1
Human Dis	Cancers: Sp	ko05222	Small cell lu	188340	154589.7	117309.6	87126	19167.82
Human Dis	Infectious c	ko05164	Influenza A	188340	154589.7	117304.6	87126	19167.82
Human Dis	Infectious c	ko05168	Herpes sim	188340	154589.7	117299.6	87126	19167.82
Human Dis	Infectious c	ko05161	Hepatitis B	188340	154589.7	117299.6	87126	19167.82
Human Dis	Infectious c	ko05145	Toxoplasm	188340	154589.7	117299.6	87126	19167.82
Cellular Pro	Cell growth	ko04215	Apoptosis -	188340	154589.7	117299.6	87126	19167.82
Human Dis	Cancers: Sp	ko05210	Colorectal c	188340	154589.7	117299.6	87126	19167.82
Human Dis	Cardiovasc	ko05416	Viral myoca	188340	154589.7	117299.6	87126	19167.82
Cellular Pro	Cell growth	ko04115	p53 signali	188340	154589.7	117299.6	87126	19167.82
Organismal	Endocrine s	ko04920	Adipocytok	123566.8	117780.5	101407.1	65395.1	74059.99
Cellular Pro	Cell growth	ko04216	Ferroptosis	118577.6	105401	100896.1	64714.58	84808.08
Metabolism	Xenobiotics	ko00623	Toluene de	118773.3	105249.5	155738.4	65709.16	48614.38
Human Dis	Infectious c	ko05120	Epithelial c	114324	93258.2	105004.7	44157.9	81912.41
Human Dis	Endocrine a	ko04931	Insulin resi	87162.03	87300.39	83152.42	45463.43	95351.73
Environme	Signal trans	ko04152	AMPK signa	96014.67	111767.6	72299.6	26994.55	69143.76
Metabolism	Metabolism	ko00785	Lipoic acid	87324.53	87199	77839.56	45428.57	72103.11
Human Dis	Infectious c	ko05132	Salmonella	43769.28	44083.52	66446.9	66764.61	85689.33
Metabolism	Biosynthes	ko00940	Phenylprop	76078	62141.43	97546.61	23246.61	74076.41
Metabolism	Biosynthes	ko00405	Phenazine	91486.28	99379.33	77637.1	46038.34	56743.62
Metabolism	Biosynthes	ko00525	Acarbose a	86481.28	87052.57	78772.05	45430.09	54534.08
Metabolism	Biosynthes	ko00332	Carbapene	87192.28	87309.34	76053.84	45420.34	62049.24
Organismal	Circulatory	ko04260	Cardiac mu	92284	99066.1	78829.23	68107.5	24411.99
Environme	Signal trans	ko04068	FoxO signa	53730.81	68553.19	78580.68	44965.14	93180.62
Organismal	Endocrine s	ko04910	Insulin sign	87003.78	87173.9	76389.47	25629.95	67595.75
Human Dis	Infectious c	ko05133	Pertussis	86555.64	86954.41	130449.2	24451.7	37279.97
Organismal	Aging	ko04211	Longevity r	48693.67	56123.68	75410.21	44281.14	85376.99
Human Dis	Endocrine a	ko04940	Type I diab	81403.89	74566.7	62411.24	43655.58	46670.25
Metabolism	Lipid metab	ko00565	Ether lipid r	118433.9	105178.3	71469.29	43588.25	22785.28
Genetic Inf	Folding, sor	ko04141	Protein pro	48763.39	56368.22	45758.29	45460.45	60659.45
Organismal	Endocrine s	ko04918	Thyroid hor	48492.28	56075.22	67361.79	45406.51	47686.15
Organismal	Endocrine s	ko04919	Thyroid hor	113393.8	92741.8	67980.86	42958.25	12797.41
Metabolism	Lipid metab	ko00592	alpha-Linol	85326.28	86638.74	62199.89	23351.83	32103.28
Metabolism	Glycan bios	ko00510	N-Glycan b	85253	86632.36	57189.3	43181.52	18847.24
Genetic Inf	Translation	ko03008	Ribosome b	48870.53	56113.01	59025.64	24479.42	52243
Metabolism	Metabolism	ko01051	Biosynthesi	43375.89	43701.56	61172.03	22721.92	75741.69
Genetic Inf	Translation	ko03013	RNA transp	43854.64	43772.69	67323.2	22722.14	65064.28
Metabolism	Xenobiotics	ko00622	Xylene deg	43040.5	43383.53	96073.2	23826.5	61159.98
Metabolism	Metabolism	ko00981	Insect horn	42804.39	43520.46	45560.75	42582.05	67340.79
Metabolism	Xenobiotics	ko00642	Ethylbenze	80305.14	74222.8	59685.87	23783.83	26788.81
Human Dis	Endocrine a	ko04930	Type II diab	44138.75	43727.09	48736	23768.56	51244.61
Human Dis	Cancers: Ov	ko05203	Viral carcin	44137.75	43727.09	48691.5	23768.56	50875.11
Metabolism	Glycan bios	ko00531	Glycosamir	43261.64	43574.66	42854.47	23798.28	37359.64

Metabolisn	Lipid metal	ko00600	Sphingolipi	38312.75	31201.96	38717.14	20969.37	48197.99
Metabolisn	Biosynthes	ko00966	Glucosinola	43238.14	43533.8	39086.29	43630.67	30876.31
Environme	Signal trans	ko04013	MAPK signa	43274.39	43554.5	42110.25	22715.36	45751.79
Environme	Signal trans	ko04151	PI3K-Akt sig	48262.53	55941.18	39370.39	23393.42	35285.25
Metabolisn	Metabolisn	ko00908	Zeatin bios	43262.39	43557	39717.25	22713.62	39053.54
Human Dis	Cancers: O	ko05205	Proteoglyca	43259.89	43550.34	38761.76	22712.42	38979.2
Metabolisn	Metabolisn	ko00909	Sesquiterpe	76278.5	62023.4	43742.07	41881.99	5422.66
Metabolisn	Metabolisn	ko01055	Biosynthes	43250.14	43533.19	39689.47	22716.64	28649.2
Organisma	Endocrine s	ko04614	Renin-angi	80287.5	74113.5	51747.04	659.86	15771.24
Metabolisn	Xenobiotics	ko00621	Dioxin degr	37674.5	30922.83	58346.82	23188.5	34667.99
Organisma	Immune sy	ko04657	IL-17 signal	43223.39	43512.34	36052.09	22709.42	27330.62
Organisma	Endocrine s	ko04915	Estrogen si	43223.39	43512.34	36042.09	22709.42	27330.62
Human Dis	Cancers: Sp	ko05215	Prostate ca	43223.39	43512.34	36042.09	22709.42	27330.62
Organisma	Immune sy	ko04612	Antigen pro	43223.39	43512.34	36042.09	22709.42	27330.62
Organisma	Endocrine s	ko04914	Progestero	43223.39	43512.34	36042.09	22709.42	27330.62
Organisma	Immune sy	ko04659	Th17 cell di	43223.39	43512.34	36042.09	22709.42	27330.62
Metabolisn	Lipid metal	ko00590	Arachidoni	15297.42	37452.66	36922.79	25075.01	41903.04
Human Dis	Cancers: Sp	ko05211	Renal cell c	43209.64	43474	37116.85	22707.83	23022.22
Metabolisn	Glycan bios	ko00511	Other glyca	245.75	274.03	24822.87	51.41	53349.43
Metabolisn	Metabolisn	ko00472	D-Arginine	37673.5	30939.9	33696.37	43004.5	22377.83
Metabolisn	Biosynthes	ko00965	Betalain bio	42660.14	43316.34	41906.45	22692.64	15573.13
Human Dis	Immune di	ko05340	Primary im	5668.28	12683.38	28696.17	1805.17	54306.48
Metabolisn	Biosynthes	ko00524	Neomycin,	5696.75	12731.69	22057.9	22694.08	52339.71
Organisma	Digestive s	ko04978	Mineral abs	38734.14	31229.17	35346.04	20999.84	25723.68
Organisma	Nervous sy	ko04728	Dopaminer	37962	30906.56	47927.39	20946.5	10041.58
Organisma	Nervous sy	ko04726	Serotonerg	37961	30905.56	47827.39	20946.5	10025.58
Human Dis	Substance	ko05034	Alcoholism	37961	30906.56	47888.39	20946.5	10025.58
Human Dis	Substance	ko05031	Amphetam	37961	30906.56	47888.39	20946.5	10025.58
Human Dis	Substance	ko05030	Cocaine ad	37961	30906.56	47888.39	20946.5	10025.58
Environme	Signal trans	ko04011	MAPK signa	5419.28	12570.18	33494.29	21565.78	39625.2
Human Dis	Infectious	ko05143	African tryp	37641	30912.17	42086.55	35	13827.91
Human Dis	Infectious	ko05146	Amoebiasis	42669.25	43288.16	25799.91	1760.39	16174
Human Dis	Cancers: O	ko05206	MicroRNAs	5136.39	12565.47	17968.05	707.32	46662.45
Human Dis	Infectious	ko05142	Chagas dise	37647.5	30923.5	35576.65	17.33	13040.74
Metabolisn	Lipid metal	ko00100	Steroid bio	38141	31012.7	22223.2	20957.5	4065.17
Metabolisn	Xenobiotics	ko00624	Polycyclic a	34	138.34	20844.89	42923.33	20103.06
Organisma	Excretory s	ko04964	Proximal tu	5121.53	12538.5	18690.11	1821.58	36841.57
Metabolisn	Xenobiotics	ko00984	Steroid deg	50.5	11.2	73094.04	18	37326.83
Metabolisn	Biosynthes	ko00311	Penicillin ar	10058.5	24911.55	26639.54	1270.66	26850.93
Genetic Inf	Replication	ko03450	Non-homol	5145	12507.26	25495.92	663.28	36363.16
Human Dis	Infectious	ko05150	Staphyloco	142.25	178	14051.51	20931.89	37770.03
Human Dis	Cancers: O	ko05231	Choline me	5602.39	12629.7	15046.68	1753.58	23474.96
Environme	Signal trans	ko04072	Phospholip	5602.39	12629.7	15020.68	1753.58	23474.96
Cellular Pro	Transport a	ko04142	Lysosome	113	126.7	11127.17	24.27	26088.86
Metabolisn	Glycan bios	ko00603	Glycosphin	117	123.16	9110.37	16.48	26165.73
Metabolisn	Metabolisn	ko01053	Biosynthes	267.14	195.47	22189.04	14.2	33220.14
Cellular Pro	Cell growth	ko04113	Meiosis - ye	5057	12466	6234.74	657.66	15545.24

Metabolism	Lipid metal	ko00591	Linoleic aci	9995.28	24829.61	4902.36	1270	9715.61
Metabolism	Biosynthes	ko00404	Staurospor	5106	12507.5	8704.89	631	12285
Organismal	Endocrine s	ko04917	Prolactin si	60.75	73.66	5040.95	5.72	19305.73
Human Dis	Neurodege	ko05020	Prion disea	107.89	74.5	6176.54	72.25	20904.23
Metabolism	Lipid metal	ko00140	Steroid hor	24	17.03	11516.04	8.32	12547.82
Metabolism	Metabolism	ko01054	Nonribosor	39.89	49.34	4361.63	54.25	15713.23
Organismal	Digestive sy	ko04974	Protein dig	4990.5	12432.33	4369.92	631.73	7607.16
Metabolism	Lipid metal	ko00120	Primary bil	258.75	183.3	4219.56	16.25	16182.52
Metabolism	Glycan bios	ko00513	Various typ	39	35	3963.62	10	6727.85
Metabolism	Glycan bios	ko00604	Glycosphin	39	34	3887.62	10	6491.85
Metabolism	Lipid metal	ko00121	Secondary	243.75	168	9799.66	19.25	13500.92
Cellular Prc	Transport a	ko04138	Autophagy	41	46.03	3076.21	12	15994.33
Organismal	Digestive sy	ko04973	Carbohydra	33	40.33	3245.04	2.2	13926.34
Human Dis	Cancers: Sp	ko05219	Bladder car	14.5	17.06	2501.02	62	6050.66
Metabolism	Energy met	ko00196	Photosynth	0.014	12	144	22	2976
Genetic Inf	Replication	ko03460	Fanconi an	0.014	0.014	15348	4	3456
Metabolism	Biosynthes	ko00943	Isoflavonoi	4961	12415.66	1526.33	629	322.84
Metabolism	Xenobiotics	ko00363	Bisphenol c	12	6	1440.42	1	5662.08
Metabolism	Biosynthes	ko00945	Stilbenoid,	13.5	6	1537.93	1	5769.66
Genetic Inf	Folding, so	ko03050	Proteasom	15	7	1391	0.014	6996.32
Organismal	Immune sy	ko04622	RIG-I-like re	20.14	26.14	1294.16	0.014	6808.7
Metabolism	Biosynthes	ko00901	Indole alka	1	4	8001.33	2	2084.33
Human Dis	Cardiovasc	ko05410	Hypertroph	12.5	15	2118.83	0.33	2785.58
Organismal	Endocrine s	ko04924	Renin secre	12.5	15	2063.83	0.33	2734.58
Human Dis	Immune di	ko05322	Systemic lu	0.014	2.33	7834.66	2	1746
Genetic Inf	Transcriptio	ko03022	Basal trans	3	5	525.48	0.33	3976.22
Human Dis	Infectious c	ko05100	Bacterial in	1	6	793.66	0.14	3066
Environme	Signal trans	ko04024	cAMP signa	5	3.2	712.2	0.014	3166.83
Metabolism	Xenobiotics	ko00365	Furfural de	0.014	1.2	288.86	0.014	2826.32
Metabolism	Biosynthes	ko00232	Caffeine m	2	4.66	832.84	0.014	1960.67
Metabolism	Biosynthes	ko00944	Flavone an	1	0.014	525.16	1.14	765.5
Organismal	Endocrine s	ko04916	Melanogen	1	0.014	510	1	546.25
Organismal	Nervous sy	ko04723	Retrograde	2	1	209.83	1	129.33
Human Dis	Infectious c	ko05130	Pathogenic	0.014	1	250	1	1980
Environme	Signal trans	ko04071	Sphingolipi	1	0.87	333.7	0.014	1751.5
Environme	Signal trans	ko04020	Calcium sig	0.014	0.5	20.4	1	68
Metabolism	Biosynthes	ko00941	Flavonoid b	2.5	0.014	259.83	0.014	786.33
Human Dis	Infectious c	ko05110	Vibrio chol	0.014	0.014	105	9	90.83
Organismal	Immune sy	ko04640	Hematopoi	1	0.014	82	0.014	936
Organismal	Digestive sy	ko04976	Bile secreti	1	0.6	148.35	0.014	107.25
Metabolism	Metabolism	ko01057	Biosynthesi	0.014	1	28.91	0.014	193
Human Dis	Infectious c	ko05131	Shigellosis	0.014	0.014	9	0.014	660
Metabolism	Metabolism	ko00902	Monoterpe	0.014	0.014	21	0.014	175
Metabolism	Metabolism	ko01052	Type I polyl	1	1	151.53	0.014	116
Cellular Prc	Cellular cor	ko04550	Signaling pa	0.014	0.014	30	0.014	159
Cellular Prc	Transport a	ko04144	Endocytosi	0.014	1.2	198.53	0.014	0.014
Cellular Prc	Transport a	ko04145	Phagosome	0.014	1	199	0.014	0.014

Cellular Prc	Cellular cor	ko04530	Tight juncti	0.014	1	194	0.014	0.014
Cellular Prc	Cellular cor	ko04540	Gap junctic	0.014	1	194	0.014	0.014
Human Dis	Cancers: Sp	ko05220	Chronic my	0.014	0.014	22.57	0.33	80.33
Environme	Signal trans	ko04330	Notch signa	0.014	0.014	22.57	0.33	80.33
Environme	Signal trans	ko04310	Wnt signali	0.014	0.014	22.57	0.33	80.33
Metabolisn	Lipid metal	ko00062	Fatty acid e	0.5	1	26.83	0.014	1
Organismal	Endocrine s	ko04921	Oxytocin si	0.014	0.014	10	0.014	0.014
Organismal	Endocrine s	ko04913	Ovarian ste	0.014	0.014	10	0.014	0.014
Human Dis	Infectious c	ko05140	Leishmania	0.014	0.014	10	0.014	0.014
Environme	Signal trans	ko04064	NF-kappa E	0.014	0.014	10	0.014	0.014
Organismal	Endocrine s	ko04923	Regulation	0.014	0.014	10	0.014	0.014
Environme	Signal trans	ko04668	TNF signali	0.014	0.014	10	0.014	0.014
Environme	Signal trans	ko04370	VEGF signa	0.014	0.014	10	0.014	0.014
Genetic Inf	Transcriptic	ko03040	Spliceosom	0.014	1	71	0.014	0.014
Human Dis	Infectious c	ko05169	Epstein-Bar	0.014	0.014	0.014	0.014	0.014
Metabolisn	Metabolisn	ko01059	Biosynthesi	0.014	0.014	20.16	0.014	10.66
Human Dis	Cardiovasc	ko05414	Dilated car	0.014	0.014	55	0.014	51
Metabolisn	Metabolisn	ko00253	Tetracyclin	0.014	3	4.57	0.014	56
Metabolisn	Metabolisn	ko00522	Biosynthesi	0.014	1	25	0.014	98
Organismal	Digestive s	ko04972	Pancreatic	1	0.014	5	0.014	0.014
Genetic Inf	Translation	ko03015	mRNA surv	1	0.014	1	0.014	21.5
Organismal	Circulatory	ko04261	Adrenergic	1	0.014	0.014	0.014	0.014
Organismal	Excretory s	ko04960	Aldosteron	1	0.014	0.014	0.014	0.014
Organismal	Excretory s	ko04961	Endocrine a	1	0.014	0.014	0.014	0.014
Organismal	Digestive s	ko04971	Gastric acic	1	0.014	0.014	0.014	0.014
Organismal	Endocrine s	ko04911	Insulin secr	1	0.014	0.014	0.014	0.014
Organismal	Digestive s	ko04970	Salivary sec	1	0.014	0.014	0.014	0.014
Environme	Signal trans	ko04022	cGMP-PKG	1	0.014	0.014	0.014	0.014
Environme	Signal trans	ko04075	Plant horm	0.014	0.014	5	0.014	38
Human Dis	Infectious c	ko05166	HTLV-I infe	0.014	0.014	0.014	0.014	0.014
Cellular Prc	Cell motilit	ko04810	Regulation	0.014	0.014	6.33	0.014	0.014
Organismal	Nervous sy	ko04721	Synaptic ve	0.014	0.014	0.014	0.014	7
Organismal	Excretory s	ko04962	Vasopressi	0.014	0.014	0.014	0.014	7
Organismal	Nervous sy	ko04722	Neurotropl	0.014	0.014	0.014	0.014	22.5
Organismal	Developme	ko04380	Osteoclast	0.014	0.014	5	0.014	22.5
Organismal	Immune sy	ko04624	Toll and Im	0.014	0.014	0.014	0.014	13
Environme	Signaling m	ko04080	Neuroactiv	0.014	0.014	5.83	0.014	0.33
Human Dis	Immune di	ko05323	Rheumatoi	0.014	0.014	0.014	0.014	22.5
Environme	Signaling m	ko04512	ECM-recep	0.014	0.014	7.34	0.014	0.014
Organismal	Developme	ko04320	Dorso-vent	0.014	0.014	1	0.014	0.014
Cellular Prc	Cellular cor	ko04510	Focal adhe	0.014	0.014	6	0.014	0.014
Human Dis	Infectious c	ko05160	Hepatitis C	0.014	0.014	0.014	0.014	0.014
Human Dis	Drug resist	ko01521	EGFR tyrosi	0.014	0.014	0.014	0.014	0.014
Environme	Signal trans	ko04150	mTOR signa	0.014	0.014	0.014	0.014	0.014
Environme	Signaling m	ko04514	Cell adhesi	0.014	0.014	2.34	0.014	0.014
Organismal	Immune sy	ko04666	Fc gamma I	0.014	0.2	3.53	0.014	0.014
Organismal	Digestive s	ko04975	Fat digestic	0.014	0.014	0.014	0.014	0.014

Metabolism	Metabolism	ko01056	Biosynthesi	0.014	3	4	0.014	0.014
Human Dis	Cardiovasc	ko05412	Arrhythmo	0.014	0.014	6	0.014	0.014
Cellular Pro	Transport a	ko04139	Mitophagy	0.014	0.014	0.33	0.014	0.014
Organismal	Immune sy	ko04611	Platelet act	0.014	0.014	6.34	0.014	0.014
Environme	Signal trans	ko04371	Apelin sign	0.014	0.014	0.014	0.014	0.014
Cellular Pro	Transport a	ko04140	Autophagy	0.014	0.014	0.014	0.014	0.014
Cellular Pro	Cell growth	ko04110	Cell cycle	0.014	0.014	0.014	0.014	0.014
Human Dis	Infectious c	ko05162	Measles	0.014	0.014	0.014	0.014	0.014
Organismal	Endocrine s	ko04912	GnRH signa	0.014	0.2	3.2	0.014	0.014
Environme	Signal trans	ko04014	Ras signalir	0.014	0.2	3.2	0.014	0.014
Metabolism	Metabolism	ko00905	Brassinoste	0.014	0.014	0.014	0.014	0.014
Organismal	Immune sy	ko04664	Fc epsilon F	0.014	0.014	0.014	0.014	0.014
Human Dis	Substance	ko05032	Morphine a	0.014	0.014	0.83	0.014	0.33
Human Dis	Substance	ko05033	Nicotine ac	0.014	0.014	0.83	0.014	0.33
Metabolism	Biosynthes	ko00331	Clavulanic a	0.014	4	4	0.014	0.014
Human Dis	Endocrine a	ko04933	AGE-RAGE	0.014	0.014	1.34	0.014	0.014
Human Dis	Infectious c	ko05144	Malaria	0.014	0.014	1.34	0.014	0.014
Human Dis	Cancers: Sp	ko05221	Acute myel	0.014	0.014	0.014	0.014	0.014
Organismal	Immune sy	ko04662	B cell recep	0.014	0.014	0.014	0.014	0.014
Human Dis	Cancers: Sp	ko05224	Breast canc	0.014	0.014	0.014	0.014	0.014
Organismal	Immune sy	ko04062	Chemokine	0.014	0.014	0.014	0.014	0.014
Human Dis	Drug resista	ko01522	Endocrine r	0.014	0.014	0.014	0.014	0.014
Human Dis	Cancers: Sp	ko05213	Endometria	0.014	0.014	0.014	0.014	0.014
Environme	Signal trans	ko04012	ErbB signal	0.014	0.014	0.014	0.014	0.014
Human Dis	Cancers: Sp	ko05214	Glioma	0.014	0.014	0.014	0.014	0.014
Environme	Signal trans	ko04630	Jak-STAT si	0.014	0.014	0.014	0.014	0.014
Environme	Signal trans	ko04010	MAPK signa	0.014	0.014	0.014	0.014	0.014
Organismal	Immune sy	ko04650	Natural kill	0.014	0.014	0.014	0.014	0.014
Human Dis	Cancers: Sp	ko05223	Non-small c	0.014	0.014	0.014	0.014	0.014
Organismal	Immune sy	ko04660	T cell recep	0.014	0.014	0.014	0.014	0.014
Environme	Signal trans	ko04015	Rap1 signal	0.014	0.014	5	0.014	0.014
Metabolism	Glycan bios	ko00515	Mannose ty	0.014	0.014	3	0.014	0.014
Metabolism	Glycan bios	ko00514	Other type	0.014	0.014	3	0.014	0.014
Cellular Pro	Cell growth	ko04111	Cell cycle -	0.014	0.014	0.014	0.014	0.014
Organismal	Nervous sy	ko04725	Cholinergic	0.014	0.014	1	0.014	0.014
Environme	Signal trans	ko04390	Hippo signa	0.014	0.014	0.014	0.014	0.014
Environme	Signal trans	ko04391	Hippo signa	0.014	0.014	0.014	0.014	0.014
Organismal	Nervous sy	ko04730	Long-term	0.014	0.014	0.014	0.014	0.014
Cellular Pro	Cell growth	ko04114	Oocyte me	0.014	0.014	0.014	0.014	0.014
Environme	Signal trans	ko04350	TGF-beta si	0.014	0.014	0.014	0.014	0.014

HS6	HS8	HS7
3943574	3628046	3400806
2864953	2820107	3676323
3250706	3054879	3535704
2335245	2492402	2168489
1669791	1775196	2676398
2234500	2060265	1904910
2178012	1933937	1597971
1618810	1323624	1661660
1673173	1595824	1290668
1037882	826123.1	1584509
817033.3	916742	1656899
1026169	1048053	1263906
1320688	1203983	1178684
1014494	976508.7	1243283
1118791	1008494	1140864
1113250	1045479	1039961
908331.3	946737.7	1014005
825531.5	726379.6	1119573
908044.8	872785.5	978218.8
875855.7	849366.8	1016396
985900.3	898659.3	971340.3
1025205	935485.1	856154.7
1099948	968454.3	758893
962967.7	835588.7	840469.3
1103900	930287.8	766954.6
872733.4	786139.2	801894.8
687604.8	762132.6	834481.3
865715.2	725998.3	766496.4
640995.7	727626.5	779795
599073.1	587701.8	825676.6
504821	551823.1	747163.9
869042.5	762002.6	630806.3
661230.8	657237.5	678040
757566.5	647076.9	685916
714406	711981.4	594864.1
793395.4	696985.1	576274.8
799792.5	520406.8	563549.9
469959.4	434867.3	745395
734008.8	669321.8	566666.8
410657.8	474613.5	697102.6
709022.9	689412.3	549032.2
583821.1	547754.9	631614.2
585533.4	564526.2	589792.9
495214.8	508477.6	656633.9
588611.7	514127	579632.9
545952.7	494781.8	575661

642065.3	561435.5	547498.9
358109.7	424695.9	629183.2
488940.3	436840.6	577112.2
616440.7	574407.4	507149.9
531063.3	497374.7	530165.4
616799.9	511097.3	488128.3
625641.6	562120.2	429517.8
517239.2	455373.8	513851.5
353587.2	443314.7	542514.4
507014	535689	492789.5
494372.4	498896.6	487357
463896.2	443419.9	463794.7
481183.7	438615.8	510768.8
537781.5	438491.8	482643.6
520356.5	505222	429520.1
494339.1	431467.9	370938.4
326392.2	381093.5	455400
473761.6	444403.8	359869.3
349887.7	317121.9	432655.2
456778.5	436383.2	353741.7
582478.2	316158.6	365114.3
415287.1	329872.2	366384.5
348467.5	352013.2	341697
474539.5	309714.4	326310.5
291880.9	303811.1	405037.9
401515.3	406148.6	300774.5
427224.6	415932.8	279278
494453.3	348808.4	285263.1
379770.3	324900.1	314624.8
207792.1	258077.3	344782.2
154389.5	185664.9	404327.1
145242.5	159863.6	380637.8
324245.1	340230.9	266251.6
229755.1	231944.8	325748.2
262656.1	253941.5	330824.4
394019.9	292957.3	268609.7
185761.4	189799.8	349618.6
142793.9	151454.8	353032.2
297867.2	331840.8	224523.3
284472.6	237714.3	263382.8
314000	263631	265286.4
301309.3	236404	212729.8
212828.8	198513	299390.5
421304.4	268939.9	201362.6
298992.8	253753.6	236975.3
258046.6	235570	235289.9
250930.9	211836.9	275498.1

211406	182828.3	225640.2
210595.7	210610.7	219952
127505	143843	278457.4
187459.7	186783.3	222940
229262.6	169827.8	231867.1
118580.1	130001.6	264541.7
155812.3	194079.7	236599.7
153713.8	158135.1	241810.9
117683.5	107413.3	242471.6
239388.4	215025.7	178813
145894.8	141383.8	237826.8
145934.4	129006	239254.7
246298.5	221468	151719.4
168929.3	139680.5	217627.7
76119.09	66891.84	263200.3
143350.5	167420.9	181260
265081.4	221446.8	111576
143400.2	134667	180501.2
190220.8	183876.2	148560.9
134843.6	151621.7	177209.4
181194.9	176505.1	163364.7
200093	146857.2	149903.9
89842.75	80797.61	206816.9
41327.33	55694.83	222659.4
89730.75	80646.61	193031.9
80765.99	87268.24	181890.3
69247.66	63494.25	196065.8
79653	89721.54	154592.4
160131.9	144743.2	122573.2
178189.6	160795.5	91879.74
153742.2	140790	116333.1
163263.6	124698	118034.7
135685.5	129476.5	119818.4
26585.15	53668.75	177581.6
48203.84	80225.09	155914.9
33656.88	55565.11	152910
119362.1	112369	104341.7
111084.5	100923.9	117708.7
51052.13	85416.74	138631.8
148718.8	137456	91037.73
101048.3	118057.1	115933.2
26846	39405.78	150229.1
148769.8	146536	74633.45
80780.68	61429.42	120987.2
152645.5	140367	64359.62
110132.3	102825.4	90480.92
51243.09	44751.56	118258.3

107830.7	79458.89	88345.13
106519.2	107284.7	89741.88
96289.5	78324.3	88333.58
60231.02	68674.34	99237.61
25231.92	42696.95	124231.7
16155.07	34926.22	124038.8
16016.07	34897.22	124038.8
16034.07	34882.22	124038.8
15980.07	34882.22	124038.8
15967.07	34882.22	124038.8
15962.07	34882.22	124038.8
15962.07	34882.22	124038.8
15962.07	34882.22	124038.8
15962.07	34882.22	124038.8
82341.71	74618.69	89071.94
81603.73	73048.46	89030.74
32579.55	53990.73	84991.5
68427.11	62539.26	69889.49
80238.6	81167.3	58388.49
103718.2	74007.21	44937.81
81679.81	66222.27	62119.67
128495	80837.54	59349.69
94233.71	88327.53	47146.09
70089.04	60557.5	60327.49
85343.13	62209.1	59999.7
76488.68	61581.23	59947.1
71045.68	45578.39	68946.18
69636.91	84868.59	45014.9
57863.71	60012.23	44638.15
52980.74	37486.4	42136.99
63465.85	73616.96	44872.89
56386.97	40162.81	57286.51
7058.08	23266.44	70445.73
63667.44	57533.74	44209.88
56698.9	51551.7	44066.75
2509.57	12878.92	70180.44
43780.21	33953.2	43399.95
32389.78	18828.26	56874.91
77523.67	49482.43	30424.84
59953.01	59623.23	31122.12
63428.4	51914.2	32128.39
33362.27	52371.19	28781.26
33116.01	56598.89	46361.1
41254.98	27680.82	43275.59
46610.96	42966.3	30230.56
46392.71	42550.8	30224.06
68651.67	41185	28125.12

69663.05	45515.09	28507.96
39528.62	33966	43265.02
42218.93	41882.79	30645.32
39355.83	38052.45	28154.17
42977.11	36403.91	30142.91
41917.11	34802.16	30117.91
4628.16	2118.06	54589.87
46827.81	34722.24	30031.83
8633.81	23442.91	28247.07
23146.26	28375.81	28323.2
33339.61	26451.49	28008.16
33156.61	26407.49	28008.16
33156.61	26407.49	28008.16
33146.61	26407.49	28008.16
33146.61	26407.49	28008.16
33146.61	26407.49	28008.16
38955.44	44829.42	16546.08
31220.98	25909.53	29840.36
99037.99	63808.38	2921.92
14248.42	18241.4	43022.81
19496.13	18315.4	29348.48
64598.95	48909.44	944.31
37932.1	46820.83	16484.19
9835.65	19036.09	29352.95
4161.25	10486.81	27649.1
4285.25	10209.81	27647.1
4141.25	10234.81	27648.1
4131.25	10234.81	27648.1
4131.25	10234.81	27648.1
21278.92	31913.5	14227.57
21273.08	13968.58	14433.84
4936.41	13757.75	15951.5
39787.09	37215.01	3328.92
14758.57	16467.01	14397.08
12499	1985.03	27116.7
19466.24	20943.67	27343.25
37481.02	36652.34	1044.45
13155.83	20586.2	500.37
19837.33	32254.84	894.82
21470.32	32402.23	7635.28
4033.63	24957.3	14031.58
22778.91	23836.55	2464.92
22753.16	23808.55	2464.92
37735.93	28345.57	2484.59
44104.3	21983.08	239.08
19954.63	21400.72	2321.83
30402.82	18872.64	1158.9

11409.28	11388.52	223.53
3483.75	23715.5	175.5
21299.86	16323.8	2386.71
13833.14	18772.35	365.5
14293.74	13129.29	577.65
16177.64	14849.45	653.59
10100.99	10686.92	123.16
14575.72	10683.57	191.91
24856.23	9630.31	100.34
24754.23	9439.31	99.34
13868.18	5803.14	172.69
11367.49	8646.5	105.62
12092.82	9078.22	104.25
18656.58	8106.3	529.65
16658.5	2676	24
1174	1692	68
205.33	1618	15.5
5546.91	5915.5	48.92
5377.41	5668.5	50.84
3583	3724.67	39.01
1054.34	4237.14	141.64
1383.66	1351	38.17
1149.74	5581	60.74
1109.24	5532.5	59.74
658.16	1135	38
1242.87	1788.21	2017.66
800.49	2815.6	32.22
581.83	1584.7	22.37
1034.33	876.18	10.7
461.33	983.67	21.17
755	1953.57	17.16
753.75	2055.5	32.5
3086	269.17	48
445	955	9
372.33	1102.87	34.7
99	64.9	1993.5
496.5	625.5	6.67
1184.5	384	15
65.5	120	5
427.83	464.1	4.35
738.5	157	1
217	210	3
292	225	2
77	300	2.5
394	30	0.014
108.33	181.53	0.2
42	160	0.014

41	160	0.014
32	160	0.014
253.33	25	2.99
243.33	25	2.99
243.33	25	2.99
187.83	51	2
211	44	0.014
198	44	0.014
193	44	0.014
193	44	0.014
193	44	0.014
193	44	0.014
193	44	0.014
88	75	1
234	0.014	0.014
18	156	1.84
40.5	48.5	1
84	45	0.014
1	62	0.014
74	53	1
91	0.014	0.014
72	38	1
71	38	1
71	38	1
71	38	1
71	38	1
71	38	1
71	38	1
63	0.014	0.014
72	0.014	0.014
27	19.33	0.014
8	30.5	0.014
8	30.5	0.014
22	0.5	0.014
14	0.5	0.014
25	0.014	1
13	16.17	0.014
12	0.5	0.014
17	10	0.014
32	0.014	0.014
27	0.014	0.014
29	0.014	0.014
28	0.014	0.014
28	0.014	0.014
15	10	0.014
1.33	21.53	0.2
26	0.014	0.014

18	0.014	0.014
17	0.014	0.014
0.014	19.33	0.014
2	10	0.014
18	0.014	0.014
18	0.014	0.014
18	0.014	0.014
18	0.014	0.014
11.33	2.2	0.2
11.33	2.2	0.2
16	0.014	0.014
15	0.014	0.014
10	1.17	0.014
10	1.17	0.014
4	0.014	0.014
0.014	10	0.014
0.014	10	0.014
10	0.014	0.014
10	0.014	0.014
10	0.014	0.014
10	0.014	0.014
10	0.014	0.014
10	0.014	0.014
10	0.014	0.014
10	0.014	0.014
10	0.014	0.014
10	0.014	0.014
10	0.014	0.014
10	0.014	0.014
10	0.014	0.014
2	0.014	0.014
0.014	0.014	0.014
0.014	0.014	0.014
1	0.014	0.014
0.014	0.014	0.014
1	0.014	0.014
1	0.014	0.014
1	0.014	0.014
1	0.014	0.014
1	0.014	0.014
