# Supplementary figures and images for: Composition and Functional Characteristics and Influencing Factors of Bacterioplankton Community in the Huangshui River, China
Source: Microorganisms. 2021 Oct 29;9(11):2260. doi: 10.3390/microorganisms9112260 (PMC8623840; doi:10.3390/microorganisms9112260)

A

## Rarefaction curves

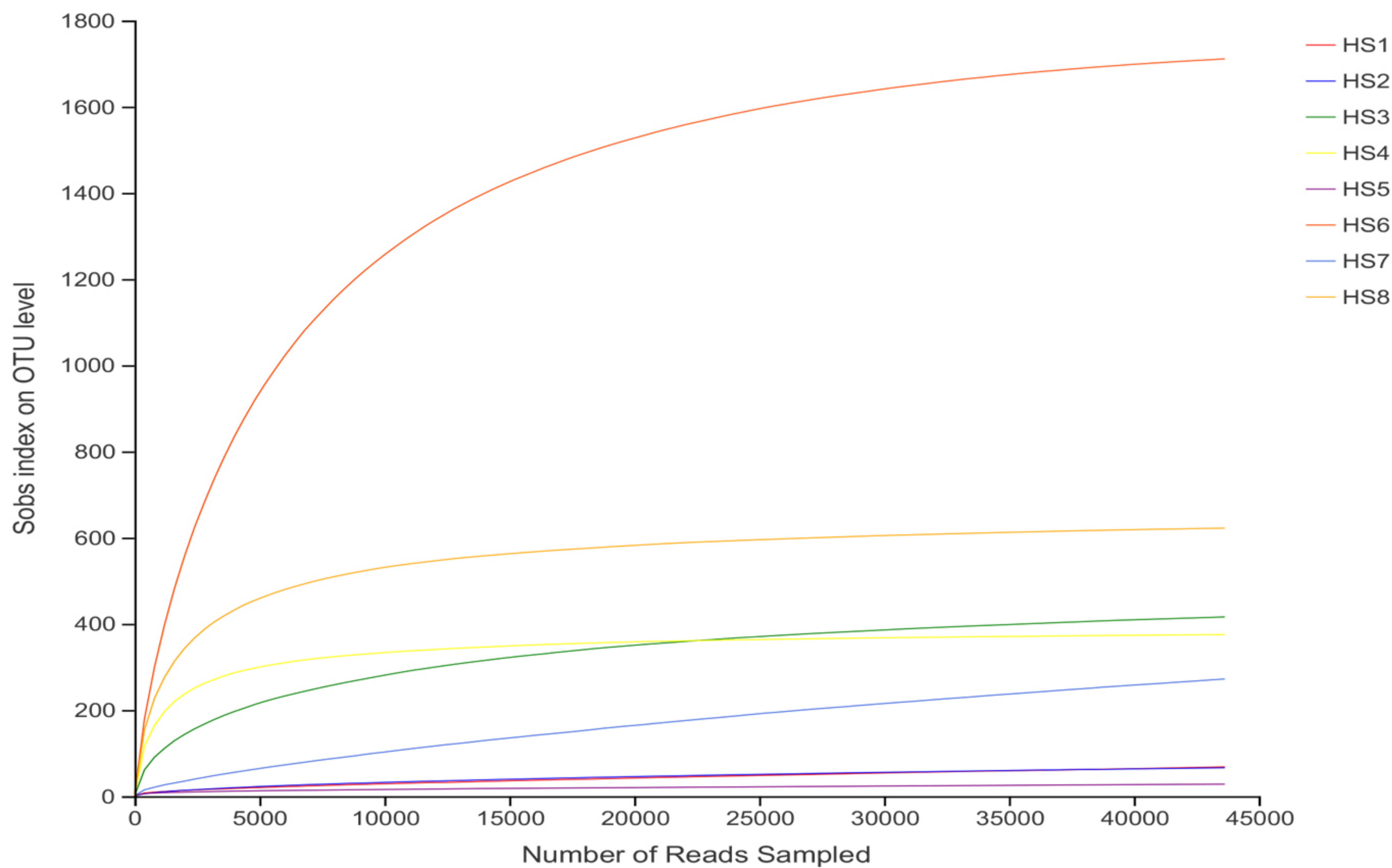

B

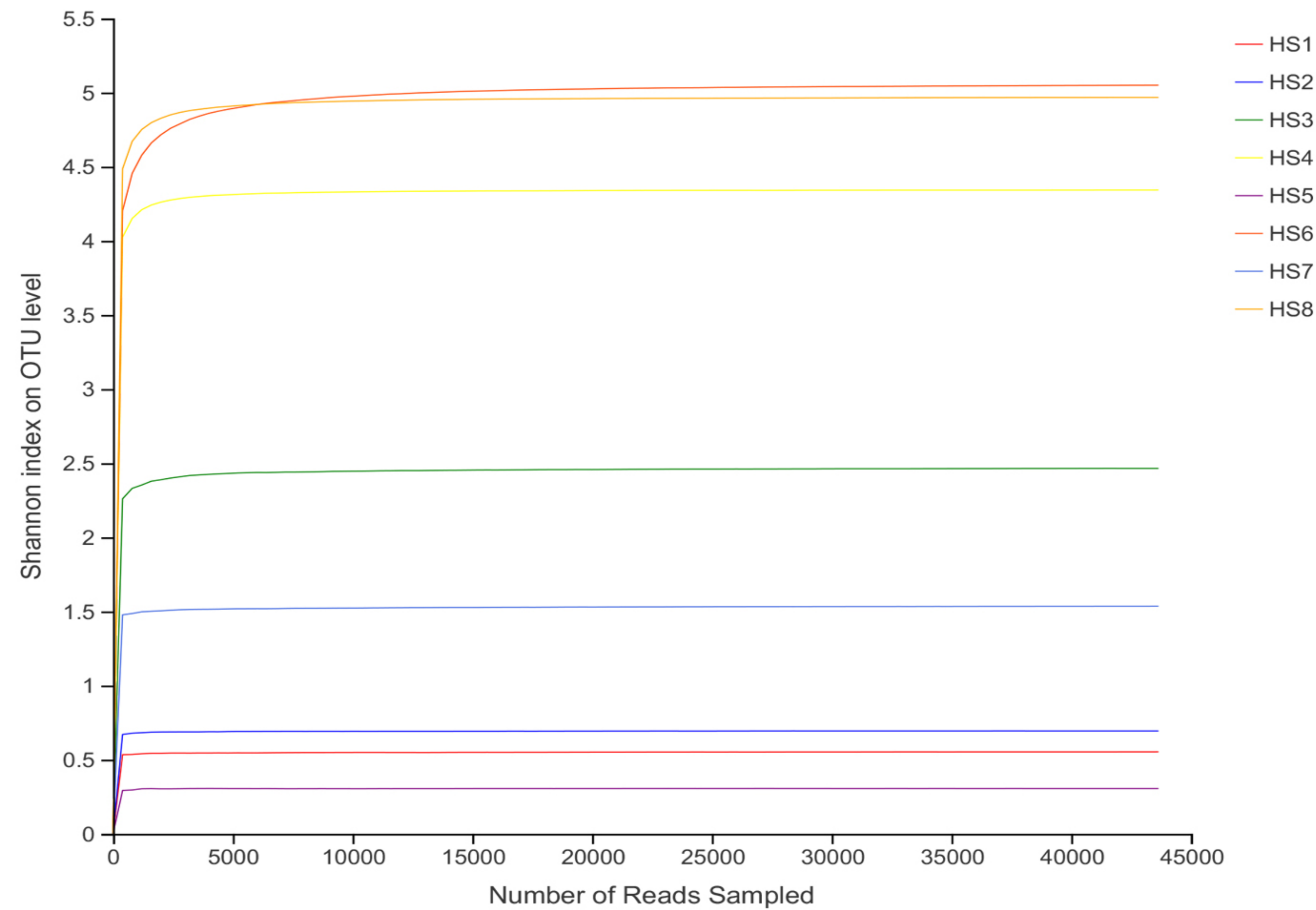

Supplement: Supplementary file 1 [file microorganisms-09-02260-s001.zip › Figure S1.pdf]

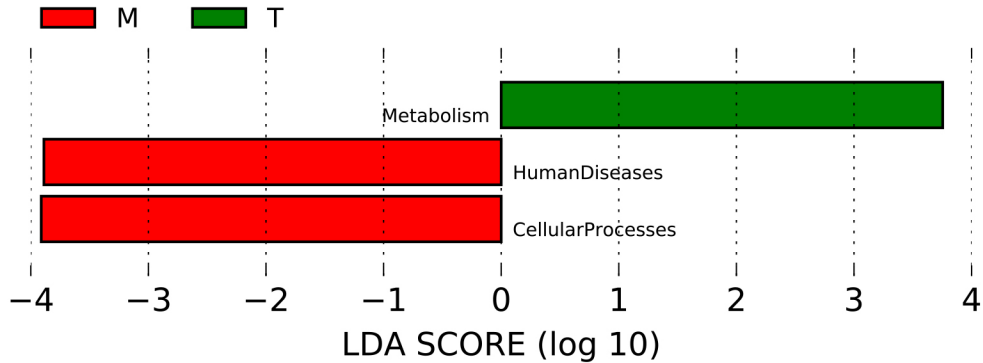

Supplement: Supplementary file 1 [file microorganisms-09-02260-s001.zip › Figure S2.pdf]
